# Looking for therapeutic antibodies in next-generation sequencing repositories

**DOI:** 10.1080/19420862.2019.1633884

**Published:** 2019-07-17

**Authors:** Konrad Krawczyk, Matthew I. J. Raybould, Aleksandr Kovaltsuk, Charlotte M. Deane

**Affiliations:** a Natural Antibody, Hamburg, Germany; b Department of Statistics, Oxford University, Oxford, UK

**Keywords:** Antibody therapeutics, next generation sequencing, patent, data mining

## Abstract

Recently it has become possible to query the great diversity of natural antibody repertoires using next-generation sequencing (NGS). These methods are capable of producing millions of sequences in a single experiment. Here we compare clinical-stage therapeutic antibodies to the ~1b sequences from 60 independent sequencing studies in the Observed Antibody Space database, which includes antibody sequences from NGS analysis of immunoglobulin gene repertoires. Of 242 post-Phase 1 antibodies, we found 16 with sequence identity matches of 95% or better for both heavy and light chains. There are also 54 perfect matches to therapeutic CDR-H3 regions in the NGS outputs, suggesting a nontrivial amount of convergence between naturally observed sequences and those developed artificially. This has potential implications for both the legal protection of commercial antibodies and the discovery of antibody therapeutics.

## Introduction

Antibodies are proteins found in jawed vertebrates that recognize noxious molecules (antigens) and aid in their elimination. An organism expresses millions of diverse antibodies to increase the chances that some of them will be able to bind the foreign antigen, initiating the adaptive immune response. This great diversity can now be queried using next-generation sequencing (NGS) of B-cell receptor repertoires, enabling the rapid collection of millions of antibody sequences from any given individual.^1–3^

The increasing volume of such NGS antibody depositions opens opportunities for alternative methods of therapeutic antibody discovery.^4^ Deep-learning methods are already being employed to data-mine the antibody repertoire for therapeutics.^5,6^ It is, however, unclear to what degree naturally-occurring antibodies are similar to those developed for therapeutic purposes. Contrasting therapeutic and naturally occurring antibodies could point to features that make safer biotherapeutics.^7^ Such large-scale comparisons could also have strategic implications for the pharmaceutical industry, as the sequence of a protein, such as an antibody, is one of the chief vehicles used to characterize the molecule in a patent.^8,9^ ‘Naturally occurring’ molecules, such as genomic or recombinant DNA, cannot be patented in the USA,^9,10^ raising questions as to what constitutes a ‘naturally occurring’ sequence for the purposes of legal protection.^11–13^ The large numbers of antibody sequences now becoming publicly available raises the possibility that naturally occurring sequences found via NGS are identical to commercial sequences.^10^

This is especially pertinent in the face of large-scale organized efforts to make naturally sourced antibody NGS data^14^ and analytics^15,16^ more accessible.^17^ Specifically, we recently created the Observed Antibody Space (OAS) database, which curates the NGS antibody data from public archives and makes them available for easy processing.^18^ OAS currently holds ~1b (~960 m heavy chain and ~60 m light chain) sequences from 60 independent studies. These datasets cover multiple organisms (primarily human, mouse, rhesus, rabbit, camel and rat), individuals and immune states. Here, we quantify how closely OAS sequences matched with current clinical stage-therapeutic (CST) antibody sequences.

## Results

**Figure 1.**
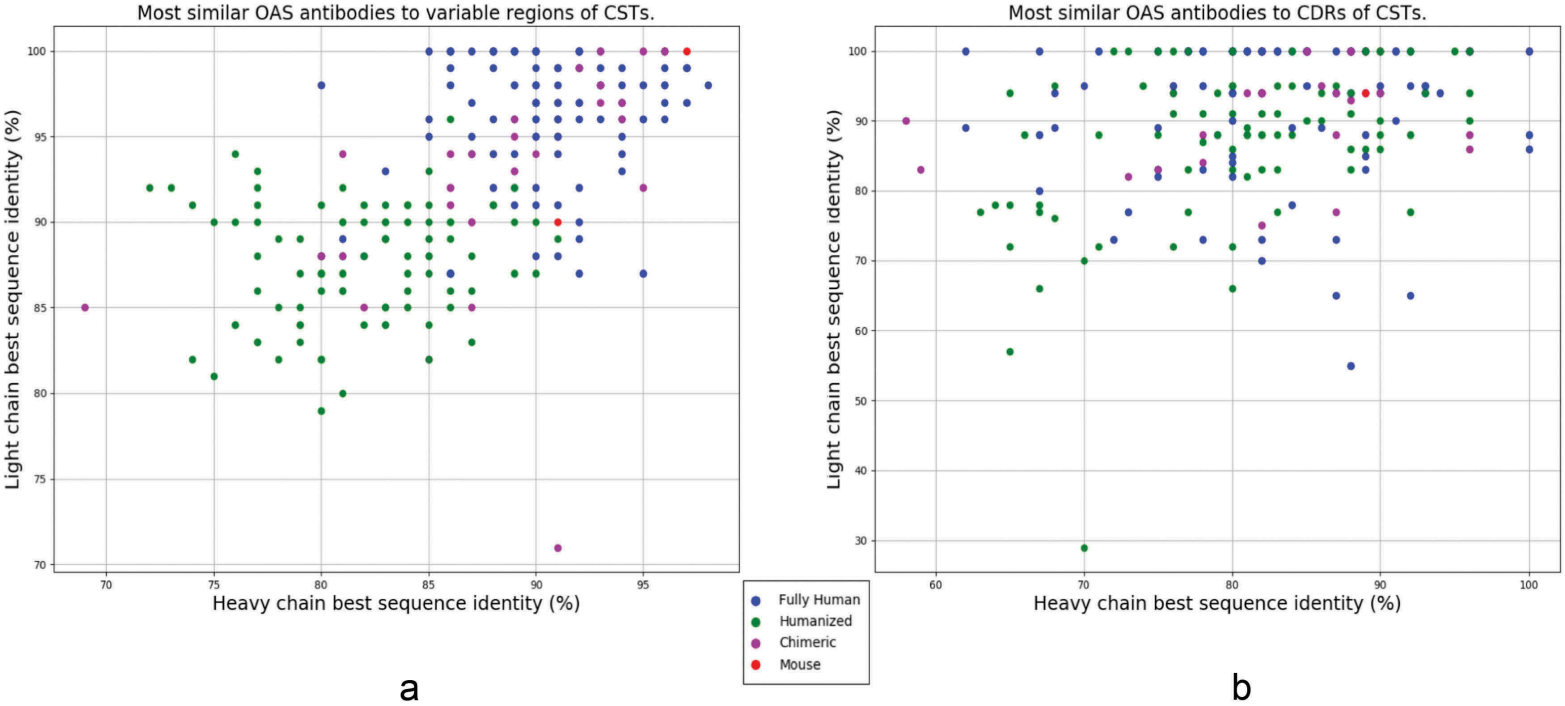
Best sequence identity matches to Clinical Stage Therapeutics (CST) in naturally sourced NGS datasets. (a) Heavy and light chain variable regions of 242 CST sequences from Raybould et al.^7^ aligned to variable region sequences in OAS.^18^ (b) Heavy and light chain IMGT CDR regions of 242 CSТs aligned to IMGT CDR regions in OAS. Fully human sequences are denoted by blue dots, humanized by green, chimeric by magenta and mouse in red. In small amount of cases where CSTs had the same identity values and different antibody type, we report the antibody type by majority vote of proximal CSTs. The precise alignment values can be found in Table 1 and their distributions in Figures 2 and 3. Interactive versions of these charts are available at http://naturalantibody.com/therapeutics.

**Figure 2.**
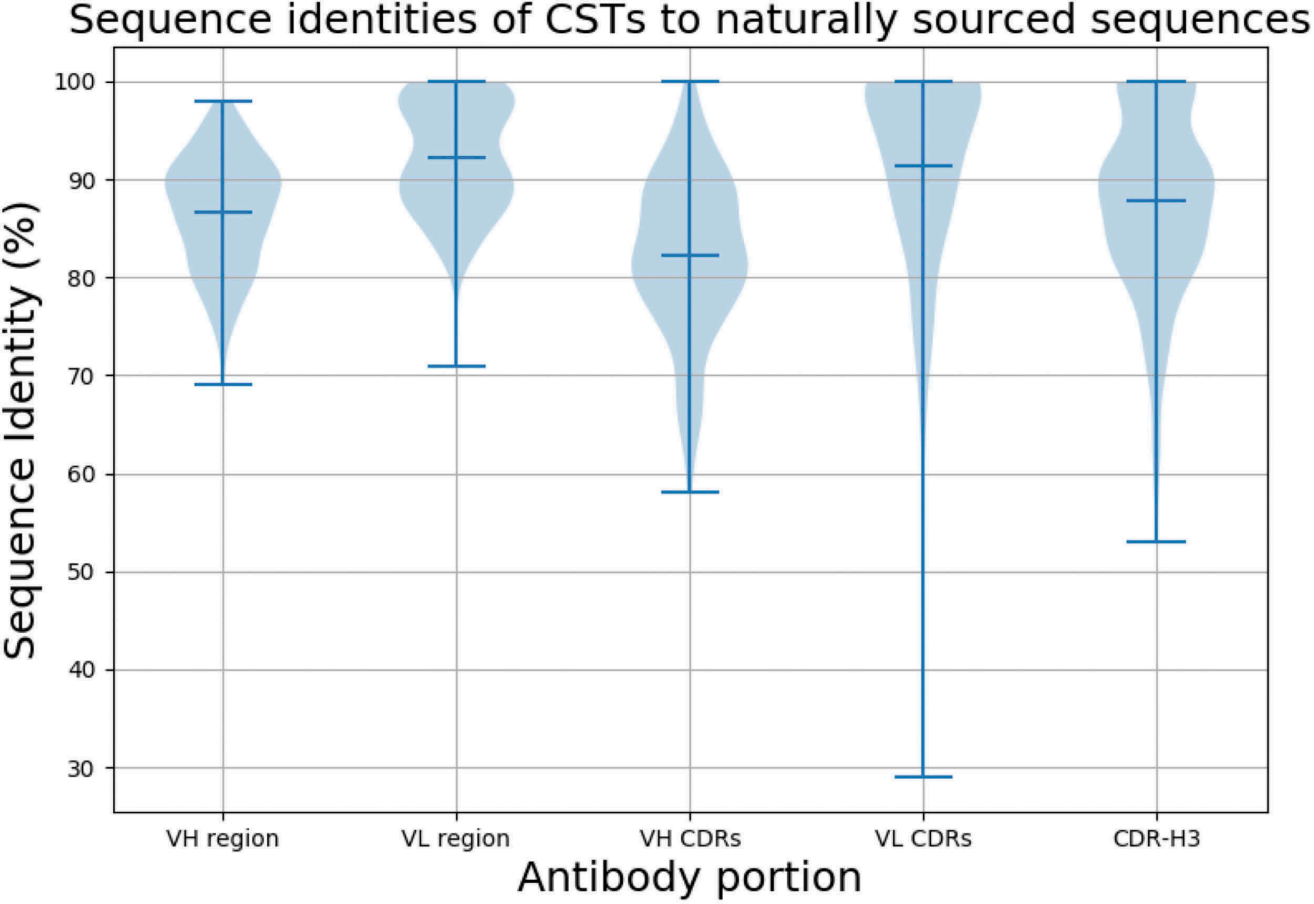
Distribution of sequence identity matches of Clinical Stage Therapeutics (CSTs) to naturally-sourced NGS. The violin plots show the distribution of sequence identities of the variable heavy (VH) and light (VL) chains, heavy and light CDR regions and CDR-H3 of CSTs to best matches in OAS.

We used a set of 242 CST antibody sequences,^7^ all of which have completed Phase 1 clinical trials. We separately aligned the CST variable regions (VH or VL), combination of the three complementarity-determining regions (CDRs) from VH or VL and CDR-H3s to all the sequences in OAS (see Methods). We performed the search across all organisms, individuals and immune states to be comprehensive and to reflect the myriad antibody types, including fully human, humanized, chimeric or fully mouse.^19^ The individual identities of the CSTs with respect to the best match from OAS are given in Figure 1 and Table 1, and their distributions are plotted in Figure 2. The aligned sequences are available in the Supplementary Material and on our website http://naturalantibody.com/therapeutics.

**Table 1.** Best sequence identities of Clinical Stage Therapeutic (CST) antibodies to sequences found in public NGS repositories. Sequence identities are given for the best alignment of a sequence from a public repository to a CST heavy or light chain variable region, heavy or light CDR region or CDR-H3 alone (IMGT-defined). The CSTs are identified by their names in the leftmost column. The entries are sorted from top to bottom by the highest heavy chain identity. An interactive version of this table together with aligned sequences are available at http://naturalantibody.com/therapeutics.

CST Name	Best Heavy Chain Identity (%)	Best Light Chain Identity (%)	Best Heavy Chain CDRs Identity (%)	Best Light Chain CDRs Identity (%)	Best CDR-H3 Identity (%)
*Enfortumab*	98	98	96	100	100
*Racotumomab*	97	100	90	100	92
*Tabalumab*	97	99	96	100	100
*Emapalumab*	97	99	93	95	87
*Tremelimumab*	97	97	94	94	88
*Ascrinvacumab*	96	100	96	100	100
*Derlotuximab*	96	100	89	100	92
*Zolbetuximab*	96	100	88	100	81
*Ganitumab*	96	99	92	100	91
*Rilotumumab*	96	98	93	94	100
*Durvalumab*	96	98	90	94	92
*Patritumab*	96	97	92	95	90
*Brazikumab*	96	96	90	95	94
*Carotuximab*	95	100	85	100	77
*Varlilumab*	95	98	89	100	91
*Brodalumab*	95	96	88	100	100
*Futuximab*	95	92	87	88	81
*Ramucirumab*	95	87	100	88	100
*Zanolimumab*	94	99	100	100	100
*Foravirumab*	94	98	89	100	100
*Dusigitumab*	94	97	100	86	100
*Rituximab*	94	97	90	94	85
*Muromonab*	94	97	82	100	83
*Ublituximab*	94	96	96	88	100
*Dectrekumab*	94	96	93	95	100
*Necitumumab*	94	95	93	94	92
*Cixutumumab*	94	94	89	85	82
*Fasinumab*	94	93	89	88	83
*Sifalimumab*	93	100	88	100	100
*Modotuximab*	93	100	82	100	91
*Golimumab*	93	99	88	94	94
*Brentuximab*	93	98	96	100	100
*Suvratoxumab*	93	98	87	94	87
*Zalutumumab*	93	98	85	100	88
*Bavituximab*	93	98	82	94	92
*Basiliximab*	93	97	88	93	90
*Radretumab*	93	96	80	84	100
*Ofatumumab*	92	100	90	100	93
*Bezlotoxumab*	92	100	89	100	91
*Daratumumab*	92	100	83	100	86
*Inclacumab*	92	100	75	100	88
*Siltuximab*	92	99	89	100	91
*Canakinumab*	92	99	85	100	100
*Lirilumab*	92	99	84	100	87
*Abrilumab*	92	97	85	100	90
*Tisotumab*	92	97	81	100	81
*Indusatumab*	92	96	82	100	84
*Carlumab*	92	92	82	70	83
*Tovetumab*	92	90	86	89	92
*Utomilumab*	92	89	88	55	100
*Tesidolumab*	92	87	92	65	100
*Glembatumumab*	91	99	92	100	100
*Ipilimumab*	91	99	88	100	90
*Iratumumab*	91	98	85	100	100
*Cetuximab*	91	97	82	94	92
*Burosumab*	91	97	80	94	90
*Anifrolumab*	91	96	84	89	90
*Pritoxaximab*	91	96	80	100	80
*Seribantumab*	91	95	78	95	83
*Girentuximab*	91	95	78	88	91
*Guselkumab*	91	94	80	82	90
*Lenzilumab*	91	91	78	83	83
*Abagovomab*	91	90	89	94	100
*Domagrozumab*	91	89	92	100	88
*Briakinumab*	91	88	87	65	75
*Otelixizumab*	91	71	82	75	83
*Intetumumab*	90	100	85	100	91
*Icrucumab*	90	100	82	100	78
*Foralumab*	90	100	81	100	90
*Fulranumab*	90	100	78	100	93
*Aducanumab*	90	100	78	100	88
*Sarilumab*	90	99	88	100	100
*Bleselumab*	90	98	80	100	84
*Tezepelumab*	90	98	80	100	80
*Opicinumab*	90	98	77	100	90
*Panitumumab*	90	97	89	94	90
*Tomuzotuximab*	90	97	82	94	92
*Timolumab*	90	97	80	100	100
*Adalimumab*	90	97	80	94	71
*Figitumumab*	90	96	91	100	88
*Evolocumab*	90	96	91	90	100
*Berlimatoxumab*	90	95	89	83	90
*Tralokinumab*	90	95	80	85	80
*Ensituximab*	90	94	81	94	85
*Anetumab*	90	92	82	73	84
*Setrusumab*	90	91	84	78	90
*Itolizumab*	90	90	82	88	83
*Ianalumab*	90	88	78	73	71
*Elotuzumab*	90	87	96	100	100
*Emibetuzumab*	90	87	87	94	100
*Evinacumab*	89	100	91	100	94
*Eldelumab*	89	100	81	100	94
*Nivolumab*	89	100	77	100	100
*Avelumab*	89	100	75	100	84
*Denosumab*	89	98	87	100	80
*Atidortoxumab*	89	98	67	88	83
*Setoxaximab*	89	96	85	100	91
*Drozitumab*	89	96	80	90	85
*Indatuximab*	89	95	87	94	100
*Tarextumab*	89	94	75	89	75
*Amatuximab*	89	93	82	94	100
*Infliximab*	89	93	75	83	90
*Lorvatuzumab*	89	92	88	86	100
*Bimagrumab*	89	92	87	73	100
*Solanezumab*	89	92	80	91	100
*Mavrilimumab*	89	91	72	73	61
*Camrelizumab*	89	90	92	88	100
*Tigatuzumab*	89	87	89	100	83
*Anrukinzumab*	89	87	85	90	91
*Urelumab*	88	100	80	100	86
*Secukinumab*	88	100	80	100	80
*Olaratumab*	88	100	77	100	78
*Erenumab*	88	99	71	100	82
*Alirocumab*	88	96	85	95	90
*Gantenerumab*	88	94	68	89	63
*Orticumab*	88	92	73	77	78
*Crenezumab*	88	91	95	100	100
*Concizumab*	88	91	80	95	85
*Bapineuzumab*	88	91	75	100	83
*Actoxumab*	87	100	83	100	86
*Dupilumab*	87	97	76	95	72
*Rafivirumab*	87	95	75	83	70
*Margetuximab*	87	94	82	94	84
*Trevogrumab*	87	94	79	88	69
*Dinutuximab*	87	90	86	95	83
*Mirvetuximab*	87	90	77	100	90
*Olendalizumab*	87	88	75	100	92
*Quilizumab*	87	86	88	91	100
*Obiltoxaximab*	87	85	100	100	100
*Lampalizumab*	87	83	79	94	75
*Pamrevlumab*	86	100	82	100	92
*Fletikumab*	86	100	80	100	85
*Lanadelumab*	86	100	67	100	73
*Ustekinumab*	86	99	78	100	83
*Teprotumumab*	86	98	85	100	90
*Refanezumab*	86	96	80	100	73
*Galiximab*	86	94	58	90	63
*Coltuximab*	86	92	96	86	100
*Ibalizumab*	86	92	87	95	80
*Isatuximab*	86	91	89	94	92
*Otlertuzumab*	86	90	92	77	88
*Rovalpituzumab*	86	90	88	94	90
*Landogrozumab*	86	89	81	89	100
*Daclizumab*	86	87	92	88	100
*Etaracizumab*	86	87	84	88	90
*Enokizumab*	86	87	80	72	86
*Robatumumab*	86	87	77	100	91
*Tislelizumab*	86	86	88	83	91
*Lacnotuzumab*	86	85	88	94	90
*Panobacumab*	85	100	84	100	80
*Fezakinumab*	85	96	70	95	71
*Fresolimumab*	85	95	62	89	84
*Romosozumab*	85	93	84	100	81
*Dalotuzumab*	85	91	80	100	90
*Imgatuzumab*	85	90	68	76	92
*Bococizumab*	85	89	77	83	81
*Atezolizumab*	85	89	77	77	90
*Visilizumab*	85	88	89	100	100
*Lodelcizumab*	85	88	70	70	90
*Lintuzumab*	85	87	96	100	100
*Bimekizumab*	85	84	67	66	66
*Veltuzumab*	85	82	90	94	92
*Rozanolixizumab*	85	82	73	82	80
*Codrituzumab*	84	91	83	91	87
*Plozalizumab*	84	91	73	100	87
*Simtuzumab*	84	90	92	100	100
*Mogamulizumab*	84	88	67	78	75
*Tildrakizumab*	84	87	92	100	100
*Gevokizumab*	84	86	79	88	75
*Sacituzumab*	84	85	96	94	100
*Gedivumab*	83	93	67	80	55
*Obinutuzumab*	83	91	78	100	83
*Ozanezumab*	83	90	90	100	83
*Ixekizumab*	83	90	78	91	75
*Abituzumab*	83	89	85	100	90
*Trastuzumab*	83	89	82	94	84
*Etrolizumab*	83	89	76	72	100
*Ponezumab*	83	89	64	78	77
*Matuzumab*	83	85	83	88	92
*Motavizumab*	83	85	75	88	83
*Inebilizumab*	83	84	90	90	92
*Lifastuzumab*	83	84	65	78	76
*Tanezumab*	82	91	80	83	86
*Olokizumab*	82	90	65	72	81
*Ocrelizumab*	82	88	93	94	93
*Sirukumab*	82	88	75	82	83
*Andecaliximab*	82	85	87	77	100
*Palivizumab*	82	84	86	94	100
*Lumiliximab*	81	94	59	83	88
*Tocilizumab*	81	92	82	100	83
*Galcanezumab*	81	90	75	83	83
*Duligotuzumab*	81	90	63	77	78
*Roledumab*	81	89	68	94	73
*Vadastuximab*	81	88	88	100	100
*Vedolizumab*	81	88	86	95	85
*Mirikizumab*	81	88	83	77	87
*Natalizumab*	81	87	90	100	100
*Eculizumab*	81	87	83	100	86
*Pinatuzumab*	81	86	89	86	100
*Ficlatuzumab*	81	86	81	88	90
*Eptinezumab*	81	80	70	29	100
*Belimumab*	80	98	62	100	62
*Crizanlizumab*	80	91	90	86	93
*Depatuxizumab*	80	88	76	94	88
*Pertuzumab*	80	88	75	83	91
*Ligelizumab*	80	88	71	88	81
*Blosozumab*	80	88	66	88	81
*Ravulizumab*	80	87	77	100	86
*Fremanezumab*	80	87	67	77	53
*Clazakizumab*	80	87	65	57	78
*Pembrolizumab*	80	86	86	90	84
*Inotuzumab*	80	82	80	95	100
*Pidilizumab*	80	82	76	94	90
*Vatelizumab*	80	79	82	88	92
*Benralizumab*	79	89	83	83	71
*Certolizumab*	79	87	81	100	100
*Lebrikizumab*	79	85	74	95	91
*Epratuzumab*	79	84	84	95	88
*Satralizumab*	79	84	71	72	83
*Risankizumab*	79	83	82	83	84
*Reslizumab*	78	89	92	77	100
*Onartuzumab*	78	85	78	87	75
*Farletuzumab*	78	82	96	90	100
*Bevacizumab*	77	93	90	88	93
*Vonlerolizumab*	77	92	65	94	80
*Idarucizumab*	77	91	83	95	87
*Polatuzumab*	77	90	80	95	80
*Rontalizumab*	77	88	76	95	90
*Parsatuzumab*	77	86	81	82	93
*Gemtuzumab*	77	83	80	86	88
*Spartalizumab*	77	83	76	91	90
*Efalizumab*	76	94	83	100	85
*Alemtuzumab*	76	90	80	66	91
*Dacetuzumab*	76	84	82	91	85
*Tregalizumab*	76	84	72	100	93
*Omalizumab*	75	90	76	100	71
*Nimotuzumab*	75	81	68	95	62
*Pateclizumab*	74	91	81	88	81
*Teplizumab*	74	82	82	100	83
*Ranibizumab*	73	92	81	88	93
*Mepolizumab*	72	92	78	95	84
*Ontuxizumab*	69	85	78	84	82

**Figure 3.**
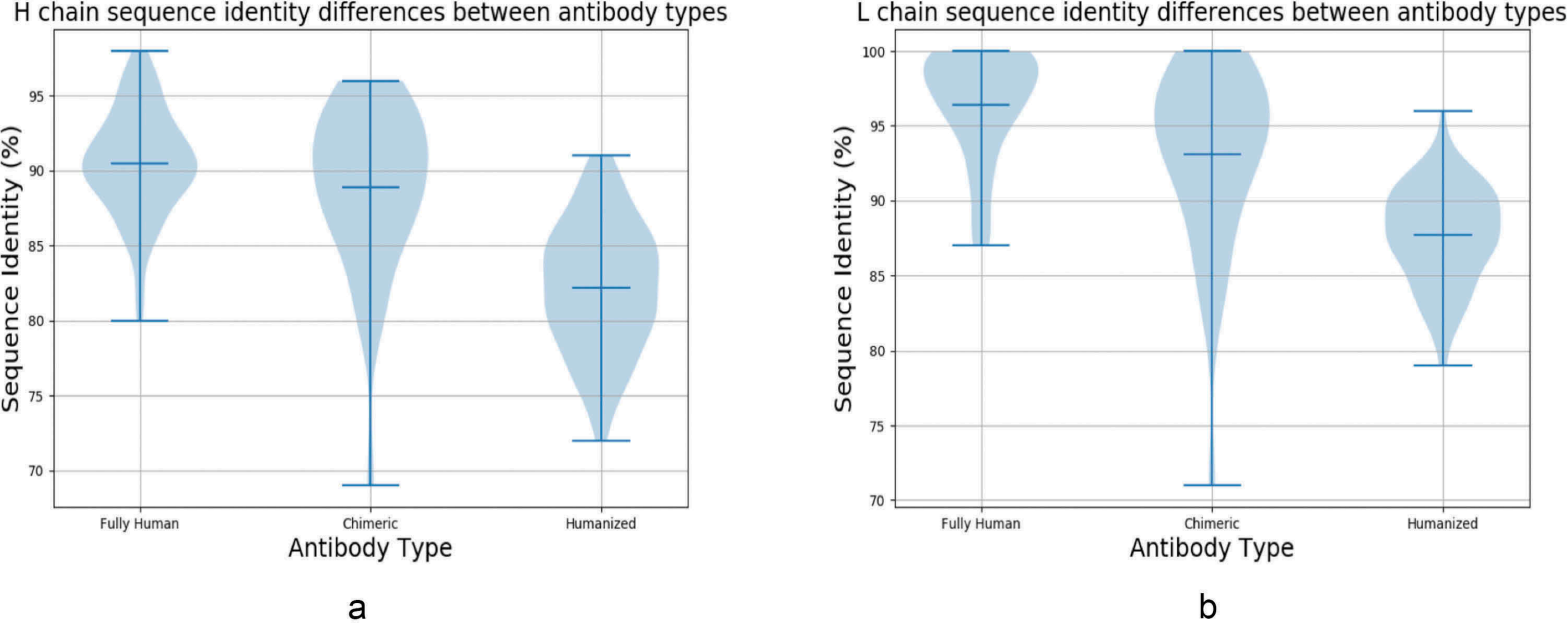
Sequence identity matches of Clinical Stage Therapeutic (CST) variable regions to naturally sourced NGS datasets stratified by CST antibody type. CST a) heavy chain and b) light chain identities to NGS sequences in OAS stratified by fully human, chimeric and humanized antibody types. The three mouse molecules were omitted as too small a sample.

## Analysis of clinical-stage therapeutic sequence matches to naturally sourced NGS datasets

The best sequence identity matches of CST variable regions to naturally sourced NGS datasets in OAS are given in Figure 1(a). Ninety (37.1%) CST heavy chains have matches within OAS of ≥ 90% sequence identity (seqID), with 18 (7.4%) ≥ 95% seqID. We find 158 (65.2%) therapeutic light chains with ≥ 90% seqID to an OAS sequence, with 96 (39.7%) ≥ 95% seqID, and 28 (11.5%) with 100% seqID. For 16 (6.6%) of the CSTs, we find both heavy and light chain matches ≥ 95% seqID. In the most extreme case, enfortumab, we were able to find both heavy and light chain matches of 98% seqID (the differences are H38:N-S, H88:S-Y, L37:G-S, L52:F-L, where the first amino acid comes from enfortumab and the second from an OAS sequence).

The largest discrepancy between the CSTs and OAS antibodies is typically concentrated in the CDR regions that determine antigen complementarity.^20^ It remains unclear, however, the extent to which the highly mutable CDR loops of engineered therapeutics differ from those that are expressed naturally. We searched for the best CST matches to the CDR regions in OAS. The sequence identity was calculated across the entire CDR region testing if all three CDR lengths matched between the CST and an NGS sequence. The search was performed using the international ImMunoGeneTics information system® (IMGT)-defined CDR triplets from the heavy or light chain, disregarding the framework region (i.e., we concatenated sequences of the CDRH1-3 loops, or CDRL1-3 loops; Table 1, Figures 1(b), and 2). We find 46 (19.0%) of CST heavy chain CDR triplets to have matches to an OAS CDR triplet with ≥ 90% seqID, 15 (6.1%) with ≥ 95% seqID and 4 (1.6%) with 100% seqID. There were 156 (64.4%) CST light CDR triplets with ≥ 90% seqID to an OAS CDR triplet, with 110 (45.4%) ≥ 95% seqID, and 90 (37.1%) with 100% seqID. For obiltoxaximab and zanolimumab, we found NGS sequences where all three heavy and light chain CDRs were identical.

Of the six CDRs, CDR-H3 is the most sequence and structurally diverse.^21,22^ Due to its key role in binding, it is subjected to extensive antibody engineering.^23,24^ We checked how likely it is to find CST-derived CDR-H3s in naturally sourced sequences. To assess this, we searched for the best CST CDR-H3 matches in OAS, regardless of the framework region and remaining CDRs (Table 1, Figure 2). Of our 242 CST CDR-H3s, we found 54 perfect matches in OAS. The perfect matches tended to be for shorter CDR-H3s, but some longer loops with perfect matches were also found (see Supplementary Section 1). We note that finding such good matches is highly unlikely by chance alone even accounting for sequencing errors, as described in Supplementary Section 1. Twenty-nine perfect matches were found in just one recent deep sequencing study of Briney et al.^3^ This study sampled the diversity of the human antibody gene repertoires of 10 individuals on an unprecedented depth. The large proportions of matches from this single study suggest that substantial CDR-H3 diversity can be found in a very limited number of individuals. Forty-seven perfect matches were found in OAS datasets other than that of Briney et al., showing that certain artificial CDR-H3 sequences can be independently observed in naturally sourced NGS. Twenty-two CDR-H3 matches were found in both Briney et al. data and other OAS datasets. These 22 shared sequences come from 9 humanized and 13 fully human CSTs. The 54 perfect CDR-H3 matches were distributed among all antibody types, with 23 humanized, 22 fully human, 8 chimeric and 1 mouse (21.9%, 22.0%, 22.8% and 50.0% of each category, respectively). These results show that, despite the large theoretical sequence space accessible to the CDR-H3 region,^3^ therapeutically exploitable CDR-H3 loops are found in just ~960 m heavy chain sequences from 60 NGS studies (see Supplementary Section 2). This convergence, coupled with the fact that CDR-H3 loops often mediate antibody specificity^25^ and binding affinity, could suggest intrinsically driven biases in antigen recognition,^26^ independent of artificial discovery methods.

## Stratifying the best CST matches in OAS by antibody type

The quality of the variable region match we could find for any given CST sequence appears to be highly dependent on the discovery platform/antibody type. Figure 3 suggests that antibodies produced via more artificial protocols such as humanization have lower variable region sequence identities to sequences in OAS from those of fully human molecules. For the majority of the fully human sequences we find matches of 90% seqID or better, whereas matches to the majority of humanized molecules fall below 90% seqID (Figure 3). Chimeric antibodies appear to have seqID values intermediate between the two classes (Figure 3).

The CST antibody type also reflects the organism that produced the best NGS seqID match. Of the 100 fully human CSTs, the 90 (90.0%) most similar heavy chains, 100 (100.0%) most similar light chains, and 55 (55.0%) most similar CDR-H3 loops come from human-sourced NGS. Of the 105 humanized antibodies, 82 (78.0%) of heavy chains, and 79 (75.2%) of light chains found closest matches in human-sourced NGS, while 71 (67.6%) of the best CDR-H3s matches were identified in mouse-sourced NGS. This further reflects the dominance of CDR-H3 in binding, as companies often graft this loop from binding mouse antibodies to transfer specificity and binding affinity. It also suggests that mining a dataset such as OAS could provide a more accurate measure of antibody ‘humanness’ than our current metrics.^27,28^

## Discussion

Our results demonstrate that, despite the theoretically large diversity accessible to antibodies,^3,29^ there exists a nontrivial convergence between artificially developed CSTs and naturally sourced NGS sequences. The closest NGS matches to CSTs were sourced from 48 of the 60 (80.0%) independent studies available in OAS, indicating that finding a close match to at least one CST is likely in most NGS datasets.

It was previously suggested that such an overlap could cause issues in patenting therapeutic antibodies.^10^ The amount of antibody NGS sequences becoming available creates a larger volume of prior art that might have to be taken into consideration when patenting a novel molecule. Firstly, a molecule’s sequence is a primary characteristic in any patent claim, but only in conjunction with a particular binding mode and/or therapeutic action.^8^ While NGS studies produce copious numbers of sequences, they do not alone relate them to any target molecule and it is unclear whether eliciting antibodies to vaccines or other delivered immunogens would be regarded as artificial or “naturally occurring”. Secondly, the antibody variable region is a product of two polypeptide chains (heavy and light) and its function is intimately related to this combination. Currently, the majority of available NGS datasets report heavy and light chains separately and OAS only contains the unpaired chains. As paired NGS technology becomes more sophisticated, it can be expected to provide a more comprehensive view of the convergence between naturally sourced and artificially developed sequences.^2,30,31^ Thirdly, artificial nucleotide mutations can be introduced at random to antibody sequences by NGS techniques as well as during DNA sample preparation.^32^ Lastly, it is unclear how close a sequence-identity match to a publicly available sequence (or important portion thereof, such as CDR-H3) would cause issues in establishing the inventiveness of a sequence. For instance, only four pairs of CSTs have heavy chain sequence identity matches of greater than 94% to each other (see Supplementary Section 3). In three of the pairs, both sequences originate from the same company while the fourth is the original patent-expired antibody and its derivative. This compares to 18 therapeutic heavy chains with matches to OAS better than 95%. Our findings offer a quantitative basis for discussions regarding patentability of antibodies,^10^ and also may have potentially wider implications for therapeutic antibody discovery. Appreciating the relatedness between engineered antibodies and their naturally expressed counterparts should facilitate the selection of better candidate biotherapeutics, assuming that those that are more closely related have more favorable biophysical properties.^7^ This assertion could be tested by investigating the covariance of important clinical indicators, such as affinity, immunogenicity and solubility, with measures of similarity to naturally occurring antibodies. Furthermore, bespoke analysis of NGS matches that came from immunized datasets and the corresponding CST targets could shed light on the mechanics of the immune recognition. The close overlap we report between therapeutic and natural sequence space suggests that it should be possible to data-mine naturally sourced NGS repositories for promising therapeutic leads.^4^

In light of ongoing efforts to further consolidate antibody NGS data and make it more accessible, it follows that finding therapeutic candidate sequences in published NGS datasets will become easier.^17,33^

## Methods

We used the Observed Antibody Space database as the source of NGS sequences. Since its first release, the database has been expanded by four datasets, most notably the recent deep sequencing of human antibody repertoire by Briney et al., as reported in 2019.^3^ We employed the processed consensus sequences from Briney et al., removing any sequences that ANARCI, which is a tool for numbering amino-acid sequences of antibody and T-cell receptor variable domains, deemed were unproductive.^34^ All the sequences in OAS originate from studies where the heavy and light chain are separated.

We used the 242 antibodies from Raybould et al.^7^ as the source of CST antibodies. We numbered the CST sequences according to the IMGT^35^ scheme using ANARCI.^34^ The CST sequences were classified into four groups (chimeric, humanized, human, mouse), based on their international nonproprietary names.^20,36^ Sequences with names containing ‘-xizumab’ or ‘-ximab’ were labeled as ‘chimeric’. Sequences not matching this criterion but containing ‘-zumab’ in their name were classified as ‘humanized’. Sequences that contained only ‘-umab’ in their name were labeled as ‘fully human’. Three mouse antibodies (muromonab, abagovomab and racotumomab), were labeled as ’mouse’.

We separately aligned the heavy chain, light chain, the combination of the three heavy or light chains IMGT-defined CDRs and the IMGT-defined CDR-H3 of CSTs to each of the sequences in OAS.^18^ We note a match if an IMGT position in a ‘query’ CST is also found in a ‘template’ sequence from OAS, and they have the same amino acid residue. For the full sequence alignments, the number of matches is divided by the length of the query and by the length of the template, producing two sequence identities. The final sequence identity is the average between these two. Calculating the sequence identity in this way prevents the scenario when one sequence is a substring of another, creating an artificially high sequence identity with a large length discrepancy. The CDR alignments were performed when the IMGT-defined loop lengths matched. The aligned sequences are available in the supplementary section 4 and through an interactive version of Figure 1 and Table 1 accessible at http://naturalantibody.com/therapeutics.

## References

[1] Miho E, Yermanos A, Weber CR, Berger CT, Reddy ST, Greiff V. Computational strategies for dissecting the high-dimensional complexity of adaptive immune repertoires. Front Immunol. 2018;9:224. doi:10.3389/fimmu.2018.00224 PMID: 29515569. 29515569 PMC5826328

[2] Georgiou G, Ippolito GC, Beausang J, Busse CE, Wardemann H, Quake SR The promise and challenge of high-throughput sequencing of the antibody repertoire. Nat Biotechnol. 2014;32(2):158–68. doi:10.1038/nbt.2782 PMID: 24441474. 24441474 PMC4113560

[3] Briney B, Inderbitzin A, Joyce C, Burton DR Commonality despite exceptional diversity in the baseline human antibody repertoire. Nature. 2019;566:393–97. doi:10.1038/s41586-019-0879-y PMID: 30664748. 30664748 PMC6411386

[4] Raybould MIJ, Wong WK, Deane CM Antibody–antigen complex modelling in the era of immunoglobulin repertoire sequencing. Mol Syst Des Eng. 2019 Advance Article. doi:10.1039/C9ME00034H.

[5] Mason DM, Friedensohn S, Weber CR, Jordi C, Wagner B, Meng S, Reddy ST Deep learning enables therapeutic antibody optimization in mammalian cells. bioRxiv. 2019. doi:10.1101/617860.

[6] Miho E, Roškar R, Greiff V, Reddy ST Large-scale network analysis reveals the sequence space architecture of antibody repertoires. Nat Commun. 2019;10(1):1321. doi:10.1038/s41467-019-09278-8 PMID: 30899025. 30899025 PMC6428871

[7] Raybould MIJ, Marks C, Krawczyk K, Taddese B, Nowak J, Lewis AP, Bujotzek A, Shi J, Deane CM Five computational developability guidelines for therapeutic antibody profiling. Proc Natl Acad Sci USA. 2019;116(10):4025–30. doi:10.1073/pnas.1810576116 PMID: 30765520. PMC6410772 30765520

[8] Germinario C, Bertoli S, Rampinelli P, Cini M Patentability of antibodies for therapeutic use in Europe. Nat Biotechnol. 2018;36(5):402–05. doi:10.1038/nbt.4134 PMID: 29734309. 29734309

[9] Association for Molecular Pathology v. Myriad genetics, Inc. (Supreme court of the United States [No. 12-398]). Biotechnol Law Rep. 2013;32. doi:10.1089/blr.2013.9877.

[10] Ponraj P Next-generation sequencing may challenge antibody patent claims. Nature. 2018;557(7704):166. doi:10.1038/d41586-018-05065-5 PMID: 29743696. 29743696

[11] Harrison C Patent watch. Nat Rev Drug Discov. 2012;11:344–45. doi:10.1038/nrd3735. 22212669

[12] Harrison C Isolated DNA patent ban creates muddy waters for biomarkers and natural products. Nat Rev Drug Discov. 2013;12(8):570–71. doi:10.1038/nrd4084 PMID: 23903213. 23903213

[13] Aboy M, Crespo C, Liddell K, Liddicoat J, Jordan M Was the Myriad decision a “surgical strike” on isolated DNA patents, or does it have wider impacts? Nat Biotechnol. 2018;36(12):1146–49. doi:10.1038/nbt.4308 PMID: 30520866. 30520866

[14] Cowell LG VDJServer: a cloud-based analysis portal and data commons for immune repertoire sequences and rearrangements. Front Immunol. 2018;9:976. doi:10.3389/fimmu.2018.00976 PMID: 29867956. 29867956 PMC5953328

[15] Krawczyk K, Kelm S, Kovaltsuk A, Galson JD, Kelly D, Trück J, Regep C, Leem J, Wong WK, Nowak J, et al Structurally mapping antibody repertoires. Front Immunol. 2018;9:1698. doi:10.3389/fimmu.2018.01698 PMID: 30083160. 30083160 PMC6064724

[16] López-Santibáñez-Jácome L, Avendaño-Vázquez SE, Flores-Jasso CF The pipeline repertoire of Ig-Seq analysis. PeerJ. 2018. doi:10.7287/peerj.preprints.27444v1. PMC6503734 31114573

[17] Rubelt F, Busse CE, Bukhari SAC, Bürckert JP, Mariotti-Ferrandiz E, Cowell LG, Watson CT, Marthandan N, Faison WJ, Hershberg U, et al Adaptive immune receptor repertoire community recommendations for sharing immune-repertoire sequencing data. Nat Immunol. 2017;18(12):1274–78. doi:10.1038/ni.3873 PMID: 29144493. 29144493 PMC5790180

[18] Kovaltsuk A, Leem J, Kelm S, Snowden J, Deane CM, Krawczyk K Observed antibody space: a resource for data mining next-generation sequencing of antibody repertoires. J Immunol. 2018;201(8):2502–09. doi:10.4049/jimmunol.1800708 PMID: 30217829. 30217829

[19] Jain T, Sun T, Durand S, Hall A, Houston NR, Nett JH, Sharkey B, Bobrowicz B, Caffry I, Yu Y, et al Biophysical properties of the clinical-stage antibody landscape. Proc Natl Acad Sci USA. 2017;114(5):944–49. doi:10.1073/pnas.1616408114 PMID: 28096333. 28096333 PMC5293111

[20] Sela-Culang I, Kunik V, Ofran Y The structural basis of antibody-antigen recognition. Front Immunol. 2013;4:302. doi:10.3389/fimmu.2013.00302 PMID: 24115948. 24115948 PMC3792396

[21] MacCallum RM, Martin AC, Thornton JM Antibody-antigen interactions: contact analysis and binding site topography. J Mol Biol. 1996;262(5):732–45. doi:10.1006/jmbi.1996.0548 PMID: 8876650. 8876650

[22] Krawczyk K, Dunbar J, Deane CM Computational tools for aiding rational antibody design. Methods Mol Biol. 2017;1529:399–416. doi:10.1007/978-1-4939-6637-0_21 PMID: 27914064. 27914064

[23] Knappik A, Ge L, Honegger A, Pack P, Fischer M, Wellnhofer G, Hoess A, Wölle J, Plückthun A, Virnekäs B Fully synthetic human combinatorial antibody libraries (HuCAL) based on modular consensus frameworks and CDRs randomized with trinucleotides. J Mol Biol. 2000;296(1):57–86. doi:10.1006/jmbi.1999.3444 PMID: 10656818. 10656818

[24] De Kruif J, Boel E, Logtenberg T Selection and application of human single chain Fv antibody fragments from a semi-synthetic phage antibody display library with designed CDR3 regions. J Mol Biol. 1995;248(1):97–105. doi:10.1006/jmbi.1995.0204 PMID: 7731047. 7731047

[25] Tsuchiya Y, Mizuguchi K The diversity of H3 loops determines the antigen-binding tendencies of antibody CDR loops. Protein Sci. 2016;25(4):815–25. doi:10.1002/pro.2874 PMID: 26749247. 26749247 PMC4941225

[26] Morea V, Tramontano A, Rustici M, Chothia C, Lesk AM Conformations of the third hypervariable region in the VH domain of immunoglobulins. J Mol Biol. 1998;275(2):269–94. doi:10.1006/jmbi.1997.1442 PMID: 9466909. 9466909

[27] Abhinandan KR, Martin ACR Analyzing the “Degree of humanness” of antibody sequences. J Mol Biol. 2007;369(3):852–62. doi:10.1016/j.jmb.2007.02.100 PMID: 17442342. 17442342

[28] Choi Y, Hua C, Sentman CL, Ackerman ME, Bailey-Kellogg C Antibody humanization by structure-based computational protein design. MAbs. 2015;7(6):1045–57. doi:10.1080/19420862.2015.1076600 PMID: 26252731. 26252731 PMC5045135

[29] Glanville J, Zhai W, Berka J, Telman D, Huerta G, Mehta GR, Ni I, Mei L, Sundar PD, Day GMR, et al Precise determination of the diversity of a combinatorial antibody library gives insight into the human immunoglobulin repertoire. Proc Natl Acad Sci USA. 2009;106(48):20216–21. doi:10.1073/pnas.0909775106 PMID: 19875695. 19875695 PMC2787155

[30] DeKosky BJ, Ippolito GC, Deschner RP, Lavinder JJ, Wine Y, Rawlings BM, Varadarajan N, Giesecke C, Dörner T, Andrews SF, et al High-throughput sequencing of the paired human immunoglobulin heavy and light chain repertoire. Nat Biotechnol. 2013;31(2):166–69. doi:10.1038/nbt.2492 PMID: 23334449. 23334449 PMC3910347

[31] DeKosky BJ, Lungu OI, Park D, Johnson EL, Charab W, Chrysostomou C, Kuroda D, Ellington AD, Ippolito GC, Gray JJ, et al Large-scale sequence and structural comparisons of human naive and antigen-experienced antibody repertoires. Proc Natl Acad Sci USA. 2016;113(19):E2636–45. doi:10.1073/pnas.1525510113 PMID: 27114511. 27114511 PMC4868480

[32] Kovaltsuk A, Krawczyk K, Kelm S, Snowden J, Deane CM Filtering next-generation sequencing of the Ig gene repertoire data using antibody structural information. J Immunol. 2018;201(12):3694–704. doi:10.4049/jimmunol.1800669 PMID: 30397033. 30397033 PMC6485405

[33] Breden F, Luning Prak ET, Peters B, Rubelt F, Schramm CA, Busse CE, Vander Heiden JA, Christley S, Bukhari SAC, Thorogood A, et al Reproducibility and reuse of adaptive immune receptor repertoire data. Front Immunol. 2017;8:1418. doi:10.3389/fimmu.2017.01418 PMID: 29163494. 29163494 PMC5671925

[34] Dunbar J, Deane CM ANARCI: antigen receptor numbering and receptor classification. Bioinformatics. 2015;32(2):298–300. doi:10.1093/bioinformatics/btv552 PMID: 26424857. 26424857 PMC4708101

[35] Lefranc M, Pommié C, Ruiz M, Giudicelli V, Foulquier E, Truong L, Thouvenin-Contet V, Lefranc G IMGT unique numbering for immunoglobulin and T cell receptor variable domains and Ig superfamily V-like domains. Dev Comp Immunol. 2003;27:55–77. doi:10.1016/S0145-305X(02)00039-3 PMID: 12477501. 12477501

[36] Jones TD, Carter PJ, Plückthun A, Vásquez M, Holgate RGE, Hötzel I, Popplewell AG, Parren PWHI, Enzelberger M, Rademaker HJ, et al The INNs and outs of antibody nonproprietary names. MAbs. 2016;8(1):1–9. doi:10.1080/19420862.2015.1114320 PMID: 26716992. 26716992 PMC4966553

