# Supplementary material for: Looking for therapeutic antibodies in next-generation sequencing repositories
Source: MAbs. 2019 Jul 17;11(7):1197–205. doi: 10.1080/19420862.2019.1633884 (PMC6748601; doi:10.1080/19420862.2019.1633884)
Supplement: Supplemental Material [file kmab-11-07-1633884-s001.docx]

**Supplementary tables and figures**

**Section 1. Distribution of CDR-H3 lengths in CSTs as compared to perfect matches in OAS.**

We found 54 perfect matches to CST CDR-H3s in OAS. We compared the CDR-H3 lengths of the perfect matches to all CDR-H3 lengths of our 242 CSTs (Supplementary Figure 1 and Supplementary Table 1). The mean length of all CST CDR-H3 is 12 whereas this of the 54 perfect matches is 10. As expected, it is easier to find perfect matches for shorter CDR-H3s, however some longer lengths were also covered (Supplementary Figure 1 and Supplementary Table 1).

Of the 54 perfect matches, 22 can be found in the deep sequencing dataset of Briney et al. 2019 and other OAS datasets. The mean length of these shared CDR-H3s is 9, further indicating that finding perfect matches across independent datasets is easier for shorter loop lengths.


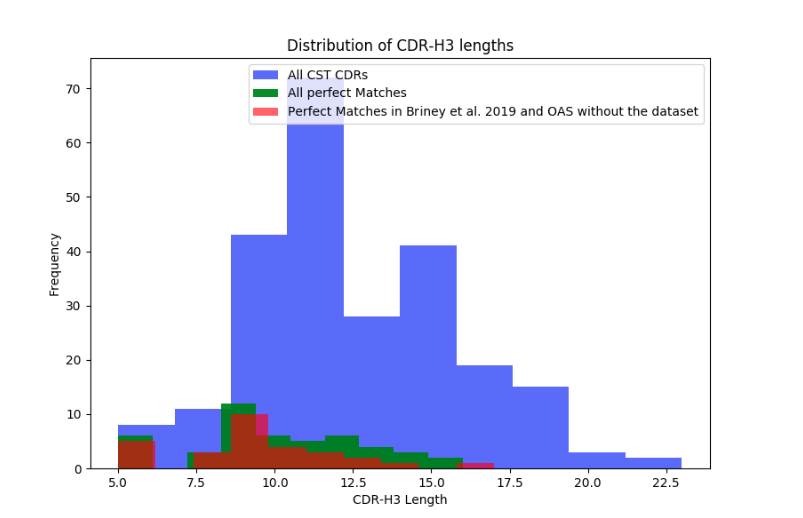


**Supplementary Figure 1.** Distribution of CST CDR-H3 lengths (blue) overlaid on the lengths of the perfect matches (green). The red histogram shows the length distribution of CST CDR-H3 that can be found in the deep sequencing dataset of Briney et al. 2019 and in other datasets in OAS when Briney et al. 2019 data are removed.

**Section 2. Theoretical estimates of probability of finding perfect matches to CDR-H3 region.**

We have estimated the number of theoretically allowed CDR-H3s for each length, assuming that each amino acid is allowed at each position. We calculated the number of possible CDR-H3s for a given length as 20^L^, where L is the length of the loop and 20 represents the number of allowed amino acids. We have also estimated how likely it is to find a single sequence for a given length, assuming 960m independent samples (number of our heavy chain sequences, disregarding H3 redundancy and length stratification in our dataset to be deliberately more permissive) as 9.4x10^8^/20^L^ for a given length L. The estimates for each loop length are given in Supplementary Table 1. For length 12, which is the mean length of the CSTs, the number of theoretically allowed CDR-H3s is 4.096x10^15^ and the probability of finding a single match in 960m independent samples is in the order of 10^-7^, whereas we find seven perfect CST matches for this particular length. The longest CDR-H3 we can find a perfect match for is length 17 and the probability of finding a single sequence here is in the order of 10^-14^.

It is known that NGS technology can introduce sequencing errors because of its high-throughput nature. The worst case scenario assumes that the OAS sequences would be erroneous to the point of uniformly random distribution of the observed amino acids. In such case, observing as many matches would be extremely unlikely as reported in Supplementary Table 1.

| CDR-H3 length | Number of possible sequences | Length frequency in CSTs | Perfect matches | Probability of finding a single sequence in 960m samples |
| --- | --- | --- | --- | --- |
| 5 | 3,200,000 | 3 | 3 | 1 |
| 6 | 64,000,000 | 5 | 3 | 1 |
| 7 | 1,280,000,000 | 2 | 0 | 0.75 |
| 8 | 25,600,000,000 | 9 | 3 | 0.0375 |
| 9 | 512,000,000,000 | 22 | 14 | 0.00187 |
| 10 | 10,240,000,000,000 | 21 | 7 | 9.375e-05 |
| 11 | 204,800,000,000,000 | 29 | 6 | 4.6875e-06 |
| 12 | 4,096,000,000,000,000 | 43 | 7 | 2.34375e-07 |
| 13 | 81,920,000,000,000,000 | 28 | 5 | 1.171875e-08 |
| 14 | 1,638,400,000,000,000,000 | 23 | 3 | 5.859375e-10 |
| 15 | 32,768,000,000,000,000,000 | 18 | 1 | 2.9296875e-11 |
| 16 | 655,360,000,000,000,000,000 | 14 | 1 | 1.46484375e-12 |
| 17 | 13,107,200,000,000,000,000,000 | 5 | 1 | 7.32421875e-14 |
| 18 | 262,144,000,000,000,000,000,000 | 7 | 0 | 3.662109375e-15 |
| 19 | 5,242,880,000,000,000,000,000,000 | 8 | 0 | 1.8310546875e-16 |
| 20 | 104,857,600,000,000,000,000,000,000 | 3 | 0 | 9.1552734375e-18 |
| 23 | 838,860,800,000,000,000,000,000,000,000 | 2 | 0 | 1.14440917969e-21 |

**Supplementary Table 1.** Estimated theoretical number of CDR-H3 sequences for each IMGT length and probabilities of finding a single sequence given 940m independent samples.

**Section 3. Quantifying pairwise sequence identities of therapeutic sequences.**

To provide context practically allowed sequence identities in patent claims, we calculated the identity of each pair of therapeutic sequences in our set of 242 CSTs (Supplementary Figure 2 for heavy chains and Supplementary Figure 3 for light chains). In only four cases is it possible to find heavy chains across two different therapeutics that are more than 94% sequence identical and these are given in Supplementary Table 2. These pairs of therapeutics however are by and large produced by the same company as Ravulizumab and Eculizumab are from Alexion Pharmaceuticals, Ranibizumab and Bevacizumab are from Genentech whereas Palivizumab and Motavizumab are from Medimmune. Tomuzotuximab, by Glycotope, is based on Cetuximab by Bristol Myers Squibb, the patent on which expired several years ago (1).


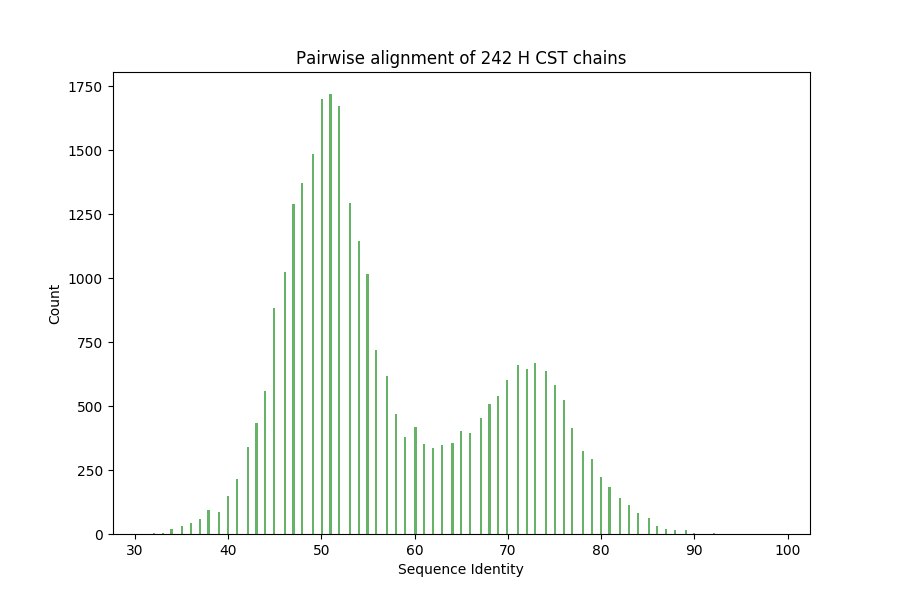


**Supplementary Figure 2.** Histogram of pairwise sequence identities of the 242 CST heavy chains from our dataset.


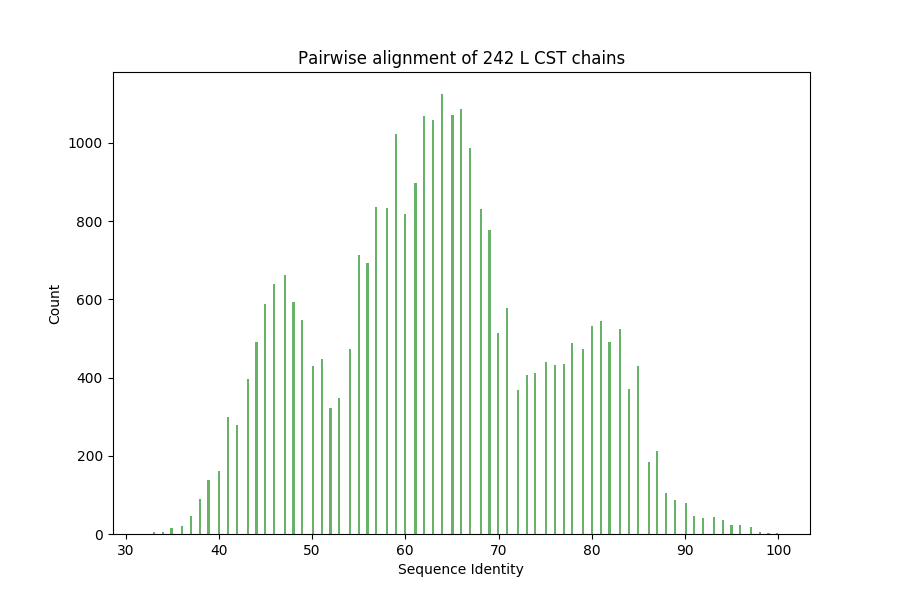


**Supplementary Figure 3**. **.** Histogram of pairwise sequence identities of the 242 CST light chains from our dataset.

| **Therapeutic Antibodies** | **Heavy Chain Sequence Identity** |
| --- | --- |
| Tomuzotuximab & Cetuximab | 99% |
| Ranibizumab & Bevacizumab | 96% |
| Palivizumab & Motavizumab | 94% |
| Ravulizumab & Eculizumab | 98% |

**Supplementary Table 2.** The pairwise sequence identities of CST heavy that are higher than or equal to 94%.

**Section 4. Alignments between CST antibodies and sequences in OAS.**

Below you can see the precise IMGT-aligned sequences of a CST heavy chain or light chain, heavy and light CDR regions and CDR-H3 alone with the best templates we could find for them in OAS. The alignments are sorted alphabetically by the name of the CST. In each of the alignments, the CST sequence comes first, followed by aligned (|) and unaligned (.) entries of the following sequence in the OAS. IMGT-CDRs are marked by the (^) symbol. The alignments for the CDR triplets and CDR-H3 are provided together with the alignment of the entire variable region NGS sequence where the match was found to offer more context.

Therapeutic : Abagovomab

Best Alignment of Abagovomab heavy chain to a sequence from OAS

QVKLQESGAELARPGASVKLSCKASGYTFTNYWMQWVKQRPGQGLDWIGAIYPGDGNTRYTHKFKGKATLTADKSSSTAYMQLSSLASEDSGVYYCARGEGN-YAWFAYWGQGTTVTVSS

.|||||||||||||||||||||||||||||.||||||||||||||.||||||||||.||||.|||||||||||||||||||||||||||||.||||||||....|||||||||||.||||

-VKLQESGAELARPGASVKLSCKASGYTFTSYWMQWVKQRPGQGLEWIGAIYPGDGDTRYTQKFKGKATLTADKSSSTAYMQLSSLASEDSAVYYCARGEYGNSAWFAYWGQGTTLTVSS

^^^^^^^^ ^^^^^^^^ ^^^^^^^^^^^^^

Best Alignment of Abagovomab light chain to a sequence from OAS

DIELTQSPASLSASVGETVTITCQASENIYSYLAWHQQKQGKSPQLLVYNAKTLAGGVSSRFSGSGSGTHFSLKIKSLQPEDFGIYYCQHHYGILPTFGGGTKLEIK

||.||||||||||||||||||||.|||||||||||.|||||||||||||||||||.||.||||||||||.|||||.||||||||.||||||||..||||||||||||

DIVLTQSPASLSASVGETVTITCRASENIYSYLAWYQQKQGKSPQLLVYNAKTLAEGVPSRFSGSGSGTQFSLKINSLQPEDFGSYYCQHHYGTPPTFGGGTKLEIK

^^^^^^ ^^^ ^^^^^^^^^

Best Alignment of Abagovomab heavy chain CDRs to a sequence from OAS

QVKLQESGAELARPGASVKLSCKASGYTFTNYWMQWVKQRPGQGLDWIGAIYPGDGNTRYTHKFKGKATLTADKSSSTAYMQLSSLASEDSGVYYCARGEGNYAWFAYWGQGTTVTVSS

.|.|.||||||.|||.|||.|||||||||||||..|.|||||.||.|||.||||.|.|.|..||||||||||||||||||||.|||.||||..||||||.|||||||||||||.||||.

-VRLVESGAELVRPGTSVKMSCKASGYTFTNYWIGWAKQRPGHGLEWIGDIYPGGGYTNYNEKFKGKATLTADKSSSTAYMQFSSLTSEDSAIYYCARGYGNYAWFAYWGQGTLVTVSA

^^^^^^^^ ^^^^^^^^ ^^^^^^^^^^^^

Best Alignment of Abagovomab light chain CDRs to a sequence from OAS

DIELTQSPASLSASVGETVTITCQASENIYSYLAWHQQKQGKSPQLLVYNAKTLAGGVSSRFSGSGSGTHFSLKIKSLQPEDFGIYYCQHHYGILPTFGGGTKLEIK

...................||||..||||||||||.|||||||||||||||||||.||.||||||||||.|||||.||||||||.|||||||||.||||.|||||.|

-------------------TITCRPSENIYSYLAWYQQKQGKSPQLLVYNAKTLAEGVPSRFSGSGSGTQFSLKINSLQPEDFGSYYCQHHYGIPPTFGAGTKLELK

^^^^^^ ^^^ ^^^^^^^^^

Best Alignment of Abagovomab CDR-H3 to a sequence from OAS

QVKLQESGAELARPGASVKLSCKASGYTFTNYWMQWVKQRPGQGLDWIGAIYPGDGNTRYTHKFKGKATLTADKSSSTAYMQLSSLASEDSGVYYCARGEGNYAWFAYWGQGTTVTVSS

.|.|.|||..|..||.|.|.||.|||..|..|||.||.|.||.||.|||.|.|......||...|.|.....|....|.|.|.|...|||...||||||||||||||||||||.||||.

EVMLVESGGGLVQPGGSLKPSCAASGFDFSRYWMSWVRQAPGKGLEWIGEINPDSSTINYTPSLKDKFIISRDNAKNTLYLQMSKVRSEDTALYYCARGEGNYAWFAYWGQGTLVTVSA

^^^^^^^^ ^^^^^^^^ ^^^^^^^^^^^^

Therapeutic : Abituzumab

Best Alignment of Abituzumab heavy chain to a sequence from OAS

QVQLQQSGGELAKPGASVKVSCKASGYTFSSFWMHWVRQAPGQGLEWIGYINPRSGYTEYNEIFRDKATMTTDTSTSTAYMELSSLRSEDTAVYYCASFLGRGAMDYWGQGTTVTVSS

||||||||.||||||||||.|||||||||.|.|||||.|.|||||||||||||.||||.||..|.||||.|.|.|.|||||.||||.|||.||||||.||||||||||||||.|||||

QVQLQQSGAELAKPGASVKLSCKASGYTFTSYWMHWVKQRPGQGLEWIGYINPSSGYTKYNQKFKDKATLTADKSSSTAYMQLSSLTSEDSAVYYCARFLGRGAMDYWGQGTSVTVSS

^^^^^^^^ ^^^^^^^^ ^^^^^^^^^^^

Best Alignment of Abituzumab light chain to a sequence from OAS

DIQMTQSPSSLSASVGDRVTITCRASQDISNYLAWYQQKPGKAPKLLIYYTSKIHSGVPSRFSGSGSGTDYTFTISSLQPEDIATYYCQQGNTFPYTFGQGTKVEIK

|||||||||||||||||||||||.|||||||||.|||||||||||||||..|....||||||||||||||.|||||||||||||||||||.||||||||||||.|||

DIQMTQSPSSLSASVGDRVTITCQASQDISNYLNWYQQKPGKAPKLLIYDASNLETGVPSRFSGSGSGTDFTFTISSLQPEDIATYYCQQSNTFPYTFGQGTKLEIK

^^^^^^ ^^^ ^^^^^^^^^

Best Alignment of Abituzumab heavy chain CDRs to a sequence from OAS

QVQLQQSGGELAKPGASVKVSCKASGYTFSSFWMHWVRQAPGQGLEWIGYINPRSGYTEYNEIFRDKATMTTDTSTSTAYMELSSLRSEDTAVYYCASFLGRGAMDYWGQGTTVTVSS

||||||||.||||||||||.|||||||||.|.|||||.|.|||||||||||||.||||.||..|.||||.|.|.|.|||||.||||.|||.||||||.||||||||||||||.|||||

QVQLQQSGAELAKPGASVKLSCKASGYTFTSYWMHWVKQRPGQGLEWIGYINPSSGYTKYNQKFKDKATLTADKSSSTAYMQLSSLTSEDSAVYYCARFLGRGAMDYWGQGTSVTVSS

^^^^^^^^ ^^^^^^^^ ^^^^^^^^^^^

Best Alignment of Abituzumab light chain CDRs to a sequence from OAS

DIQMTQSPSSLSASVGDRVTITCRASQDISNYLAWYQQKPGKAPKLLIYYTSKIHSGVPSRFSGSGSGTDYTFTISSLQPEDIATYYCQQGNTFPYTFGQGTKVEIK

||.|||..||||||.||||||.|||||||||||.||||||....||||||||..|||||||||||||||||..|||.|..||||||.||||||||||||.|||.|||

DIKMTQTTSSLSASLGDRVTISCRASQDISNYLNWYQQKPDGTVKLLIYYTSRLHSGVPSRFSGSGSGTDYSLTISNLEQEDIATYFCQQGNTFPYTFGGGTKLEIK

^^^^^^ ^^^ ^^^^^^^^^

Best Alignment of Abituzumab CDR-H3 to a sequence from OAS

QVQLQQSGGELAKPGASVKVSCKASGYTFSSFWMHWVRQAPGQGLEWIGYINPRSGYTEYNEIFRDKATMTTDTSTSTAYMELSSLRSEDTAVYYCASFLGRGAMDYWGQGTTVTVSS

.|.|..|||.|.|||.|.|.||.|||.|||...|.||||.|...|||...|.....||.|........|...|......|...|||.|||||.|||||.|||||||||||||.|||||

EVKLMESGGGLVKPGGSLKLSCAASGFTFSDYYMYWVRQTPEKRLEWVATISDGGSYTYYPDSVKGRFTISRDNAKNNLYLQMSSLKSEDTAMYYCASELGRGAMDYWGQGTSVTVSS

^^^^^^^^ ^^^^^^^^ ^^^^^^^^^^^

Therapeutic : Abrilumab

Best Alignment of Abrilumab heavy chain to a sequence from OAS

QVQLVQSGAEVKKPGASVKVSCKVSGYTLSDLSIHWVRQAPGKGLEWMGGFDPQDGETIYAQKFQGRVTMTEDTSTDTAYMELSSLKSEDTAVYYCATGSSS----SWFDPWGQGTLVTVSS

|||||||||||||||||||||||||||||..||.|||||||||||||||||||.||||||||||||||||||||||||||||||||.|||||||||||.|||.....|||||||||||||||

QVQLVQSGAEVKKPGASVKVSCKVSGYTLTELSMHWVRQAPGKGLEWMGGFDPEDGETIYAQKFQGRVTMTEDTSTDTAYMELSSLRSEDTAVYYCATYSSSWYKKGWFDPWGQGTLVTVSS

^^^^^^^^ ^^^^^^^^ ^^^^^^^^^^^^^^^

Best Alignment of Abrilumab light chain to a sequence from OAS

DIQMTQSPSSVSASVGDRVTITCRASQGISSWLAWYQQKPGKAPKLLIYGASNLESGVPSRFSGSGSGTDFTLTISSLQPEDFANYYCQQANSFPWTFGQGTKVEIK

||||||||||||||||||||||||||||||||||||||||||||||||||||.|.|||||||||||||||||||||||||||||.||||||||||||||||||||||

DIQMTQSPSSVSASVGDRVTITCRASQGISSWLAWYQQKPGKAPKLLIYGASSLQSGVPSRFSGSGSGTDFTLTISSLQPEDFATYYCQQANSFPWTFGQGTKVEIK

^^^^^^ ^^^ ^^^^^^^^^

Best Alignment of Abrilumab heavy chain CDRs to a sequence from OAS

QVQLVQSGAEVKKPGASVKVSCKVSGYTLSDLSIHWVRQAPGKGLEWMGGFDPQDGETIYAQKFQGRVTMTEDTSTDTAYMELSSLKSEDTAVYYCATGSSSSWFDPWGQGTLVTVSS

................|||||||||||||..||.|||||||||||||||||||.||||||||||||||||||||||||||||||||.||||||||||||.||||||||||||||||||

----------------SVKVSCKVSGYTLTELSMHWVRQAPGKGLEWMGGFDPEDGETIYAQKFQGRVTMTEDTSTDTAYMELSSLRSEDTAVYYCATGYSSSWFDPWGQGTLVTVSS

^^^^^^^^ ^^^^^^^^ ^^^^^^^^^^^

Best Alignment of Abrilumab light chain CDRs to a sequence from OAS

DIQMTQSPSSVSASVGDRVTITCRASQGISSWLAWYQQKPGKAPKLLIYGASNLESGVPSRFSGSGSGTDFTLTISSLQPEDFANYYCQQANSFPWTFGQGTKVEIK

.||.||||||||||||||||||||||||||||||||||||||||||||||||.|.|||||||||||||||||||||||||||||.||||||||||||||||||||||

-IQTTQSPSSVSASVGDRVTITCRASQGISSWLAWYQQKPGKAPKLLIYGASSLQSGVPSRFSGSGSGTDFTLTISSLQPEDFATYYCQQANSFPWTFGQGTKVEIK

^^^^^^ ^^^ ^^^^^^^^^

Best Alignment of Abrilumab CDR-H3 to a sequence from OAS

QVQLVQSGAEVKKPGASVKVSCKVSGYTL--SDLSIHWVRQAPGKGLEWMGGFDPQDGETIYAQKFQGRVTMTEDTSTDTAYMELSSLKSEDTAVYYCATGSSSSWFDPWGQGTLVTVSS

.....................|.|||.....|.....|.||.||.||||.|......|.|.|......|||...|||.......|||....||||||||.||||||||||||||||||||

--------------SETLSLTCTVSGGSISSSSYYWGWIRQPPGTGLEWIGSIYYS-GSTYYNPSLKSRVTISVDTSKNQFSLKLSSVTAADTAVYYCARGSSSSWFDPWGQGTLVTVSS

^^^^^^^^^^ ^^^^^^^^ ^^^^^^^^^^^

Therapeutic : Actoxumab

Best Alignment of Actoxumab heavy chain to a sequence from OAS

QVQLVESGGGVVQPGRSLRLSCAASGFSFSNYGMHWVRQAPGKGLEWVALIWYDGSNEDYTDSVKGRFTISRDNSKNTLYLQMNSLRAEDTAVYYCARWGMVRG------VIDVFDIWGQGTVVTVSS

|||||||||||||||||||||||||||.||.||||||||||||||||||.|||||||..|.|||||||||||||||||||||||||||||||||||||.|||||.......|..||.|||||.|||||

QVQLVESGGGVVQPGRSLRLSCAASGFTFSSYGMHWVRQAPGKGLEWVAVIWYDGSNKYYADSVKGRFTISRDNSKNTLYLQMNSLRAEDTAVYYCARGGMVRGVWSRRNAIRSFDYWGQGTLVTVSS

^^^^^^^^ ^^^^^^^^ ^^^^^^^^^^^^^^^^^^^^^

Best Alignment of Actoxumab light chain to a sequence from OAS

DIQMTQSPSSVSASVGDRVTITCRASQGISSWLAWYQHKPGKAPKLLIYAASSLQSGVPSRFSGSGSGTDFTLTISSLQPEDFATYYCQQANSFPWTFGQGTKVEIK

|||||||||||||||||||||||||||||||||||||||||||||||||||||||||||||||||||||||||||||||||||||||||||||||||||||||||||

DIQMTQSPSSVSASVGDRVTITCRASQGISSWLAWYQHKPGKAPKLLIYAASSLQSGVPSRFSGSGSGTDFTLTISSLQPEDFATYYCQQANSFPWTFGQGTKVEIK

^^^^^^ ^^^ ^^^^^^^^^

Best Alignment of Actoxumab heavy chain CDRs to a sequence from OAS

QVQLVESGGGVVQPGRSLRLSCAASGFSFSNYGMHWVRQAPGKGLEWVALIWYDGSNEDYTDSVKGRFTISRDNSKNTLYLQMNSLRAEDTAVYYCARWGMVRGVIDVFDIWGQGTVVTVSS

................|||||||||||.||.||||||||||||||||||.|||||||..|.|||||||||||||||||||||||||||||||||||||.||||||||.||||||||.|||||

----------------SLRLSCAASGFTFSSYGMHWVRQAPGKGLEWVAVIWYDGSNKYYADSVKGRFTISRDNSKNTLYLQMNSLRAEDTAVYYCARSGMVRGVIDAFDIWGQGTMVTVSS

^^^^^^^^ ^^^^^^^^ ^^^^^^^^^^^^^^^

Best Alignment of Actoxumab light chain CDRs to a sequence from OAS

DIQMTQSPSSVSASVGDRVTITCRASQGISSWLAWYQHKPGKAPKLLIYAASSLQSGVPSRFSGSGSGTDFTLTISSLQPEDFATYYCQQANSFPWTFGQGTKVEIK

|||||||||||||||||||||||||||||||||||||.||||||||||||||||||||||||||||||||||||||||||||.||||||||||||||||||||||||

DIQMTQSPSSVSASVGDRVTITCRASQGISSWLAWYQQKPGKAPKLLIYAASSLQSGVPSRFSGSGSGTDFTLTISSLQPEDLATYYCQQANSFPWTFGQGTKVEIK

^^^^^^ ^^^ ^^^^^^^^^

Best Alignment of Actoxumab CDR-H3 to a sequence from OAS

QVQLVESGGGVVQPGRSLRLSCAASGFSFSNYGMHWVRQAPGKGLEWVALIWYDGSNEDYTDSVKGRFTISRDNSKNTLYLQMNSLRAEDTAVYYCARWGMVRGVIDVFDIWGQGTVVTVSS

................|||||||||||.||.||||||||||||||||||.|||||||..|.|||||||||||||||||||||||||||||||||||||.||||||||.||||||||.|||||

----------------SLRLSCAASGFTFSSYGMHWVRQAPGKGLEWVAVIWYDGSNKYYADSVKGRFTISRDNSKNTLYLQMNSLRAEDTAVYYCARSGMVRGVIDAFDIWGQGTMVTVSS

^^^^^^^^ ^^^^^^^^ ^^^^^^^^^^^^^^^

Therapeutic : Adalimumab

Best Alignment of Adalimumab heavy chain to a sequence from OAS

EVQLVESGGGLVQPGRSLRLSCAASGFTFDDYAMHWVRQAPGKGLEWVSAITWNSGHIDYADSVEGRFTISRDNAKNSLYLQMNSLRAEDTAVYYCAKVSYLS-TASSLDYWGQGTLVTVSS

|||||||||||||||||||||||||||||||||||||||||||||||||.|.||||.|.|||||.|||||||||||||||||||||||||||.|||||.||...||.|||||||||||||||

EVQLVESGGGLVQPGRSLRLSCAASGFTFDDYAMHWVRQAPGKGLEWVSGISWNSGSIGYADSVKGRFTISRDNAKNSLYLQMNSLRAEDTALYYCAKASYTVTTAFSLDYWGQGTLVTVSS

^^^^^^^^ ^^^^^^^^ ^^^^^^^^^^^^^^^

Best Alignment of Adalimumab light chain to a sequence from OAS

DIQMTQSPSSLSASVGDRVTITCRASQGIRNYLAWYQQKPGKAPKLLIYAASTLQSGVPSRFSGSGSGTDFTLTISSLQPEDVATYYCQRYNRAPYTFGQGTKVEIK

||||||||||||||||||||||||||||||||||||||||||.|||||||||||||||||||||||||||||||||||||||||||||||||.||.|||||||||||

DIQMTQSPSSLSASVGDRVTITCRASQGIRNYLAWYQQKPGKVPKLLIYAASTLQSGVPSRFSGSGSGTDFTLTISSLQPEDVATYYCQRYNSAPRTFGQGTKVEIK

^^^^^^ ^^^ ^^^^^^^^^

Best Alignment of Adalimumab heavy chain CDRs to a sequence from OAS

EVQLVESGGGLVQPGRSLRLSCAASGFTFDDYAMHWVRQAPGKGLEWVSAITWNSGHIDYADSVEGRFTISRDNAKNSLYLQMNSLRAEDTAVYYCAKVSYLSTASSLDYWGQGTLVTVSS

..............|.|||||||||||||||||||||||||||||||||.|.||||.|.|||.|.||||||||||||||||||||||.||||.|||||.||.|..||||||||||||||||

--------------GGSLRLSCAASGFTFDDYAMHWVRQAPGKGLEWVSGISWNSGSIGYADFVKGRFTISRDNAKNSLYLQMNSLRVEDTALYYCAKDSYSSSWSSLDYWGQGTLVTVSS

^^^^^^^^ ^^^^^^^^ ^^^^^^^^^^^^^^

Best Alignment of Adalimumab light chain CDRs to a sequence from OAS

DIQMTQSPSSLSASVGDRVTITCRASQGIRNYLAWYQQKPGKAPKLLIYAASTLQSGVPSRFSGSGSGTDFTLTISSLQPEDVATYYCQRYNRAPYTFGQGTKVEIK

|||||||||||||||||||||||||||||.||||||||||||.||||||||||||||||||||||||||||||||||||||||||||||||||||||||||||.|||

DIQMTQSPSSLSASVGDRVTITCRASQGINNYLAWYQQKPGKVPKLLIYAASTLQSGVPSRFSGSGSGTDFTLTISSLQPEDVATYYCQRYNRAPYTFGQGTKLEIK

^^^^^^ ^^^ ^^^^^^^^^

Best Alignment of Adalimumab CDR-H3 to a sequence from OAS

EVQLVESGGGLVQPGRSLRLSCAASGFTFDDYAMHWVRQAPGKGLEWVSAITWNSGHIDYADSVEGRFTISRDNAKNSLYLQMNSLRAEDTAVYYCAKVSYLSTASSLDYWGQGTLVTVSS

..............|.|||||||||||||..|.|||||||||||||||..|........|||||.|||||||||.||.|||||||||||||||||||||..||..||||||||||||||||

--------------GGSLRLSCAASGFTFSSYGMHWVRQAPGKGLEWVAVISYDGSNKYYADSVKGRFTISRDNSKNTLYLQMNSLRAEDTAVYYCAKVQGLSRRSSLDYWGQGTLVTVSS

^^^^^^^^ ^^^^^^^^ ^^^^^^^^^^^^^^

Therapeutic : Aducanumab

Best Alignment of Aducanumab heavy chain to a sequence from OAS

QVQLVESGGGVVQPGRSLRLSCAASGFAFSSYGMHWVRQAPGKGLEWVAVIWFDGTKKYYTDSVKGRFTISRDNSKNTLYLQMNTLRAEDTAVYYCARDRGIGAR-RGPYYMDVWGKGTTVTVSS

|||||||||||||||||||||||||||.||||||||||||||||||||||||.||..|||.|||||||||||||||||||||||.||||||||||||||.|...|.||.||||||||||||||||

QVQLVESGGGVVQPGRSLRLSCAASGFTFSSYGMHWVRQAPGKGLEWVAVIWYDGSNKYYADSVKGRFTISRDNSKNTLYLQMNSLRAEDTAVYYCARDWGSITRNRGYYYMDVWGKGTTVTVSS

^^^^^^^^ ^^^^^^^^ ^^^^^^^^^^^^^^^^^^

Best Alignment of Aducanumab light chain to a sequence from OAS

DIQMTQSPSSLSASVGDRVTITCRASQSISSYLNWYQQKPGKAPKLLIYAASSLQSGVPSRFSGSGSGTDFTLTISSLQPEDFATYYCQQSYSTPLTFGGGTKVEIK

|||||||||||||||||||||||||||||||||||||||||||||||||||||||||||||||||||||||||||||||||||||||||||||||||||||||||||

DIQMTQSPSSLSASVGDRVTITCRASQSISSYLNWYQQKPGKAPKLLIYAASSLQSGVPSRFSGSGSGTDFTLTISSLQPEDFATYYCQQSYSTPLTFGGGTKVEIK

^^^^^^ ^^^ ^^^^^^^^^

Best Alignment of Aducanumab heavy chain CDRs to a sequence from OAS

QVQLVESGGGVVQPGRSLRLSCAASGFAFSSYGMHWVRQAPGKGLEWVAVIWFDGTKKYYTDSVKGRFTISRDNSKNTLYLQMNTLRAEDTAVYYCARDRGIGARRGPYYMDVWGKGTTVTVSS

..............|.|||||||||||.|||||||||||||||||||||||||||.|||..|||||||||||||||||||||||.||||||||||||||||.|.|...||||||||||||||||

--------------GGSLRLSCAASGFIFSSYGMHWVRQAPGKGLEWVAVIWFDGSKKYFADSVKGRFTISRDNSKNTLYLQMNSLRAEDTAVYYCARDRGYGTRYSYYYMDVWGKGTTVTVSS

^^^^^^^^ ^^^^^^^^ ^^^^^^^^^^^^^^^^^

Best Alignment of Aducanumab light chain CDRs to a sequence from OAS

DIQMTQSPSSLSASVGDRVTITCRASQSISSYLNWYQQKPGKAPKLLIYAASSLQSGVPSRFSGSGSGTDFTLTISSLQPEDFATYYCQQSYSTPLTFGGGTKVEIK

|||||||||||||||||||||||||||||||||||||||||||||.|||||||||||||||||||||||||||||||||||||.|||||||||||||||||||||||

DIQMTQSPSSLSASVGDRVTITCRASQSISSYLNWYQQKPGKAPKPLIYAASSLQSGVPSRFSGSGSGTDFTLTISSLQPEDFGTYYCQQSYSTPLTFGGGTKVEIK

^^^^^^ ^^^ ^^^^^^^^^

Best Alignment of Aducanumab CDR-H3 to a sequence from OAS

QVQLVESGGGVVQPGRSLRLSCAASGFAFSSYGMHWVRQAPGKGLEWVAVIWFDGTKKYYTDSVKGRFTISRDNSKNTLYLQMNTLRAEDTAVYYCARDRGIGARRGPYYMDVWGKGTTVTVSS

.............||.|||||||||||.||.|.|.|.|||||||||||..|.......||.|||||||||||||..|.||||||.||||||||||||||||||||.|.|||||||||||||||.

-------------PGGSLRLSCAASGFTFSDYYMSWIRQAPGKGLEWVSYISSSDSTIYYADSVKGRFTISRDNAENSLYLQMNSLRAEDTAVYYCARDRGIGARPGDYYMDVWGKGTTVTVS-

^^^^^^^^ ^^^^^^^^ ^^^^^^^^^^^^^^^^^

Therapeutic : Alemtuzumab

Best Alignment of Alemtuzumab heavy chain to a sequence from OAS

QVQLQESGPGLVRPSQTLSLTCTVSGFTF--TDFYMNWVRQPPGRGLEWIGFIRDKAKGYTTEYNPSVKGRVTMLVDTSKNQFSLRLSSVTAADTAVYYCAREGHT----AAPFDYWGQGSLVTVSS

||||||||||||.|||||||||||||......|||..|.|||||.||||||||.|.....||.||||.|.|||..||||||||||.||||||||||||||||...|......||||||||.||||||

QVQLQESGPGLVKPSQTLSLTCTVSGGSISTADFYWSWIRQPPGKGLEWIGFIHDS---GTTYYNPSLKSRVTISVDTSKNQFSLKLSSVTAADTAVYYCARGLPTRLRTQRPFDYWGQGTLVTVSS

^^^^^^^^^^ ^^^^^^^^^^ ^^^^^^^^^^^^^^^^

Best Alignment of Alemtuzumab light chain to a sequence from OAS

DIQMTQSPSSLSASVGDRVTITCKASQNIDKYLNWYQQKPGKAPKLLIYNTNNLQTGVPSRFSGSGSGTDFTFTISSLQPEDIATYYCLQHISRPRTFGQGTKVEIK

|||||||||||||||||||||||.|||.|..||||||||||||||||||...||.||||||||||||||||||||||||||||||||||||.|.|||||||||||||

DIQMTQSPSSLSASVGDRVTITCQASQDISNYLNWYQQKPGKAPKLLIYDASNLETGVPSRFSGSGSGTDFTFTISSLQPEDIATYYCLQHNSYPRTFGQGTKVEIK

^^^^^^ ^^^ ^^^^^^^^^

Best Alignment of Alemtuzumab heavy chain CDRs to a sequence from OAS

QVQLQESGPGLVRPSQTLSLTCTVSGFTFTDFYMNWVRQPPGRGLEWIGFIRDKAKGYTTEYNPSVKGRVTMLVDTSKNQFSLRLSSVTAADTAVYYCAREGHTAAPFDYWGQGSLVTVSS

.|.|.|||.|||.|...|.|.|..|||||||.||.|||||||..|||.||||.||.||||||..|||||.|...|.|.....|......|.|.|.|||||||.|.||.||||||..|||||

EVHLVESGGGLVQPGGSLRLSCATSGFTFTDYYMSWVRQPPGKALEWLGFIRNKANGYTTEYSASVKGRFTISRDNSQSILYLQMNTLRAEDSATYYCAREGITTAPMDYWGQGTSVTVSS

^^^^^^^^ ^^^^^^^^^^ ^^^^^^^^^^^^

Best Alignment of Alemtuzumab light chain CDRs to a sequence from OAS

DIQMTQSPSSLSASVGDRVTITCKASQNIDKYLNWYQQKPGKAPKLLIYNTNNLQTGVPSRFSGSGSGTDFTFTISSLQPEDIATYYCLQHISRPRTFGQGTKVEIK

|||||||||||||||||..||||.|||.|.|||.|||||||||||.||..|..||.||||||||.||.||.|..||.|||.|.||||.|||.|.|||||||||||||

DIQMTQSPSSLSASVGDTGTITCQASQGISKYLAWYQQKPGKAPKPLINDTSTLQSGVPSRFSGIGSWTDCTLPISTLQPADFATYYDLQHNSYPRTFGQGTKVEIK

^^^^^^ ^^^ ^^^^^^^^^

Best Alignment of Alemtuzumab CDR-H3 to a sequence from OAS

QVQLQESGPGLVRPSQTLSLTCTVSGFTF-TDFYMNWVRQPPGRGLEWIGFIRDKAKGYTTEYNPSVKGRVTMLVDTSKNQFSLRLSSVTAADTAVYYCAREGHTAAPFDYWGQGSLVTVSS

.....||||.||.|||.|||||||.|..........|.||.||..|||.|.|........|.||||.|.|.....|||||||.|.|.|||..|||.|||||||.|||||||||||...||||

-----ESGPDLVKPSQSLSLTCTVTGYSITSGYSWHWIRQFPGNKLEWMGYIHYS---GSTNYNPSLKSRISITRDTSKNQFFLQLNSVTTEDTATYYCAREGITAAPFDYWGQGTTLTVSS

^^^^^^^^^ ^^^^^^^^^^ ^^^^^^^^^^^^

Therapeutic : Alirocumab

Best Alignment of Alirocumab heavy chain to a sequence from OAS

EVQLVESGGGLVQPGGSLRLSCAASGFTFNNYAMNWVRQAPGKGLDWVSTISGSGGTTNYADSVKGRFIISRDSSKHTLYLQMNSLRAEDTAVYYCAKDSNW--GNFDLWGRGTLVTVSS

||||.||||||||||||||||||||||||..||||||||||||||.||||||||||.|.|||||||||.||||.||.|||||||||||||||||||||||....|.||||||||||||||

EVQLLESGGGLVQPGGSLRLSCAASGFTFSSYAMNWVRQAPGKGLEWVSTISGSGGSTYYADSVKGRFTISRDNSKNTLYLQMNSLRAEDTAVYYCAKDSSGWGGGFDLWGRGTLVTVSS

^^^^^^^^ ^^^^^^^^ ^^^^^^^^^^^^^

Best Alignment of Alirocumab light chain to a sequence from OAS

DIVMTQSPDSLAVSLGERATINCKSSQSVLYRSNNRNFLGWYQQKPGQPPNLLIYWASTRESGVPDRFSGSGSGTDFTLTISSLQAEDVAVYYCQQYYTTPYTFGQGTKLEIK

|||||||||||||||||||||||||||||||.|||.|.||||||||||||.||||||||||||||||||||||||||||||||||||||||||||||||||||||||||||||

DIVMTQSPDSLAVSLGERATINCKSSQSVLYSSNNNNYLGWYQQKPGQPPKLLIYWASTRESGVPDRFSGSGSGTDFTLTISSLQAEDVAVYYCQQYYTTPYTFGQGTKLEIK

^^^^^^^^^^^^ ^^^ ^^^^^^^^^

Best Alignment of Alirocumab heavy chain CDRs to a sequence from OAS

EVQLVESGGGLVQPGGSLRLSCAASGFTFNNYAMNWVRQAPGKGLDWVSTISGSGGTTNYADSVKGRFIISRDSSKHTLYLQMNSLRAEDTAVYYCAKDSNWGNFDLWGRGTLVTVSS

..............||||||||||||||||.|||.||||||||||.||||||||||.|.|||||||||.||||.||.||||||||||||||||||||||||||.||.||.||||||||

--------------GGSLRLSCAASGFTFNSYAMSWVRQAPGKGLEWVSTISGSGGSTYYADSVKGRFTISRDNSKNTLYLQMNSLRAEDTAVYYCAKDSNWGYFDYWGQGTLVTVSS

^^^^^^^^ ^^^^^^^^ ^^^^^^^^^^^

Best Alignment of Alirocumab light chain CDRs to a sequence from OAS

DIVMTQSPDSLAVSLGERATINCKSSQSVLYRSNNRNFLGWYQQKPGQPPNLLIYWASTRESGVPDRFSGSGSGTDFTLTISSLQAEDVAVYYCQQYYTTPYTFGQGTKLEIK

||||||||||||||||||||.||||||||||||||||||.||||||||||.|||||||||||||||||||||||||||||||||||||||.|||||||.|||||||||||.||

DIVMTQSPDSLAVSLGERATLNCKSSQSVLYRSNNRNFLAWYQQKPGQPPKLLIYWASTRESGVPDRFSGSGSGTDFTLTISSLQAEDVAGYYCQQYYSTPYTFGQGTKLQIK

^^^^^^^^^^^^ ^^^ ^^^^^^^^^

Best Alignment of Alirocumab CDR-H3 to a sequence from OAS

EVQLVESGGGLVQPGGSLRLSCAASGFTFNNYAMNWVRQAPGKGLDWVSTISGS--GGTTNYADSVKGRFIISRDSSKHTLYLQMNSLRAEDTAVYYCAKDSNWGNFDLWGRGTLVTVSS

..............||||||||||||||..||.|.||||||.|||.|.|.|.....|..|..||||||||.||||.|..||||||.||..||||||||||||.||||||||||.||||||

--------------GGSLRLSCAASGFTLSNYPMGWVRQAPVKGLEWLSAIGEEKSGSWTKSADSVKGRFTISRDNSENTLYLQMDSLTVEDTAVYYCAKDSSWGNFDLWGRGALVTVSS

^^^^^^^^ ^^^^^^^^^^ ^^^^^^^^^^^

Therapeutic : Amatuximab

Best Alignment of Amatuximab heavy chain to a sequence from OAS

QVQLQQSGPELEKPGASVKISCKASGYSFTGYTMNWVKQSHGKSLEWIGLITPYNGASSYNQKFRGKATLTVDKSSSTAYMDLLSLTSEDSAVYFCARGGYD-GRGFDYWGSGTPVTVSS

.||||||||||||||||||||||||||||||||||||||||||.|||||||.||||..||||||.||||||||||||||||.||||||||||||.|||||||..|||.|||.||.||||.

-VQLQQSGPELEKPGASVKISCKASGYSFTGYTMNWVKQSHGKNLEWIGLINPYNGGTSYNQKFKGKATLTVDKSSSTAYMELLSLTSEDSAVYYCARGGYDEDRGFAYWGQGTLVTVSA

^^^^^^^^ ^^^^^^^^ ^^^^^^^^^^^^^

Best Alignment of Amatuximab light chain to a sequence from OAS

DIELTQSPAIMSASPGEKVTMTCSASSSVSYMHWYQQKSGTSPKRWIYDTSKLASGVPGRFSGSGSGNSYSLTISSVEAEDDATYYCQQWSKHPLTFGSGTKVEIK

||.||||||||||||||||||||||||||||||||||||.||||.||||||||||||||||||||||||||||||||||||||||||||||..|.|||||||.|||

DIVLTQSPAIMSASPGEKVTMTCSASSSVSYMHWYQQKSSTSPKLWIYDTSKLASGVPGRFSGSGSGNSYSLTISSVEAEDDATYYCQQWSGYPFTFGSGTKLEIK

^^^^^ ^^^ ^^^^^^^^^

Best Alignment of Amatuximab heavy chain CDRs to a sequence from OAS

QVQLQQSGPELEKPGASVKISCKASGYSFTGYTMNWVKQSHGKSLEWIGLITPYNGASSYNQKFRGKATLTVDKSSSTAYMDLLSLTSEDSAVYFCARGGYDGRGFDYWGSGTPVTVSS

.||||.|||||.|.||||||||||||||||||.|.||||||||||||||.|..||||.||||||.||||.|||.|||||||...||||||||||.|||||||||||.|||.||.||||.

DVQLQESGPELVKTGASVKISCKASGYSFTGYYMHWVKQSHGKSLEWIGYISCYNGATSYNQKFKGKATFTVDTSSSTAYMQFNSLTSEDSAVYYCARGGYDGRGFAYWGQGTLVTVSA

^^^^^^^^ ^^^^^^^^ ^^^^^^^^^^^^

Best Alignment of Amatuximab light chain CDRs to a sequence from OAS

DIELTQSPAIMSASPGEKVTMTCSASSSVSYMHWYQQKSGTSPKRWIYDTSKLASGVPGRFSGSGSGNSYSLTISSVEAEDDATYYCQQWSKHPLTFGSGTKVEIK

.|.|||||||||||||||||||||||||||||||||||||||||||||||||||||||.||||||||.||||||||.||||.|||||||||.||||||.|||.|.|

QIVLTQSPAIMSASPGEKVTMTCSASSSVSYMHWYQQKSGTSPKRWIYDTSKLASGVPARFSGSGSGTSYSLTISSMEAEDAATYYCQQWSSHPLTFGAGTKLELK

^^^^^ ^^^ ^^^^^^^^^

Best Alignment of Amatuximab CDR-H3 to a sequence from OAS

QVQLQQSGPELEKPGASVKISCKASGYSF-TGYTMNWVKQSHGKSLEWIGLITPYNGASSYNQKFRGKATLTVDKSSSTAYMDLLSLTSEDSAVYFCARGGYDGRGFDYWGSGTPVTVSS

.||||||||.|.||..|....|...|||...||..||..|..|..|||.|.|....|...||.........|.|.|.......|.|.|.||.|.|.|||||||||||||||.||.||||.

EVQLQQSGPGLVKPSQSLSLTCSVTGYSITSGYYWNWIRQFPGNKLEWMGYISYD-GSNNYNPSLKNRVSITRDTSKNQFFLKLNSVTTEDTATYYCARGGYDGRGFDYWGQGTLVTVSA

^^^^^^^^^ ^^^^^^^^ ^^^^^^^^^^^^

Therapeutic : Andecaliximab

Best Alignment of Andecaliximab heavy chain to a sequence from OAS

QVQLQESGPGLVKPSETLSLTCTVSGFSLLSYGVHWVRQPPGKGLEWLGVIWTGGTTNYNSALMSRFTISKDDSKNTVYLKMNSLKTEDTAIYYCARYYY-----GMDYWGQGTLVTVSS

||||.|||||||.||..||.|||||||||.||||||||||||||||||||||.||.||||||||||..||||.|||.|.||||||.|||||.|||||.||......||||||||.|||||

QVQLKESGPGLVAPSQSLSITCTVSGFSLTSYGVHWVRQPPGKGLEWLGVIWAGGSTNYNSALMSRLSISKDNSKNQVVLKMNSLQTEDTAMYYCARGYYGNPYYAMDYWGQGTSVTVSS

^^^^^^^^ ^^^^^^^ ^^^^^^^^^^^^^^

Best Alignment of Andecaliximab light chain to a sequence from OAS

DIQMTQSPSSLSASVGDRVTITCKASQDVRNTVAWYQQKPGKAPKLLIYSSSYRNTGVPDRFSGSGSGTDFTLTISSLQAEDVAVYYCQQHYITPYTFGGGTKVEIK

|||||||||||||||||||||||.||||..|...|||||||||||||||..|...||||.||||||||||||||||||||||||||||||.|.||||||.|||.|||

DIQMTQSPSSLSASVGDRVTITCQASQDISNYLNWYQQKPGKAPKLLIYHASNLETGVPSRFSGSGSGTDFTLTISSLQAEDVAVYYCQQYYSTPYTFGQGTKLEIK

^^^^^^ ^^^ ^^^^^^^^^

Best Alignment of Andecaliximab heavy chain CDRs to a sequence from OAS

QVQLQESGPGLVKPSETLSLTCTVSGFSLLSYGVHWVRQPPGKGLEWLGVIWTGGTTNYNSALMSRFTISKDDSKNTVYLKMNSLKTEDTAIYYCARYYYGMDYWGQGTLVTVSS

.||..|||||||.||..||.|||||||||.|||||||||.||||||||||||.||.|.||.|..||..||||.||..|..|||||...|||||||||||||||||||||.|||||

-VQRVESGPGLVQPSQSLSITCTVSGFSLTSYGVHWVRQSPGKGLEWLGVIWSGGSTDYNAAFISRLSISKDNSKSQVFFKMNSLQADDTAIYYCARYYYGMDYWGQGTSVTVSS

^^^^^^^^ ^^^^^^^ ^^^^^^^^^

Best Alignment of Andecaliximab light chain CDRs to a sequence from OAS

DIQMTQSPSSLSASVGDRVTITCKASQDVRNTVAWYQQKPGKAPKLLIYSSSYRNTGVPDRFSGSGSGTDFTLTISSLQAEDVAVYYCQQHYITPYTFGGGTKVEIK

.|..|.|....|.||.|||.||||||||||.||||||||||..|||||...|.|.|||||||.|||||||.||||||.||||.|.|||||||.||||||||||.|||

-IVLTLSHKFMSTSVVDRVSITCKASQDVRTTVAWYQQKPGQSPKLLIDWASTRDTGVPDRFTGSGSGTDYTLTISSVQAEDLALYYCQQHYSTPYTFGGGTKLEIK

^^^^^^ ^^^ ^^^^^^^^^

Best Alignment of Andecaliximab CDR-H3 to a sequence from OAS

QVQLQESGPGLVKPSETLSLTCTVSGFSLLSYGVHWVRQPPGKGLEWLGVIWTG-GTTNYNSALMSRFTISKDDSKNTVYLKMNSLKTEDTAIYYCARYYYGMDYWGQGTLVTVSS

.||||.|||.||||.......|..||.|...|...||.|..||.|||.|.|....|.|.||.......|...|.|..|.|....||..||.|.|||||||||||||||||.|||||

EVQLQQSGPELVKPGASMKIYCKASGYSFTGYTMNWVKQSHGKNLEWIGLINPYNGGTSYNQKFKGKATLTVDKSSSTAYMELLSLTSEDSAVYYCARYYYGMDYWGQGTSVTVSS

^^^^^^^^ ^^^^^^^^ ^^^^^^^^^

Therapeutic : Anetumab

Best Alignment of Anetumab heavy chain to a sequence from OAS

QVELVQSGAEVKKPGESLKISCKGSGYSFTSYWIGWVRQAPGKGLEWMGIIDPGDSRTRYSPSFQGQVTISADKSISTAYLQWSSLKASDTAMYYCARGQLYG------GTYMDGWGQGTLVTVSS

.|.||||||||||||||||||||||||||||||||||||.|||||||||||.||||.||||||||||||||||||||||||||||||||||||||||||.||.......|||.|.|||||||||||

EVQLVQSGAEVKKPGESLKISCKGSGYSFTSYWIGWVRQMPGKGLEWMGIIYPGDSDTRYSPSFQGQVTISADKSISTAYLQWSSLKASDTAMYYCARGILYYDSSGYKGTYFDYWGQGTLVTVSS

^^^^^^^^ ^^^^^^^^ ^^^^^^^^^^^^^^^^^^^

Best Alignment of Anetumab light chain to a sequence from OAS

DIALTQPASVSGSPGQSITISCTGTSSDIGGYNSVSWYQQHPGKAPKLMIYGVNNRPSGVSNRFSGSKSGNTASLTISGLQAEDEADYYCSSYDIESATPVFGGGTKLTVL

..||||||||||||||||||||||||||.||||.|||||||||||||||||.|||||||||||||||||||||||||||||||||||||||||.|.|.|||||||||||||

QSALTQPASVSGSPGQSITISCTGTSSDVGGYNYVSWYQQHPGKAPKLMIYDVNNRPSGVSNRFSGSKSGNTASLTISGLQAEDEADYYCSSYTI-SSTPVFGGGTKLTVL

^^^^^^^^^ ^^^ ^^^^^^^^^^^

Best Alignment of Anetumab heavy chain CDRs to a sequence from OAS

QVELVQSGAEVKKPGESLKISCKGSGYSFTSYWIGWVRQAPGKGLEWMGIIDPGDSRTRYSPSFQGQVTISADKSISTAYLQWSSLKASDTAMYYCARGQLYGGTYMDGWGQGTLVTVSS

.............||||||||||||||||||||||||||.|||||||||||.||||.||||||||||||||||||||||||||||||||||||||||||..|||||.|||||||||||||

-------------PGESLKISCKGSGYSFTSYWIGWVRQMPGKGLEWMGIIYPGDSDTRYSPSFQGQVTISADKSISTAYLQWSSLKASDTAMYYCARGRDYGGTYLDGWGQGTLVTVSS

^^^^^^^^ ^^^^^^^^ ^^^^^^^^^^^^^

Best Alignment of Anetumab light chain CDRs to a sequence from OAS

DIALTQPASVSGSPGQSITISCTGTSSDIGGYNSVSWYQQHPGKAPKLMIYGVNNRPSGVSNRFSGSKSGNTASLTISGLQAEDEADYYCSSYDIESATPVFGGGTKLTVL

..|||||.|.|||||||.||||||.|||||||||||||||.||||||||||.||.|||||..||||||||||||||.|||||||||.||||||...||..|||||||||||

QSALTQPPSASGSPGQSVTISCTGSSSDIGGYNSVSWYQQFPGKAPKLMIYEVNKRPSGVPDRFSGSKSGNTASLTVSGLQAEDEAYYYCSSYTSTSAPGVFGGGTKLTVL

^^^^^^^^^ ^^^ ^^^^^^^^^^^

Best Alignment of Anetumab CDR-H3 to a sequence from OAS

QVELVQSGAEVKKPGESLKISCKGSGYSFTSYWIGWVRQAPGKGLEWMGIIDPGDSRTRYSPSFQGQVTISADKSISTAYLQWSSLKASDTAMYYCARGQLYGGTYMDGWGQGTLVTVSS

...............|.|...|...|.|...|...|.||.|||||||.|.|........||||.....|||.|.|.....|..||..|.|||.|||||||.|||||.|||||||||||||

--------------SETLSLTCAVCGGSLSGYYWSWIRQPPGKGLEWIGEILHS-GSNHYSPSLESRLTISVDTSKNQFSLKLSSVTAADTAVYYCARGQDYGGTYLDGWGQGTLVTVSS

^^^^^^^^ ^^^^^^^^ ^^^^^^^^^^^^^

Therapeutic : Anifrolumab

Best Alignment of Anifrolumab heavy chain to a sequence from OAS

EVQLVQSGAEVKKPGESLKISCKGSGYIFTNYWIAWVRQMPGKGLESMGIIYPGDSDIRYSPSFQGQVTISADKSITTAYLQWSSLKASDTAMYYCARHDI----EGFDYWGRGTLVTVSS

||||||||||||||||||||||||||||||.|||.|||||||||||.||||||||||.||||||||||||||||||.||||||||||||||||||||||.|.....||||||.||||||||

EVQLVQSGAEVKKPGESLKISCKGSGYIFTSYWIGWVRQMPGKGLEWMGIIYPGDSDTRYSPSFQGQVTISADKSISTAYLQWSSLKASDTAMYYCARHGIAAAGTGFDYWGQGTLVTVSS

^^^^^^^^ ^^^^^^^^ ^^^^^^^^^^^^^^

Best Alignment of Anifrolumab light chain to a sequence from OAS

EIVLTQSPGTLSLSPGERATLSCRASQSVSSSFFAWYQQKPGQAPRLLIYGASSRATGIPDRLSGSGSGTDFTLTITRLEPEDFAVYYCQQYDSSAITFGQGTRLEIK

||||||||||||||||||||||||||||||||..||||||||||||||||||||||||||||.|||||||||||||||||||||||||||||.|||||||||||||||

EIVLTQSPGTLSLSPGERATLSCRASQSVSSSYLAWYQQKPGQAPRLLIYGASSRATGIPDRFSGSGSGTDFTLTITRLEPEDFAVYYCQQYGSSAITFGQGTRLEIK

^^^^^^^ ^^^ ^^^^^^^^^

Best Alignment of Anifrolumab heavy chain CDRs to a sequence from OAS

EVQLVQSGAEVKKPGESLKISCKGSGYIFTNYWIAWVRQMPGKGLESMGIIYPGDSDIRYSPSFQGQVTISADKSITTAYLQWSSLKASDTAMYYCARHDIEGFDYWGRGTLVTVSS

..............|||||||||||||.||||||.|||||||||||.|||||||||||||||||||||||||||||.|||||||||||||||||||||..||.|||||.||||||||

--------------GESLKISCKGSGYSFTNYWIGWVRQMPGKGLEWMGIIYPGDSDIRYSPSFQGQVTISADKSISTAYLQWSSLKASDTAMYYCARRPIEEFDYWGQGTLVTVSS

^^^^^^^^ ^^^^^^^^ ^^^^^^^^^^

Best Alignment of Anifrolumab light chain CDRs to a sequence from OAS

EIVLTQSPGTLSLSPGERATLSCRASQSVSSSFFAWYQQKPGQAPRLLIYGASSRATGIPDRLSGSGSGTDFTLTITRLEPEDFAVYYCQQYDSSAITFGQGTRLEIK

||||||||.|||.|||||||||||||||||||||||||||.||||||||||||.|.||..||.|||||||||||||.||||.|.||||||||.||.|||||||||||.

EIVLTQSPDTLSVSPGERATLSCRASQSVSSSFFAWYQQKGGQAPRLLIYGASNRDTGVSDRFSGSGSGTDFTLTISRLEPDDSAVYYCQQYGSSPITFGQGTRLEIE

^^^^^^^ ^^^ ^^^^^^^^^

Best Alignment of Anifrolumab CDR-H3 to a sequence from OAS

EVQLVQSGAEVKKPGESLKISCKGSGYIFTNYWIAWVRQMPGKGLESMGIIYPGDSDIRYSPSFQGQVTISADKSITTAYLQWSSLKASDTAMYYCARHDIEGFDYWGRGTLVTVSS

............||.|.|...|..||.....|...|.||.||||||..|.||.......|.||....||||.|.|.....|..||..|.|||.|||||||.|||||||.||||||||

---------GLVKPSETLSLTCTVSGGSISSYYWSWIRQPPGKGLEWIGYIYYS-GSTNYNPSLKSRVTISVDTSKNQFSLKLSSVTAADTAVYYCARHDREGFDYWGQGTLVTVSS

^^^^^^^^ ^^^^^^^^ ^^^^^^^^^^

Therapeutic : Anrukinzumab

Best Alignment of Anrukinzumab heavy chain to a sequence from OAS

EVQLVESGGGLVQPGGSLRLSCAASGFTFISYAMSWVRQAPGKGLEWVASISSG-GNTYYPDSVKGRFTISRDNAKNSLYLQMNSLRAEDTAVYYCARLDGYYFGFAYWGQGTLVTVSS

|||||||||||||||||||||||||||||.||||||||||||||||||..||...|.|||.|||||||||||||||||||||||||||||||||||||..|.|.||.||||||||||||

EVQLVESGGGLVQPGGSLRLSCAASGFTFSSYAMSWVRQAPGKGLEWVSAISGSGGSTYYADSVKGRFTISRDNAKNSLYLQMNSLRAEDTAVYYCARGSGWYAGFDYWGQGTLVTVSS

^^^^^^^^ ^^^^^^^^ ^^^^^^^^^^^^

Best Alignment of Anrukinzumab light chain to a sequence from OAS

DIQMTQSPSSLSASVGDRVTITCKASESVDNYGKSLMHWYQQKPGKAPKLLIYRASNLESGVPSRFSGSGSGTDFTLTISSLQPEDFATYYCQQSNEDPWTFGGGTKVEIK

|||||||||||||||||||||||.||||......|...|||||||||||||||.||.|||||||||||||||||||||||||||||||||||||||..|.|||||||||||

DIQMTQSPSSLSASVGDRVTITCRASESI----SSYLNWYQQKPGKAPKLLIYAASSLESGVPSRFSGSGSGTDFTLTISSLQPEDFATYYCQQSNSNPLTFGGGTKVEIK

^^^^^^^^^^ ^^^ ^^^^^^^^^

Best Alignment of Anrukinzumab heavy chain CDRs to a sequence from OAS

EVQLVESGGGLVQPGGSLRLSCAASGFTFISYAMSWVRQAPGKGLEWVASISSGGNTYYPDSVKGRFTISRDNAKNSLYLQMNSLRAEDTAVYYCARLDGYYFGFAYWGQGTLVTVSS

||.|||||||||.|||||.||||||||||.|||||||||.|.|.|||||||||||.||||||||||||||||||.|.|||||.|||.||||.|||||.||||.||||||||||||||.

EVKLVESGGGLVKPGGSLKLSCAASGFTFSSYAMSWVRQTPEKRLEWVASISSGGSTYYPDSVKGRFTISRDNARNILYLQMSSLRSEDTAMYYCARSDGYYGGFAYWGQGTLVTVSA

^^^^^^^^ ^^^^^^^ ^^^^^^^^^^^^

Best Alignment of Anrukinzumab light chain CDRs to a sequence from OAS

DIQMTQSPSSLSASVGDRVTITCKASESVDNYGKSLMHWYQQKPGKAPKLLIYRASNLESGVPSRFSGSGSGTDFTLTISSLQPEDFATYYCQQSNEDPWTFGGGTKVEIK

||..||||.||..|...|..|.|.||||||.||||.|||||||||..||||||||||||||.|.|||||||.|||||||......|.||||||||||||||||||||.|||

DILLTQSPASLAVSLEQRASIACRASESVDSYGKSFMHWYQQKPGQPPKLLIYRASNLESGIPARFSGSGSRTDFTLTINPVEADDVATYYCQQSNEDPWTFGGGTKLEIK

^^^^^^^^^^ ^^^ ^^^^^^^^^

Best Alignment of Anrukinzumab CDR-H3 to a sequence from OAS

EVQLVESGGGLVQPGGSLRLSCAASGFTFISYAMSWVRQAPGKGLEWVASISSG-GNTYYPDSVKGRFTISRDNAKNSLYLQMNSLRAEDTAVYYCARLDGYYFGFAYWGQGTLVTVSS

||||..||..||.||.|..|||.||||....|.|.||.|....||||...|....|.|.|.....|..||..|...|..|||..||..|||||||||||||||.||||||||||||||.

EVQLQQSGAELVKPGASVKLSCTASGFNIKDYYMHWVKQRTEQGLEWIGRIDPEDGETKYAPKFQGKATITADTSSNTAYLQLSSLTSEDTAVYYCARLDGYYEGFAYWGQGTLVTVSA

^^^^^^^^ ^^^^^^^^ ^^^^^^^^^^^^

Therapeutic : Ascrinvacumab

Best Alignment of Ascrinvacumab heavy chain to a sequence from OAS

QVQLQESGPGLVKPSQTLSLTCTVSGGSISSGEYYWNWIRQHPGKGLEWIGYIYYSGSTYYNPSLKSRVTISVDTSKNQFSLKLSSVTAADTAVYYCARESV--AGFDYWGQGTLVTVSS

||||||||||||||||||||||||||||||||.|||.|||||||||||||||||||||||||||||||||||||||||||||||||||||||||||||||||..|.||||||||||||||

QVQLQESGPGLVKPSQTLSLTCTVSGGSISSGGYYWSWIRQHPGKGLEWIGYIYYSGSTYYNPSLKSRVTISVDTSKNQFSLKLSSVTAADTAVYYCARESVDTANFDYWGQGTLVTVSS

^^^^^^^^^^ ^^^^^^^ ^^^^^^^^^^^^

Best Alignment of Ascrinvacumab light chain to a sequence from OAS

EIVLTQSPGTLSLSPGERATLSCRASQSVSSSYLAWYQQKPGQAPRLLIYGTSSRATGIPDRFSGSGSGTDFTLTISRLEPEDFAVYYCQQYGSSPITFGQGTRLEIK

||||||||||||||||||||||||||||||||||||||||||||||||||||||||||||||||||||||||||||||||||||||||||||||||||||||||||||

EIVLTQSPGTLSLSPGERATLSCRASQSVSSSYLAWYQQKPGQAPRLLIYGTSSRATGIPDRFSGSGSGTDFTLTISRLEPEDFAVYYCQQYGSSPITFGQGTRLEIK

^^^^^^^ ^^^ ^^^^^^^^^

Best Alignment of Ascrinvacumab heavy chain CDRs to a sequence from OAS

QVQLQESGPGLVKPSQTLSLTCTVSGGSISSGEYYWNWIRQHPGKGLEWIGYIYYSGSTYYNPSLKSRVTISVDTSKNQFSLKLSSVTAADTAVYYCARESVAGFDYWGQGTLVTVSS

||||||||||||.|||||||||.|||||||||||||.||||.|||||||||||||||||.||||||||.|.|.||||.||||.|.||||||||||||||.||||||||||||||||||

QVQLQESGPGLVNPSQTLSLTCNVSGGSISSGEYYWTWIRQPPGKGLEWIGYIYYSGSTSYNPSLKSRLTLSLDTSKIQFSLRLASVTAADTAVYYCARVSVAGFDYWGQGTLVTVSS

^^^^^^^^^^ ^^^^^^^ ^^^^^^^^^^

Best Alignment of Ascrinvacumab light chain CDRs to a sequence from OAS

EIVLTQSPGTLSLSPGERATLSCRASQSVSSSYLAWYQQKPGQAPRLLIYGTSSRATGIPDRFSGSGSGTDFTLTISRLEPEDFAVYYCQQYGSSPITFGQGTRLEIK

|.|||.||||||.|||||||||||||||||||||||||||||||||||||||||||||||||||||||||||||||||||||||||||||||||||||||||||||||

ELVLTLSPGTLSFSPGERATLSCRASQSVSSSYLAWYQQKPGQAPRLLIYGTSSRATGIPDRFSGSGSGTDFTLTISRLEPEDFAVYYCQQYGSSPITFGQGTRLEIK

^^^^^^^ ^^^ ^^^^^^^^^

Best Alignment of Ascrinvacumab CDR-H3 to a sequence from OAS

QVQLQESGPGLVKPSQTLSLTCTVSGGSISSGEYYWNWIRQHPGKGLEWIGYIYYSGSTYYNPSLKSRVTISVDTSKNQFSLKLSSVTAADTAVYYCARESVAGFDYWGQGTLVTVSS

.................|.|.|..||.......|...|.||..||||||...|...|.|||..|.|.|.|||....||...|...|..|.||||||||||||||||||||||||||||

----------------SLRLSCAASGFTF--SSYDMHWVRQATGKGLEWVSAIGTAGDTYYPGSVKGRFTISRENAKNSLYLQMNSLRAGDTAVYYCARESVAGFDYWGQGTLVTVSS

^^^^^^^^^^ ^^^^^^^ ^^^^^^^^^^

Therapeutic : Atezolizumab

Best Alignment of Atezolizumab heavy chain to a sequence from OAS

EVQLVESGGGLVQPGGSLRLSCAASGFTFSDSWIHWVRQAPGKGLEWVAWISPYGGSTYYADSVKGRFTISADTSKNTAYLQMNSLRAEDTAVYYCARRHWP------GGFDYWGQGTLVTVSS

||||||||||||||||||||||||||||||..|.||||||||||||||..||..|||||||||||||||||.|.||||.||||||||||||||||||||.........|.||||||||||||||

EVQLVESGGGLVQPGGSLRLSCAASGFTFSSYWMHWVRQAPGKGLEWVSAISGSGGSTYYADSVKGRFTISRDNSKNTLYLQMNSLRAEDTAVYYCARRYYDSSGYPGGDFDYWGQGTLVTVSS

^^^^^^^^ ^^^^^^^^ ^^^^^^^^^^^^^^^^^

Best Alignment of Atezolizumab light chain to a sequence from OAS

DIQMTQSPSSLSASVGDRVTITCRASQDVSTAVAWYQQKPGKAPKLLIYSASFLYSGVPSRFSGSGSGTDFTLTISSLQPEDFATYYCQQYLYHPATFGQGTKVEIK

|||||||||||||||||||||||||||..||..||||||||||||||||.||.|.||||||||||||||||||||||||||||||||||||...|.|||||||||||

DIQMTQSPSSLSASVGDRVTITCRASQGISTGLAWYQQKPGKAPKLLIYRASSLQSGVPSRFSGSGSGTDFTLTISSLQPEDFATYYCQQYNSTPPTFGQGTKVEIK

^^^^^^ ^^^ ^^^^^^^^^

Best Alignment of Atezolizumab heavy chain CDRs to a sequence from OAS

EVQLVESGGGLVQPGGSLRLSCAASGFTFSDSWIHWVRQAPGKGLEWVAWISPYGGSTYYADSVKGRFTISADTSKNTAYLQMNSLRAEDTAVYYCARRHWPGGFDYWGQGTLVTVSS

||.|||||||||||||||.||||||||||||....||||.|.|.|||||.||..||||||.|.||||||||.|..|||.||||..|..||||.||||||.|.||||||||||..||||

EVMLVESGGGLVQPGGSLKLSCAASGFTFSDYYMYWVRQTPEKRLEWVAYISNGGGSTYYPDTVKGRFTISRDNAKNTLYLQMSRLKSEDTAMYYCARRNWDGGFDYWGQGTTLTVSS

^^^^^^^^ ^^^^^^^^ ^^^^^^^^^^^

Best Alignment of Atezolizumab light chain CDRs to a sequence from OAS

DIQMTQSPSSLSASVGDRVTITCRASQDVSTAVAWYQQKPGKAPKLLIYSASFLYSGVPSRFSGSGSGTDFTLTISSLQPEDFATYYCQQYLYHPATFGQGTKVEIK

|...|.........|||||.|||.|||||||||||||||||..|||||||||....|||.||.|||||||||||||..|.||.|.||||||...|.|||.|||.|||

DFVLTRPKNFIPTPVGDRVSITCKASQDVSTAVAWYQQKPGQSPKLLIYSASNRHTGVPDRFTGSGSGTDFTLTISNVQAEDLADYYCQQYYSYPRTFGGGTKLEIK

^^^^^^ ^^^ ^^^^^^^^^

Best Alignment of Atezolizumab CDR-H3 to a sequence from OAS

EVQLVESGGGLVQPGGSLRLSCAASGFTFSDSWIHWVRQAPGKGLEWVAWISPYGGSTYYADSVKGRFTISADTSKNTAYLQMNSLRAEDTAVYYCARRHWPGGFDYWGQGTLVTVSS

................|||||||||||..|.....|||||||||||||..|....||.|||||||||||||.|.||||.|||||||||.||||||||||||.||||||||||||||||

----------------SLRLSCAASGFSVSTNYMSWVRQAPGKGLEWVSLIDSG-GSSYYADSVKGRFTISRDNSKNTLYLQMNSLRADDTAVYYCARRHWNGGFDYWGQGTLVTVSS

^^^^^^^^ ^^^^^^^^ ^^^^^^^^^^^

Therapeutic : Atidortoxumab

Best Alignment of Atidortoxumab heavy chain to a sequence from OAS

EVQLQESGPGLVRPSETLSLTCAVSGYSISSGMGWGWIRQPPGKGLEWIGSIDQRGSTYYNPSLKSRVTISVDTSKNQFSLKLSSVTAADTAVYYCARDAGH--AVDMDVWGKGTTVTVSS

.|||||||||||.|||||||||||||||||||..|||||||||||||||||||..|||||||||||||||||||||||||||||||||||||||||||..||.....||||||||||||||

QVQLQESGPGLVKPSETLSLTCAVSGYSISSGYYWGWIRQPPGKGLEWIGSIDHSGSTYYNPSLKSRVTISVDTSKNQFSLKLSSVTAADTAVYYCARHSGHRRHYYMDVWGKGTTVTVSS

^^^^^^^^^ ^^^^^^^ ^^^^^^^^^^^^^^

Best Alignment of Atidortoxumab light chain to a sequence from OAS

DIQMTQSPSSVSASVGDRVTITCRASQGISRWLAWYQQKPGKAPKLLIYAASSLQSGVPSRFSGSGSGTDFTLTISSLQPEDFATYYCQQGYVFPLTFGGGTKVEIK

||||||||||||||||||||||||||||||||||||||||||||||||||||||||||||||||||||||||||||||||||||||||||.|.||||||||||||||

DIQMTQSPSSVSASVGDRVTITCRASQGISRWLAWYQQKPGKAPKLLIYAASSLQSGVPSRFSGSGSGTDFTLTISSLQPEDFATYYCQQAYSFPLTFGGGTKVEIK

^^^^^^ ^^^ ^^^^^^^^^

Best Alignment of Atidortoxumab heavy chain CDRs to a sequence from OAS

EVQLQESGPGLVRPSETLSLTCAVSGYSISSGMGWGWIRQPPGKGLEWIGSIDQRGSTYYNPSLKSRVTISVDTSKNQFSLKLSSVTAADTAVYYCARDAGHAVDMDVWGKGTTVTVSS

.........|||.|||||||||.|||||||||..||||||||||||||||||...||||||.|||||||||||||||||||||.|||||||||||||||.||...|||||.||||||||

---------GLVKPSETLSLTCTVSGYSISSGYFWGWIRQPPGKGLEWIGSIYHSGSTYYNSSLKSRVTISVDTSKNQFSLKLGSVTAADTAVYYCARDGGHYFGMDVWGQGTTVTVSS

^^^^^^^^^ ^^^^^^^ ^^^^^^^^^^^^

Best Alignment of Atidortoxumab light chain CDRs to a sequence from OAS

DIQMTQSPSSVSASVGDRVTITCRASQGISRWLAWYQQKPGKAPKLLIYAASSLQSGVPSRFSGSGSGTDFTLTISSLQPEDFATYYCQQGYVFPLTFGGGTKVEIK

||||||||||.||||||.||||||||||||||..|||||||||||||.|||||.|||.|||||||||||...||||||||||||||||||||..||||||||||.||

DIQMTQSPSSLSASVGDKVTITCRASQGISRWVDWYQQKPGKAPKLLSYAASSWQSGAPSRFSGSGSGTVYPLTISSLQPEDFATYYCQQGYNTPLTFGGGTKVAIK

^^^^^^ ^^^ ^^^^^^^^^

Best Alignment of Atidortoxumab CDR-H3 to a sequence from OAS

EVQLQESGPGLVRPSETLSLTCAVSGYSISSGMGWGWIRQPPGKGLEWIGSIDQR-GSTYYNPSLKSRVTISVDTSKNQFSLKLSSVTAADTAVYYCARDAGHAVDMDVWGKGTTVTVSS

...............|.|...||.||..........|.||.|||||||...|....|...|..|...|.|.|.|.||....|...|....||||||||||.|.|||||||||||||.|||

--------------GESLKISCATSGFIF-RDYAMHWVRQAPGKGLEWVALISFDRGTQHYADSVRGRFTVSSDSSKDTVFLQMNSLRPEDTAVYYCARDRGIAVDMDVWGKGTTVIVSS

^^^^^^^^^ ^^^^^^^^ ^^^^^^^^^^^^

Therapeutic : Avelumab

Best Alignment of Avelumab heavy chain to a sequence from OAS

EVQLLESGGGLVQPGGSLRLSCAASGFTFSSYIMMWVRQAPGKGLEWVSSIYPSGGITFYADTVKGRFTISRDNSKNTLYLQMNSLRAEDTAVYYCARIKLGT-VTTVDYWGQGTLVTVSS

||||||||||||||||||||||||||||||||.|.||||||||||||||.|..|||.|.|||.|||||||||||||||||||||||||||||||||||..||..|||.|||||||||||||

EVQLLESGGGLVQPGGSLRLSCAASGFTFSSYAMSWVRQAPGKGLEWVSAISGSGGSTYYADSVKGRFTISRDNSKNTLYLQMNSLRAEDTAVYYCARDHLGATVTTADYWGQGTLVTVSS

^^^^^^^^ ^^^^^^^^ ^^^^^^^^^^^^^^

Best Alignment of Avelumab light chain to a sequence from OAS

QSALTQPASVSGSPGQSITISCTGTSSDVGGYNYVSWYQQHPGKAPKLMIYDVSNRPSGVSNRFSGSKSGNTASLTISGLQAEDEADYYCSSYTSSSTRVFGTGTKVTVL

||||||||||||||||||||||||||||||||||||||||||||||||||||||||||||||||||||||||||||||||||||||||||||||||||||||||||||||

QSALTQPASVSGSPGQSITISCTGTSSDVGGYNYVSWYQQHPGKAPKLMIYDVSNRPSGVSNRFSGSKSGNTASLTISGLQAEDEADYYCSSYTSSSTRVFGTGTKVTVL

^^^^^^^^^ ^^^ ^^^^^^^^^^

Best Alignment of Avelumab heavy chain CDRs to a sequence from OAS

EVQLLESGGGLVQPGGSLRLSCAASGFTFSSYIMMWVRQAPGKGLEWVSSIYPSGGITFYADTVKGRFTISRDNSKNTLYLQMNSLRAEDTAVYYCARIKLGTVTTVDYWGQGTLVTVSS

................||||||||||||||||.|.||||||||||||||.|..|||.|.|||.|||||||||||.||.||||||||||||||||||||...|||||||||||||||||||

----------------SLRLSCAASGFTFSSYAMSWVRQAPGKGLEWVSAISGSGGSTYYADSVKGRFTISRDNAKNSLYLQMNSLRAEDTAVYYCARSSGGTVTTVDYWGQGTLVTVSS

^^^^^^^^ ^^^^^^^^ ^^^^^^^^^^^^^

Best Alignment of Avelumab light chain CDRs to a sequence from OAS

QSALTQPASVSGSPGQSITISCTGTSSDVGGYNYVSWYQQHPGKAPKLMIYDVSNRPSGVSNRFSGSKSGNTASLTISGLQAEDEADYYCSSYTSSSTRVFGTGTKVTVL

|||||||||||||||||||||||||||||||||||||.|||||||||||||||||||||||||||||||||||||||||||.||||||||||||||||||||.|||.|||

QSALTQPASVSGSPGQSITISCTGTSSDVGGYNYVSWCQQHPGKAPKLMIYDVSNRPSGVSNRFSGSKSGNTASLTISGLQTEDEADYYCSSYTSSSTRVFGGGTKLTVL

^^^^^^^^^ ^^^ ^^^^^^^^^^

Best Alignment of Avelumab CDR-H3 to a sequence from OAS

EVQLLESGGGLVQPGGSLRLSCAASGFTF--SSYIMMWVRQAPGKGLEWVSSIYPSGGITFYADTVKGRFTISRDNSKNTLYLQMNSLRAEDTAVYYCARIKLGTVTTVDYWGQGTLVTVSS

.............|...|.|.|..|||....|.....|.||.|||.|||...|........|....|.|.|||.|.|||...|.|......|||.|||||||..||||||||||||||||||

------------KPTQTLTLTCTFSGFSLSTSGMCVSWIRQPPGKALEWLARIDWD-DDKYYSTSLKTRLTISKDTSKNQVVLTMTNMDPVDTATYYCARIKMTTVTTVDYWGQGTLVTVSS

^^^^^^^^^^ ^^^^^^^^ ^^^^^^^^^^^^^

Therapeutic : Bapineuzumab

Best Alignment of Bapineuzumab heavy chain to a sequence from OAS

EVQLLESGGGLVQPGGSLRLSCAASGFTFSNYGMSWVRQAPGKGLEWVASIRSGGGRTYYSDNVKGRFTISRDNSKNTLYLQMNSLRAEDTAVYYCVRYDHY-SGSSDYWGQGTLVTVSS

||||||||||||||||||||||||||||||.|.|||||||||||||||..|.|||..|||.|.|||||||||||||||||||||||||||||||||...|.|.|||||||||||||||||

EVQLLESGGGLVQPGGSLRLSCAASGFTFSSYAMSWVRQAPGKGLEWVSVIYSGGSSTYYADSVKGRFTISRDNSKNTLYLQMNSLRAEDTAVYYCAKVDYYGSGSSDYWGQGTLVTVSS

^^^^^^^^ ^^^^^^^^ ^^^^^^^^^^^^^

Best Alignment of Bapineuzumab light chain to a sequence from OAS

DVVMTQSPLSLPVTPGEPASISCKSSQSLLDSDGKTYLNWLLQKPGQSPQRLIYLVSKLDSGVPDRFSGSGSGTDFTLKISRVEAEDVGVYYCWQGTHFPRTFGQGTKVEIK

|||||||||.|.||.|.||||||||||||||||||||||||||.|||||.|||||||||||||||||.|||||||||||||||||||.||||||||||||||||.|||.|||

DVVMTQSPLTLSVTIGQPASISCKSSQSLLDSDGKTYLNWLLQRPGQSPKRLIYLVSKLDSGVPDRFTGSGSGTDFTLKISRVEAEDLGVYYCWQGTHFPRTFGGGTKLEIK

^^^^^^^^^^^ ^^^ ^^^^^^^^^

Best Alignment of Bapineuzumab heavy chain CDRs to a sequence from OAS

EVQLLESGGGLVQPGGSLRLSCAASGFTFSNYGMSWVRQAPGKGLEWVASIRSGGGRTYYSDNVKGRFTISRDNSKNTLYLQMNSLRAEDTAVYYCVRYDHYSGSSDYWGQGTLVTVSS

||.|.|||||||||||||.|||||||||||.|.|.||||.|.|.|||||.||.|||||||.|.|||||||||||.||||||||..|..||||.|||.|..||.||||||||||..||||

EVMLVESGGGLVQPGGSLKLSCAASGFTFSDYYMYWVRQTPEKRLEWVAYIRNGGGRTYYPDTVKGRFTISRDNAKNTLYLQMSRLKSEDTAMYYCARGGHYYGSSDYWGQGTTLTVSS

^^^^^^^^ ^^^^^^^^ ^^^^^^^^^^^^

Best Alignment of Bapineuzumab light chain CDRs to a sequence from OAS

DVVMTQSPLSLPVTPGEPASISCKSSQSLLDSDGKTYLNWLLQKPGQSPQRLIYLVSKLDSGVPDRFSGSGSGTDFTLKISRVEAEDVGVYYCWQGTHFPRTFGQGTKVEIK

||||||.||.|.||.|.|||.||||||||||||||||||||||.|||||.|||||||||||||||||.|||||||||||||||||||.||||||||||||||||.|||.|||

DVVMTQTPLTLSVTIGQPASFSCKSSQSLLDSDGKTYLNWLLQRPGQSPKRLIYLVSKLDSGVPDRFTGSGSGTDFTLKISRVEAEDLGVYYCWQGTHFPRTFGGGTKLEIK

^^^^^^^^^^^ ^^^ ^^^^^^^^^

Best Alignment of Bapineuzumab CDR-H3 to a sequence from OAS

EVQLLESGGGLVQPGGSLRLSCAASGFTFSNYGMSWVRQAPGKGLEWVASIRSGGGRTYYSDNVKGRFTISRDNSKNTLYLQMNSLRAEDTAVYYCVRYDHYSGSSDYWGQGTLVTVSS

.|||...|..||.||.|..|||.|||.||..|.|.||.|.||.||||...|....|.|.|....|...|...|.|..|.|.|..||..||.|||||.|.||||||||||||||..||||

QVQLQQPGAELVKPGASVKLSCKASGYTFTSYWMHWVKQRPGQGLEWIGMIHPNSGSTNYNEKFKSKATLTVDKSSSTAYMQLSSLTSEDSAVYYCARCDHYSGSSDYWGQGTTLTVSS

^^^^^^^^ ^^^^^^^^ ^^^^^^^^^^^^

Therapeutic : Basiliximab

Best Alignment of Basiliximab heavy chain to a sequence from OAS

QVQLQQSGTVLARPGASVKMSCKASGYSFTRYWMHWIKQRPGQGLEWIGAIYPGNSDTSYNQKFEGKAKLTAVTSASTAYMELSSLTHEDSAVYYCSRDYG--YYFDFWGQGTTLTVSS

.|||||||||||||||||||||||||||||.|||||.|||||||||||||||||||||||||||.||||||||||||||||||||||.||||||||.||||..||||.|||||||||||

EVQLQQSGTVLARPGASVKMSCKASGYSFTSYWMHWVKQRPGQGLEWIGAIYPGNSDTSYNQKFKGKAKLTAVTSASTAYMELSSLTNEDSAVYYCTRDYGSPYYFDYWGQGTTLTVSS

^^^^^^^^ ^^^^^^^^ ^^^^^^^^^^^^

Best Alignment of Basiliximab light chain to a sequence from OAS

QIVSTQSPAIMSASPGEKVTMTCSASSSRSYMQWYQQKPGTSPKRWIYDTSKLASGVPARFSGSGSGTSYSLTISSMEAEDAATYYCHQRSSYTFGGGTKLEIK

|||.||||||||||||||||||||||||.|||.|||||||||||||||||||||||||||||||||||||||||||||||||||||||||||||||||||||||

QIVLTQSPAIMSASPGEKVTMTCSASSSISYMHWYQQKPGTSPKRWIYDTSKLASGVPARFSGSGSGTSYSLTISSMEAEDAATYYCHQRSSYTFGGGTKLEIK

^^^^^ ^^^ ^^^^^^^

Best Alignment of Basiliximab heavy chain CDRs to a sequence from OAS

QVQLQQSGTVLARPGASVKMSCKASGYSFTRYWMHWIKQRPGQGLEWIGAIYPGNSDTSYNQKFEGKAKLTAVTSASTAYMELSSLTHEDSAVYYCSRDYGYYFDFWGQGTTLTVSS

.|.||.||||||||||||||||||||||||.|||||.|||||||||||||||||||||||||||.||||||||||||||||.|||||.||||||||.||||||||.|||||||||||

EVKLQESGTVLARPGASVKMSCKASGYSFTSYWMHWVKQRPGQGLEWIGAIYPGNSDTSYNQKFKGKAKLTAVTSASTAYMQLSSLTSEDSAVYYCARDYGYYFDYWGQGTTLTVSS

^^^^^^^^ ^^^^^^^^ ^^^^^^^^^^

Best Alignment of Basiliximab light chain CDRs to a sequence from OAS

QIVSTQSPAIMSASPGEKVTMTCSASSSRSYMQWYQQKPGTSPKRWIYDTSKLASGVPARFSGSGSGTSYSLTISSMEAEDAATYYCHQRSSYTFGGGTKLEIK

||..||||||||||||||||||||||||.|||.|||||||||||||||||||||.|||||||||||||||.|||||||||||||||.|||||||||||||||||

QILLTQSPAIMSASPGEKVTMTCSASSSISYMHWYQQKPGTSPKRWIYDTSKLAAGVPARFSGSGSGTSYALTISSMEAEDAATYYGHQRSSYTFGGGTKLEIK

^^^^^ ^^^ ^^^^^^^

Best Alignment of Basiliximab CDR-H3 to a sequence from OAS

QVQLQQSGTVLARPGASVKMSCKASGYSFTRYWMHWIKQRPGQGLEWIGAIYPGNSDTSYNQKFEGKAKLTAVTSASTAYMELSSLTHEDSAVYYCSRDYGYYFDFWGQGTTLTVSS

||...|.|..|..||||||.|||||||.||.|||||.||||||||||||.|.|....|.||.||..||.||...|.|||||.|||||.||||||.||||||||||.|||||||||||

QVHVKQPGAELVKPGASVKLSCKASGYTFTSYWMHWVKQRPGQGLEWIGMIHPNSGSTNYNEKFKSKATLTVDKSSSTAYMQLSSLTSEDSAVYSCSRDYGYYFDYWGQGTTLTVSS

^^^^^^^^ ^^^^^^^^ ^^^^^^^^^^

Therapeutic : Bavituximab

Best Alignment of Bavituximab heavy chain to a sequence from OAS

EVQLQQSGPELEKPGASVKLSCKASGYSFTGYNMNWVKQSHGKSLEWIGHIDPYYGDTSYNQKFRGKATLTVDKSSSTAYMQLKSLTSEDSAVYYCVKGGYYG--HWYFDVWGAGTTVTVSS

|||||||||||||||||||.||||||||||||||||||||.||||||||.||||||.|||||||.|||||||||||||||||||||||||||||||..|||||..|||||||||||||||||

EVQLQQSGPELEKPGASVKISCKASGYSFTGYNMNWVKQSNGKSLEWIGNIDPYYGGTSYNQKFKGKATLTVDKSSSTAYMQLKSLTSEDSAVYYCARGGYYGSSHWYFDVWGAGTTVTVSS

^^^^^^^^ ^^^^^^^^ ^^^^^^^^^^^^^^^

Best Alignment of Bavituximab light chain to a sequence from OAS

DIQMTQSPSSLSASLGERVSLTCRASQDIGSSLNWLQQGPDGTIKRLIYATSSLDSGVPKRFSGSRSGSDYSLTISSLESEDFVDYYCLQYVSSPPTFGAGTKLELK

||||||||||||||||||||||||||||||||||||||.||||||||||||||||||||||||||||||||||||||||||||||||||||.|||||||||||||||

DIQMTQSPSSLSASLGERVSLTCRASQDIGSSLNWLQQEPDGTIKRLIYATSSLDSGVPKRFSGSRSGSDYSLTISSLESEDFVDYYCLQYASSPPTFGAGTKLELK

^^^^^^ ^^^ ^^^^^^^^^

Best Alignment of Bavituximab heavy chain CDRs to a sequence from OAS

EVQLQQSGPELEKPGASVKLSCKASGYSFTGYNMNWVKQSHGKSLEWIGHIDPYYGDTSYNQKFRGKATLTVDKSSSTAYMQLKSLTSEDSAVYYCVKGGYYGHWYFDVWGAGTTVTVSS

.|||||.|.||..|||||||||||||||||||||||||||.||||||||.||||||.|||||||.|||||||||||||||||||||||||||||||...||||.||||||||||||||||

-VQLQQPGAELVRPGASVKLSCKASGYSFTGYNMNWVKQSNGKSLEWIGNIDPYYGGTSYNQKFKGKATLTVDKSSSTAYMQLKSLTSEDSAVYYCARFGYYGNWYFDVWGAGTTVTVSS

^^^^^^^^ ^^^^^^^^ ^^^^^^^^^^^^^

Best Alignment of Bavituximab light chain CDRs to a sequence from OAS

DIQMTQSPSSLSASLGERVSLTCRASQDIGSSLNWLQQGPDGTIKRLIYATSSLDSGVPKRFSGSRSGSDYSLTISSLESEDFVDYYCLQYVSSPPTFGAGTKLELK

|||||||||||||||||||||||||.||||||.|||||.||||.|||||||||||||.||||||||||||||||||||||||.||||||||.|||||||.|||||.|

DIQMTQSPSSLSASLGERVSLTCRARQDIGSSVNWLQQEPDGTNKRLIYATSSLDSGDPKRFSGSRSGSDYSLTISSLESEDCVDYYCLQYASSPPTFGGGTKLESK

^^^^^^ ^^^ ^^^^^^^^^

Best Alignment of Bavituximab CDR-H3 to a sequence from OAS

EVQLQQSGPELEKPGASVKLSCKASGYSFTGYNMNWVKQSHGKSLEWIGHIDPYYGDTSYNQKFRGKATLTVDKSSSTAYMQLKSLTSEDSAVYYCVKGGYYGHWYFDVWGAGTTVTVSS

|||||||||||.|||||.|.||||||||||||.||||||||||.|||||.|.||.|.|||||||.||||||||.|||||||.|.||||||||||||.|||||||||||||||||||||||

EVQLQQSGPELVKPGASMKISCKASGYSFTGYTMNWVKQSHGKNLEWIGLINPYNGGTSYNQKFKGKATLTVDESSSTAYMELLSLTSEDSAVYYCAKGGYYGHWYFDVWGAGTTVTVSS

^^^^^^^^ ^^^^^^^^ ^^^^^^^^^^^^^

Therapeutic : Belimumab

Best Alignment of Belimumab heavy chain to a sequence from OAS

QVQLQQSGAEVKKPGSSVRVSCKASGGTFNNNAINWVRQAPGQGLEWMGGIIPMFGTAKYSQNFQGRVAITADESTGTASMELSSLRSEDTAVYYCARSRDLLL-----FPHHALSPWGRGTMVTVSS

||||.|||||||||||||.||||||||||...||.||||||||||||||||||.||||.|.|.|||||.||||||||||.||||||||||||||||||.|..|........|||...||.||||||||

QVQLVQSGAEVKKPGSSVKVSCKASGGTFSSYAISWVRQAPGQGLEWMGGIIPIFGTANYAQKFQGRVTITADESTGTAYMELSSLRSEDTAVYYCARDRQVLRYFDWLDRHHAFDIWGQGTMVTVSS

^^^^^^^^ ^^^^^^^^ ^^^^^^^^^^^^^^^^^^^^^

Best Alignment of Belimumab light chain to a sequence from OAS

SSELTQDPAVSVALGQTVRVTCQGDSLRSYYASWYQQKPGQAPVLVIYGKNNRPSGIPDRFSGSSSGNTASLTITGAQAEDEADYYCSSRDSSGNHWVFGGGTELTVL

|||||||||||||||||||||||||||||||||||||||||||||||||||||||||||||||||||||||||||||||||||||||.|||||||||||||||.||||

SSELTQDPAVSVALGQTVRVTCQGDSLRSYYASWYQQKPGQAPVLVIYGKNNRPSGIPDRFSGSSSGNTASLTITGAQAEDEADYYCNSRDSSGNHWVFGGGTKLTVL

^^^^^^ ^^^ ^^^^^^^^^^^

Best Alignment of Belimumab heavy chain CDRs to a sequence from OAS

QVQLQQSGAEVKKPGSSVRVSCKASGGTFNNNAINWVRQAPGQGLEWMGGIIPMFGTAKYSQNFQGRVAITADESTGTASMELSSLRSEDTAVYYCARSRDLLLFPHHALSPWGRGTMVTVSS

................||.||||||||||...||.||||||||||||||||||.||||.|.|.|||||..|.|.||.||.|||.|||..|||||||||..|..|.||.||..||.||||||||

----------------SVKVSCKASGGTFSSYAISWVRQAPGQGLEWMGGIIPIFGTANYAQKFQGRVTMTTDTSTSTAYMELRSLRFDDTAVYYCARFVDRALAPHGALDIWGQGTMVTVSS

^^^^^^^^ ^^^^^^^^ ^^^^^^^^^^^^^^^^

Best Alignment of Belimumab light chain CDRs to a sequence from OAS

SSELTQDPAVSVALGQTVRVTCQGDSLRSYYASWYQQKPGQAPVLVIYGKNNRPSGIPDRFSGSSSGNTASLTITGAQAEDEADYYCSSRDSSGNHWVFGGGTELTVL

|||||||||||||||||||.||||||||||||||||||||||||||||||||||||||||||||||||||||||||||||||||||||||||||||||||.||..|||

SSELTQDPAVSVALGQTVRITCQGDSLRSYYASWYQQKPGQAPVLVIYGKNNRPSGIPDRFSGSSSGNTASLTITGAQAEDEADYYCSSRDSSGNHWVFGTGTKVTVL

^^^^^^ ^^^ ^^^^^^^^^^^

Best Alignment of Belimumab CDR-H3 to a sequence from OAS

QVQLQQSGAEVKKPGSSVRVSCKASGGTFNNNAINWVRQAPGQGLEWMGGIIPMFGTAKYSQNFQGRVAITADESTGTASMELSSLRSEDTAVYYCARSRDLLLFPHHALSPWGRGTMVTVSS

.|||||||.|..|||.||..||||||.||....||||.|.|||||||.|.|.|..|..||...|.|....|.|.|..||.|||.||.|||.|||.|||.|.||||||.|...||.||.|||||

-VQLQQSGPELVKPGASVKLSCKASGYTFTSYDINWVKQRPGQGLEWIGWIYPRDGSTKYNEKFKGKATLTVDTSSSTAYMELHSLTSEDSAVYFCARERGLLLFPHYAMDYWGQGTSVTVSS

^^^^^^^^ ^^^^^^^^ ^^^^^^^^^^^^^^^^

Therapeutic : Benralizumab

Best Alignment of Benralizumab heavy chain to a sequence from OAS

EVQLVQSGAEVKKPGASVKVSCKASGYTFTSYVIHWVRQRPGQGLAWMGYINPYNDGTKYNERFKGKVTITSDRSTSTVYMELSSLRSEDTAVYLCGREGIRYYGLLGDYWGQGTLVTVSS

||||.|||.|..|||||||.|||||||||||||.|||.|.|||||.|.||||||||||||||.||||.|.|||.|.||.|||||||.|||.|||.|.||||.|||...|||||||.|||||

EVQLQQSGPELVKPGASVKMSCKASGYTFTSYVMHWVKQKPGQGLEWIGYINPYNDGTKYNEKFKGKATLTSDKSSSTAYMELSSLTSEDSAVYYCAREGIFYYGGAMDYWGQGTSVTVSS

^^^^^^^^ ^^^^^^^^ ^^^^^^^^^^^^^^

Best Alignment of Benralizumab light chain to a sequence from OAS

DIQMTQSPSSLSASVGDRVTITCGTSEDIINYLNWYQQKPGKAPKLLIYHTSRLQSGVPSRFSGSGSGTDFTLTISSLQPEDFATYYCQQGYT-LPYTFGQGTKVEIK

|||||||||||||||||||||||..|.||.|||||||||||||||||||..|.|||||||||||||||||||||||||||||||||||||.|...|||||||||||||

DIQMTQSPSSLSASVGDRVTITCQASQDISNYLNWYQQKPGKAPKLLIYAASSLQSGVPSRFSGSGSGTDFTLTISSLQPEDFATYYCQQFYSTPPYTFGQGTKVEIK

^^^^^^ ^^^ ^^^^^^^^^^

Best Alignment of Benralizumab heavy chain CDRs to a sequence from OAS

EVQLVQSGAEVKKPGASVKVSCKASGYTFTSYVIHWVRQRPGQGLAWMGYINPYNDGTKYNERFKGKVTITSDRSTSTVYMELSSLRSEDTAVYLCGREGIRYYGLLGDYWGQGTLVTVSS

||||.|||.|..|||||||.|||||||||||||.|||.|.|||||.|.||||||||||||||.||||.|.|||.|.||.|||||||.|||.|||.|.||||.|||...|||||||.|||||

EVQLQQSGPELVKPGASVKMSCKASGYTFTSYVMHWVKQKPGQGLEWIGYINPYNDGTKYNEKFKGKATLTSDKSSSTAYMELSSLTSEDSAVYYCAREGIFYYGGAMDYWGQGTSVTVSS

^^^^^^^^ ^^^^^^^^ ^^^^^^^^^^^^^^

Best Alignment of Benralizumab light chain CDRs to a sequence from OAS

DIQMTQSPSSLSASVGDRVTITCGTSEDIINYLNWYQQKPGKAPKLLIYHTSRLQSGVPSRFSGSGSGTDFTLTISSLQPEDFATYYCQQGYTLPYTFGQGTKVEIK

||||||..||||||.||||||.|..|.|||||||||||||....|||||.||||.|||||||||||||||..||||.|..||.|||.||||.|||||||.|||.|||

DIQMTQTTSSLSASLGDRVTISCRASQDIINYLNWYQQKPDGTVKLLIYYTSRLHSGVPSRFSGSGSGTDYSLTISNLEQEDIATYFCQQGNTLPYTFGGGTKLEIK

^^^^^^ ^^^ ^^^^^^^^^

Best Alignment of Benralizumab CDR-H3 to a sequence from OAS

EVQLVQSGAEVKKPGASVKVSCKASGYTFTSYVIHWVRQRPGQGLAWMGYINPYNDGTKYNERFKGKVTITSDRSTSTVYMELSSLRSEDTAVYLCGREGIRYYGLLGDYWGQGTLVTVSS

.|||.|.|||...||.|||.||||||||||||..|||.|||.|||.|.|.|.|....|.||..||.|.|.|.|.|.||.||.||||.|||.|||.|.||||.|||..||||||||..||||

-VQLQQPGAELVRPGSSVKLSCKASGYTFTSYWMHWVKQRPIQGLEWIGNIDPSDSETHYNQKFKDKATLTVDKSSSTAYMQLSSLTSEDSAVYYCAREGIYYYGSSGDYWGQGTTLTVSS

^^^^^^^^ ^^^^^^^^ ^^^^^^^^^^^^^^

Therapeutic : Berlimatoxumab

Best Alignment of Berlimatoxumab heavy chain to a sequence from OAS

ELQLQESGPGLVKPSETLSLTCTVSGGSISSGSYYWDWIRQPPGKGLEWIGNIYKSGSTYYNPSLKSRVTISVDTSKNQFSLKLSSVTAADTAVYYCARERGM----------HYMDVWGKGTTVTVSS

.||||||||||||||||||||||||||||||.||||.||||||||||||||.||.|||||||||||||||||||||||||||||||||||||||||||||||............|||||||||||||||

QLQLQESGPGLVKPSETLSLTCTVSGGSISSSSYYWGWIRQPPGKGLEWIGSIYYSGSTYYNPSLKSRVTISVDTSKNQFSLKLSSVTAADTAVYYCARERGYCSGGSCRYYYYYMDVWGKGTTVTVSS

^^^^^^^^^^ ^^^^^^^ ^^^^^^^^^^^^^^^^^^^^^

Best Alignment of Berlimatoxumab light chain to a sequence from OAS

DIQMTQSPSSLSASVGDRVTITCRASQSINSYLNWYQQKPGKAPKLLIYAASSLQSGVPSRFSGSGSGTDFTLTISSLQPEDFATYYCQQQFD-PPFTFGGGTKVEIK

|||||||||||||||||||||||||||||.||||||||||||||||||||||||||||||||||||||||||||||||||||||||||||....||||||||||||||

DIQMTQSPSSLSASVGDRVTITCRASQSISSYLNWYQQKPGKAPKLLIYAASSLQSGVPSRFSGSGSGTDFTLTISSLQPEDFATYYCQQSYSTPPFTFGGGTKVEIK

^^^^^^ ^^^ ^^^^^^^^^^

Best Alignment of Berlimatoxumab heavy chain CDRs to a sequence from OAS

ELQLQESGPGLVKPSETLSLTCTVSGGSISSGSYYWDWIRQPPGKGLEWIGNIYKSGSTYYNPSLKSRVTISVDTSKNQFSLKLSSVTAADTAVYYCARERGMHYMDVWGKGTTVTVSS

.............|||||||||||||||||||||||.||||||||||||||.||.|||||||||||||||||||||||||||||||||||||||||||||||..||||||||||||||.

-------------PSETLSLTCTVSGGSISSGSYYWSWIRQPPGKGLEWIGYIYYSGSTYYNPSLKSRVTISVDTSKNQFSLKLSSVTAADTAVYYCARERGGYYMDVWGKGTTVTVS-

^^^^^^^^^^ ^^^^^^^ ^^^^^^^^^^^

Best Alignment of Berlimatoxumab light chain CDRs to a sequence from OAS

DIQMTQSPSSLSASVGDRVTITCRASQSINSYLNWYQQKPGKAPKLLIYAASSLQSGVPSRFSGSGSGTDFTLTISSLQPEDFATYYCQQQFDPPFTFGGGTKVEIK

|||||.||||||||||||||||||||||||||||||||||||||..||||||||.||||||||.|||||||||.||||||||.|||.|||..|.|||||.||.|..|

DIQMTPSPSSLSASVGDRVTITCRASQSINSYLNWYQQKPGKAPNVLIYAASSLESGVPSRFSASGSGTDFTLIISSLQPEDSATYFCQQSDDVPFTFGPGTAVDVK

^^^^^^ ^^^ ^^^^^^^^^

Best Alignment of Berlimatoxumab CDR-H3 to a sequence from OAS

ELQLQESGPGLVKPSETLSLTCTVSGGSISSGSYYWDWIRQPPGKGLEWIGNIYKS-GSTYYNPSLKSRVTISVDTSKNQFSLKLSSVTAADTAVYYCARERGMHYMDVWGKGTTVTVSS

.............|...|.|.|..||......||...|.||.|||||||...|..|.|||||..|.|.|.|||.|.|||...|...|..|.||||||||||||.|||||||||||||||.

-------------PRGSLRLSCAASGFTF--SSYAMSWVRQAPGKGLEWVSAISGSGGSTYYADSVKGRFTISRDNSKNTLYLQMNSLRAEDTAVYYCARERGEHYMDVWGKGTTVTVS-

^^^^^^^^^^ ^^^^^^^^ ^^^^^^^^^^^

Therapeutic : Bevacizumab

Best Alignment of Bevacizumab heavy chain to a sequence from OAS

EVQLVESGGGLVQPGGSLRLSCAASGYTFTNYGMNWVRQAPGKGLEWVGWINTYTGEPTYAADFKRRFTFSLDTSKSTAYLQMNSLRAEDTAVYYCAKYPHYYGSSHWYFDVWGQGTLVTVSS

.|||||||||||||||||||||||||||||.|.|||||||||.||||.|||||.||.||||..|..||.||||||.||||||..||.||||||||||..|.........||.|||||||||||

-VQLVESGGGLVQPGGSLRLSCAASGYTFTSYAMNWVRQAPGQGLEWMGWINTNTGNPTYAQGFTGRFVFSLDTSVSTAYLQISSLKAEDTAVYYCARDPTITI-FEYWFDPWGQGTLVTVSS

^^^^^^^^ ^^^^^^^^ ^^^^^^^^^^^^^^^^

Best Alignment of Bevacizumab light chain to a sequence from OAS

DIQMTQSPSSLSASVGDRVTITCSASQDISNYLNWYQQKPGKAPKVLIYFTSSLHSGVPSRFSGSGSGTDFTLTISSLQPEDFATYYCQQYSTVPWTFGQGTKVEIK

|||||||||||||||||||||||.|||||||||||||||||||||||||.||.|.|||||||||||||||||||||||||||||||||||.||.|.|||||||||||

DIQMTQSPSSLSASVGDRVTITCQASQDISNYLNWYQQKPGKAPKVLIYGTSILQSGVPSRFSGSGSGTDFTLTISSLQPEDFATYYCQQSSTTPRTFGQGTKVEIK

^^^^^^ ^^^ ^^^^^^^^^

Best Alignment of Bevacizumab heavy chain CDRs to a sequence from OAS

EVQLVESGGGLVQPGGSLRLSCAASGYTFTNYGMNWVRQAPGKGLEWVGWINTYTGEPTYAADFKRRFTFSLDTSKSTAYLQMNSLRAEDTAVYYCAKYPHYYGSSHWYFDVWGQGTLVTVSS

..|||.||..|..||.....||.||||||||||||||.|||||||.|.|||||||||||||.|||.||.|||.||.||||||.|.|..||.|.|.||.||.|||||.|||||||.||.|||||

QIQLVQSGPELKKPGETVKISCKASGYTFTNYGMNWVKQAPGKGLKWMGWINTYTGEPTYADDFKGRFAFSLETSASTAYLQINNLKNEDMATYFCARYPFYYGSSYWYFDVWGAGTTVTVSS

^^^^^^^^ ^^^^^^^^ ^^^^^^^^^^^^^^^^

Best Alignment of Bevacizumab light chain CDRs to a sequence from OAS

DIQMTQSPSSLSASVGDRVTITCSASQDISNYLNWYQQKPGKAPKVLIYFTSSLHSGVPSRFSGSGSGTDFTLTISSLQPEDFATYYCQQYSTVPWTFGQGTKVEIK

||||||..||||||.||||||.||||||||||||||||||....|.||||||||||||||||||||||||..||||.|.|||.|||||||||..|||||.|||.|||

DIQMTQTTSSLSASLGDRVTISCSASQDISNYLNWYQQKPDGTVKLLIYFTSSLHSGVPSRFSGSGSGTDYFLTISNLEPEDIATYYCQQYSKLPWTFGGGTKLEIK

^^^^^^ ^^^ ^^^^^^^^^

Best Alignment of Bevacizumab CDR-H3 to a sequence from OAS

EVQLVESGGGLVQPGGSLRLSCAASGYTFTNYGMNWVRQAPGKGLEWVGWINTYTGEPTYAADFKRRFTFSLDTSKSTAYLQMNSLRAEDTAVYYCAKYPHYYGSSHWYFDVWGQGTLVTVSS

.|||..||..||.||.|...||.||||.|..|.||||.|.|||||||.|.|....|...|...||...|...|.|.||||.|..||..||.|||.||.||||||||||||||||.||.|||||

QVQLQQSGAELVKPGASVKISCKASGYAFSSYWMNWVKQRPGKGLEWIGQIYPGDGDTNYNGKFKGKATLTADKSSSTAYMQLSSLTSEDSAVYFCARYPHYYGSSHWYFDVWGTGTTVTVSS

^^^^^^^^ ^^^^^^^^ ^^^^^^^^^^^^^^^^

Therapeutic : Bezlotoxumab

Best Alignment of Bezlotoxumab heavy chain to a sequence from OAS

EVQLVQSGAEVKKSGESLKISCKGSGYSFTSYWIGWVRQMPGKGLEWMGIFYPGDSSTRYSPSFQGQVTISADKSVNTAYLQWSSLKASDTAMYYCARRRNW---GNAFDIWGQGTMVTVSS

|||||||||||||.||||||||||||||||||||||||||||||||||||.|||||.||||||||||||||||||..|||||||||||||||||||||||||.....|||||||||||||||

EVQLVQSGAEVKKPGESLKISCKGSGYSFTSYWIGWVRQMPGKGLEWMGIIYPGDSDTRYSPSFQGQVTISADKSISTAYLQWSSLKASDTAMYYCARRRNWNYVRGAFDIWGQGTMVTVSS

^^^^^^^^ ^^^^^^^^ ^^^^^^^^^^^^^^^

Best Alignment of Bezlotoxumab light chain to a sequence from OAS

EIVLTQSPGTLSLSPGERATLSCRASQSVSSSYLAWYQQKPGQAPRLLIYGASSRATGIPDRFSGSGSGTDFTLTISRLEPEDFAVYYCQQYGSSTWTFGQGTKVEIK

||||||||||||||||||||||||||||||||||||||||||||||||||||||||||||||||||||||||||||||||||||||||||||||||||||||||||||

EIVLTQSPGTLSLSPGERATLSCRASQSVSSSYLAWYQQKPGQAPRLLIYGASSRATGIPDRFSGSGSGTDFTLTISRLEPEDFAVYYCQQYGSSTWTFGQGTKVEIK

^^^^^^^ ^^^ ^^^^^^^^^

Best Alignment of Bezlotoxumab heavy chain CDRs to a sequence from OAS

EVQLVQSGAEVKKSGESLKISCKGSGYSFTSYWIGWVRQMPGKGLEWMGIFYPGDSSTRYSPSFQGQVTISADKSVNTAYLQWSSLKASDTAMYYCARRRNWGNAFDIWGQGTMVTVSS

..............||||||||||||||||||||||||||||||||||||.|||||.||||||||||||||||||..||||||||||||||||||||||||.|||||||||||||||||

--------------GESLKISCKGSGYSFTSYWIGWVRQMPGKGLEWMGIIYPGDSDTRYSPSFQGQVTISADKSISTAYLQWSSLKASDTAMYYCARRRNYGNAFDIWGQGTMVTVSS

^^^^^^^^ ^^^^^^^^ ^^^^^^^^^^^^

Best Alignment of Bezlotoxumab light chain CDRs to a sequence from OAS

EIVLTQSPGTLSLSPGERATLSCRASQSVSSSYLAWYQQKPGQAPRLLIYGASSRATGIPDRFSGSGSGTDFTLTISRLEPEDFAVYYCQQYGSSTWTFGQGTKVEIK

||||||||||||||||||||||||||||||||||||||||||||||||||||||||||||||||||||||||||||||||||||||||||||||||||||||||||||

EIVLTQSPGTLSLSPGERATLSCRASQSVSSSYLAWYQQKPGQAPRLLIYGASSRATGIPDRFSGSGSGTDFTLTISRLEPEDFAVYYCQQYGSSTWTFGQGTKVEIK

^^^^^^^ ^^^ ^^^^^^^^^

Best Alignment of Bezlotoxumab CDR-H3 to a sequence from OAS

EVQLVQSGAEVKKSGESLKISCKGSGYSFTSYWIGWVRQMPGKGLEWMGIFYPGDSSTRYSPSFQGQVTISADKSVNTAYLQWSSLKASDTAMYYCARRRNWGNAFDIWGQGTMVTVSS

...............|.|...|..||.||......|.||.|||||||.|.......||.|.||....||||.|.|.|...|..||..|.|||.||||||||||.|||||||||||||||

--------------SETLSLTCAVSGGSFSGFYWSWIRQPPGKGLEWIGEINHS-GSTNYNPSLKSRVTISVDTSKNQFSLKLSSVTAADTAVYYCARRRNWGRAFDIWGQGTMVTVSS

^^^^^^^^ ^^^^^^^^ ^^^^^^^^^^^^

Therapeutic : Bimagrumab

Best Alignment of Bimagrumab heavy chain to a sequence from OAS

QVQLVQSGAEVKKPGASVKVSCKASGYTFTSSYINWVRQAPGQGLEWMGTINPVSGSTSYAQKFQGRVTMTRDTSISTAYMELSRLRSDDTAVYYCARGG----------WFDYWGQGTLVTVSS

|||||||||||||||||||||||||||||||..||||||||||||||||.|||.||.|.|||||||||||||||||||||||||||||||||||||||||............|||||||||||||

QVQLVQSGAEVKKPGASVKVSCKASGYTFTSYDINWVRQAPGQGLEWMGWINPNSGGTNYAQKFQGRVTMTRDTSISTAYMELSRLRSDDTAVYYCARGGEYYDFWSGYYPTDYWGQGTLVTVSS

^^^^^^^^ ^^^^^^^^ ^^^^^^^^^^^^^^^^^^

Best Alignment of Bimagrumab light chain to a sequence from OAS

QSALTQPASVSGSPGQSITISCTGTSSDVGSYNYVNWYQQHPGKAPKLMIYGVSKRPSGVSNRFSGSKSGNTASLTISGLQAEDEADYYCGTFAGGSYYGVFGGGTKLTVL

||||||||||||||||||||||||||||||.||||.|||||||||||||||.||||||||||||||||||||||||||||||||||||||...||.|.|||||||||||||

QSALTQPASVSGSPGQSITISCTGTSSDVGGYNYVSWYQQHPGKAPKLMIYDVSKRPSGVSNRFSGSKSGNTASLTISGLQAEDEADYYCCSYAGSSTYGVFGGGTKLTVL

^^^^^^^^^ ^^^ ^^^^^^^^^^^

Best Alignment of Bimagrumab heavy chain CDRs to a sequence from OAS

QVQLVQSGAEVKKPGASVKVSCKASGYTFTSSYINWVRQAPGQGLEWMGTINPVSGSTSYAQKFQGRVTMTRDTSISTAYMELSRLRSDDTAVYYCARGGWFDYWGQGTLVTVSS

..............|||||||||||||||||.|..||||||||||||||.|||.|||||||||||||||||||||.||.|||||.|||.||||||||||||||.|||||||||||

--------------GASVKVSCKASGYTFTSYYMHWVRQAPGQGLEWMGIINPSSGSTSYAQKFQGRVTMTRDTSTSTVYMELSSLRSEDTAVYYCARGGWFDPWGQGTLVTVSS

^^^^^^^^ ^^^^^^^^ ^^^^^^^^

Best Alignment of Bimagrumab light chain CDRs to a sequence from OAS

QSALTQPASVSGSPGQSITISCTGTSSDVGSYNYVNWYQQHPGKAPKLMIYGVSKRPSGVSNRFSGSKSGNTASLTISGLQAEDEADYYCGTFAGGSYYGVFGGGTKLTVL

||||||||||||.||.|||..||||||||||||...||||||||||.|||||||||||||.|||||||||||||.||.|||||||||||||..||.|.|.|||||.|||||

QSALTQPASVSGAPGPSITNPCTGTSSDVGSYNLFSWYQQHPGKAPNLMIYGVSKRPSGVANRFSGSKSGNTASVTIAGLQAEDEADYYCGSEAGISTYWVFGGGIKLTVL

^^^^^^^^^ ^^^ ^^^^^^^^^^^

Best Alignment of Bimagrumab CDR-H3 to a sequence from OAS

QVQLVQSGAEVKKPGASVKVSCKASGYTFTSSYINWVRQAPGQGLEWMGTINPVSGSTSYAQKFQGRVTMTRDTSISTAYMELSRLRSDDTAVYYCARGGWFDYWGQGTLVTVSS

.|.||.|......||.|.|.||.|||.||...|..||||.|..||||...||....||.|......|....||......|...|.|.|.|||.||||||||||||||||..||||

EVKLVESEGGLVQPGSSMKLSCTASGFTFSDYYMAWVRQVPEKGLEWVANINYDGSSTYYLDSLKSRFIISRDNAKNILYLQMSSLKSEDTATYYCARGGWFDYWGQGTTLTVSS

^^^^^^^^ ^^^^^^^^ ^^^^^^^^

Therapeutic : Bimekizumab

Best Alignment of Bimekizumab heavy chain to a sequence from OAS

EVQLVESGGGLVQPGGSLRLSCAASGFTFSDYNMAWVRQAPGKGLEWVATITYEGRNTYYRDSVKGRFTISRDNAKNSLYLQMNSLRAEDTAVYYCASPPQYYEGSIYRLWFAHWGQGTLVTVSS

|||||||||||||||.||.||||||||||||||||||||||.|||||||||.|.|..|||||||||||||||||||..|||||||||.||||.|||||.|.||.||.|..|||.|||||||||||

EVQLVESGGGLVQPGRSLKLSCAASGFTFSDYNMAWVRQAPKKGLEWVATISYDGSSTYYRDSVKGRFTISRDNAKSTLYLQMNSLRSEDTATYYCASSPYYYDGSYYYGWFAYWGQGTLVTVSS

^^^^^^^^ ^^^^^^^^ ^^^^^^^^^^^^^^^^^^

Best Alignment of Bimekizumab light chain to a sequence from OAS

AIQLTQSPSSLSASVGDRVTITCRADESVRTLMHWYQQKPGKAPKLLIYLVSNSEIGVPDRFSGSGSGTDFRLTISSLQPEDFATYYCQQTWSDPWTFGQGTKVEIK

|||||||||||||||||||||||||.........|||||||||||||||..||.|.|||.|||||||||||.||||||||||||||||||..|||||||||||||||

AIQLTQSPSSLSASVGDRVTITCRASQGISSALAWYQQKPGKAPKLLIYDASNLESGVPSRFSGSGSGTDFTLTISSLQPEDFATYYCQQFNSDPWTFGQGTKVEIK

^^^^^^ ^^^ ^^^^^^^^^

Best Alignment of Bimekizumab heavy chain CDRs to a sequence from OAS

EVQLVESGGGLVQPGGSLRLSCAASGFTFSDYNMAWVRQAPGKGLEWVATITYEGRNTYYRDSVKGRFTISRDNAKNSLYLQMNSLRAEDTAVYYCASPPQYYEGSIYRLWFAHWGQGTLVTVSS

|||||||||||||||.||.|||||||||||||.||||||||.|||||||.|.|||..|||.|||||||||||||||..|||||||||.||||.||||.||.||.||.|..|||.|||||||||||

EVQLVESGGGLVQPGRSLKLSCAASGFTFSDYYMAWVRQAPKKGLEWVASISYEGSSTYYGDSVKGRFTISRDNAKSTLYLQMNSLRSEDTATYYCARPPYYYDGSYYANWFAYWGQGTLVTVSS

^^^^^^^^ ^^^^^^^^ ^^^^^^^^^^^^^^^^^^

Best Alignment of Bimekizumab light chain CDRs to a sequence from OAS

AIQLTQSPSSLSASVGDRVTITCRADESVRTLMHWYQQKPGKAPKLLIYLVSNSEIGVPDRFSGSGSGTDFRLTISSLQPEDFATYYCQQTWSDPWTFGQGTKVEIK

.|.|||||...|...|..|||||||..||.|..|||||.|...|||||..||.|..|||.|||||||||...|||.||..||.||||||||.|.|||||||||||||

EIALTQSPDFQSVTPGEKVTITCRASQSVGTSLHWYQQQPEQSPKLLIKYVSQSFSGVPSRFSGSGSGTTYTLTIKSLEAEDGATYYCQQTSSLPWTFGQGTKVEIK

^^^^^^ ^^^ ^^^^^^^^^

Best Alignment of Bimekizumab CDR-H3 to a sequence from OAS

EVQLVESGGGLVQPGGSLRLSCAASGFTFSDYNMAWVRQAPGKGLEWVATITYEGRNTYYRDSVKGRFTISRDNAKNSLYLQMNSLRAEDTAVYYCASPPQYYEGSIYRLWFAHWGQGTLVTVSS

.|||...|..||.||.|..|||.|||.||..|.|.||.|.||.||||...|.............|...|..........|.|..||..||.|||||||||.||.||.|..|||.||||||||||.

QVQLQQPGAELVKPGASVKLSCKASGYTFTSYWMHWVKQRPGQGLEWIGMIHPNSGRINHNEKFKSKATLTVVKSSSTAYMQLSSLTSEDSAVYYCASPPIYYYGSSYLSWFAYWGQGTLVTVSA

^^^^^^^^ ^^^^^^^^ ^^^^^^^^^^^^^^^^^^

Therapeutic : Bleselumab

Best Alignment of Bleselumab heavy chain to a sequence from OAS

QLQLQESGPGLLKPSETLSLTCTVSGGSISSPGYYGGWIRQPPGKGLEWIGSIYKSGSTYHNPSLKSRVTISVDTSKNQFSLKLSSVTAADTAVYYCTRPVVRY-----FGWFDPWGQGTLVTVSS

|||||||||||.|||||||||||||||||||..||.||||||||||||||||||.|||||.||||||||||||||||||||||||||||||||||||.|||.||......||||||||||||||||

QLQLQESGPGLVKPSETLSLTCTVSGGSISSSSYYWGWIRQPPGKGLEWIGSIYYSGSTYYNPSLKSRVTISVDTSKNQFSLKLSSVTAADTAVYYCARPVIRYSYGSLRGWFDPWGQGTLVTVSS

^^^^^^^^^^ ^^^^^^^ ^^^^^^^^^^^^^^^^^^

Best Alignment of Bleselumab light chain to a sequence from OAS

AIQLTQSPSSLSASVGDRVTITCRASQGISSALAWYQQKPGKAPKLLIYDASNLESGVPSRFSGSGSGTDFTLTISSLQPEDFATYYCQQFNSYPTFGQGTKVEIK

||||||||||||||||||||||||||||||||||||||||||||||||||||.|||||||||||||||||||||||||||||||||||||||.|||||||||||||

AIQLTQSPSSLSASVGDRVTITCRASQGISSALAWYQQKPGKAPKLLIYDASSLESGVPSRFSGSGSGTDFTLTISSLQPEDFATYYCQQFNNYPTFGQGTKVEIK

^^^^^^ ^^^ ^^^^^^^^

Best Alignment of Bleselumab heavy chain CDRs to a sequence from OAS

QLQLQESGPGLLKPSETLSLTCTVSGGSISSPGYYGGWIRQPPGKGLEWIGSIYKSGSTYHNPSLKSRVTISVDTSKNQFSLKLSSVTAADTAVYYCTRPVVRYFGWFDPWGQGTLVTVSS

.........||.|||.|||||||||||||||.|||..||||.|||||||||.||.|||||.||||||.|||||||||||||||||||||||||||||.||||...||||||||||||||||

---------GLVKPSQTLSLTCTVSGGSISSGGYYWSWIRQHPGKGLEWIGYIYYSGSTYYNPSLKSLVTISVDTSKNQFSLKLSSVTAADTAVYYCARPVVTPSGWFDPWGQGTLVTVSS

^^^^^^^^^^ ^^^^^^^ ^^^^^^^^^^^^^

Best Alignment of Bleselumab light chain CDRs to a sequence from OAS

AIQLTQSPSSLSASVGDRVTITCRASQGISSALAWYQQKPGKAPKLLIYDASNLESGVPSRFSGSGSGTDFTLTISSLQPEDFATYYCQQFNSYPTFGQGTKVEIK

|||||||||||||||||||||||||||||||||.||||||||||||||||||.||||||||||||||||||||||||||||||||||||||||||||||||..|||

AIQLTQSPSSLSASVGDRVTITCRASQGISSALVWYQQKPGKAPKLLIYDASSLESGVPSRFSGSGSGTDFTLTISSLQPEDFATYYCQQFNSYPTFGQGTRLEIK

^^^^^^ ^^^ ^^^^^^^^

Best Alignment of Bleselumab CDR-H3 to a sequence from OAS

QLQLQESGPGLLKPSETLSLTCTVSGGSISSPGYYGGWIRQPPGKGLEWIGSIYKSGSTYHNPSLKSRVTISVDTSKNQFSLKLSSVTAADTAVYYCTRPVVRYFGWFDPWGQGTLVTVSS

..............|||||||||||||||....||..||||||||||||||.||.||||..|||||||||||||||||||||||||||||||||.||.||||||.||||||||||||||||

--------------SETLSLTCTVSGGSI--SSYYWSWIRQPPGKGLEWIGYIYYSGSTNYNPSLKSRVTISVDTSKNQFSLKLSSVTAADTAVHYCARPVVRYMGWFDPWGQGTLVTVSS

^^^^^^^^^^ ^^^^^^^ ^^^^^^^^^^^^^

Therapeutic : Blosozumab

Best Alignment of Blosozumab heavy chain to a sequence from OAS

QVQLVQSGAEVKKPGASVKVSCKVSGFPIKDTFQHWVRQAPGKGLEWMGWSDPEIGDTEYASKFQGRVTMTEDTSTDTAYMELSSLRSEDTAVYYCATGDTT------------YKFDFWGQGTTVTVSS

||||||||||||||||||||||||||........|||||||||||||||..|||.|.|.||.||||||||||||||||||||||||||||||||||||||..............|..|||||||||||||

QVQLVQSGAEVKKPGASVKVSCKVSGYTLTELSMHWVRQAPGKGLEWMGGFDPEDGETIYAQKFQGRVTMTEDTSTDTAYMELSSLRSEDTAVYYCATGDPLAVAVAPTNYYYYYGMDFWGQGTTVTVSS

^^^^^^^^ ^^^^^^^^ ^^^^^^^^^^^^^^^^^^^^^^^

Best Alignment of Blosozumab light chain to a sequence from OAS

DIQMTQSPSSLSASVGDRVTITCKASQDVHTAVAWYQQKPGKAPKLLIYWASTRWTGVPSRFSGSGSGTDFTLTISSLQPEDFATYYCQQYSDYPWTFGGGTKVEIK

|||||||||.|||||||||||||.|||.|.|..||||||||||||||||.|||..||||||||||||||||||||||||||||||||||||..||.|||||||||||

DIQMTQSPSTLSASVGDRVTITCRASQTVNTWLAWYQQKPGKAPKLLIYKASTLETGVPSRFSGSGSGTDFTLTISSLQPEDFATYYCQQYNSYPLTFGGGTKVEIK

^^^^^^ ^^^ ^^^^^^^^^

Best Alignment of Blosozumab heavy chain CDRs to a sequence from OAS

QVQLVQSGAEVKKPGASVKVSCKVSGFPIKDTFQHWVRQAPGKGLEWMGWSDPEIGDTEYASKFQGRVTMTEDTSTDTAYMELSSLRSEDTAVYYCATGDTTYKFDFWGQGTTVTVSS

.|||.|||||...||||||.||..|||.|||...|||.|....||||.|..|||.|||.||.||||..|.|.||...||...||||.||||||||||.||||..||.||.||||||||

EVQLQQSGAELVTPGASVKLSCTASGFNIKDYYMHWVKQRTEQGLEWIGRIDPEDGDTKYAPKFQGKATLTADTASNTADLQLSSLTSEDTAVYYCARGDTTWYFDVWGTGTTVTVSS

^^^^^^^^ ^^^^^^^^ ^^^^^^^^^^^

Best Alignment of Blosozumab light chain CDRs to a sequence from OAS

DIQMTQSPSSLSASVGDRVTITCKASQDVHTAVAWYQQKPGKAPKLLIYWASTRWTGVPSRFSGSGSGTDFTLTISSLQPEDFATYYCQQYSDYPWTFGGGTKVEIK

||.||||....|.||||||.|||||||||.|||||||||||..||||||||||..||||.||.|||||||||||||..|.||.|.|.|||||.||||||||||.|||

DIVMTQSHKFMSTSVGDRVSITCKASQDVGTAVAWYQQKPGQSPKLLIYWASTLHTGVPDRFTGSGSGTDFTLTISNVQSEDLADYFCQQYSSYPWTFGGGTKLEIK

^^^^^^ ^^^ ^^^^^^^^^

Best Alignment of Blosozumab CDR-H3 to a sequence from OAS

QVQLVQSGAEVKKPGASVKVSCKVSGFPIKDTFQHWVRQAPGKGLEWMGWSDPEIGDTEYASKFQGRVTMTEDTSTDTAYMELSSLRSEDTAVYYCATGDTTYKFDFWGQGTTVTVSS

||||.|||||..|||||||.|||.||.........||.|.|||||||.|...|..|||.|...|.|..|.|.|.|..||||.||||.|||.|||.||.|||||.|||||||||.||||

QVQLQQSGAELVKPGASVKISCKASGYAFSNYWMNWVKQRPGKGLEWIGQIYPGDGDTNYNGNFKGKATLTADKSSSTAYMQLSSLTSEDSAVYFCARGDTTYYFDFWGQGTTLTVSS

^^^^^^^^ ^^^^^^^^ ^^^^^^^^^^^

Therapeutic : Bococizumab

Best Alignment of Bococizumab heavy chain to a sequence from OAS

QVQLVQSGAEVKKPGASVKVSCKASGYTFTSYYMHWVRQAPGQGLEWMGEISPFGGRTNYNEKFKSRVTMTRDTSTSTVYMELSSLRSEDTAVYYCARERPL-------YASDLWGQGTTVTVSS

|||||||||||||||||||||||||||||||||||||||||||||||||.|||.||.|.|..||..||||||||||||||||||||||||||||||||.|.........|..|.|||||||||||

QVQLVQSGAEVKKPGASVKVSCKASGYTFTSYYMHWVRQAPGQGLEWMGIISPSGGSTSYAQKFQGRVTMTRDTSTSTVYMELSSLRSEDTAVYYCARDRRVTSLYYYYYGMDVWGQGTTVTVSS

^^^^^^^^ ^^^^^^^^ ^^^^^^^^^^^^^^^^^^

Best Alignment of Bococizumab light chain to a sequence from OAS

DIQMTQSPSSLSASVGDRVTITCRASQGISSALAWYQQKPGKAPKLLIYSASYRYTGVPSRFSGSGSGTDFTFTISSLQPEDIATYYCQQRYSLWRTFGQGTKLEIK

|||||||||||||||||||||||||||.|||.|.||||||||||||||||||....||||||||||||||||.|||||||||||||||||..||.||||||||||||

DIQMTQSPSSLSASVGDRVTITCRASQSISSYLNWYQQKPGKAPKLLIYSASSLQSGVPSRFSGSGSGTDFTLTISSLQPEDIATYYCQQCDSLPRTFGQGTKLEIK

^^^^^^ ^^^ ^^^^^^^^^

Best Alignment of Bococizumab heavy chain CDRs to a sequence from OAS

QVQLVQSGAEVKKPGASVKVSCKASGYTFTSYYMHWVRQAPGQGLEWMGEISPFGGRTNYNEKFKSRVTMTRDTSTSTVYMELSSLRSEDTAVYYCARERPLYASDLWGQGTTVTVSS

................|||||||||||||||||||||||||||||||||.|.|.||.|.|..||..|||||||||||||||||||||||||||||||||||.||.|.|||||.||..|

----------------SVKVSCKASGYTFTSYYMHWVRQAPGQGLEWMGIINPSGGSTSYAQKFQDRVTMTRDTSTSTVYMELSSLRSEDTAVYYCARERPGYACDIWGQGTMVTAPS

^^^^^^^^ ^^^^^^^^ ^^^^^^^^^^^

Best Alignment of Bococizumab light chain CDRs to a sequence from OAS

DIQMTQSPSSLSASVGDRVTITCRASQGISSALAWYQQKPGKAPKLLIYSASYRYTGVPSRFSGSGSGTDFTFTISSLQPEDIATYYCQQRYSLWRTFGQGTKLEIK

|||.||||||||||||||||||||.||||||.|.||.|||||.|||||||||....||||||||||||||||.|||||||||.|||||||.|||.||||||||.|||

DIQLTQSPSSLSASVGDRVTITCRVSQGISSYLNWYRQKPGKVPKLLIYSASNLQSGVPSRFSGSGSGTDFTLTISSLQPEDFATYYCQQSYSLPRTFGQGTKVEIK

^^^^^^ ^^^ ^^^^^^^^^

Best Alignment of Bococizumab CDR-H3 to a sequence from OAS

QVQLVQSGAEVKKPGASVKVSCKASGYTFTSYYMHWVRQAPGQGLEWMGEISPFGGRTNYNEKFKSRVTMTRDTSTSTVYMELSSLRSEDTAVYYCARERPLYASDLWGQGTTVTVSS

.|||.|||.|..|||||||.|||.||||||.|.||||.|..|..|||.|.|.|..|.|.||.|||...|.|.|.|.||.||||.||.|||.|||||||||||||.|.|||||.|||||

EVQLQQSGPELVKPGASVKISCKTSGYTFTEYTMHWVKQSHGKSLEWIGGINPNNGGTSYNQKFKGKATLTVDKSSSTAYMELRSLTSEDSAVYYCARERPLYAMDYWGQGTSVTVSS

^^^^^^^^ ^^^^^^^^ ^^^^^^^^^^^

Therapeutic : Brazikumab

Best Alignment of Brazikumab heavy chain to a sequence from OAS

QVQLVESGGGVVQPGRSLRLSCAASGFTFSSYGMHWVRQAPGKGLEWVAVIWYDGSNEYYADSVKGRFTISRDNSKNTLYLQMNSLRAEDTAVYYCARDRGYTSS--WYPDAFDIWGQGTMVTVSS

|||||||||||||||||||||||||||||||||||||||||||||||||||||||||.||||||||||||||||||||||||||||||||||||||||||||.||...||||||||||||||||||

QVQLVESGGGVVQPGRSLRLSCAASGFTFSSYGMHWVRQAPGKGLEWVAVIWYDGSNKYYADSVKGRFTISRDNSKNTLYLQMNSLRAEDTAVYYCARDRGYCSSTSCYPDAFDIWGQGTMVTVSS

^^^^^^^^ ^^^^^^^^ ^^^^^^^^^^^^^^^^^^^

Best Alignment of Brazikumab light chain to a sequence from OAS

QSVLTQPPSVSGAPGQRVTISCTGSSSNTGAGYDVHWYQQVPGTAPKLLIYGSGNRPSGVPDRFSGSKSGTSASLAITGLQAEDEADYYCQSYDSSLSGWVFGGGTRLTVL

||||||||||||||||||||||||||||||||||||||||||||||||||||..|||||||||||||||||||||||||||||||||||||||||||||.||||||.||||

QSVLTQPPSVSGAPGQRVTISCTGSSSNTGAGYDVHWYQQVPGTAPKLLIYGNSNRPSGVPDRFSGSKSGTSASLAITGLQAEDEADYYCQSYDSSLSGVVFGGGTKLTVL

^^^^^^^^^ ^^^ ^^^^^^^^^^^

Best Alignment of Brazikumab heavy chain CDRs to a sequence from OAS

QVQLVESGGGVVQPGRSLRLSCAASGFTFSSYGMHWVRQAPGKGLEWVAVIWYDGSNEYYADSVKGRFTISRDNSKNTLYLQMNSLRAEDTAVYYCARDRGYTSSWYPDAFDIWGQGTMVTVSS

................|||||||||||||||||||||||||||||||||.|||||||..|||||||||||||||||||||||||||||||||.||.||||||.||||.||||||||||||||||

---------------GSLRLSCAASGFTFSSYGMHWVRQAPGKGLEWVAFIWYDGSNKFYADSVKGRFTISRDNSKNTLYLQMNSLRAEDTAMYYSARDRGYSSSWYDDAFDIWGQGTMVTVSS

^^^^^^^^ ^^^^^^^^ ^^^^^^^^^^^^^^^^^

Best Alignment of Brazikumab light chain CDRs to a sequence from OAS

QSVLTQPPSVSGAPGQRVTISCTGSSSNTGAGYDVHWYQQVPGTAPKLLIYGSGNRPSGVPDRFSGSKSGTSASLAITGLQAEDEADYYCQSYDSSLSGWVFGGGTRLTVL

||||||||||||||||||||||||||||||||||||||||.||||||||||||.|||.||||||||||||||||||||||||||||||||||||||||||||||||.||||

QSVLTQPPSVSGAPGQRVTISCTGSSSNTGAGYDVHWYQQLPGTAPKLLIYGSINRPPGVPDRFSGSKSGTSASLAITGLQAEDEADYYCQSYDSSLSGWVFGGGTKLTVL

^^^^^^^^^ ^^^ ^^^^^^^^^^^

Best Alignment of Brazikumab CDR-H3 to a sequence from OAS

QVQLVESGGGVVQPGRSLRLSCAASGFTF--SSYGMHWVRQAPGKGLEWVAVIWYDGSNEYYADSVKGRFTISRDNSKNTLYLQMNSLRAEDTAVYYCARDRGYTSSWYPDAFDIWGQGTMVTVSS

.|||.|||.|.|.|...|.|.|..||.......|...|.||.|||||||...|.|.....||..|.|.|.|||.|.|||...|...|..|.|||||||||||||.|||||||||||||||.|||||

-VQLQESGPGLVKPSQTLSLTCTVSGGSISSGDYYWSWIRQPPGKGLEWIGYIYYS-GSTYYNPSLKSRVTISVDTSKNQFSLKLSSVTAADTAVYYCARDRGYSSSWYPDAFDIWGQGTTVTVSS

^^^^^^^^^^ ^^^^^^^^ ^^^^^^^^^^^^^^^^^

Therapeutic : Brentuximab

Best Alignment of Brentuximab heavy chain to a sequence from OAS

QIQLQQSGPEVVKPGASVKISCKASGYTFTDYYITWVKQKPGQGLEWIGWIYPGSGNTKYNEKFKGKATLTVDTSSSTAFMQLSSLTSEDTAVYFCANYGN-YWFAYWGQGTQVTVSA

||||||||||.|||||||||||||||||||||||.||||||||||||||||||||||||||||||||||||||||||||.|||||||||||||||||.|||...||||||||.|||||

QIQLQQSGPELVKPGASVKISCKASGYTFTDYYINWVKQKPGQGLEWIGWIYPGSGNTKYNEKFKGKATLTVDTSSSTAYMQLSSLTSEDTAVYFCASYGNYVGFAYWGQGTLVTVSA

^^^^^^^^ ^^^^^^^^ ^^^^^^^^^^^

Best Alignment of Brentuximab light chain to a sequence from OAS

DIVLTQSPASLAVSLGQRATISCKASQSVDFDGDSYMNWYQQKPGQPPKVLIYAASNLESGIPARFSGSGSGTDFTLNIHPVEEEDAATYYCQQSNEDPWTFGGGTKLEIK

|||||||||||||||||||||||||||||||||||||||||||||||||.|||||||||||||||||||||||||||||||||||||||||||||||||.|||||||||||

DIVLTQSPASLAVSLGQRATISCKASQSVDFDGDSYMNWYQQKPGQPPKLLIYAASNLESGIPARFSGSGSGTDFTLNIHPVEEEDAATYYCQQSNEDPRTFGGGTKLEIK

^^^^^^^^^^ ^^^ ^^^^^^^^^

Best Alignment of Brentuximab heavy chain CDRs to a sequence from OAS

QIQLQQSGPEVVKPGASVKISCKASGYTFTDYYITWVKQKPGQGLEWIGWIYPGSGNTKYNEKFKGKATLTVDTSSSTAFMQLSSLTSEDTAVYFCANYGNYWFAYWGQGTQVTVSA

|.||||||.|...||||||.||||||||||||||.||||..||||||||.||||||||.||||||||||||.|.|||||.||||||||||.||||||.|||||||||||||.|||||

QVQLQQSGAELARPGASVKLSCKASGYTFTDYYINWVKQRTGQGLEWIGEIYPGSGNTYYNEKFKGKATLTADKSSSTAYMQLSSLTSEDSAVYFCARYGNYWFAYWGQGTLVTVSA

^^^^^^^^ ^^^^^^^^ ^^^^^^^^^^

Best Alignment of Brentuximab light chain CDRs to a sequence from OAS

DIVLTQSPASLAVSLGQRATISCKASQSVDFDGDSYMNWYQQKPGQPPKVLIYAASNLESGIPARFSGSGSGTDFTLNIHPVEEEDAATYYCQQSNEDPWTFGGGTKLEIK

|||.||.||||||||||||||||||||||||||||||||||||||||||.|||||||||||||||||||||||||||||||||||||||||||||||||||||||||||||

DIVITQTPASLAVSLGQRATISCKASQSVDFDGDSYMNWYQQKPGQPPKLLIYAASNLESGIPARFSGSGSGTDFTLNIHPVEEEDAATYYCQQSNEDPWTFGGGTKLEIK

^^^^^^^^^^ ^^^ ^^^^^^^^^

Best Alignment of Brentuximab CDR-H3 to a sequence from OAS

QIQLQQSGPEVVKPGASVKISCKASGYTFTDYYITWVKQKPGQGLEWIGWIYPGSGNTKYNEKFKGKATLTVDTSSSTAFMQLSSLTSEDTAVYFCANYGNYWFAYWGQGTQVTVSA

..||||||||.||||||||||||.||||||.|...||||..|..|||||.|.|..|.|.||.|||||||||||.|||||.|.|.||||||.|||.||||||||||||||||.|||||

EVQLQQSGPELVKPGASVKISCKTSGYTFTEYTMHWVKQSHGKSLEWIGGINPNNGGTSYNQKFKGKATLTVDKSSSTAYMELRSLTSEDSAVYYCANYGNYWFAYWGQGTLVTVSA

^^^^^^^^ ^^^^^^^^ ^^^^^^^^^^

Therapeutic : Briakinumab

Best Alignment of Briakinumab heavy chain to a sequence from OAS

QVQLVESGGGVVQPGRSLRLSCAASGFTFSSYGMHWVRQAPGKGLEWVAFIRYDGSNKYYADSVKGRFTISRDNSKNTLYLQMNSLRAEDTAVYYCKTHG---------SHDNWGQGTMVTVSS

||||||||||||||||||||||||||||||||||||||||||||||||||||||||||||||||||||||||||||||||||||||||||||||||.||............|.|||||||||||

QVQLVESGGGVVQPGRSLRLSCAASGFTFSSYGMHWVRQAPGKGLEWVAFIRYDGSNKYYADSVKGRFTISRDNSKNTLYLQMNSLRAEDTAVYYCATHPRYSSGWYWDAFDIWGQGTMVTVSS

^^^^^^^^ ^^^^^^^^ ^^^^^^^^^^^^^^^^^

Best Alignment of Briakinumab light chain to a sequence from OAS

QSVLTQPPSVSGAPGQRVTISCSGSRSNIGSNTVKWYQQLPGTAPKLLIYYNDQRPSGVPDRFSGSKSGTSASLAITGLQAEDEADYYCQSYDRYTHPALLFGTGTKVTVL

|||||||||.||.||||||||||||.||||||||.|||||||||||||||.|.||||||||||||||||||||||||||||.|||||||||||.....||.||||||||||

QSVLTQPPSASGTPGQRVTISCSGSSSNIGSNTVNWYQQLPGTAPKLLIYRNNQRPSGVPDRFSGSKSGTSASLAITGLQADDEADYYCQSYDGS-LSALVFGTGTKVTVL

^^^^^^^^ ^^^ ^^^^^^^^^^^^

Best Alignment of Briakinumab heavy chain CDRs to a sequence from OAS

QVQLVESGGGVVQPGRSLRLSCAASGFTFSSYGMHWVRQAPGKGLEWVAFIRYDGSNKYYADSVKGRFTISRDNSKNTLYLQMNSLRAEDTAVYYCKTHGSHDNWGQGTMVTVSS

................||||||||||||||||||||||||||||||||||||||||||||||||||||||||||||||||||||||||||||||||.|.||||.|||||.|||||

----------------SLRLSCAASGFTFSSYGMHWVRQAPGKGLEWVAFIRYDGSNKYYADSVKGRFTISRDNSKNTLYLQMNSLRAEDTAVYYCATSGSHDYWGQGTLVTVSS

^^^^^^^^ ^^^^^^^^ ^^^^^^^^

Best Alignment of Briakinumab light chain CDRs to a sequence from OAS

QSVLTQPPSVSGAPGQRVTISCSGSRSNIGSNTVKWYQQLPGTAPKLLIYYNDQRPSGVPDRFSGSKSGTSASLAITGLQAEDEADYYCQSYDRYTHPALLFGTGTKVTVL

|||||||||.||.|||.|||||||||||||||||.|||||||||||||||.|||||||||||||||||||||||||||||||||||||||||||.......||.|||.|||

QSVLTQPPSASGTPGQSVTISCSGSRSNIGSNTVTWYQQLPGTAPKLLIYTNDQRPSGVPDRFSGSKSGTSASLAITGLQAEDEADYYCQSYDRRLSASVVFGGGTKLTVL

^^^^^^^^ ^^^ ^^^^^^^^^^^^

Best Alignment of Briakinumab CDR-H3 to a sequence from OAS

QVQLVESGGGVVQPGRSLRLSCAASGFTFSSYGMHWVRQAPGKGLEWVAFIRYDGSNKYYADSVKGRFTISRDNSKNTLYLQMNSLRAEDTAVYYCKTHGSHDNWGQGTMVTVSS

.|||..||...|.||.|..|||.||||......||||.|.|..||||...|........||....|..||..|.|.||.|||..||..||||||||.||||.||||||...||.|

-VQLQQSGAELVRPGASVKLSCTASGFNIKDDYMHWVKQRPEQGLEWIGWIDPENGDTEYASKFQGKATITADTSSNTAYLQLSSLTSEDTAVYYCTTHGSPDNWGQGDTLTVCS

^^^^^^^^ ^^^^^^^^ ^^^^^^^^

Therapeutic : Brodalumab

Best Alignment of Brodalumab heavy chain to a sequence from OAS

QVQLVQSGAEVKKPGASVKVSCKASGYTFTRYGISWVRQAPGQGLEWMGWISTYSGNTNYAQKLQGRVTMTTDTSTSTAYMELRSLRSDDTAVYYCARRQL---YFDYWGQGTLVTVSS

||||||||||||||||||||||||||||||.|||||||||||||||||||||.|||||||||||||||||||||||||||||||||||||||||||||.||...|||||||||||||||

QVQLVQSGAEVKKPGASVKVSCKASGYTFTSYGISWVRQAPGQGLEWMGWISAYSGNTNYAQKLQGRVTMTTDTSTSTAYMELRSLRSDDTAVYYCARTQLLKAYFDYWGQGTLVTVSS

^^^^^^^^ ^^^^^^^^ ^^^^^^^^^^^^

Best Alignment of Brodalumab light chain to a sequence from OAS

EIVMTQSPATLSVSPGERATLSCRASQSVSSNLAWFQQKPGQAPRPLIYDASTRATGVPARFSGSGSGTDFTLTISSLQSEDFAVYYCQQYDNWPLTFGGGTKVEIK

|||||||||||||||||||||||||||||||||.|.|||||||||.|||||||||||||||||||||||||||||||||||||||||||||.|||||||||||||||

EIVMTQSPATLSVSPGERATLSCRASQSVSSNLTWYQQKPGQAPRLLIYDASTRATGVPARFSGSGSGTDFTLTISSLQSEDFAVYYCQQYNNWPLTFGGGTKVEIK

^^^^^^ ^^^ ^^^^^^^^^

Best Alignment of Brodalumab heavy chain CDRs to a sequence from OAS

QVQLVQSGAEVKKPGASVKVSCKASGYTFTRYGISWVRQAPGQGLEWMGWISTYSGNTNYAQKLQGRVTMTTDTSTSTAYMELRSLRSDDTAVYYCARRQLYFDYWGQGTLVTVSS

...............|||||||||||||||.|||||||||||||||||||||||.|||||||||||.|||||||.|||||||||||.||||||||||||.||||||||||||||||

---------------ASVKVSCKASGYTFTNYGISWVRQAPGQGLEWMGWISTYTGNTNYAQKLQGSVTMTTDTATSTAYMELRSLTSDDTAVYYCARRALYFDYWGQGTLVTVSS

^^^^^^^^ ^^^^^^^^ ^^^^^^^^^

Best Alignment of Brodalumab light chain CDRs to a sequence from OAS

EIVMTQSPATLSVSPGERATLSCRASQSVSSNLAWFQQKPGQAPRPLIYDASTRATGVPARFSGSGSGTDFTLTISSLQSEDFAVYYCQQYDNWPLTFGGGTKVEIK

..||||||||||||.|.||||||||.||||||.||.||||.||||.|||||||||||||||||.|||||.||||||||||||||||||||||||||||||||.||||

--VMTQSPATLSVSLGDRATLSCRAGQSVSSNVAWYQQKPEQAPRILIYDASTRATGVPARFSCSGSGTEFTLTISSLQSEDFAVYYCQQYDNWPLTFGGGTNVEIK

^^^^^^ ^^^ ^^^^^^^^^

Best Alignment of Brodalumab CDR-H3 to a sequence from OAS

QVQLVQSGAEVKKPGASVKVSCKASGYTFTRYGISWVRQAPGQGLEWMGWISTYSGNTNYAQKLQGRVTMTTDTSTSTAYMELRSLRSDDTAVYYCARRQLYFDYWGQGTLVTVSS

.|||.|||||...||||||.||||||||||.|...||.|.||.||||.|........|||.||..|..|.|.|.|.|||||.|.||.|.|.|||||||||||||||||||..||||

-VQLQQSGAELVMPGASVKLSCKASGYTFTSYWMHWVKQRPGKGLEWIGEFDPSDSYTNYNQKFKGKSTLTVDKSSSTAYMQLSSLTSEDSAVYYCARRQLYFDYWGQGTTLTVSS

^^^^^^^^ ^^^^^^^^ ^^^^^^^^^

Therapeutic : Burosumab

Best Alignment of Burosumab heavy chain to a sequence from OAS

QVQLVQSGAEVKKPGASVKVSCKASGYTFTNHYMHWVRQAPGQGLEWMGIINPISGSTSNAQKFQGRVTMTRDTSTSTVYMELSSLRSEDTAVYYCARDIV-------DAFDFWGQGTMVTVSS

||||||||||||||||||||||||||||||..|||||||||||||||||||||..||||.||||||||||||||||||||||||||||||||||||||.||.......||||.|||||||||||

QVQLVQSGAEVKKPGASVKVSCKASGYTFTSYYMHWVRQAPGQGLEWMGIINPSGGSTSYAQKFQGRVTMTRDTSTSTVYMELSSLRSEDTAVYYCARNIVVVPAATGDAFDIWGQGTMVTVSS

^^^^^^^^ ^^^^^^^^ ^^^^^^^^^^^^^^^^^

Best Alignment of Burosumab light chain to a sequence from OAS

AIQLTQSPSSLSASVGDRVTITCRASQGISSALVWYQQKPGKAPKLLIYDASSLESGVPSRFSGSGSGTDFTLTISSLQPEDFATYYCQQFN-DYFTFGPGTKVDIK

|||||||||||||||||||||||||||||||||.||||||||||||||||||||||||||||||||||||||||||||||||||||||||||..|||||||||||||

AIQLTQSPSSLSASVGDRVTITCRASQGISSALAWYQQKPGKAPKLLIYDASSLESGVPSRFSGSGSGTDFTLTISSLQPEDFATYYCQQFNNYYFTFGPGTKVDIK

^^^^^^ ^^^ ^^^^^^^^^

Best Alignment of Burosumab heavy chain CDRs to a sequence from OAS

QVQLVQSGAEVKKPGASVKVSCKASGYTFTNHYMHWVRQAPGQGLEWMGIINPISGSTSNAQKFQGRVTMTRDTSTSTVYMELSSLRSEDTAVYYCARDIVDAFDFWGQGTMVTVSS

................||||||||||||||..|||||||||||||||||||||.|||||.||||||||||||||||||||||||||||||||||||||||.||||.|||||||||||

----------------SVKVSCKASGYTFTSYYMHWVRQAPGQGLEWMGIINPSSGSTSYAQKFQGRVTMTRDTSTSTVYMELSSLRSEDTAVYYCARDISDAFDIWGQGTMVTVSS

^^^^^^^^ ^^^^^^^^ ^^^^^^^^^^

Best Alignment of Burosumab light chain CDRs to a sequence from OAS

AIQLTQSPSSLSASVGDRVTITCRASQGISSALVWYQQKPGKAPKLLIYDASSLESGVPSRFSGSGSGTDFTLTISSLQPEDFATYYCQQFNDYFTFGPGTKVDIK

|||||||||||||||||||||||||||||||||.||||||||||||||||||||||||||||||||||||||||||||||||||||||||||.|||||.||...||

AIQLTQSPSSLSASVGDRVTITCRASQGISSALAWYQQKPGKAPKLLIYDASSLESGVPSRFSGSGSGTDFTLTISSLQPEDFATYYCQQFNSYFTFGQGTRLEIK

^^^^^^ ^^^ ^^^^^^^^

Best Alignment of Burosumab CDR-H3 to a sequence from OAS

QVQLVQSGAEVKKPGASVKVSCKASGYTFTNHYMHWVRQAPGQGLEWMGIINPISGSTSNAQKFQGRVTMTRDTSTSTVYMELSSLRSEDTAVYYCARDIVDAFDFWGQGTMVTVSS

||||||||.||||||||||||||.|||||..|...||||.|||||||||.|....|.|..||.|||||.||.||||||.||||..|.|.|||||.||||.|||||||||||.|||||

QVQLVQSGTEVKKPGASVKVSCKTSGYTFSGHGISWVRQTPGQGLEWMGWISGSNGKTIYAQRFQGRVSMTTDTSTSTGYMELRNLTSDDTAVYFCARDRVDAFDFWGQGTVVTVSS

^^^^^^^^ ^^^^^^^^ ^^^^^^^^^^

Therapeutic : Camrelizumab

Best Alignment of Camrelizumab heavy chain to a sequence from OAS

EVQLVESGGGLVQPGGSLRLSCAASGFTFSSYMMSWVRQAPGKGLEWVATISGGGANTYYPDSVKGRFTISRDNAKNSLYLQMNSLRAEDTAVYYCARQLY--------YFDYWGQGTTVTVSS

||||||||||||||||||||||||||||||||.|||||||||||||||..|||.|..|||.||||||||||||||||||||||||||||||||||||||.|.........||||||||||||||

EVQLVESGGGLVQPGGSLRLSCAASGFTFSSYAMSWVRQAPGKGLEWVSAISGSGGSTYYADSVKGRFTISRDNAKNSLYLQMNSLRAEDTAVYYCARQTYCGGDCYSGIFDYWGQGTTVTVSS

^^^^^^^^ ^^^^^^^^ ^^^^^^^^^^^^^^^^^

Best Alignment of Camrelizumab light chain to a sequence from OAS

DIQMTQSPSSLSASVGDRVTITCLASQTIGTWLTWYQQKPGKAPKLLIYTATSLADGVPSRFSGSGSGTDFTLTISSLQPEDFATYYCQQVYSIPWTFGGGTKVEIK

|||||||||||||||||||||||.|||||||.|.|||||||||||||||.|.||..||||||||||||||||||||||||||||||||||.||.|||||.|||||||

DIQMTQSPSSLSASVGDRVTITCRASQTIGTTLNWYQQKPGKAPKLLIYAASSLQSGVPSRFSGSGSGTDFTLTISSLQPEDFATYYCQQSYSTPWTFGQGTKVEIK

^^^^^^ ^^^ ^^^^^^^^^

Best Alignment of Camrelizumab heavy chain CDRs to a sequence from OAS

EVQLVESGGGLVQPGGSLRLSCAASGFTFSSYMMSWVRQAPGKGLEWVATISGGGANTYYPDSVKGRFTISRDNAKNSLYLQMNSLRAEDTAVYYCARQLYYFDYWGQGTTVTVSS

||||||||||||.|||||.|||||||||||||.||||||.|.|.|||||||||||.||||||||||||.||||||||.|||||.|||.||||.||||||||||||||.|||.||||

EVQLVESGGGLVKPGGSLKLSCAASGFTFSSYTMSWVRQTPEKRLEWVATISGGGGNTYYPDSVKGRFPISRDNAKNNLYLQMSSLRSEDTALYYCARQLYYFDYWGKGTTLTVSS

^^^^^^^^ ^^^^^^^^ ^^^^^^^^^

Best Alignment of Camrelizumab light chain CDRs to a sequence from OAS

DIQMTQSPSSLSASVGDRVTITCLASQTIGTWLTWYQQKPGKAPKLLIYTATSLADGVPSRFSGSGSGTDFTLTISSLQPEDFATYYCQQVYSIPWTFGGGTKVEIK

||||||||.|.|||.|..|||||||||||||||.||||||||.|.||||||||||||||||||||||||.|...|||||.|||..|||||.||.|||||||||.|||

DIQMTQSPASQSASLGESVTITCLASQTIGTWLAWYQQKPGKSPQLLIYTATSLADGVPSRFSGSGSGTKFSFKISSLQAEDFVSYYCQQLYSTPWTFGGGTKLEIK

^^^^^^ ^^^ ^^^^^^^^^

Best Alignment of Camrelizumab CDR-H3 to a sequence from OAS

EVQLVESGGGLVQPGGSLRLSCAASGFTFSSYMMSWVRQAPGKGLEWVATISGGGANTYYPDSVKGRFTISRDNAKNSLYLQMNSLRAEDTAVYYCARQLYYFDYWGQGTTVTVSS

.|||..||..||.||.|..|||.|||.||..|...||.|.||.||||.|.|..|..||||....||..|..........|.|..||..||.|||.||||||||||||||||.||||

QVQLKQSGAELVRPGASVKLSCKASGYTFTDYYINWVKQRPGQGLEWIARIYPGSGNTYYNEKFKGKATLTAEKSSSTAYMQLSSLTSEDSAVYFCARQLYYFDYWGQGTTLTVSS

^^^^^^^^ ^^^^^^^^ ^^^^^^^^^

Therapeutic : Canakinumab

Best Alignment of Canakinumab heavy chain to a sequence from OAS

QVQLVESGGGVVQPGRSLRLSCAASGFTFSVYGMNWVRQAPGKGLEWVAIIWYDGDNQYYADSVKGRFTISRDNSKNTLYLQMNGLRAEDTAVYYCARDLRT-----GPFDYWGQGTLVTVSS

||||||||||||||||||||||||||||||.|||.||||||||||||||.|||||.|.||||||||||||||||||||||||||.||||||||||||||||......||||||||||||||||

QVQLVESGGGVVQPGRSLRLSCAASGFTFSSYGMHWVRQAPGKGLEWVAVIWYDGSNKYYADSVKGRFTISRDNSKNTLYLQMNSLRAEDTAVYYCARDLRDHGGNSGPFDYWGQGTLVTVSS

^^^^^^^^ ^^^^^^^^ ^^^^^^^^^^^^^^^^

Best Alignment of Canakinumab light chain to a sequence from OAS

EIVLTQSPDFQSVTPKEKVTITCRASQSIGSSLHWYQQKPDQSPKLLIKYASQSFSGVPSRFSGSGSGTDFTLTINSLEAEDAAAYYCHQSSSLPFTFGPGTKVDIK

||||||||||||||||||||||||||||||||||||||||||||||||||||||||||||||||||||||||||||||||||||.||||||||||||||||||||||

EIVLTQSPDFQSVTPKEKVTITCRASQSIGSSLHWYQQKPDQSPKLLIKYASQSFSGVPSRFSGSGSGTDFTLTINSLEAEDAATYYCHQSSSLPFTFGPGTKVDIK

^^^^^^ ^^^ ^^^^^^^^^

Best Alignment of Canakinumab heavy chain CDRs to a sequence from OAS

QVQLVESGGGVVQPGRSLRLSCAASGFTFSVYGMNWVRQAPGKGLEWVAIIWYDGDNQYYADSVKGRFTISRDNSKNTLYLQMNGLRAEDTAVYYCARDLRTGPFDYWGQGTLVTVSS

..............|.||..||||||||||.|||.||||||||||||||.|||||.|.||||||||||||||||||||||||||.||||||||||||||||||.||||||||||||||

--------------GESLKISCAASGFTFSSYGMHWVRQAPGKGLEWVAVIWYDGSNKYYADSVKGRFTISRDNSKNTLYLQMNSLRAEDTAVYYCARDLRTGAFDYWGQGTLVTVSS

^^^^^^^^ ^^^^^^^^ ^^^^^^^^^^^

Best Alignment of Canakinumab light chain CDRs to a sequence from OAS

EIVLTQSPDFQSVTPKEKVTITCRASQSIGSSLHWYQQKPDQSPKLLIKYASQSFSGVPSRFSGSGSGTDFTLTINSLEAEDAAAYYCHQSSSLPFTFGPGTKVDIK

|.||||||||||||.|||||.|||||||||||.|||.|||||||||||||||||.||||||||||||||||.||||||||||||.||||||||||||||.||...||

EVVLTQSPDFQSVTSKEKVTVTCRASQSIGSSFHWYNQKPDQSPKLLIKYASQSISGVPSRFSGSGSGTDFALTINSLEAEDAATYYCHQSSSLPFTFGQGTRLEIK

^^^^^^ ^^^ ^^^^^^^^^

Best Alignment of Canakinumab CDR-H3 to a sequence from OAS

QVQLVESGGGVVQPGRSLRLSCAASGFTFSVYGMNWVRQAPGKGLEWVAIIWYDGDNQYYADSVKGRFTISRDNSKNTLYLQMNGLRAEDTAVYYCARDLRTGPFDYWGQGTLVTVSS

..............|.||||||||||||||.|.|.||||||||||.||..|..||....|||||||||||||||.|||||||||.|||||||||||||||||||||||||||||||||

--------------GGSLRLSCAASGFTFSSYWMHWVRQAPGKGLVWVSRINTDGSSTSYADSVKGRFTISRDNAKNTLYLQMNSLRAEDTAVYYCARDLRTGPFDYWGQGTLVTVSS

^^^^^^^^ ^^^^^^^^ ^^^^^^^^^^^

Therapeutic : Carlumab

Best Alignment of Carlumab heavy chain to a sequence from OAS

QVQLVQSGAEVKKPGSSVKVSCKASGGTFSSYGISWVRQAPGQGLEWMGGIIPIFGTANYAQKFQGRVTITADESTSTAYMELSSLRSEDTAVYYCARYDGI-------YGELDFWGQGTLVTVSS

||||||||||||||||||||||||||||||||.|||||||||||||||||||||||||||||||||||||||||||||||||||||||||||||||||..||........||||.|||||||||||

QVQLVQSGAEVKKPGSSVKVSCKASGGTFSSYAISWVRQAPGQGLEWMGGIIPIFGTANYAQKFQGRVTITADESTSTAYMELSSLRSEDTAVYYCARDLGIVVVPAALFGELDYWGQGTLVTVSS

^^^^^^^^ ^^^^^^^^ ^^^^^^^^^^^^^^^^^^^

Best Alignment of Carlumab light chain to a sequence from OAS

EIVLTQSPATLSLSPGERATLSCRASQSVSDAYLAWYQQKPGQAPRLLIYDASSRATGVPARFSGSGSGTDFTLTISSLEPEDFAVYYCHQYIQLHSFTFGQGTKVEIK

|||||||||||||||||||||||||||||.|.|||||||||||||||||||||.|||||||||||||||||||||||||||||||||||.||....|.|||||||||||

EIVLTQSPATLSLSPGERATLSCRASQSV-DSYLAWYQQKPGQAPRLLIYDASYRATGVPARFSGSGSGTDFTLTISSLEPEDFAVYYCQQYG--GSPTFGQGTKVEIK

^^^^^^^ ^^^ ^^^^^^^^^^

Best Alignment of Carlumab heavy chain CDRs to a sequence from OAS

QVQLVQSGAEVKKPGSSVKVSCKASGGTFSSYGISWVRQAPGQGLEWMGGIIPIFGTANYAQKFQGRVTITADESTSTAYMELSSLRSEDTAVYYCARYDGIYGELDFWGQGTLVTVSS

||||||||||||||||||||||||||||||||.|||||||||||||||||||||||||||||||||||||||||||||||||||||||||||||||||.||.|.|||.|||||||||||

QVQLVQSGAEVKKPGSSVKVSCKASGGTFSSYAISWVRQAPGQGLEWMGGIIPIFGTANYAQKFQGRVTITADESTSTAYMELSSLRSEDTAVYYCAREDGGYYELDYWGQGTLVTVSS

^^^^^^^^ ^^^^^^^^ ^^^^^^^^^^^^

Best Alignment of Carlumab light chain CDRs to a sequence from OAS

EIVLTQSPATLSLSPGERATLSCRASQSVSDAYLAWYQQKPGQAPRLLIYDASSRATGVPARFSGSGSGTDFTLTISSLEPEDFAVYYCHQYIQLHSFTFGQGTKVEIK

.|||||||||||.||||||||||.||||||..|||.||.|||||||||.||||.|..|...||.||||||..|||.|..||.||||||||||....|||.||||.||||

-IVLTQSPATLSVSPGERATLSCGASQSVSQNYLARYQKKPGQAPRLLMYDASRRDIGIADRFRGSGSGTNVTLTTSRVEPKDFAVYYCHQYGSPPSFTIGQGTQVEIK

^^^^^^^ ^^^ ^^^^^^^^^^

Best Alignment of Carlumab CDR-H3 to a sequence from OAS

QVQLVQSGAEVKKPGSSVKVSCKASGGTFSSYGISWVRQAPGQGLEWMGGIIPIFGTANYAQKFQGRVTITADESTSTAYMELSSLRSEDTAVYYCARYDGIYGELDFWGQGTLVTVSS

||||.|||||..|||.|||.||||.|.||..|.|.||.|.||.||||.|.|.|..|..||..||.|..|.|||.|..||||.||||..||.|.||||||||.|||||.|||||..||||

QVQLQQSGAELMKPGASVKLSCKATGYTFTGYWIEWVKQRPGHGLEWIGEILPGSGSTNYNEKFKGKATFTADTSSNTAYMQLSSLTTEDSAIYYCARYDGYYGELDYWGQGTTLTVSS

^^^^^^^^ ^^^^^^^^ ^^^^^^^^^^^^

Therapeutic : Carotuximab

Best Alignment of Carotuximab heavy chain to a sequence from OAS

EVKLEESGGGLVQPGGSMKLSCAASGFTFSDAWMDWVRQSPEKGLEWVAEIRSKASNHATYYAESVKGRFTISRDDSKSSVYLQMNSLRAEDTGIYYCTRWRR--FFDSWGQGTTLTVSS

|||||||||||||||||||||||||||||||||||||||||||||||||||||||.||||||||||||||||||||||||||||||||||||||||||||.|...|||.|||||||||||

EVKLEESGGGLVQPGGSMKLSCAASGFTFSDAWMDWVRQSPEKGLEWVAEIRSKANNHATYYAESVKGRFTISRDDSKSSVYLQMNSLRAEDTGIYYCTRRRLGGFFDYWGQGTTLTVSS

^^^^^^^^ ^^^^^^^^^^ ^^^^^^^^^^^

Best Alignment of Carotuximab light chain to a sequence from OAS

QIVLSQSPAILSASPGEKVTMTCRASSSVSYMHWYQQKPGSSPKPWIYATSNLASGVPVRFSGSGSGTSYSLTISRVEAEDAATYYCQQWSSNPLTFGAGTKLELK

||||||||||||||||||||||||||||||||||||||||||||||||||||||||||||||||||||||||||||||||||||||||||||||||||||||||||

QIVLSQSPAILSASPGEKVTMTCRASSSVSYMHWYQQKPGSSPKPWIYATSNLASGVPVRFSGSGSGTSYSLTISRVEAEDAATYYCQQWSSNPLTFGAGTKLELK

^^^^^ ^^^ ^^^^^^^^^

Best Alignment of Carotuximab heavy chain CDRs to a sequence from OAS

EVKLEESGGGLVQPGGSMKLSCAASGFTFSDAWMDWVRQSPEKGLEWVAEIRSKASNHATYYAESVKGRFTISRDDSKSSVYLQMNSLRAEDTGIYYCTRWRRFFDSWGQGTTLTVSS

||.|.||||||||||||||||||||||||||||||||||||||||||||||||||.||||||||||||||||||||||||||||||||||||||||||||.||.||.|||||||||||

EVMLVESGGGLVQPGGSMKLSCAASGFTFSDAWMDWVRQSPEKGLEWVAEIRSKANNHATYYAESVKGRFTISRDDSKSSVYLQMNSLRAEDTGIYYCTRGRRYFDYWGQGTTLTVSS

^^^^^^^^ ^^^^^^^^^^ ^^^^^^^^^

Best Alignment of Carotuximab light chain CDRs to a sequence from OAS

QIVLSQSPAILSASPGEKVTMTCRASSSVSYMHWYQQKPGSSPKPWIYATSNLASGVPVRFSGSGSGTSYSLTISRVEAEDAATYYCQQWSSNPLTFGAGTKLELK

.|||.|||...|.||||||||||.||||||||.||||||||||.|.||||||||||||.|||||||||||||||||.|||||||||||||||||||||||||||.|

HIVLTQSPELMSVSPGEKVTMTCSASSSVSYMYWYQQKPGSSPRPLIYATSNLASGVPARFSGSGSGTSYSLTISRMEAEDAATYYCQQWSSNPLTFGAGTKLEIK

^^^^^ ^^^ ^^^^^^^^^

Best Alignment of Carotuximab CDR-H3 to a sequence from OAS

EVKLEESGGGLVQPGGSMKLSCAASGFTF--SDAWMDWVRQSPEKGLEWVAEIRSKASNHATYYAESVKGRFTISRDDSKSSVYLQMNSLRAEDTGIYYCTRWRRFFDSWGQGTTLTVSS

...................|.|...|...........|.||||..||||......|..|....||.|||.|...|.|.|.....|..||....|...|||||.||.||||||||..||||

--------------SQTLSLTCVIYGDSVSNKNSAWNWIRQSPSRGLEWLGRTYYKS-NWYNEYAVSVKSRISFSADSSENQFSLHLNSVTPDDSAVYYCTRGRREFDSWGQGTLVTVSS

^^^^^^^^^^ ^^^^^^^^^^ ^^^^^^^^^

Therapeutic : Certolizumab

Best Alignment of Certolizumab heavy chain to a sequence from OAS

EVQLVESGGGLVQPGGSLRLSCAASGYVFTDYGMNWVRQAPGKGLEWMGWINTYIGEPIYADSVKGRFTFSLDTSKSTAYLQMNSLRAEDTAVYYCARGYRS---YAMDYWGQGTLVTVSS

||||||||||||||||||||||||||..|.||||.||||||||||||...||...|...||||||||||.|.|.||.|.|||||||||||||||||||..|....|..|||||||||||||

EVQLVESGGGLVQPGGSLRLSCAASGFTFDDYGMSWVRQAPGKGLEWVSGINWNGGSTGYADSVKGRFTISRDNSKNTLYLQMNSLRAEDTAVYYCAREGRGYGDYVIDYWGQGTLVTVSS

^^^^^^^^ ^^^^^^^^ ^^^^^^^^^^^^^^

Best Alignment of Certolizumab light chain to a sequence from OAS

DIQMTQSPSSLSASVGDRVTITCKASQNVGTNVAWYQQKPGKAPKALIYSASFLYSGVPYRFSGSGSGTDFTLTISSLQPEDFATYYCQQYNIYPLTFGQGTKVEIK

|||||||||||||||||||||||.||||..|...|||||||||||.|||.||.|.||||.|||||||||||||||||||||||||||||||.|||||||.|||||||

DIQMTQSPSSLSASVGDRVTITCRASQNIATYLNWYQQKPGKAPKLLIYAASSLQSGVPSRFSGSGSGTDFTLTISSLQPEDFATYYCQQYKIYPLTFGGGTKVEIK

^^^^^^ ^^^ ^^^^^^^^^

Best Alignment of Certolizumab heavy chain CDRs to a sequence from OAS

EVQLVESGGGLVQPGGSLRLSCAASGYVFTDYGMNWVRQAPGKGLEWMGWINTYIGEPIYADSVKGRFTFSLDTSKSTAYLQMNSLRAEDTAVYYCARGYRSYAMDYWGQGTLVTVSS

..|||.||..|..||.....||.||||.||.||||||.|||||||.||||||||.|||.|||..||||.|||.||.||||||.|.|..||||.|.||||..|||||||||||.|||||

QIQLVQSGPELKKPGETVKISCKASGYTFTNYGMNWVKQAPGKGLKWMGWINTYTGEPTYADDFKGRFAFSLETSASTAYLQINNLKNEDTATYFCARGGLSYAMDYWGQGTSVTVSS

^^^^^^^^ ^^^^^^^^ ^^^^^^^^^^^

Best Alignment of Certolizumab light chain CDRs to a sequence from OAS

DIQMTQSPSSLSASVGDRVTITCKASQNVGTNVAWYQQKPGKAPKALIYSASFLYSGVPYRFSGSGSGTDFTLTISSLQPEDFATYYCQQYNIYPLTFGQGTKVEIK

||.||.|....|.||||.|..||||||||||||||||||||..|||||||||..|||||.||.|||||||||||||..|.|..|.|.||||||||||||.|||.|||

DIVMTESQKFMSTSVGDGVSVTCKASQNVGTNVAWYQQKPGESPKALIYSASYRYSGVPGRFTGSGSGTDFTLTISNVQSENLAEYFCQQYNIYPLTFGSGTKLEIK

^^^^^^ ^^^ ^^^^^^^^^

Best Alignment of Certolizumab CDR-H3 to a sequence from OAS

EVQLVESGGGLVQPGGSLRLSCAASGYVFTDYGMNWVRQAPGKGLEWMGWINTYIGEPIYADSVKGRFTFSLDTSKSTAYLQMNSLRAEDTAVYYCARGYRSYAMDYWGQGTLVTVSS

.|.|.|||.|||.|..||...|..||...|.||..||||.|||||||.|.|....|...|......|...|.|.|||...|.||||...|||.|||||||||||||||||||.|||||

-VKLMESGPGLVAPSQSLSITCTVSGFSLTSYGVHWVRQPPGKGLEWLGVIWAG-GSTNYNSALMSRLSISKDNSKSQVFLKMNSLQTDDTAMYYCARGYRSYAMDYWGQGTSVTVSS

^^^^^^^^ ^^^^^^^^ ^^^^^^^^^^^

Therapeutic : Cetuximab

Best Alignment of Cetuximab heavy chain to a sequence from OAS

QVQLKQSGPGLVQPSQSLSITCTVSGFSLTNYGVHWVRQSPGKGLEWLGVIWSGGNTDYNTPFTSRLSINKDNSKSQVFFKMNSLQSNDTAIYYCARALTYYDYEFAYWGQGTLVTVSA

||||||||||||||||||||||||||||||.||||||||||||||||||||||||.||||..|.|||||.||||||||||||||||.||||||||||.|.|.|||||||||||||||||

QVQLKQSGPGLVQPSQSLSITCTVSGFSLTSYGVHWVRQSPGKGLEWLGVIWSGGSTDYNAAFISRLSISKDNSKSQVFFKMNSLQANDTAIYYCARGLYY-DYEFAYWGQGTLVTVSA

^^^^^^^^ ^^^^^^^ ^^^^^^^^^^^^^

Best Alignment of Cetuximab light chain to a sequence from OAS

DILLTQSPVILSVSPGERVSFSCRASQSIGTNIHWYQQRTNGSPRLLIKYASESISGIPSRFSGSGSGTDFTLSINSVESEDIADYYCQQNNNWPTTFGAGTKLELK

||||||||.||||||||||||||||||||||.|||||||||||||||||||||||||||||||||||||||||||||||||||||||||||||||.|||||||||||

DILLTQSPAILSVSPGERVSFSCRASQSIGTSIHWYQQRTNGSPRLLIKYASESISGIPSRFSGSGSGTDFTLSINSVESEDIADYYCQQNNNWPLTFGAGTKLELK

^^^^^^ ^^^ ^^^^^^^^^

Best Alignment of Cetuximab heavy chain CDRs to a sequence from OAS

QVQLKQSGPGLVQPSQSLSITCTVSGFSLTNYGVHWVRQSPGKGLEWLGVIWSGGNTDYNTPFTSRLSINKDNSKSQVFFKMNSLQSNDTAIYYCARALTYYDYEFAYWGQGTLVTVSA

.|.|..||||||||||||||||||||||||.||||||||||||||||||||||||.||||..|.|||||.||||||||||||||||..|||||||||.|.||||.||||||||||||||

-VMLVESGPGLVQPSQSLSITCTVSGFSLTSYGVHWVRQSPGKGLEWLGVIWSGGSTDYNAAFISRLSISKDNSKSQVFFKMNSLQADDTAIYYCARNLDYYDYGFAYWGQGTLVTVSA

^^^^^^^^ ^^^^^^^ ^^^^^^^^^^^^^

Best Alignment of Cetuximab light chain CDRs to a sequence from OAS

DILLTQSPVILSVSPGERVSFSCRASQSIGTNIHWYQQRTNGSPRLLIKYASESISGIPSRFSGSGSGTDFTLSINSVESEDIADYYCQQNNNWPTTFGAGTKLELK

||||||||.||||||||||||||||||||||.|||||||||||||||||||||||||||||||||||||.|||.|||||||||||||||||||||||||.|||||.|

DILLTQSPAILSVSPGERVSFSCRASQSIGTSIHWYQQRTNGSPRLLIKYASESISGIPSRFSGSGSGTYFTLTINSVESEDIADYYCQQNNNWPTTFGGGTKLEIK

^^^^^^ ^^^ ^^^^^^^^^

Best Alignment of Cetuximab CDR-H3 to a sequence from OAS

QVQLKQSGPGLVQPSQSLSITCTVSGFSLTNYGVHWVRQSPGKGLEWLGVIWSG-GNTDYNTPFTSRLSINKDNSKSQVFFKMNSLQSNDTAIYYCARALTYYDYEFAYWGQGTLVTVSA

.|||.|||..||.|..|....|..||...|.|...|..|..||.|||.|........|.||..|..........|.|.|......|.|.|.|.|||||||.|||||||||||||||||||

-VQLQQSGAELVKPGASVKMSCKASGYTFTTYPIEWMKQNHGKSLEWIGNFHPYNDDTKYNEKFKGKATLTVEKSSSTVYLELSRLTSDDSAVYYCARALIYYDYEFAYWGQGTLVTVSA

^^^^^^^^ ^^^^^^^^ ^^^^^^^^^^^^^

Therapeutic : Cixutumumab

Best Alignment of Cixutumumab heavy chain to a sequence from OAS

EVQLVQSGAEVKKPGSSVKVSCKASGGTFSSYAISWVRQAPGQGLEWMGGIIPIFGTANYAQKFQGRVTITADKSTSTAYMELSSLRSEDTAVYYCARAPLRFLEWSTQDHYYYYYMDVWGKGTTVTVSS

..|||||||||||||||||||||||||||||||||||||||||||||||||||||||||||||||||||||||||||||||||||||||||||||||||..|||||.....|||||||||||||||||||

--QLVQSGAEVKKPGSSVKVSCKASGGTFSSYAISWVRQAPGQGLEWMGGIIPIFGTANYAQKFQGRVTITADKSTSTAYMELSSLRSEDTAVYYCARASRRFLEWL-LEDYYYYYMDVWGKGTTVTVSS

^^^^^^^^ ^^^^^^^^ ^^^^^^^^^^^^^^^^^^^^^^^

Best Alignment of Cixutumumab light chain to a sequence from OAS

SSELTQDPAVSVALGQTVRITCQGDSLRSYYATWYQQKPGQAPILVIYGENKRPSGIPDRFSGSSSGNTASLTITGAQAEDEADYYCKSRDGSGQHLVFGGGTKLTVL

||||||||||||||||||||||||||||||||.||||||||||.|||||||.|||||||||||||||||||||||||||||||||||.|||.||.|||||||||||||

SSELTQDPAVSVALGQTVRITCQGDSLRSYYASWYQQKPGQAPVLVIYGENNRPSGIPDRFSGSSSGNTASLTITGAQAEDEADYYCNSRDSSGNHLVFGGGTKLTVL

^^^^^^ ^^^ ^^^^^^^^^^^

Best Alignment of Cixutumumab heavy chain CDRs to a sequence from OAS

EVQLVQSGAEVKKPGSSVKVSCKASGGTFSSYAISWVRQAPGQGLEWMGGIIPIFGTANYAQKFQGRVTITADKSTSTAYMELSSLRSEDTAVYYCARAPLRFLEWSTQDHYYYYYMDVWGKGTTVTVSS

................||||||||||||||||||||||.||||||||||||||||||||||||||||||||||.||||||||||||||||||||||||.|||||||||...||||||||||||||||||.

---------------ASVKVSCKASGGTFSSYAISWVREAPGQGLEWMGGIIPIFGTANYAQKFQGRVTITADESTSTAYMELSSLRSEDTAVYYCARCPLRFLEWSTVGYYYYYYMDVWGKGTTVTVS-

^^^^^^^^ ^^^^^^^^ ^^^^^^^^^^^^^^^^^^^^^^^

Best Alignment of Cixutumumab light chain CDRs to a sequence from OAS

SSELTQDPAVSVALGQTVRITCQGDSLRSYYATWYQQKPGQAPILVIYGENKRPSGIPDRFSGSSSGNTASLTITGAQAEDEADYYCKSRDGSGQHLVFGGGTKLTVL

||||||||||||||||||||||||||||||.|||||||.|||||||.||||.||||||.|||||||||||||||||.||||||||||.|||.|||||||.||||.|||

SSELTQDPAVSVALGQTVRITCQGDSLRSYHATWYQQKQGQAPILVMYGENNRPSGIPERFSGSSSGNTASLTITGTQAEDEADYYCNSRDSSGQHLVFAGGTKVTVL

^^^^^^ ^^^ ^^^^^^^^^^^

Best Alignment of Cixutumumab CDR-H3 to a sequence from OAS

EVQLVQSGAEVKKPGSSVKVSCKASGGTFSSYAISWVRQAPGQGLEWMGGIIPIFGTANYAQKFQGRVTITADKSTSTAYMELSSLRSEDTAVYYCARAPLRFLEWSTQDHYYYYYMDVWGKGTTVTVSS

................||||||||||||||||||||||.||||||||||||||||||||||||||||||||||.||||||||||||||||||||||||.|||||||||...||||||||||||||||||.

---------------ASVKVSCKASGGTFSSYAISWVREAPGQGLEWMGGIIPIFGTANYAQKFQGRVTITADESTSTAYMELSSLRSEDTAVYYCARCPLRFLEWSTVGYYYYYYMDVWGKGTTVTVS-

^^^^^^^^ ^^^^^^^^ ^^^^^^^^^^^^^^^^^^^^^^^

Therapeutic : Clazakizumab

Best Alignment of Clazakizumab heavy chain to a sequence from OAS

EVQLVESGGGLVQPGGSLRLSCAASGFSLSNYYVTWVRQAPGKGLEWVGIIYGS-DETAYATSAIGRFTISRDNSKNTLYLQMNSLRAEDTAVYYCARDDSSD-WDAKFNLWGQGTLVTVSS

||||.||||||||||||||||||||||..|||..||||||||||||||..|.||...|.||.|..||||||||||||||||||||||||||||||||||.||......|..|||||||||||

EVQLLESGGGLVQPGGSLRLSCAASGFTFSNYAMTWVRQAPGKGLEWVSAISGSGGSTYYADSVKGRFTISRDNSKNTLYLQMNSLRAEDTAVYYCARDPSSGYYGGFFDYWGQGTLVTVSS

^^^^^^^^ ^^^^^^^^ ^^^^^^^^^^^^^^^

Best Alignment of Clazakizumab light chain to a sequence from OAS

AIQMTQSPSSLSASVGDRVTITCQASQSINNELSWYQQKPGKAPKLLIYRASTLASGVPSRFSGSGSGTDFTLTISSLQPDDFATYYCQQGYSLRNIDNAFGGGTKVEIK

.||||||||||||||||||||||.|||||||.|||||||||||||||||.||.|.|||||||||||||||||||||||||.||||||||||||.......||||||||||

DIQMTQSPSSLSASVGDRVTITCRASQSINNYLSWYQQKPGKAPKLLIYAASSLQSGVPSRFSGSGSGTDFTLTISSLQPEDFATYYCQQGYS--TPRITFGGGTKVEIK

^^^^^^ ^^^ ^^^^^^^^^^^^

Best Alignment of Clazakizumab heavy chain CDRs to a sequence from OAS

EVQLVESGGGLVQPGGSLRLSCAASGFSLSNYYVTWVRQAPGKGLEWVGIIYGSDETAYATSAIGRFTISRDNSKNTLYLQMNSLRAEDTAVYYCARDDSSDWDAKFNLWGQGTLVTVSS

..||.||||.||.|.|||.|||.||||.||.||..||||||||||||.|.|..|..|.||....|||.||.||..||..||||||.|.|||.|.||||.||||...|||||.|||||||.

--QLEESGGDLVKPEGSLTLSCKASGFDLSTYYMNWVRQAPGKGLEWIGMILPSGTTYYASWVNGRFAISSDNAQNTVDLQMNSLTAADTATYFCARDYSSDWGWEFNLWGPGTLVTVS-

^^^^^^^^ ^^^^^^^ ^^^^^^^^^^^^^^

Best Alignment of Clazakizumab light chain CDRs to a sequence from OAS

AIQMTQSPSSLSASVGDRVTITCQASQSINNELSWYQQKPGKAPKLLIYRASTLASGVPSRFSGSGSGTDFTLTISSLQPDDFATYYCQQGYSLRNIDNAFGGGTKVEIK

..................|.|||.|||||||.|.|||||||||||||||.||.|.|||||||||||||||||||||||||.|||||||||.||.....|.||.|||.|||

------------------VSITCRASQSINNYLNWYQQKPGKAPKLLIYAASSLQSGVPSRFSGSGSGTDFTLTISSLQPEDFATYYCQQSYSDPPKLNTFGQGTKLEIK

^^^^^^ ^^^ ^^^^^^^^^^^^

Best Alignment of Clazakizumab CDR-H3 to a sequence from OAS

EVQLVESGGGLVQPGGSLRLSCAASGFSL-SNYYVTWVRQAPGKGLEWVGIIYGS-DETAYATSAIGRFTISRDNSKNTLYLQMNSLRAEDTAVYYCARDDSSDWDAKFNLWGQGTLVTVSS

...|.|||||||||||||.|.|.|||||....|...||||||||||||...|..|...|.||..|.|.||||...|..|..||||||.|.|||.|.|||||||||...|||||.|||||||.

Q-SLEESGGGLVQPGGSLTLTCKASGFSFSNRYVMCWVRQAPGKGLEWIACINTSSGNTVYASWAKGPFTISKTSS-TTVTLQMNSLTAADTATYFCARDDSSDWGWEFNLWGPGTLVTVS-

^^^^^^^^^ ^^^^^^^^ ^^^^^^^^^^^^^^

Therapeutic : Codrituzumab

Best Alignment of Codrituzumab heavy chain to a sequence from OAS

QVQLVQSGAEVKKPGASVKVSCKASGYTFTDYEMHWVRQAPGQGLEWMGALDPKTGDTAYSQKFKGRVTLTADKSTSTAYMELSSLTSEDTAVYYCTRFY-SYTYWGQGTLVTVSS

||||||||||||||||||||||||||||||.|.||||||||||||||||...|..|.|.|.|||.||||.||||||||||||||||.|||||||||.|.|..|.||||||||||||

QVQLVQSGAEVKKPGASVKVSCKASGYTFTSYYMHWVRQAPGQGLEWMGIINPSGGSTSYAQKFQGRVTITADKSTSTAYMELSSLRSEDTAVYYCARGYSNYDYWGQGTLVTVSS

^^^^^^^^ ^^^^^^^^ ^^^^^^^^^

Best Alignment of Codrituzumab light chain to a sequence from OAS

DVVMTQSPLSLPVTPGEPASISCRSSQSLVHSNRNTYLHWYLQKPGQSPQLLIYKVSNRFSGVPDRFSGSGSGTDFTLKISRVEAEDVGVYYCSQNTHVPPTFGQGTKLEIK

||||||.||||||.||.||||||||||||||||.|||||||||||||||.|||||||||||||||||||||||||||||||||||||.|||.|||.||||.|||.|||||||

DVVMTQTPLSLPVSPGDPASISCRSSQSLVHSNGNTYLHWYLQKPGQSPKLLIYKVSNRFSGVPDRFSGSGSGTDFTLKISRVEAEDLGVYFCSQSTHVPWTFGGGTKLEIK

^^^^^^^^^^^ ^^^ ^^^^^^^^^

Best Alignment of Codrituzumab heavy chain CDRs to a sequence from OAS

QVQLVQSGAEVKKPGASVKVSCKASGYTFTDYEMHWVRQAPGQGLEWMGALDPKTGDTAYSQKFKGRVTLTADKSTSTAYMELSSLTSEDTAVYYCTRFYSYTYWGQGTLVTVSS

.|||.|||||...|||||..|||||||||||||||||.|.|..||||.||.||.||.|||.|||||...||||||.||||.||.||||||.|||||||||.||||||||..||||

-VQLQQSGAELVRPGASVTLSCKASGYTFTDYEMHWVKQTPVHGLEWIGAIDPETGGTAYNQKFKGKAILTADKSSSTAYLELRSLTSEDSAVYYCTRFYGYTYWGQGTTLTVSS

^^^^^^^^ ^^^^^^^^ ^^^^^^^^

Best Alignment of Codrituzumab light chain CDRs to a sequence from OAS

DVVMTQSPLSLPVTPGEPASISCRSSQSLVHSNRNTYLHWYLQKPGQSPQLLIYKVSNRFSGVPDRFSGSGSGTDFTLKISRVEAEDVGVYYCSQNTHVPPTFGQGTKLEIK

||||||.||||||..|..|||||||||||||||.|||||||||||||||.|||||||||||||||||||||||||||||||||||||.|||.|||.||||||||.|||||||

DVVMTQTPLSLPVSLGDQASISCRSSQSLVHSNGNTYLHWYLQKPGQSPKLLIYKVSNRFSGVPDRFSGSGSGTDFTLKISRVEAEDLGVYFCSQSTHVPPTFGGGTKLEIK

^^^^^^^^^^^ ^^^ ^^^^^^^^^

Best Alignment of Codrituzumab CDR-H3 to a sequence from OAS

QVQLVQSGAEVKKPGASVKVSCKASGYTFTDYEMHWVRQAPGQGLEWMGALDPK--TGDTAYSQKFKGRVTLTADKSTSTAYMELSSLTSEDTAVYYCTRFYSYTYWGQGTLVTVSS

...||.||.....||.|.|.||.|||.||..|.|.||||.|..||||......|.....|.|....|||.|...|.|.|..|.....|..|||..|||||.|||||||.||..||||

DGMLVESGGGLVQPGGSMKLSCVASGFTFSNYWMNWVRQSPEKGLEWVAEIRLKSNNYATHYAESVKGRFTISRDDSKSSVYLQMNNLRAEDTGIYYCTRGYSYTYWGPGTTLTVSS

^^^^^^^^ ^^^^^^^^^^ ^^^^^^^^

Therapeutic : Coltuximab

Best Alignment of Coltuximab heavy chain to a sequence from OAS

QVQLVQPGAEVVKPGASVKLSCKTSGYTFTSNWMHWVKQAPGQGLEWIGEIDPSDSYTNYNQNFQGKAKLTVDKSTSTAYMEVSSLRSDDTAVYYCARGSNPYYYAMDYWGQGTSVTVSS

||||.|||||.||||||||||||.|||||||.||.||||.||||||||||||||||||||||.|.|||.||||.|.|||||..|||.|.|.|||||||||||||||||||||||||||||

QVQLQQPGAELVKPGASVKLSCKASGYTFTSYWMQWVKQRPGQGLEWIGEIDPSDSYTNYNQKFKGKATLTVDTSSSTAYMQLSSLTSEDSAVYYCARGSNPYYYAMDYWGQGTSVTVSS

^^^^^^^^ ^^^^^^^^ ^^^^^^^^^^^^^

Best Alignment of Coltuximab light chain to a sequence from OAS

EIVLTQSPAIMSASPGERVTMTCSASSGVNYMHWYQQKPGTSPRRWIYDTSKLASGVPARFSGSGSGTDYSLTISSMEPEDAATYYCHQRGSYTFGGGTKLEIK

.||||||||||||||||.|||||||||..||||||||||||||.||||||||||||||||||||||||.|||||||||.|||||||||||.|||||||||||||

QIVLTQSPAIMSASPGEKVTMTCSASSSINYMHWYQQKPGTSPKRWIYDTSKLASGVPARFSGSGSGTSYSLTISSMEAEDAATYYCHQRSSYTFGGGTKLEIK

^^^^^ ^^^ ^^^^^^^

Best Alignment of Coltuximab heavy chain CDRs to a sequence from OAS

QVQLVQPGAEVVKPGASVKLSCKTSGYTFTSNWMHWVKQAPGQGLEWIGEIDPSDSYTNYNQNFQGKAKLTVDKSTSTAYMEVSSLRSDDTAVYYCARGSNPYYYAMDYWGQGTSVTVSS

.|||.|||||.|.||||||||||.|||||||.|||||||.||||||||||||||||||||||.|.||..||||||.|||||..|||.|.|.|||||||||||||||||||||||||||||

-VQLQQPGAELVMPGASVKLSCKASGYTFTSYWMHWVKQRPGQGLEWIGEIDPSDSYTNYNQKFKGKSTLTVDKSSSTAYMQLSSLTSEDSAVYYCARGSNPYYYAMDYWGQGTSVTVSS

^^^^^^^^ ^^^^^^^^ ^^^^^^^^^^^^^

Best Alignment of Coltuximab light chain CDRs to a sequence from OAS

EIVLTQSPAIMSASPGERVTMTCSASSGVNYMHWYQQKPGTSPRRWIYDTSKLASGVPARFSGSGSGTDYSLTISSMEPEDAATYYCHQRGSYTFGGGTKLEIK

.||.||..||||||.||.|||.|.|||.||||.|||||...||.|||||||||||||||||||||.||.|||||||||.|||||||||||.|||||||||||||

-IVMTQTTAIMSASLGEKVTMSCRASSSVNYMYWYQQKSDASPKRWIYDTSKLASGVPARFSGSGPGTSYSLTISSMEAEDAATYYCHQRSSYTFGGGTKLEIK

^^^^^ ^^^ ^^^^^^^

Best Alignment of Coltuximab CDR-H3 to a sequence from OAS

QVQLVQPGAEVVKPGASVKLSCKTSGYTFTSNWMHWVKQAPGQGLEWIGEIDPSDSYTNYNQNFQGKAKLTVDKSTSTAYMEVSSLRSDDTAVYYCARGSNPYYYAMDYWGQGTSVTVSS

.|||.|.|.|.||.|||||.|||.|||.||...||||||..|..|||||.|......|.|||.|.|||..|||.|.|||||...||.|.|.|||||||||||||||||||||||||||||

-VQLQQSGPELVKTGASVKISCKASGYSFTGYYMHWVKQSHGKSLEWIGYISCYNGATSYNQKFKGKATFTVDTSSSTAYMQSNSLTSEDSAVYYCARGSNPYYYAMDYWGQGTSVTVSS

^^^^^^^^ ^^^^^^^^ ^^^^^^^^^^^^^

Therapeutic : Concizumab

Best Alignment of Concizumab heavy chain to a sequence from OAS

EVQLVESGGGLVKPGGSLRLSCAASGFTFSNYAMSWVRQTPEKRLEWVATISRSGSYSYFPDSVQGRFTISRDNAKNSLYLQMNSLRAEDTAVYYCARLGGYDEGDAMDSWGQGTTVTVSS

||||||||||||||||||.|||||||||||.|||||||||||||||||||||..|||.|.||||.||||||||||||.|||||.|||.||||.|||||||||||.||||.|||||.|||||

EVQLVESGGGLVKPGGSLKLSCAASGFTFSSYAMSWVRQTPEKRLEWVATISSGGSYTYYPDSVKGRFTISRDNAKNTLYLQMSSLRSEDTAMYYCARLGGYDERDAMDYWGQGTSVTVSS

^^^^^^^^ ^^^^^^^^ ^^^^^^^^^^^^^^

Best Alignment of Concizumab light chain to a sequence from OAS

DIVMTQTPLSLSVTPGQPASISCKSSQSLLESDGKTYLNWYLQKPGQSPQLLIYLVSILDSGVPDRFSGSGSGTDFTLKISRVEAEDVGVYYCLQATHFPQTFGGGTKVEIK

||||||||||||||||||||||||||||||.|||||||.|||||||||||||||.||...|||||||||||||||||||||||||||||||||||.||.|.|||||||||||

DIVMTQTPLSLSVTPGQPASISCKSSQSLLHSDGKTYLCWYLQKPGQSPQLLIYEVSSRFSGVPDRFSGSGSGTDFTLKISRVEAEDVGVYYCLQGTHLPLTFGGGTKVEIK

^^^^^^^^^^^ ^^^ ^^^^^^^^^

Best Alignment of Concizumab heavy chain CDRs to a sequence from OAS

EVQLVESGGGLVKPGGSLRLSCAASGFTFSNYAMSWVRQTPEKRLEWVATISRSGSYSYFPDSVQGRFTISRDNAKNSLYLQMNSLRAEDTAVYYCARLGGYDEGDAMDSWGQGTTVTVSS

.|||..||||||||||||.|||||||||||.|||||||||||||||||||||..|||.|.||||.||||||||||||.|||||.|||.||||.|||||||||||.||||.|||||.|||||

-VQLKQSGGGLVKPGGSLKLSCAASGFTFSSYAMSWVRQTPEKRLEWVATISSGGSYTYYPDSVKGRFTISRDNAKNTLYLQMSSLRSEDTAMYYCARLGGYDERDAMDYWGQGTSVTVSS

^^^^^^^^ ^^^^^^^^ ^^^^^^^^^^^^^^

Best Alignment of Concizumab light chain CDRs to a sequence from OAS

DIVMTQTPLSLSVTPGQPASISCKSSQSLLESDGKTYLNWYLQKPGQSPQLLIYLVSILDSGVPDRFSGSGSGTDFTLKISRVEAEDVGVYYCLQATHFPQTFGGGTKVEIK

|.|||||||.||||.|||||||||||||||.|||||||||.||.||.||.|||||||.|||||||||.|||||||||||||||||||.||||||||||||||||||||.|||

DVVMTQTPLTLSVTIGQPASISCKSSQSLLDSDGKTYLNWLLQRPGESPKLLIYLVSKLDSGVPDRFTGSGSGTDFTLKISRVEAEDLGVYYCLQATHFPQTFGGGTKLEIK

^^^^^^^^^^^ ^^^ ^^^^^^^^^

Best Alignment of Concizumab CDR-H3 to a sequence from OAS

EVQLVESGGGLVKPGGSLRLSCAASGFTFSNYAMSWVRQTPEKRLEWVATISRSGSYSYFPDSVQGRFTISRDNAKNSLYLQMNSLRAEDTAVYYCARLGGYDEGDAMDSWGQGTTVTVSS

.|||..||..|||||.|...||.|||.||..|.|.||.|...|.|||...|..............|..|...|......|...|||..||.|||||||.||||||||||.|||||.|||||

-VQLQQSGPVLVKPGASVKMSCQASGYTFTDYYMNWVKQSHGKSLEWIGVINPYNGGTSYNQKFKGKATLTVDKSSSTAYMELNSLTSEDSAVYYCAREGGYDEGDAMDYWGQGTSVTVSS

^^^^^^^^ ^^^^^^^^ ^^^^^^^^^^^^^^

Therapeutic : Crenezumab

Best Alignment of Crenezumab heavy chain to a sequence from OAS

EVQLVESGGGLVQPGGSLRLSCAASGFTFSSYGMSWVRQAPGKGLELVASINSNGGSTYYPDSVKGRFTISRDNAKNSLYLQMNSLRAEDTAVYYCASG--------DYWGQGTTVTVSS

||||.|||||||||||||||||||||||||||.|||||||||||||.|..|...||||||.||||||||||||||||||||||||||||||||||||||........|||||||.|||||

EVQLLESGGGLVQPGGSLRLSCAASGFTFSSYAMSWVRQAPGKGLEWVSAISGSGGSTYYADSVKGRFTISRDNAKNSLYLQMNSLRAEDTAVYYCASGYNYGYLYFDYWGQGTLVTVSS

^^^^^^^^ ^^^^^^^^ ^^^^^^^^^^^^^

Best Alignment of Crenezumab light chain to a sequence from OAS

DIVMTQSPLSLPVTPGEPASISCRSSQSLVYSNGDTYLHWYLQKPGQSPQLLIYKVSNRFSGVPDRFSGSGSGTDFTLKISRVEAEDVGVYYCSQSTHVPWTFGQGTKVEIK

||||||||||||||||||||||||||||||.|||..||.|||||||||||||||.||||.|||||||||||||||||||||||||||||||||.|..|.|||||||||||||

DIVMTQSPLSLPVTPGEPASISCRSSQSLVRSNGNIYLDWYLQKPGQSPQLLIYLVSNRASGVPDRFSGSGSGTDFTLKISRVEAEDVGVYYCMQGIHLPWTFGQGTKVEIK

^^^^^^^^^^^ ^^^ ^^^^^^^^^

Best Alignment of Crenezumab heavy chain CDRs to a sequence from OAS

EVQLVESGGGLVQPGGSLRLSCAASGFTFSSYGMSWVRQAPGKGLELVASINSNGGSTYYPDSVKGRFTISRDNAKNSLYLQMNSLRAEDTAVYYCASGDYWGQGTTVTVSS

.|||..||||||||||||.|||||||||||||||||||..|...|||||.|||||||||||||||||.|||||||||.|||||.||..||||.|||||.||||||||.||||

-VQLKQSGGGLVQPGGSLKLSCAASGFTFSSYGMSWVRRTPDERLELVATINSNGGSTYYPDSVKGRSTISRDNAKNTLYLQMSSLKSEDTAMYYCASWDYWGQGTTLTVSS

^^^^^^^^ ^^^^^^^^ ^^^^^

Best Alignment of Crenezumab light chain CDRs to a sequence from OAS

DIVMTQSPLSLPVTPGEPASISCRSSQSLVYSNGDTYLHWYLQKPGQSPQLLIYKVSNRFSGVPDRFSGSGSGTDFTLKISRVEAEDVGVYYCSQSTHVPWTFGQGTKVEIK

|||.|||||||||..|..|||||.|||||||||||||||||||||||||.||||||||||||.||||||||||||||||||||||||.|||.||||||||||||.|||.|||

DIVITQSPLSLPVSLGDQASISCSSSQSLVYSNGDTYLHWYLQKPGQSPKLLIYKVSNRFSGAPDRFSGSGSGTDFTLKISRVEAEDLGVYVCSQSTHVPWTFGGGTKLEIK

^^^^^^^^^^^ ^^^ ^^^^^^^^^

Best Alignment of Crenezumab CDR-H3 to a sequence from OAS

EVQLVESGGGLVQPGGSLRLSCAASGFTFSSYGMSWVRQAPGKGLELVASINSNGGSTYYPDSVKGRFTISRDNAKNSLYLQMNSLRAEDTAVYYCASGDYWGQGTTVTVSS

||||..|...||.||.|....|.|||.||..|.|.||.|..||.||....||.|.|.|.|....||..|...|......|....||..||||||||||||||||||.|||||

EVQLQQSRPELVKPGASVKIPCKASGYTFTDYNMDWVNQSHGKSLEWIGDINPNNGGTIYNQKFKGKATLTVDKSSSTAYMELRSLTSEDTAVYYCASGDYWGQGTSVTVSS

^^^^^^^^ ^^^^^^^^ ^^^^^

Therapeutic : Crizanlizumab

Best Alignment of Crizanlizumab heavy chain to a sequence from OAS

QVQLVQSGAEVKKPGASVKVSCKVSGYTFTSYDINWVRQAPGKGLEWMGWIYPGDGSIKYNEKFKGRVTMTVDKSTDTAYMELSSLRSEDTAVYYCARRGEYGN--YEGAMDYWGQGTLVTVSS

|||||||||||||||||||||||.||||||||...|||||||.||||||.|.|..||..|..||.||||||.|.||.|||||||||||||||||||||.|.||.....||.|||||||||||||

QVQLVQSGAEVKKPGASVKVSCKASGYTFTSYYMHWVRQAPGQGLEWMGIINPSGGSTSYAQKFQGRVTMTRDTSTSTAYMELSSLRSEDTAVYYCARAGTYGITGTTGAFDYWGQGTLVTVSS

^^^^^^^^ ^^^^^^^^ ^^^^^^^^^^^^^^^^^

Best Alignment of Crizanlizumab light chain to a sequence from OAS

DIQMTQSPSSLSASVGDRVTITCKASQSVDYDGHSYMNWYQQKPGKAPKLLIYAASNLESGVPSRFSGSGSGTDFTLTISSLQPEDFATYYCQQSDENPLTFGGGTKVEIK

|||||||||||||||||||||||.||||......||.|||||||||||||||||||.|.|||||||||||||||||||||||||||||||||||||.||||||||||||||

DIQMTQSPSSLSASVGDRVTITCRASQSI----SSYLNWYQQKPGKAPKLLIYAASSLQSGVPSRFSGSGSGTDFTLTISSLQPEDFATYYCQQSDSNPLTFGGGTKVEIK

^^^^^^^^^^ ^^^ ^^^^^^^^^

Best Alignment of Crizanlizumab heavy chain CDRs to a sequence from OAS

QVQLVQSGAEVKKPGASVKVSCKVSGYTFTSYDINWVRQAPGKGLEWMGWIYPGDGSIKYNEKFKGRVTMTVDKSTDTAYMELSSLRSEDTAVYYCARRGEYGNYEGAMDYWGQGTLVTVSS

||||.|||.|..||||.||.|||.|||||||||||||.|.||.||||.|||||||||.||||||||..|.|.|||..||||.||||.||..|||.|||.||||||.||||||||||.|||||

QVQLQQSGPELVKPGALVKISCKASGYTFTSYDINWVKQRPGQGLEWIGWIYPGDGSTKYNEKFKGKATLTADKSSSTAYMQLSSLTSENSAVYFCARSGEYGNYGGAMDYWGQGTSVTVSS

^^^^^^^^ ^^^^^^^^ ^^^^^^^^^^^^^^^

Best Alignment of Crizanlizumab light chain CDRs to a sequence from OAS

DIQMTQSPSSLSASVGDRVTITCKASQSVDYDGHSYMNWYQQKPGKAPKLLIYAASNLESGVPSRFSGSGSGTDFTLTISSLQPEDFATYYCQQSDENPLTFGGGTKVEIK

||..||||.||..|.|.|.||.|||||||||||.||||||||||...|||||||||||.||.|.|||||||||||||.|.....||.|||.||||.|||.|||||||.|||

DIVLTQSPASLAVSLGQRATISCKASQSVDYDGDSYMNWYQQKPVQPPKLLIYAASNLGSGIPARFSGSGSGTDFTLNIHPVEEEDAATYCCQQSNENPYTFGGGTKLEIK

^^^^^^^^^^ ^^^ ^^^^^^^^^

Best Alignment of Crizanlizumab CDR-H3 to a sequence from OAS

QVQLVQSGAEVKKPGASVKVSCKVSGYTFTSYDINWVRQAPGKGLEWMGWIYPGDGSIKYNEKFKGRVTMTVDKSTDTAYMELSSLRSEDTAVYYCARRGEYGNYEGAMDYWGQGTLVTVSS

||||.|.|||...||.|||.|||.||||||||...||.|.|..||||.|.|.|.|....||.|.|...|.|||||..||||.||||.|||..|||||||||||||.||||||||||.|||||

QVQLKQPGAELVRPGSSVKLSCKASGYTFTSYWMHWVKQRPIQGLEWIGNIDPSDSETYYNQKIKDHATLTVDKSSSTAYMQLSSLTSEDSEVYYCARRGEYGNYGGAMDYWGQGTSVTVSS

^^^^^^^^ ^^^^^^^^ ^^^^^^^^^^^^^^^

Therapeutic : Dacetuzumab

Best Alignment of Dacetuzumab heavy chain to a sequence from OAS

EVQLVESGGGLVQPGGSLRLSCAASGYSFTGYYIHWVRQAPGKGLEWVARVIPNAGGTSYNQKFKGRFTLSVDNSKNTAYLQMNSLRAEDTAVYYCAREG-----IYWWGQGTLVTVSS

||||||||||||||||||||||||||..|..|..|||||||||||.||.|.......|||....|||||.|.||.|||.|||||||||||||||||||||.....|..|||||||||||

EVQLVESGGGLVQPGGSLRLSCAASGFTFSSYWMHWVRQAPGKGLVWVSRINSDGSSTSYADSVKGRFTISRDNAKNTLYLQMNSLRAEDTAVYYCAREGYYDNYIDYWGQGTLVTVSS

^^^^^^^^ ^^^^^^^^ ^^^^^^^^^^^^

Best Alignment of Dacetuzumab light chain to a sequence from OAS

DIQMTQSPSSLSASVGDRVTITCRSSQSLVHSNGNTFLHWYQQKPGKAPKLLIYTVSNRFSGVPSRFSGSGSGTDFTLTISSLQPEDFATYFCSQTTHVPWTFGQGTKVEIK

||||||||||||||||||||||||.|||.......|||||||||||||||||||..||..|||||||||||||||||||||||||||||||.|.|....|.|||||||||||

DIQMTQSPSSLSASVGDRVTITCRASQSI-----STFLHWYQQKPGKAPKLLIYAASNLQSGVPSRFSGSGSGTDFTLTISSLQPEDFATYYCQQIYSTPKTFGQGTKVEIK

^^^^^^^^^^^ ^^^ ^^^^^^^^^

Best Alignment of Dacetuzumab heavy chain CDRs to a sequence from OAS

EVQLVESGGGLVQPGGSLRLSCAASGYSFTGYYIHWVRQAPGKGLEWVARVIPNAGGTSYNQKFKGRFTLSVDNSKNTAYLQMNSLRAEDTAVYYCAREGIYWWGQGTLVTVSS

||.|.|||..||.||.|...||.||||||||||.|||.|..||.|||..||.||.|||||||||||...|.||.|..|||....||..||.|||||||||.|.|||||..||||

EVKLMESGPDLVKPGASVKISCKASGYSFTGYYMHWVKQSHGKSLEWIGRVNPNNGGTSYNQKFKGKAILTVDKSSSTAYMELRSLTSEDSAVYYCAREGYYGWGQGTTLTVSS

^^^^^^^^ ^^^^^^^^ ^^^^^^^

Best Alignment of Dacetuzumab light chain CDRs to a sequence from OAS

DIQMTQSPSSLSASVGDRVTITCRSSQSLVHSNGNTFLHWYQQKPGKAPKLLIYTVSNRFSGVPSRFSGSGSGTDFTLTISSLQPEDFATYFCSQTTHVPWTFGQGTKVEIK

|..|||.|.||..|.||...|.||||||||||||||.||||.||||..||||||||||||||||.|||||||||||||.||....||...|||||.||||||||.|||.|||

DVVMTQTPLSLPVSLGDQASISCRSSQSLVHSNGNTYLHWYLQKPGQSPKLLIYTVSNRFSGVPDRFSGSGSGTDFTLKISRVEAEDLGVYFCSQSTHVPWTFGGGTKLEIK

^^^^^^^^^^^ ^^^ ^^^^^^^^^

Best Alignment of Dacetuzumab CDR-H3 to a sequence from OAS

EVQLVESGGGLVQPGGSLRLSCAASGYSFTGYYIHWVRQAPGKGLEWVARVIPNAGGTSYNQKFKGRFTLSVDNSKNTAYLQMNSLRAEDTAVYYCAREGIYWWGQGTLVTVSS

||.|||||||||.|||||.|||||||..|..|...||||.|.|.|||||......|.|.|....|||||.|.||..|..||||.|||.||||.|||||||||.|||.|..||||

EVMLVESGGGLVKPGGSLKLSCAASGFTFSSYAMSWVRQTPEKRLEWVASISSG-GSTYYPDSVKGRFTISRDNARNILYLQMSSLRSEDTAMYYCAREGIYYWGQCTTLTVSS

^^^^^^^^ ^^^^^^^^ ^^^^^^^

Therapeutic : Daclizumab

Best Alignment of Daclizumab heavy chain to a sequence from OAS

QVQLVQSGAEVKKPGSSVKVSCKASGYTFTSYRMHWVRQAPGQGLEWIGYINPSTGYTEYNQKFKDKATITADESTNTAYMELSSLRSEDTAVYYCARGGG---VFDYWGQGTLVTVSS

|||||||||||||||.||||||||||||||||.||||||||||||||.|.||||.|.|.|.|||.|..||||||||.|||||||||||||||||||||.||....||||||||||||||

QVQLVQSGAEVKKPGASVKVSCKASGYTFTSYYMHWVRQAPGQGLEWMGIINPSGGSTSYAQKFQDRVTITADESTSTAYMELSSLRSEDTAVYYCARDGGSGWDFDYWGQGTLVTVSS

^^^^^^^^ ^^^^^^^^ ^^^^^^^^^^^^

Best Alignment of Daclizumab light chain to a sequence from OAS

DIQMTQSPSTLSASVGDRVTITCSASSSI-SYMHWYQQKPGKAPKLLIYTTSNLASGVPARFSGSGSGTEFTLTISSLQPDDFATYYCHQRSTYPLTFGQGTKVEVK

|||||||||||||||||||||||.||.||.|...|||||||||||||||..|||.||||.||||||||||||||||||||||||||||.|.||||.|||||||||||

DIQMTQSPSTLSASVGDRVTITCRASQSISSWLAWYQQKPGKAPKLLIYKASNLESGVPSRFSGSGSGTEFTLTISSLQPDDFATYYCQQYSTYPWTFGQGTKVEVK

^^^^^^ ^^^ ^^^^^^^^^

Best Alignment of Daclizumab heavy chain CDRs to a sequence from OAS

QVQLVQSGAEVKKPGSSVKVSCKASGYTFTSYRMHWVRQAPGQGLEWIGYINPSTGYTEYNQKFKDKATITADESTNTAYMELSSLRSEDTAVYYCARGGGVFDYWGQGTLVTVSS

.|.|..||||..|||.|||.|||||||||||||||||.|.|||||||||||||||||||||||||||||.|||.|..||||.||||.|||.||||||||||.|.|||||||||||.

-VKLMESGAELAKPGASVKMSCKASGYTFTSYRMHWVKQRPGQGLEWIGYINPSTGYTEYNQKFKDKATLTADKSSSTAYMQLSSLTSEDSAVYYCARGGGWFAYWGQGTLVTVSA

^^^^^^^^ ^^^^^^^^ ^^^^^^^^^

Best Alignment of Daclizumab light chain CDRs to a sequence from OAS

DIQMTQSPSTLSASVGDRVTITCSASSSISYMHWYQQKPGKAPKLLIYTTSNLASGVPARFSGSGSGTEFTLTISSLQPDDFATYYCHQRSTYPLTFGQGTKVEVK

.|..|||....|||.|..||.||.||||||||||||||||..|...||.||.||||||||||||||||...|||||....|.|||||||||.||||||.|||.|.|

QIVLTQSRAIMSASPGEKVTMTCRASSSISYMHWYQQKPGTSPQSWIYYTSKLASGVPARFSGSGSGTAYSLTISSMEAEDAATYYCHQRSNYPLTFGGGTKLEIK

^^^^^ ^^^ ^^^^^^^^^

Best Alignment of Daclizumab CDR-H3 to a sequence from OAS

QVQLVQSGAEVKKPGSSVKVSCKASGYTFTSYRMHWVRQAPGQGLEWIGYINPSTGYTEYNQKFKDKATITADESTNTAYMELSSLRSEDTAVYYCARGGGVFDYWGQGTLVTVSS

.............|.......|..||....||...|.||.||.||||||||..|.|.|.||...|...||..|.|.|.....|||....|||||||||||||||||||||||||.|

-------------PSETLSLTCTVSGGSISSYYWSWIRQPPGKGLEWIGYIYYS-GSTNYNPSLKSRVTISVDTSKNQFSLKLSSVTAADTAVYYCARGGGVFDYWGQGTLVTVYS

^^^^^^^^ ^^^^^^^^ ^^^^^^^^^

Therapeutic : Dalotuzumab

Best Alignment of Dalotuzumab heavy chain to a sequence from OAS

QVQLQESGPGLVKPSETLSLTCTVSGYSITGGYLWNWIRQPPGKGLEWIGYISYDGTNNYKPSLKDRVTISRDTSKNQFSLKLSSVTAADTAVYYCARYGR-------VFFDYWGQGTLVTVSS

||||||||||||||||||||||||||.||...|.|.||||||||||||||||.|.|..||.||||.|||||.||||||||||||||||||||||||||.||.......||||||||||||||||

QVQLQESGPGLVKPSETLSLTCTVSGGSI-SSYYWSWIRQPPGKGLEWIGYIYYSGSTNYNPSLKSRVTISVDTSKNQFSLKLSSVTAADTAVYYCARGGRYYDSSSAVFFDYWGQGTLVTVSS

^^^^^^^^^ ^^^^^^^ ^^^^^^^^^^^^^^^^^

Best Alignment of Dalotuzumab light chain to a sequence from OAS

DIVMTQSPLSLPVTPGEPASISCRSSQSIVHSNGNTYLQWYLQKPGQSPQLLIYKVSNRLYGVPDRFSGSGSGTDFTLKISRVEAEDVGVYYCFQGSHVPWTFGQGTKVEIK

||||||||||||||||||||||||||||.|.||||.||.|||||||||||||||.||||..||||||||||||||||||||||||||||||||.||.|.|||||||||||||

DIVMTQSPLSLPVTPGEPASISCRSSQSLVRSNGNIYLDWYLQKPGQSPQLLIYLVSNRASGVPDRFSGSGSGTDFTLKISRVEAEDVGVYYCMQGIHLPWTFGQGTKVEIK

^^^^^^^^^^^ ^^^ ^^^^^^^^^

Best Alignment of Dalotuzumab heavy chain CDRs to a sequence from OAS

QVQLQESGPGLVKPSETLSLTCTVSGYSITGGYLWNWIRQPPGKGLEWIGYISYDGTNNYKPSLKDRVTISRDTSKNQFSLKLSSVTAADTAVYYCARYGRVFFDYWGQGTLVTVSS

.||||||||||||||..|||||.|.|||||.||.||||||.||..|||.|||||||.|||.||||.|..|.||||||||.|||.|||..|||.||||||.|.|||||||||..||||

EVQLQESGPGLVKPSQSLSLTCSVTGYSITSGYYWNWIRQFPGNKLEWMGYISYDGSNNYNPSLKNRISITRDTSKNQFFLKLNSVTTEDTATYYCARYYRPFFDYWGQGTTLTVSS

^^^^^^^^^ ^^^^^^^ ^^^^^^^^^^

Best Alignment of Dalotuzumab light chain CDRs to a sequence from OAS

DIVMTQSPLSLPVTPGEPASISCRSSQSIVHSNGNTYLQWYLQKPGQSPQLLIYKVSNRLYGVPDRFSGSGSGTDFTLKISRVEAEDVGVYYCFQGSHVPWTFGQGTKVEIK

|..|||.||||||..|..||||||||||||||||||||.||||||||||.||||||||||.||||||||||||||||||||||||||.||||||||||||||||.|||.|||

DVLMTQTPLSLPVSLGDHASISCRSSQSIVHSNGNTYLEWYLQKPGQSPKLLIYKVSNRLSGVPDRFSGSGSGTDFTLKISRVEAEDLGVYYCFQGSHVPWTFGGGTKLEIK

^^^^^^^^^^^ ^^^ ^^^^^^^^^

Best Alignment of Dalotuzumab CDR-H3 to a sequence from OAS

QVQLQESGPGLVKPSETLSLTCTVSGYSITGGYLWNWIRQPPGKGLEWIGYISYD-GTNNYKPSLKDRVTISRDTSKNQFSLKLSSVTAADTAVYYCARYGRVFFDYWGQGTLVTVSS

.||||.|||.||||.......|..|||.....|..||..|..||.|||||.|....|...|....|...|...|.|.......|.|.|..|.|||||||||||.||||||||..||||

EVQLQQSGPVLVKPGASVKMSCKASGYTF-TDYYMNWVKQSHGKSLEWIGVINPYNGGTSYNQKFKGKATLTVDKSSSTAYMELNSLTSEDSAVYYCARYGRVYFDYWGQGTTLTVSS

^^^^^^^^^ ^^^^^^^^ ^^^^^^^^^^

Therapeutic : Daratumumab

Best Alignment of Daratumumab heavy chain to a sequence from OAS

EVQLLESGGGLVQPGGSLRLSCAVSGFTFNSFAMSWVRQAPGKGLEWVSAISGSGGGTYYADSVKGRFTISRDNSKNTLYLQMNSLRAEDTAVYFCAKDKILWFGEPVFDYWGQGTLVTVSS

|||||||||||||||||||||||.|||||.|.||||||||||||||||||||||||.|||||||||||||||||||||||||||||||||||||.||||..|||||..||||||||||||||

EVQLLESGGGLVQPGGSLRLSCAASGFTFSSYAMSWVRQAPGKGLEWVSAISGSGGSTYYADSVKGRFTISRDNSKNTLYLQMNSLRAEDTAVYYCAKDASLWFGEGYFDYWGQGTLVTVSS

^^^^^^^^ ^^^^^^^^ ^^^^^^^^^^^^^^^

Best Alignment of Daratumumab light chain to a sequence from OAS

EIVLTQSPATLSLSPGERATLSCRASQSVSSYLAWYQQKPGQAPRLLIYDASNRATGIPARFSGSGSGTDFTLTISSLEPEDFAVYYCQQRSNWPPTFGQGTKVEIK

|||||||||||||||||||||||||||||||||||||||||||||||||||||||||||||||||||||||||||||||||||||||||||||||||||||||||||

EIVLTQSPATLSLSPGERATLSCRASQSVSSYLAWYQQKPGQAPRLLIYDASNRATGIPARFSGSGSGTDFTLTISSLEPEDFAVYYCQQRSNWPPTFGQGTKVEIK

^^^^^^ ^^^ ^^^^^^^^^

Best Alignment of Daratumumab heavy chain CDRs to a sequence from OAS

EVQLLESGGGLVQPGGSLRLSCAVSGFTFNSFAMSWVRQAPGKGLEWVSAISGSGGGTYYADSVKGRFTISRDNSKNTLYLQMNSLRAEDTAVYFCAKDKILWFGEPVFDYWGQGTLVTVSS

..............|||||||||.|||||.|.||||||||||||||||||||||||.|||||||||||||||||||||||||||||||||||||.|||||.||||||.||||||||||||||

--------------GGSLRLSCAASGFTFSSYAMSWVRQAPGKGLEWVSAISGSGGSTYYADSVKGRFTISRDNSKNTLYLQMNSLRAEDTAVYYCAKDKELWFGEPYFDYWGQGTLVTVSS

^^^^^^^^ ^^^^^^^^ ^^^^^^^^^^^^^^^

Best Alignment of Daratumumab light chain CDRs to a sequence from OAS

EIVLTQSPATLSLSPGERATLSCRASQSVSSYLAWYQQKPGQAPRLLIYDASNRATGIPARFSGSGSGTDFTLTISSLEPEDFAVYYCQQRSNWPPTFGQGTKVEIK

|||||||||||||||||||||||||.|||||||||||||||||||||.|||||||||||.|||||||||||||||.||||||.|.||||||||||||.||||..|||

EIVLTQSPATLSLSPGERATLSCRARQSVSSYLAWYQQKPGQAPRLLMYDASNRATGIPGRFSGSGSGTDFTLTINSLEPEDSAIYYCQQRSNWPPTLGQGTRLEIK

^^^^^^ ^^^ ^^^^^^^^^

Best Alignment of Daratumumab CDR-H3 to a sequence from OAS

EVQLLESGGGLVQPGGSLRLSCAVSGFTFNSFAMSWVRQAPGKGLEWVSAISGSGGGTYYADSVKGRFTISRDNSKNTLYLQMNSLRAEDTAVYFCAKDKILWFGEPVFDYWGQGTLVTVSS

..............|||||||||.|||||...|||||||.|||||||||||.|||..||||||||.||||||||||||||||||||||||||.|.|||||.||||||.||||||||||||||

--------------GGSLRLSCAASGFTFSNYAMSWVRQNPGKGLEWVSAIRGSGRRTYYADSVKARFTISRDNSKNTLYLQMNSLRAEDTAIYYCAKDKELWFGEPYFDYWGQGTLVTVSS

^^^^^^^^ ^^^^^^^^ ^^^^^^^^^^^^^^^

Therapeutic : Dectrekumab

Best Alignment of Dectrekumab heavy chain to a sequence from OAS

EVQLVESGGGVVQPGRSLRLSCAASGFTFSSYGMHWVRQAPGKGLEWVAIIWYDGSNKYYADSVKGRFTISRDNSKNTLYLQMNSLRAEDTAVYYCARLWFGD---LDAFDIWGQGTMVTVSS

.||||||||||||||||||||||||||||||||||||||||||||||||.||||||||||||||||||||||||||||||||||||||||||||||||.|.|.....||||||||||||||||

-VQLVESGGGVVQPGRSLRLSCAASGFTFSSYGMHWVRQAPGKGLEWVAVIWYDGSNKYYADSVKGRFTISRDNSKNTLYLQMNSLRAEDTAVYYCARDWGGRVATQDAFDIWGQGTMVTVSS

^^^^^^^^ ^^^^^^^^ ^^^^^^^^^^^^^^^^

Best Alignment of Dectrekumab light chain to a sequence from OAS

EIVLTQSPATLSLSPGERAILSCRAGQSVSSYLVWYQQKPGQAPRLLIYDASNRATGIPARFSGSGSGTDFTLTISSLEPEDFAVYYCQQRSSWPPVYTFGQGTKLEIK

|||||||||||||||||||.|||||.|||||||.||||||||||||||||||||||||||||||||||||||||||||||||||||||||||.||||||||||||||||

EIVLTQSPATLSLSPGERATLSCRASQSVSSYLAWYQQKPGQAPRLLIYDASNRATGIPARFSGSGSGTDFTLTISSLEPEDFAVYYCQQRSNWPPVYTFGQGTKLEIK

^^^^^^ ^^^ ^^^^^^^^^^^

Best Alignment of Dectrekumab heavy chain CDRs to a sequence from OAS

EVQLVESGGGVVQPGRSLRLSCAASGFTFSSYGMHWVRQAPGKGLEWVAIIWYDGSNKYYADSVKGRFTISRDNSKNTLYLQMNSLRAEDTAVYYCARLWFGDLDAFDIWGQGTMVTVSS

..............|.|||||||||||||||||||||||||||||||||.||||||||||||||||||||||||||||||||||||||||||||||||||||..||||||||||||||||

--------------GGSLRLSCAASGFTFSSYGMHWVRQAPGKGLEWVAVIWYDGSNKYYADSVKGRFTISRDNSKNTLYLQMNSLRAEDTAVYYCARLWFGEPDAFDIWGQGTMVTVSS

^^^^^^^^ ^^^^^^^^ ^^^^^^^^^^^^^

Best Alignment of Dectrekumab light chain CDRs to a sequence from OAS

EIVLTQSPATLSLSPGERAILSCRAGQSVSSYLVWYQQKPGQAPRLLIYDASNRATGIPARFSGSGSGTDFTLTISSLEPEDFAVYYCQQRSSWPPVYTFGQGTKLEIK

|||||||||||||||||||.|||||.|||||||.||||||||||||||||||||||||||||||||||||||||||||||||||||||||||.||||||||||||||||

EIVLTQSPATLSLSPGERATLSCRASQSVSSYLAWYQQKPGQAPRLLIYDASNRATGIPARFSGSGSGTDFTLTISSLEPEDFAVYYCQQRSNWPPVYTFGQGTKLEIK

^^^^^^ ^^^ ^^^^^^^^^^^

Best Alignment of Dectrekumab CDR-H3 to a sequence from OAS

EVQLVESGGGVVQPGRSLRLSCAASGFTFSSYGMHWVRQAPGKGLEWVAIIWYDGSNKYYADSVKGRFTISRDNSKNTLYLQMNSLRAEDTAVYYCARLWFGDLDAFDIWGQGTMVTVSS

..............|.||||||||||||||||||.|||||||||||||..|...|...||||||||||||||||.||.||||||||||||||||||||||||||||||||||||||||||

------------KSGGSLRLSCAASGFTFSSYGMSWVRQAPGKGLEWVSSISSSGGYIYYADSVKGRFTISRDNAKNSLYLQMNSLRAEDTAVYYCARLWFGDLDAFDIWGQGTMVTVSS

^^^^^^^^ ^^^^^^^^ ^^^^^^^^^^^^^

Therapeutic : Denosumab

Best Alignment of Denosumab heavy chain to a sequence from OAS

EVQLLESGGGLVQPGGSLRLSCAASGFTFSSYAMSWVRQAPGKGLEWVSGITGSGGSTYYADSVKGRFTISRDNSKNTLYLQMNSLRAEDTAVYYCAKDPGTTV----------------IMSWFDPWGQGTLVTVSS

|||||||||||||||||||||||||||||||||||||||||||||||||.|.||||||||||||||||||||||||||||||||||||||||||||||||.||...................||||||||||||||||

EVQLLESGGGLVQPGGSLRLSCAASGFTFSSYAMSWVRQAPGKGLEWVSAISGSGGSTYYADSVKGRFTISRDNSKNTLYLQMNSLRAEDTAVYYCAKDPFTTPLSRLSTIAVAGMLGDLAESWFDPWGQGTLVTVSS

^^^^^^^^ ^^^^^^^^ ^^^^^^^^^^^^^^^^^^^^^^^^^^^^^^^

Best Alignment of Denosumab light chain to a sequence from OAS

EIVLTQSPGTLSLSPGERATLSCRASQSVRGRYLAWYQQKPGQAPRLLIYGASSRATGIPDRFSGSGSGTDFTLTISRLEPEDFAVFYCQQYGSSPRTFGQGTKVEIK

|||||||||||||||||||||||||||||.||||||||||||||||||||||||||||||||||||||||||||||||||||||||.|||||||||||||||||||||

EIVLTQSPGTLSLSPGERATLSCRASQSV-GRYLAWYQQKPGQAPRLLIYGASSRATGIPDRFSGSGSGTDFTLTISRLEPEDFAVYYCQQYGSSPRTFGQGTKVEIK

^^^^^^^ ^^^ ^^^^^^^^^

Best Alignment of Denosumab heavy chain CDRs to a sequence from OAS

EVQLLESGGGLVQPGGSLRLSCAASGFTFSSYAMSWVRQAPGKGLEWVSGITGSGGSTYYADSVKGRFTISRDNSKNTLYLQMNSLRAEDTAVYYCAKDPGTTVIMSWFDPWGQGTLVTVSS

..............|.||..|||||||||||||||||||||||||||||.|.||||||||||||||||||||||||||||||||||||||||||||||||||||...|||||||||||||||

--------------GESLKISCAASGFTFSSYAMSWVRQAPGKGLEWVSAISGSGGSTYYADSVKGRFTISRDNSKNTLYLQMNSLRAEDTAVYYCAKDPGTTVTTGWFDPWGQGTLVTVSS

^^^^^^^^ ^^^^^^^^ ^^^^^^^^^^^^^^^

Best Alignment of Denosumab light chain CDRs to a sequence from OAS

EIVLTQSPGTLSLSPGERATLSCRASQSVRGRYLAWYQQKPGQAPRLLIYGASSRATGIPDRFSGSGSGTDFTLTISRLEPEDFAVFYCQQYGSSPRTFGQGTKVEIK

||||||||||||||||||||.|.|||||||||||||||||||||||||||||||||||||||||||||||||||||||||||||||.|||||||||||||||||||||

EIVLTQSPGTLSLSPGERATFSGRASQSVRGRYLAWYQQKPGQAPRLLIYGASSRATGIPDRFSGSGSGTDFTLTISRLEPEDFAVYYCQQYGSSPRTFGQGTKVEIK

^^^^^^^ ^^^ ^^^^^^^^^

Best Alignment of Denosumab CDR-H3 to a sequence from OAS

EVQLLESGGGLVQPGGSLRLSCAASGFTFSSYAMSWVRQAPGKGLEWVSGITGSGGSTYYADSVKGRFTISRDNSKNTLYLQMNSLRAEDTAVYYCAKDPGTTVIMSWFDPWGQGTLVTVSS

..............|.||..|||||||||||||||||||||||||||||.|.||||||||||||||||||||||||||||||||||||||||||||||||||||...|||||||||||||||

--------------GESLKISCAASGFTFSSYAMSWVRQAPGKGLEWVSAISGSGGSTYYADSVKGRFTISRDNSKNTLYLQMNSLRAEDTAVYYCAKDPGTTVTTGWFDPWGQGTLVTVSS

^^^^^^^^ ^^^^^^^^ ^^^^^^^^^^^^^^^

Therapeutic : Depatuxizumab

Best Alignment of Depatuxizumab heavy chain to a sequence from OAS

QVQLQESGPGLVKPSQTLSLTCTVSGYSIS-SDFAWNWIRQPPGKGLEWMGYISYSGNTRYQPSLKSRITISRDTSKNQFFLKLNSVTAADTATYYCVTAGR-----------GFPYWGQGTLVTVSS

||||||||||||||||||||||||||.|||..|..|.||||||||||||.|||.|||.|.|.||||||.|||.|||||||.||||||||||||.||||.||.............|.||||||||||||

QVQLQESGPGLVKPSQTLSLTCTVSGGSISSGDYYWSWIRQPPGKGLEWIGYIYYSGSTYYNPSLKSRVTISIDTSKNQFSLKLNSVTAADTAVYYCVRAGVENFYDSSASGGCFDYWGQGTLVTVSS

^^^^^^^^^^ ^^^^^^^ ^^^^^^^^^^^^^^^^^^^^

Best Alignment of Depatuxizumab light chain to a sequence from OAS

DIQMTQSPSSMSVSVGDRVTITCHSSQDINSNIGWLQQKPGKSFKGLIYHGTNLDDGVPSRFSGSGSGTDYTLTISSLQPEDFATYYCVQYAQFPWTFGGGTKLEIK

||.|||||||||||.||.|.||||.||||.||||||||||||||||||||||||.|||||||||||||.||.||||||..||||.||||||||||||||||||||||

DILMTQSPSSMSVSLGDTVSITCHASQDISSNIGWLQQKPGKSFKGLIYHGTNLEDGVPSRFSGSGSGADYSLTISSLESEDFADYYCVQYAQFPWTFGGGTKLEIK

^^^^^^ ^^^ ^^^^^^^^^

Best Alignment of Depatuxizumab heavy chain CDRs to a sequence from OAS

QVQLQESGPGLVKPSQTLSLTCTVSGYSISSDFAWNWIRQPPGKGLEWMGYISYSGNTRYQPSLKSRITISRDTSKNQFFLKLNSVTAADTATYYCVTAGRGFPYWGQGTLVTVSS

.||||.||||||||||.|||||||.||||.||.|||||.|.||..|||||||||||.|.|.|||||||.|.||||||||||.|||||..|||||||.|.||||.|||||||||||.

EVQLQQSGPGLVKPSQSLSLTCTVTGYSITSDYAWNWIWQFPGNKLEWMGYISYSGSTSYNPSLKSRISITRDTSKNQFFLQLNSVTTEDTATYYCATLGRGFAYWGQGTLVTVSA

^^^^^^^^^ ^^^^^^^ ^^^^^^^^^

Best Alignment of Depatuxizumab light chain CDRs to a sequence from OAS

DIQMTQSPSSMSVSVGDRVTITCHSSQDINSNIGWLQQKPGKSFKGLIYHGTNLDDGVPSRFSGSGSGTDYTLTISSLQPEDFATYYCVQYAQFPWTFGGGTKLEIK

||.|.|.|||||||..|...||.|.||.||||||||||||||||||||||||||.|||||||||||||.||.||||||..||||.||||||||||||||||||||||

DILMSQPPSSMSVSLEDTASITFHASQGINSNIGWLQQKPGKSFKGLIYHGTNLEDGVPSRFSGSGSGADYSLTISSLESEDFADYYCVQYAQFPWTFGGGTKLEIK

^^^^^^ ^^^ ^^^^^^^^^

Best Alignment of Depatuxizumab CDR-H3 to a sequence from OAS

QVQLQESGPGLVKPSQTLSLTCTVSGYSISSDFAWNWIRQPPGKGLEWMGYISYSGNTRYQPSLKSRITISRDTSKNQFFLKLNSVTAADTATYYCVTAGRGFPYWGQGTLVTVSS

.||||||||.||||||.|||||||.||||.|...|.||||.||..|||||||.|.|||.|.|||||||.|.||||.|||||.|||||..|||||||.|||||||||.||||||||.

EVQLQESGPDLVKPSQSLSLTCTVTGYSITSGYSWHWIRQFPGNKLEWMGYIHYGGNTNYNPSLKSRISITRDTSRNQFFLQLNSVTTEDTATYYCATAGRGFPYWCQGTLVTVSA

^^^^^^^^^ ^^^^^^^ ^^^^^^^^^

Therapeutic : Derlotuximab

Best Alignment of Derlotuximab heavy chain to a sequence from OAS

QVQLKESGPGLVAPSQSLSITCTVSGFSLTDYGVRWIRQPPGKGLEWLGVIWGGGSTYYNSALKSRLSISKDNSKSQVFLKMNSLQTDDTAMYYCAKEKRRGYYYAMDYWGQGTSVTVSS

||||||||||||||||||||||||||||||||||.||||||||||||||||||||||||||||||||||||||||||||||||||||||||||||||..|||.|||||||||||||||||

QVQLKESGPGLVAPSQSLSITCTVSGFSLTDYGVSWIRQPPGKGLEWLGVIWGGGSTYYNSALKSRLSISKDNSKSQVFLKMNSLQTDDTAMYYCAKHVRRGDYYAMDYWGQGTSVTVSS

^^^^^^^^ ^^^^^^^ ^^^^^^^^^^^^^^

Best Alignment of Derlotuximab light chain to a sequence from OAS

ENVLTQSPAIMSASPGEKVTMTCRASSSVSSSYLHWYQQKSGASPKLWIYSTSNLASGVPARFSGSGSGTSYSLTISSVEAEDAATYYCQQYSGYPLTFGGGTKLEIK

||||||||||||||||||||||||||||||||||||||||||||||||||||||||||||||||||||||||||||||||||||||||||||||||||||||||||||

ENVLTQSPAIMSASPGEKVTMTCRASSSVSSSYLHWYQQKSGASPKLWIYSTSNLASGVPARFSGSGSGTSYSLTISSVEAEDAATYYCQQYSGYPLTFGGGTKLEIK

^^^^^^^ ^^^ ^^^^^^^^^

Best Alignment of Derlotuximab heavy chain CDRs to a sequence from OAS

QVQLKESGPGLVAPSQSLSITCTVSGFSLTDYGVRWIRQPPGKGLEWLGVIWGGGSTYYNSALKSRLSISKDNSKSQVFLKMNSLQTDDTAMYYCAKEKRRGYYYAMDYWGQGTSVTVSS

.|||..||||||||||||||||||||||||.|||.|.||||||||||||||||.|||.|.|||.||||.||||||||||||.|||||||||.||||||.|||||||||||||||||||||

-VQLQQSGPGLVAPSQSLSITCTVSGFSLTSYGVSWVRQPPGKGLEWLGVIWGDGSTNYHSALISRLSFSKDNSKSQVFLKLNSLQTDDTATYYCAKEVRRGYYYAMDYWGQGTSVTVSS

^^^^^^^^ ^^^^^^^ ^^^^^^^^^^^^^^

Best Alignment of Derlotuximab light chain CDRs to a sequence from OAS

ENVLTQSPAIMSASPGEKVTMTCRASSSVSSSYLHWYQQKSGASPKLWIYSTSNLASGVPARFSGSGSGTSYSLTISSVEAEDAATYYCQQYSGYPLTFGGGTKLEIK

|||.|.||||||||||||||||||||||||||||||||..||||||||||||||||||||||||||||||||||||||||||||||||||||||||||||.|||||||

ENVHTPSPAIMSASPGEKVTMTCRASSSVSSSYLHWYQENSGASPKLWIYSTSNLASGVPARFSGSGSGTSYSLTISSVEAEDAATYYCQQYSGYPLTFGSGTKLEIK

^^^^^^^ ^^^ ^^^^^^^^^

Best Alignment of Derlotuximab CDR-H3 to a sequence from OAS

QVQLKESGPGLVAPSQSLSITCTVSGFSLTDYGVRWIRQPPGKGLEWLGVIWGGGSTYYNSALKSRLSISKDNSKSQVFLKMNSLQTDDTAMYYCAKEKRRGYYYAMDYWGQGTSVTVSS

.|||..||||||||||||||||||||||||.|||.|.||||||||||||||||.|||.|.|||.||||.||||||||||||.|||||||||.||||||.|||||||||||||||||||||

-VQLQQSGPGLVAPSQSLSITCTVSGFSLTSYGVSWVRQPPGKGLEWLGVIWGDGSTNYHSALISRLSFSKDNSKSQVFLKLNSLQTDDTATYYCAKEVRRGYYYAMDYWGQGTSVTVSS

^^^^^^^^ ^^^^^^^ ^^^^^^^^^^^^^^

Therapeutic : Dinutuximab

Best Alignment of Dinutuximab heavy chain to a sequence from OAS

EVQLLQSGPELEKPGASVMISCKASGSSFTGYNMNWVRQNIGKSLEWIGAIDPYYGGTSYNQKFKGRATLTVDKSSSTAYMHLKSLTSEDSAVYYCVSG-------MEYWGQGTSVTVSS

||||.|||||||||||||.|||||||.||||||||||.|..||||||||.||||||||||||||||.||||||||||||||.||||||||||||||.||.......|.||||||||||||

EVQLQQSGPELEKPGASVKISCKASGYSFTGYNMNWVKQSNGKSLEWIGNIDPYYGGTSYNQKFKGKATLTVDKSSSTAYMQLKSLTSEDSAVYYCASGGKPDYYAMDYWGQGTSVTVSS

^^^^^^^^ ^^^^^^^^ ^^^^^^^^^^^^^

Best Alignment of Dinutuximab light chain to a sequence from OAS

EIVMTQSPATLSVSPGERATLSCRSSQSLVHRNGNTYLHWYLQKPGQSPKLLIHKVSNRFSGVPDRFSGSGSGTDFTLKISRVEAEDLGVYFCSQSTHVPPLTFGAGTKLELK

.|||||||..|.||.|..|..||||||||||.|||||||||||||||||||||.|||||||||||||||||||||||||||||||||||||||||||||||||||||||||||

DIVMTQSPLSLPVSLGDQASISCRSSQSLVHSNGNTYLHWYLQKPGQSPKLLIYKVSNRFSGVPDRFSGSGSGTDFTLKISRVEAEDLGVYFCSQSTHVPPLTFGAGTKLELK

^^^^^^^^^^^ ^^^ ^^^^^^^^^^

Best Alignment of Dinutuximab heavy chain CDRs to a sequence from OAS

EVQLLQSGPELEKPGASVMISCKASGSSFTGYNMNWVRQNIGKSLEWIGAIDPYYGGTSYNQKFKGRATLTVDKSSSTAYMHLKSLTSEDSAVYYCVSGMEYWGQGTSVTVSS

||||.|||||||||||||.|||||||.||||||||||.|..||||||||.||||||||||||||||.||||||||||||||.|||||||||||||||.||.||||||||||.|

EVQLQQSGPELEKPGASVKISCKASGYSFTGYNMNWVKQSNGKSLEWIGNIDPYYGGTSYNQKFKGKATLTVDKSSSTAYMQLKSLTSEDSAVYYCVMGMDYWGQGTSVTVAS

^^^^^^^^ ^^^^^^^^ ^^^^^^

Best Alignment of Dinutuximab light chain CDRs to a sequence from OAS

EIVMTQSPATLSVSPGERATLSCRSSQSLVHRNGNTYLHWYLQKPGQSPKLLIHKVSNRFSGVPDRFSGSGSGTDFTLKISRVEAEDLGVYFCSQSTHVPPLTFGAGTKLELK

..||||.|..|.||.|..|..||||||||||.|||||||||||||||||||||.|||||||||||||||||||||||||||||||||||||||||||||||||||.|||||.|

DVVMTQTPLSLPVSLGDQASISCRSSQSLVHSNGNTYLHWYLQKPGQSPKLLIYKVSNRFSGVPDRFSGSGSGTDFTLKISRVEAEDLGVYFCSQSTHVPPLTFGSGTKLEIK

^^^^^^^^^^^ ^^^ ^^^^^^^^^^

Best Alignment of Dinutuximab CDR-H3 to a sequence from OAS

EVQLLQSGPELEKPGASVMISCKASGSSFTGYNMNWVRQNIGKSLEWIGAIDPYYGGTSYNQKFKGRATLTVDKSSSTAYMHLKSLTSEDSAVYYCVSGMEYWGQGTSVTVSS

||||..||..|.|||.|...||.|||..|..|.|.||||...|.|||...|......|.|....|||.|...|......|.....|.|||.|.||.|.|||||||.||||||.

EVQLVESGGGLVKPGGSLTLSCAASGFTFSSYAMSWVRQTPEKRLEWVVSISDCGNYTYYPDNVKGRFTSSRDNDKNNLYLQMSHLKSEDTAMYYGVRGMEYWGQETSVTVST

^^^^^^^^ ^^^^^^^^ ^^^^^^

Therapeutic : Domagrozumab

Best Alignment of Domagrozumab heavy chain to a sequence from OAS

EVQLLESGGGLVQPGGSLRLSCAASGFTFSSYAMSWVRQAPGKGLEWVSTISSGGSYTSYPDSVKGRFTISRDNSKNTLYLQMNSLRAEDTAVYYCAKQDY----AMNYWGQGTLVTVSS

|||||||||||||||||||||||||||||||||||||||||||||||||.|.||||.|.|.|||||||||||||||||||||||||||||||||||||||.....|..||||||||||||

EVQLLESGGGLVQPGGSLRLSCAASGFTFSSYAMSWVRQAPGKGLEWVSVIYSGGSSTYYADSVKGRFTISRDNSKNTLYLQMNSLRAEDTAVYYCAKQDGYKERAFDYWGQGTLVTVSS

^^^^^^^^ ^^^^^^^^ ^^^^^^^^^^^^^

Best Alignment of Domagrozumab light chain to a sequence from OAS

DIQMTQSPSSLSASVGDRVTITCKASQDVSTAVAWYQQKPGKAPKLLIYSASYRYTGVPSRFSGSGSGTDFTLTISSLQPEDFATYYCQQHYSTPWTFGGGTKVEIK

|||||||||||||||||||||||.||||.||..||||||||||||||||.||....||||||||||||||||||||||||||||||||||.||||||||.|||||||

DIQMTQSPSSLSASVGDRVTITCRASQDISTSLAWYQQKPGKAPKLLIYAASSLQSGVPSRFSGSGSGTDFTLTISSLQPEDFATYYCQQSYSTPWTFGQGTKVEIK

^^^^^^ ^^^ ^^^^^^^^^

Best Alignment of Domagrozumab heavy chain CDRs to a sequence from OAS

EVQLLESGGGLVQPGGSLRLSCAASGFTFSSYAMSWVRQAPGKGLEWVSTISSGGSYTSYPDSVKGRFTISRDNSKNTLYLQMNSLRAEDTAVYYCAKQDYAMNYWGQGTLVTVSS

||.|.|||||||.|||||.||||||||||||||||||||.|.|.||||.|||||||||.|||||||||||||||.||||||||.|||.||||.||||.|||||.||||||.|||||

EVKLVESGGGLVKPGGSLKLSCAASGFTFSSYAMSWVRQTPEKRLEWVATISSGGSYTYYPDSVKGRFTISRDNAKNTLYLQMSSLRSEDTAMYYCARQDYAMDYWGQGTSVTVSS

^^^^^^^^ ^^^^^^^^ ^^^^^^^^^

Best Alignment of Domagrozumab light chain CDRs to a sequence from OAS

DIQMTQSPSSLSASVGDRVTITCKASQDVSTAVAWYQQKPGKAPKLLIYSASYRYTGVPSRFSGSGSGTDFTLTISSLQPEDFATYYCQQHYSTPWTFGGGTKVEIK

||.||||....|.||||||.|||||||||||||||||||||..||||||||||||||||.||.|||||||||.||||.|.||.|.||||||||||||||||||.|||

DIVMTQSHKFMSTSVGDRVSITCKASQDVSTAVAWYQQKPGHSPKLLIYSASYRYTGVPDRFTGSGSGTDFTFTISSVQAEDLAVYYCQQHYSTPWTFGGGTKLEIK

^^^^^^ ^^^ ^^^^^^^^^

Best Alignment of Domagrozumab CDR-H3 to a sequence from OAS

EVQLLESGGGLVQPGGSLRLSCAASGFTFSSYAMSWVRQAPGKGLEWVSTISSGGSYTSYPDSVKGRFTISRDNSKNTLYLQMNSLRAEDTAVYYCAKQDYAMNYWGQGTLVTVSS

|||..||||.||.|||||.|||||||||||||.||||||.|.|.||||.|||||||||.|||||||||||||||.||||||||.||..||||.||||||||||.||||||.|||||

EVQRVESGGDLVKPGGSLKLSCAASGFTFSSYGMSWVRQTPDKRLEWVATISSGGSYTYYPDSVKGRFTISRDNAKNTLYLQMSSLKSEDTAMYYCAKQDYAMDYWGQGTSVTVSS

^^^^^^^^ ^^^^^^^^ ^^^^^^^^^

Therapeutic : Drozitumab

Best Alignment of Drozitumab heavy chain to a sequence from OAS

EVQLVQSGGGVERPGGSLRLSCAASGFTFDDYAMSWVRQAPGKGLEWVSGINWQGGSTGYADSVKGRVTISRDNAKNSLYLQMNSLRAEDTAVYYCAKILGAG--RGWYFDYWGKGTTVTVSS

|||||.|||||.||||||||||||||||||||.||||||||||||||||||||.|||||||||||||.||||||||||||||||||||||||.|||||...||..||.||||||.||.|||||

EVQLVESGGGVVRPGGSLRLSCAASGFTFDDYGMSWVRQAPGKGLEWVSGINWNGGSTGYADSVKGRFTISRDNAKNSLYLQMNSLRAEDTALYYCAKYTVAGTSRGGYFDYWGQGTMVTVSS

^^^^^^^^ ^^^^^^^^ ^^^^^^^^^^^^^^^^

Best Alignment of Drozitumab light chain to a sequence from OAS

-SELTQDPAVSVALGQTVRITCSGDSLRSYYASWYQQKPGQAPVLVIYGANNRPSGIPDRFSGSSSGNTASLTITGAQAEDEADYYCNSADSSGNHVVFGGGTKLTVL

.|||||||||||||||||||||.||||||||||||||||||||||||||.|||||||||||||||||||||||||||||||||||||||.||||||||||||||||||

SSELTQDPAVSVALGQTVRITCQGDSLRSYYASWYQQKPGQAPVLVIYGKNNRPSGIPDRFSGSSSGNTASLTITGAQAEDEADYYCNSRDSSGNHVVFGGGTKLTVL

^^^^^^ ^^^ ^^^^^^^^^^^

Best Alignment of Drozitumab heavy chain CDRs to a sequence from OAS

EVQLVQSGGGVERPGGSLRLSCAASGFTFDDYAMSWVRQAPGKGLEWVSGINWQGGSTGYADSVKGRVTISRDNAKNSLYLQMNSLRAEDTAVYYCAKILGAGRGWYFDYWGKGTTVTVSS

................||||||||||||||||.|.||||||||||||||.|.|.||||.||||||||.||||||.||||||||||||.||||.|||||.||||||..|||||.||.|||||

----------------SLRLSCAASGFTFDDYTMHWVRQAPGKGLEWVSLISWDGGSTYYADSVKGRFTISRDNSKNSLYLQMNSLRTEDTALYYCAKDLGAGRGTAFDYWGQGTLVTVSS

^^^^^^^^ ^^^^^^^^ ^^^^^^^^^^^^^^

Best Alignment of Drozitumab light chain CDRs to a sequence from OAS

-SELTQDPAVSVALGQTVRITCSGDSLRSYYASWYQQKPGQAPVLVIYGANNRPSGIPDRFSGSSSGNTASLTITGAQAEDEADYYCNSADSSGNHVVFGGGTKLTVL

.|||||||||||||||||||||.||||||||||||||||||||.|||||.|||||||||||||||||||||||||||||||||||||||.||||||||||||||||||

SSELTQDPAVSVALGQTVRITCQGDSLRSYYASWYQQKPGQAPLLVIYGKNNRPSGIPDRFSGSSSGNTASLTITGAQAEDEADYYCNSRDSSGNHVVFGGGTKLTVL

^^^^^^ ^^^ ^^^^^^^^^^^

Best Alignment of Drozitumab CDR-H3 to a sequence from OAS

EVQLVQSGGGVERPGGSLRLSCAASGFTFDDYAMSWVRQAPGKGLEWVSGINWQGGSTGYADSVKGRVTISRDNAKNSLYLQMNSLRAEDTAVYYCAKILGAGRGWYFDYWGKGTTVTVSS

................|||||||||||||..|||.||||||||||||||.|...|..|.||||||||.||||||.|..||||||||||.|||||||||.||||||.||||||.||.|||||

----------------SLRLSCAASGFTFRSYAMNWVRQAPGKGLEWVSAISGSGVRTYYADSVKGRFTISRDNSKKTLYLQMNSLRADDTAVYYCAKDLGAGRGYYFDYWGQGTLVTVSS

^^^^^^^^ ^^^^^^^^ ^^^^^^^^^^^^^^

Therapeutic : Duligotuzumab

Best Alignment of Duligotuzumab heavy chain to a sequence from OAS

EVQLVESGGGLVQPGGSLRLSCAASGFTLSGDWIHWVRQAPGKGLEWVGEISAAGGYTDYADSVKGRFTISADTSKNTAYLQMNSLRAEDTAVYYCARESRVS-----FEAAMDYWGQGTLVTVSS

.|||..|||||||||||||||||||||..|...|||||||||||||||..||...|||.|||||||||||||||||||||||||||||||||||||||..|.|........|||||||||||||||

QVQLLKSGGGLVQPGGSLRLSCAASGFNFSSSSIHWVRQAPGKGLEWVAYISSSYGYTYYADSVKGRFTISADTSKNTAYLQMNSLRAEDTAVYYCARTVRGSKKPYFSGWAMDYWGQGTLVTVSS

^^^^^^^^ ^^^^^^^^ ^^^^^^^^^^^^^^^^^^^

Best Alignment of Duligotuzumab light chain to a sequence from OAS

DIQMTQSPSSLSASVGDRVTITCRASQNIATDVAWYQQKPGKAPKLLIYSASFLYSGVPSRFSGSGSGTDFTLTISSLQPEDFATYYCQQSEPEPYTFGQGTKVEIK

|||||||||||||||||||||||||||||||...|||||||||||||||.||.|.||||||||||||||||||||||||||||||||||||...|.|||||||||||

DIQMTQSPSSLSASVGDRVTITCRASQNIATFLNWYQQKPGKAPKLLIYAASSLQSGVPSRFSGSGSGTDFTLTISSLQPEDFATYYCQQSYSTPWTFGQGTKVEIK

^^^^^^ ^^^ ^^^^^^^^^

Best Alignment of Duligotuzumab heavy chain CDRs to a sequence from OAS

EVQLVESGGGLVQPGGSLRLSCAASGFTLSGDWIHWVRQAPGKGLEWVGEISAAGGYTDYADSVKGRFTISADTSKNTAYLQMNSLRAEDTAVYYCARESRVSFEAAMDYWGQGTLVTVSS

|||.||||||||.|||||.|||||||||.|.....||||.|.|.||||..||..|.||.|.||||||||||.|..||..||||.||..||||.|||||||.|.|..|||||||||.|||||

EVQRVESGGGLVKPGGSLKLSCAASGFTFSDYYMYWVRQTPEKRLEWVATISDGGSYTYYPDSVKGRFTISRDNAKNNLYLQMSSLKSEDTAMYYCARESMVTFRYAMDYWGQGTSVTVSS

^^^^^^^^ ^^^^^^^^ ^^^^^^^^^^^^^^

Best Alignment of Duligotuzumab light chain CDRs to a sequence from OAS

DIQMTQSPSSLSASVGDRVTITCRASQNIATDVAWYQQKPGKAPKLLIYSASFLYSGVPSRFSGSGSGTDFTLTISSLQPEDFATYYCQQSEPEPYTFGQGTKVEIK

||.|||||.||||||||||.|||||||||.||..||||||||||||||||||.|..|||||||||||||||||||||||.||..|.|||||...|||||||||.|||

DIRMTQSPPSLSASVGDRVIITCRASQNINTDLNWYQQKPGKAPKLLIYSASNLEGGVPSRFSGSGSGTDFTLTISSLQLEDLSTFYCQQSYSAPYTFGQGTKLEIK

^^^^^^ ^^^ ^^^^^^^^^

Best Alignment of Duligotuzumab CDR-H3 to a sequence from OAS

EVQLVESGGGLVQPGGSLRLSCAASGFTLSGDWIHWVRQAPGKGLEWVGEISAAGGYTDYADSVKGRFTISADTSKNTAYLQMNSLRAEDTAVYYCARESRVSFEAAMDYWGQGTLVTVSS

.|||...|..||.||.|..|||.|||.|....|..||.|.||.||||.|.|......|.|....|...|...|.|..|||.|..||..||.||||||||.||||..|||||||||.|||||

-VQLQQPGAELVRPGSSVKLSCKASGYTFTSYWMDWVKQRPGQGLEWIGNIYPSDSETHYNQKFKDKATLTVDKSSSTAYMQLSSLTSEDSAVYYCARENRVSFFYAMDYWGQGTSVTVSS

^^^^^^^^ ^^^^^^^^ ^^^^^^^^^^^^^^

Therapeutic : Dupilumab

Best Alignment of Dupilumab heavy chain to a sequence from OAS

EVQLVESGGGLEQPGGSLRLSCAGSGFTFRDYAMTWVRQAPGKGLEWVSSISGSGGNTYYADSVKGRFTISRDNSKNTLYLQMNSLRAEDTAVYYCAKDRLSITI--RPRYYGLDVWGQGTTVTVSS

||||.||||||.|||||||||||.|||||..||||||||||||||||||.||||||||||||||||||||||||||||||||||||||||||||||||||..........|||.|||||||||||||

EVQLLESGGGLVQPGGSLRLSCAASGFTFSSYAMTWVRQAPGKGLEWVSAISGSGGNTYYADSVKGRFTISRDNSKNTLYLQMNSLRAEDTAVYYCAKDRSITMVRGQSGYYGMDVWGQGTTVTVSS

^^^^^^^^ ^^^^^^^^ ^^^^^^^^^^^^^^^^^^^^

Best Alignment of Dupilumab light chain to a sequence from OAS

DIVMTQSPLSLPVTPGEPASISCRSSQSLLYSIGYNYLDWYLQKSGQSPQLLIYLGSNRASGVPDRFSGSGSGTDFTLKISRVEAEDVGFYYCMQALQTPYTFGQGTKLEIK

||||||||||||||||||||||||||||||.|.||||||||||||||||||||||||||||||||||||||||||||||||||||||||.||||||||||||||||||||||

DIVMTQSPLSLPVTPGEPASISCRSSQSLLHSNGYNYLDWYLQKSGQSPQLLIYLGSNRASGVPDRFSGSGSGTDFTLKISRVEAEDVGVYYCMQALQTPYTFGQGTKLEIK

^^^^^^^^^^^ ^^^ ^^^^^^^^^

Best Alignment of Dupilumab heavy chain CDRs to a sequence from OAS

EVQLVESGGGLEQPGGSLRLSCAGSGFTFRDYAMTWVRQAPGKGLEWVSSISGSGGNTYYADSVKGRFTISRDNSKNTLYLQMNSLRAEDTAVYYCAKDRLSITIRPRYYGLDVWGQGTTVTVSS

..............|||||||||.|||||..|||.||||||||||||||.||||||.|||||||||||||||||||||||||||||||||||||||||||.||||...|||.||||||||||||.

--------------GGSLRLSCAASGFTFSSYAMSWVRQAPGKGLEWVSAISGSGGSTYYADSVKGRFTISRDNSKNTLYLQMNSLRAEDTAVYYCAKDRGSITIWDYYYGMDVWGQGTTVTVS-

^^^^^^^^ ^^^^^^^^ ^^^^^^^^^^^^^^^^^^

Best Alignment of Dupilumab light chain CDRs to a sequence from OAS

DIVMTQSPLSLPVTPGEPASISCRSSQSLLYSIGYNYLDWYLQKSGQSPQLLIYLGSNRASGVPDRFSGSGSGTDFTLKISRVEAEDVGFYYCMQALQTPYTFGQGTKLEIK

||||||.|||||||||||||||||||||||||.|||||||||||.||||||||||||.|||||||||||||.||||||.||||||||||.||||||||||||||||||||||

DIVMTQPPLSLPVTPGEPASISCRSSQSLLYSNGYNYLDWYLQKPGQSPQLLIYLGSTRASGVPDRFSGSGTGTDFTLNISRVEAEDVGMYYCMQALQTPYTFGQGTKLEIK

^^^^^^^^^^^ ^^^ ^^^^^^^^^

Best Alignment of Dupilumab CDR-H3 to a sequence from OAS

EVQLVESGGGLEQPGGSLRLSCAGSGFTFRDYAMTWVRQAPGKGLEWVSSISGSGGNTYYADSVKGRFTISRDNSKNTLYLQMNSLRAEDTAVYYCAKDRLSITIRPRYYGLDVWGQGTTVTVSS

..............|||||||||.|||||..|||.||||||||||||||.||||||.|||||||||||||||||||||||||||||||||||||||||||.||||...|||.||||||||||||.

--------------GGSLRLSCAASGFTFSSYAMSWVRQAPGKGLEWVSAISGSGGSTYYADSVKGRFTISRDNSKNTLYLQMNSLRAEDTAVYYCAKDRGSITIWDYYYGMDVWGQGTTVTVS-

^^^^^^^^ ^^^^^^^^ ^^^^^^^^^^^^^^^^^^

Therapeutic : Durvalumab

Best Alignment of Durvalumab heavy chain to a sequence from OAS

EVQLVESGGGLVQPGGSLRLSCAASGFTFSRYWMSWVRQAPGKGLEWVANIKQDGSEKYYVDSVKGRFTISRDNAKNSLYLQMNSLRAEDTAVYYCAREGGWFGELAFDYWGQGTLVTVSS

||||||||||||||||||||||||||||||.|||||||||||||||||||||||||||||||||||||||||||||||||||||||||||||||||||.|.|||||.||||||||||||||

EVQLVESGGGLVQPGGSLRLSCAASGFTFSSYWMSWVRQAPGKGLEWVANIKQDGSEKYYVDSVKGRFTISRDNAKNSLYLQMNSLRAEDTAVYYCARSGLWFGELPFDYWGQGTLVTVSS

^^^^^^^^ ^^^^^^^^ ^^^^^^^^^^^^^^

Best Alignment of Durvalumab light chain to a sequence from OAS

EIVLTQSPGTLSLSPGERATLSCRASQRVSSSYLAWYQQKPGQAPRLLIYDASSRATGIPDRFSGSGSGTDFTLTISRLEPEDFAVYYCQQYGS-LPWTFGQGTKVEIK

|||||||||||||||||||||||||||||||.||||||||||||||||||||||||||||||||||||||||||||||||||||||||||||||.||||||||||||||

EIVLTQSPGTLSLSPGERATLSCRASQRVSSNYLAWYQQKPGQAPRLLIYDASSRATGIPDRFSGSGSGTDFTLTISRLEPEDFAVYYCQQYGSSLPWTFGQGTKVEIK

^^^^^^^ ^^^ ^^^^^^^^^^

Best Alignment of Durvalumab heavy chain CDRs to a sequence from OAS

EVQLVESGGGLVQPGGSLRLSCAASGFTFSRYWMSWVRQAPGKGLEWVANIKQDGSEKYYVDSVKGRFTISRDNAKNSLYLQMNSLRAEDTAVYYCAREGGWFGELAFDYWGQGTLVTVSS

................||||||||||||||.|||||||||||||||||||||||||||||||||||||||||||||||||||||||||||||||||||.|||||||.||||||||||||||

----------------SLRLSCAASGFTFSSYWMSWVRQAPGKGLEWVANIKQDGSEKYYVDSVKGRFTISRDNAKNSLYLQMNSLRAEDTAVYYCARDGGWFGELPFDYWGQGTLVTVSS

^^^^^^^^ ^^^^^^^^ ^^^^^^^^^^^^^^

Best Alignment of Durvalumab light chain CDRs to a sequence from OAS

EIVLTQSPGTLSLSPGERATLSCRASQRVSSSYLAWYQQKPGQAPRLLIYDASSRATGIPDRFSGSGSGTDFTLTISRLEPEDFAVYYCQQYGSLPWTFGQGTKVEIK

||||||||.||||||||||||||.||||||||||||||||||.|||||||||||||||||||||||||||||||||||||||||||||||||||.|||||||||||||

EIVLTQSPATLSLSPGERATLSCGASQRVSSSYLAWYQQKPGLAPRLLIYDASSRATGIPDRFSGSGSGTDFTLTISRLEPEDFAVYYCQQYGSSPWTFGQGTKVEIK

^^^^^^^ ^^^ ^^^^^^^^^

Best Alignment of Durvalumab CDR-H3 to a sequence from OAS

EVQLVESGGGLVQPGGSLRLSCAASGFTFSRYWMSWVRQAPGKGLEWVANIKQDGSEKYYVDSVKGRFTISRDNAKNSLYLQMNSLRAEDTAVYYCAREGGWFGELAFDYWGQGTLVTVSS

...............|||||||||||||||.|.|.||||||||||||||.|..|||.|||.||||||||||||||||||.||||||||||||.|||||||.|||||||||||||||||||.

---------------GSLRLSCAASGFTFSSYGMHWVRQAPGKGLEWVAVIWYDGSNKYYADSVKGRFTISRDNAKNSLFLQMNSLRAEDTAMYYCAREGLWFGELAFDYWGQGTLVTVS-

^^^^^^^^ ^^^^^^^^ ^^^^^^^^^^^^^^

Therapeutic : Dusigitumab

Best Alignment of Dusigitumab heavy chain to a sequence from OAS

QVQLVQSGAEVKKPGASVKVSCKASGYTFTSYDINWVRQATGQGLEWMGWMNPNSGNTGYAQKFQGRVTMTRNTSISTAYMELSSLRSEDTAVYYCARDPYYY-------------YYGMDVWGQGTTVTVSS

||||||||||||||||||||||||||||||||||||||||||||||||||||||||||||||||||||||||||||||||||||||||||||||||||||.||.............|||||||||||||||||

QVQLVQSGAEVKKPGASVKVSCKASGYTFTSYDINWVRQATGQGLEWMGWMNPNSGNTGYAQKFQGRVTMTRNTSISTAYMELSSLRSEDTAVYYCARDPSYYYGSGSYRPPLEYYYYGMDVWGQGTTVTVSS

^^^^^^^^ ^^^^^^^^ ^^^^^^^^^^^^^^^^^^^^^^^^^^

Best Alignment of Dusigitumab light chain to a sequence from OAS

QSVLTQPPSVSAAPGQKVTISCSGSSSNIENNHVSWYQQLPGTAPKLLIYDNNKRPSGIPDRFSGSKSGTSATLGITGLQTGDEADYYCETWDTSLSAGRVFGGGTKLTVL

|||||||||||||||||||||||||||||.||.||||||||||||||||||||||||||||||||||||||||||||||||||||||||.|||||||||||||||||||||

QSVLTQPPSVSAAPGQKVTISCSGSSSNIGNNYVSWYQQLPGTAPKLLIYDNNKRPSGIPDRFSGSKSGTSATLGITGLQTGDEADYYCGTWDTSLSAGRVFGGGTKLTVL

^^^^^^^^ ^^^ ^^^^^^^^^^^^

Best Alignment of Dusigitumab heavy chain CDRs to a sequence from OAS

QVQLVQSGAEVKKPGASVKVSCKASGYTFTSYDINWVRQATGQGLEWMGWMNPNSGNTGYAQKFQGRVTMTRNTSISTAYMELSSLRSEDTAVYYCARDPYYYYYGMDVWGQGTTVTVSS

..............|||||||||||||||||||||||||||||||||||||||||||||||||||||||||||||||||||||||||||||||||||||||||||||||||||||||||.

--------------GASVKVSCKASGYTFTSYDINWVRQATGQGLEWMGWMNPNSGNTGYAQKFQGRVTMTRNTSISTAYMELSSLRSEDTAVYYCARDPYYYYYGMDVWGQGTTVTVS-

^^^^^^^^ ^^^^^^^^ ^^^^^^^^^^^^^

Best Alignment of Dusigitumab light chain CDRs to a sequence from OAS

QSVLTQPPSVSAAPGQKVTISCSGSSSNIENNHVSWYQQLPGTAPKLLIYDNNKRPSGIPDRFSGSKSGTSATLGITGLQTGDEADYYCETWDTSLSAGRVFGGGTKLTVL

||||||||||||||||.|.|||||||||||||.|||||.||||||||||||||||||||.|||||||||||||||||||||||||||||.|||||||||.|||.|||.|||

QSVLTQPPSVSAAPGQNVNISCSGSSSNIENNFVSWYQKLPGTAPKLLIYDNNKRPSGIRDRFSGSKSGTSATLGITGLQTGDEADYYCGTWDTSLSAGGVFGSGTKVTVL

^^^^^^^^ ^^^ ^^^^^^^^^^^^

Best Alignment of Dusigitumab CDR-H3 to a sequence from OAS

QVQLVQSGAEVKKPGASVKVSCKASGYTFTSYDINWVRQATGQGLEWMGWMNPNSGNTGYAQKFQGRVTMTRNTSISTAYMELSSLRSEDTAVYYCARDPYYYYYGMDVWGQGTTVTVSS

..............||||||||||||.||.||.|.|||||.||||||||...|..|...||||||||||.|...|.||||||||||||||||||||||||||||||||||||||||||||

--------------GASVKVSCKASGGTFSSYAISWVRQAPGQGLEWMGGIIPIFGTANYAQKFQGRVTITADESTSTAYMELSSLRSEDTAVYYCARDPYYYYYGMDVWGQGTTVTVSS

^^^^^^^^ ^^^^^^^^ ^^^^^^^^^^^^^

Therapeutic : Eculizumab

Best Alignment of Eculizumab heavy chain to a sequence from OAS

QVQLVQSGAEVKKPGASVKVSCKASGYIFSNYWIQWVRQAPGQGLEWMGEILPGSGSTEYTENFKDRVTMTRDTSTSTVYMELSSLRSEDTAVYYCARYFFGSS---PNWYFDVWGQGTLVTVSS

|||||||||||||||||||||||||||.|..|...||||||||||||||.|.|..|||.|...|..||||||||||||||||||||||||||||||||.|.||....||.|||.|||||||||||

QVQLVQSGAEVKKPGASVKVSCKASGYTFTSYYMHWVRQAPGQGLEWMGIINPSGGSTSYAQKFQGRVTMTRDTSTSTVYMELSSLRSEDTAVYYCARGFGGSYQINPNYYFDYWGQGTLVTVSS

^^^^^^^^ ^^^^^^^^ ^^^^^^^^^^^^^^^^^^

Best Alignment of Eculizumab light chain to a sequence from OAS

DIQMTQSPSSLSASVGDRVTITCGASENIYGALNWYQQKPGKAPKLLIYGATNLADGVPSRFSGSGSGTDFTLTISSLQPEDFATYYCQNVLNTPLTFGQGTKVEIK

|||||||||||||||||||||||.||.||...|||||||||||||||||.|.||..|||||||||||||||||||||||||||||||||...|||||||.|||||||

DIQMTQSPSSLSASVGDRVTITCRASQNIRNYLNWYQQKPGKAPKLLIYTASNLQSGVPSRFSGSGSGTDFTLTISSLQPEDFATYYCQQSDNTPLTFGGGTKVEIK

^^^^^^ ^^^ ^^^^^^^^^

Best Alignment of Eculizumab heavy chain CDRs to a sequence from OAS

QVQLVQSGAEVKKPGASVKVSCKASGYIFSNYWIQWVRQAPGQGLEWMGEILPGSGSTEYTENFKDRVTMTRDTSTSTVYMELSSLRSEDTAVYYCARYFFGSSPNWYFDVWGQGTLVTVSS

.|||.|||||..|||||||.||||.||.||.|||.||.|.||.||||.||||||||||.|.|.||...|.|.|||..|.||.||||.|||.|||||||...||||||||||||.||.|||||

-VQLQQSGAELMKPGASVKMSCKATGYTFSSYWIEWVKQRPGHGLEWIGEILPGSGSTNYNEKFKGKATFTADTSSNTAYMQLSSLTSEDSAVYYCARDYYGSSPNWYFDVWGAGTTVTVSS

^^^^^^^^ ^^^^^^^^ ^^^^^^^^^^^^^^^

Best Alignment of Eculizumab light chain CDRs to a sequence from OAS

DIQMTQSPSSLSASVGDRVTITCGASENIYGALNWYQQKPGKAPKLLIYGATNLADGVPSRFSGSGSGTDFTLTISSLQPEDFATYYCQNVLNTPLTFGQGTKVEIK

||||||||.|||||||..|||||||||||||||||||.|.|..|.||||||||||||..|||||||||....|..|||.|.|.||||||||||||||||.|||.|.|

DIQMTQSPASLSASVGETVTITCGASENIYGALNWYQRKQGISPQLLIYGATNLADGMSSRFSGSGSGRQYSLKVSSLHPDDVATYYCQNVLNTPLTFGAGTKLELK

^^^^^^ ^^^ ^^^^^^^^^

Best Alignment of Eculizumab CDR-H3 to a sequence from OAS

QVQLVQSGAEVKKPGASVKVSCKASGYIFSNYWIQWVRQAPGQGLEWMGEILPGSGSTEYTENFKDRVTMTRDTSTSTVYMELSSLRSEDTAVYYCARYFFGSSPNWYFDVWGQGTLVTVSS

.|.||.||||..|||||||.||||.||.|..|||.||.|.||.||||.||||||||||.|.|.||...|.|.|||..|.||.||||..||.|.||||||..||||||||||||.||.|||||

-VKLVESGAELMKPGASVKLSCKATGYTFTGYWIEWVKQRPGHGLEWIGEILPGSGSTNYNEKFKGKATFTADTSSNTAYMQLSSLTTEDSAIYYCARYYYGSSPNWYFDVWGTGTTVTVSS

^^^^^^^^ ^^^^^^^^ ^^^^^^^^^^^^^^^

Therapeutic : Efalizumab

Best Alignment of Efalizumab heavy chain to a sequence from OAS

EVQLVESGGGLVQPGGSLRLSCAASGYSFTGHWMNWVRQAPGKGLEWVGMIHPSDSETRYNQKFKDRFTISVDKSKNTLYLQMNSLRAEDTAVYYCARGIYFY----------GTTYFDYWGQGTLVTVSS

||||||||||||||||||||||||||..|....|||||||||||||||..|..|...|.|....|.|||||.|.|||||||||||||||||||||||.|..||..........||.|||||||||||||||

EVQLVESGGGLVQPGGSLRLSCAASGFTFSSYSMNWVRQAPGKGLEWVSAISGSGGSTYYADSVKGRFTISRDNSKNTLYLQMNSLRAEDTAVYYCAKGSGFYDILTGYYGPTGTHYFDYWGQGTLVTVSS

^^^^^^^^ ^^^^^^^^ ^^^^^^^^^^^^^^^^^^^^^^^^

Best Alignment of Efalizumab light chain to a sequence from OAS

DIQMTQSPSSLSASVGDRVTITCRASKTISKYLAWYQQKPGKAPKLLIYSGSTLQSGVPSRFSGSGSGTDFTLTISSLQPEDFATYYCQQHNEYPLTFGQGTKVEIK

||||||||||||||||||||||||||..||.|||||||||||||||||||.|||||||||||||||||||||||||||||||||||||||||.||.|||||||||||

DIQMTQSPSSLSASVGDRVTITCRASQGISSYLAWYQQKPGKAPKLLIYSASTLQSGVPSRFSGSGSGTDFTLTISSLQPEDFATYYCQQHNSYPWTFGQGTKVEIK

^^^^^^ ^^^ ^^^^^^^^^

Best Alignment of Efalizumab heavy chain CDRs to a sequence from OAS

EVQLVESGGGLVQPGGSLRLSCAASGYSFTGHWMNWVRQAPGKGLEWVGMIHPSDSETRYNQKFKDRFTISVDKSKNTLYLQMNSLRAEDTAVYYCARGIYFYGTTYFDYWGQGTLVTVSS

.|||..||..||.||.|..|||.|||||||..|||||.|.||.||||.|||||||||||.||||||..|..||||..|.|.|..|...||.||||||||||.||..|||||||||..||||

QVQLQQSGAELVRPGASVKLSCKASGYSFTSYWMNWVKQRPGQGLEWIGMIHPSDSETRLNQKFKDKATLTVDKSSSTAYMQLSSPTSEDSAVYYCARGIYYYGSSYFDYWGQGTTLTVSS

^^^^^^^^ ^^^^^^^^ ^^^^^^^^^^^^^^

Best Alignment of Efalizumab light chain CDRs to a sequence from OAS

DIQMTQSPSSLSASVGDRVTITCRASKTISKYLAWYQQKPGKAPKLLIYSGSTLQSGVPSRFSGSGSGTDFTLTISSLQPEDFATYYCQQHNEYPLTFGQGTKVEIK

|.|.|||||.|.||.|...||.|||||||||||||||.||||..|||||||||||||.||||||||||||||||||||.|||||.||||||||||||||.|||.|.|

DVQITQSPSYLAASPGETITINCRASKTISKYLAWYQEKPGKTNKLLIYSGSTLQSGIPSRFSGSGSGTDFTLTISSLEPEDFAMYYCQQHNEYPLTFGAGTKLELK

^^^^^^ ^^^ ^^^^^^^^^

Best Alignment of Efalizumab CDR-H3 to a sequence from OAS

EVQLVESGGGLVQPGGSLRLSCAASGYSFTGHWMNWVRQAPGKGLEWVGMIHPSDSETRYNQKFKDRFTISVDKSKNTLYLQMNSLRAEDTAVYYCARGIYFYGTTYFDYWGQGTLVTVSS

||.|||||||||.|||||.|||.|||..|....|.||||.|.|.||||..|......|.|....|.|||||.|...|.|||||.|.|.||||.||||||.||||.||||||||||...|||

EVELVESGGGLVKPGGSLKLSCEASGFAFSSYVMSWVRQTPEKRLEWVASISSG-GNTYYPDSVKGRFTISRDDARNILYLQMSSQRSEDTAMYYCARGPYFYGITYFDYWGQGTTLSVSS

^^^^^^^^ ^^^^^^^^ ^^^^^^^^^^^^^^

Therapeutic : Eldelumab

Best Alignment of Eldelumab heavy chain to a sequence from OAS

QMQLVESGGGVVQPGRSLRLSCTASGFTFSNNGMHWVRQAPGKGLEWVAVIWFDGMNKFYVDSVKGRFTISRDNSKNTLYLEMNSLRAEDTAIYYCAREGDGSGI-YYYYGMDVWGQGTTVTVSS

|.||||||||||||||||||||.|||||||..||||||||||||||||||||.||.||.|.||||||||||||||||||||.||||||||||.||||||||.||..|||||||||||||||||||

QVQLVESGGGVVQPGRSLRLSCAASGFTFSSYGMHWVRQAPGKGLEWVAVIWYDGSNKYYADSVKGRFTISRDNSKNTLYLQMNSLRAEDTAVYYCAREGDSSGYYYYYYGMDVWGQGTTVTVSS

^^^^^^^^ ^^^^^^^^ ^^^^^^^^^^^^^^^^^^

Best Alignment of Eldelumab light chain to a sequence from OAS

EIVLTQSPGTLSLSPGERATLSCRASQSVSSSYLAWYQQKPGQAPRLLIYGASSRATGIPDRFSGSGSGTDFTLTISRLEPEDFAVYYCQQYGSSPIFTFGPGTKVDIK

|||||||||||||||||||||||||||||||||||||||||||||||||||||||||||||||||||||||||||||||||||||||||||||||||||||||||||||

EIVLTQSPGTLSLSPGERATLSCRASQSVSSSYLAWYQQKPGQAPRLLIYGASSRATGIPDRFSGSGSGTDFTLTISRLEPEDFAVYYCQQYGSSPIFTFGPGTKVDIK

^^^^^^^ ^^^ ^^^^^^^^^^

Best Alignment of Eldelumab heavy chain CDRs to a sequence from OAS

QMQLVESGGGVVQPGRSLRLSCTASGFTFSNNGMHWVRQAPGKGLEWVAVIWFDGMNKFYVDSVKGRFTISRDNSKNTLYLEMNSLRAEDTAIYYCAREGDGSGIYYYYGMDVWGQGTTVTVSS

|.||||||||||||||||||||.|||||||..|||||||||||||||.||||.||.||.|.||||||.|||||||||||.|.||||||||||.|.||||||||..|||||||||||||||||||

QVQLVESGGGVVQPGRSLRLSCAASGFTFSSYGMHWVRQAPGKGLEWGAVIWYDGSNKYYADSVKGRLTISRDNSKNTLHLQMNSLRAEDTAVYFCAREGDGSYGYYYYGMDVWGQGTTVTVSS

^^^^^^^^ ^^^^^^^^ ^^^^^^^^^^^^^^^^^

Best Alignment of Eldelumab light chain CDRs to a sequence from OAS

EIVLTQSPGTLSLSPGERATLSCRASQSVSSSYLAWYQQKPGQAPRLLIYGASSRATGIPDRFSGSGSGTDFTLTISRLEPEDFAVYYCQQYGSSPIFTFGPGTKVDIK

|||||||||||||||||||||||||||||||||||||||||||||||||||||||||||||||||||||||||||||||||||||||||||||||||||||||||||||

EIVLTQSPGTLSLSPGERATLSCRASQSVSSSYLAWYQQKPGQAPRLLIYGASSRATGIPDRFSGSGSGTDFTLTISRLEPEDFAVYYCQQYGSSPIFTFGPGTKVDIK

^^^^^^^ ^^^ ^^^^^^^^^^

Best Alignment of Eldelumab CDR-H3 to a sequence from OAS

QMQLVESGGGVVQPGRSLRLSCTASGFTFSNNGMHWVRQAPGKGLEWVAVIWFDGMNKFYVDSVKGRFTISRDNSKNTLYLEMNSLRAEDTAIYYCAREGDGSGIYYYYGMDVWGQGTTVTVSS

..............|.||||||.|||||.|.|.|.|||||||||||||.||........|.||||||||||||||||||||.||||||||||.|||||||||||.||||||||||||||||||.

--------------GGSLRLSCAASGFTVSSNYMSWVRQAPGKGLEWVSVIYSG-GSTYYADSVKGRFTISRDNSKNTLYLQMNSLRAEDTAVYYCAREGDGSGGYYYYGMDVWGQGTTVTVS-

^^^^^^^^ ^^^^^^^^ ^^^^^^^^^^^^^^^^^

Therapeutic : Elotuzumab

Best Alignment of Elotuzumab heavy chain to a sequence from OAS

EVQLVESGGGLVQPGGSLRLSCAASGFDFSRYWMSWVRQAPGKGLEWIGEINPDSSTINYAPSLKDKFIISRDNAKNSLYLQMNSLRAEDTAVYYCARPDGNYWYFDVWGQGTLVTVSS

||||||||||||||||||.|||||||||||||||||||||||||||||||||||||||||.||||||||||||||||.|||||...|.||||.|||||.|||||||||||.||.|||||

EVQLVESGGGLVQPGGSLKLSCAASGFDFSRYWMSWVRQAPGKGLEWIGEINPDSSTINYTPSLKDKFIISRDNAKNTLYLQMSKVRSEDTALYYCARHDGNYWYFDVWGAGTTVTVSS

^^^^^^^^ ^^^^^^^^ ^^^^^^^^^^^^

Best Alignment of Elotuzumab light chain to a sequence from OAS

DIQMTQSPSSLSASVGDRVTITCKASQDVGIAVAWYQQKPGKVPKLLIYWASTRHTGVPDRFSGSGSGTDFTLTISSLQPEDVATYYCQQYSS-YPYTFGQGTKVEIK

|||||||||||||||||||||||.|||.|.|.|||||||||||||||||.|||...|||.|||||||||||||||||||||||||||||||.|..|.|||.|||||||

DIQMTQSPSSLSASVGDRVTITCRASQGVSIYVAWYQQKPGKVPKLLIYAASTLQSGVPSRFSGSGSGTDFTLTISSLQPEDVATYYCQQYNSAPPLTFGGGTKVEIK

^^^^^^ ^^^ ^^^^^^^^^^

Best Alignment of Elotuzumab heavy chain CDRs to a sequence from OAS

EVQLVESGGGLVQPGGSLRLSCAASGFDFSRYWMSWVRQAPGKGLEWIGEINPDSSTINYAPSLKDKFIISRDNAKNSLYLQMNSLRAEDTAVYYCARPDGNYWYFDVWGQGTLVTVSS

||.|||||||||||||||.|||||||||||||||||||||||||||||||||||||||||.||||||||||||||||.|||||.....||||.||||||||.||||||||.||.|||||

EVKLVESGGGLVQPGGSLKLSCAASGFDFSRYWMSWVRQAPGKGLEWIGEINPDSSTINYTPSLKDKFIISRDNAKNTLYLQMSKVISEDTALYYCARPDGYYWYFDVWGAGTTVTVSS

^^^^^^^^ ^^^^^^^^ ^^^^^^^^^^^^

Best Alignment of Elotuzumab light chain CDRs to a sequence from OAS

DIQMTQSPSSLSASVGDRVTITCKASQDVGIAVAWYQQKPGKVPKLLIYWASTRHTGVPDRFSGSGSGTDFTLTISSLQPEDVATYYCQQYSSYPYTFGQGTKVEIK

||.||||....|.||||||.|||||||||||||||||||||..|||||||||||||||||||.|||||||||||||..|.||.|.|.||||||||||||.|||.|||

DIVMTQSHKFMSTSVGDRVSITCKASQDVGIAVAWYQQKPGQSPKLLIYWASTRHTGVPDRFTGSGSGTDFTLTISNVQSEDLADYFCQQYSSYPYTFGGGTKLEIK

^^^^^^ ^^^ ^^^^^^^^^

Best Alignment of Elotuzumab CDR-H3 to a sequence from OAS

EVQLVESGGGLVQPGGSLRLSCAASGFDFSRYWMSWVRQAPGKGLEWIGEINPDSSTINYAPSLKDKFIISRDNAKNSLYLQMNSLRAEDTAVYYCARPDGNYWYFDVWGQGTLVTVSS

||.|||||||||||||||.||||||||.||.|.|.||||.|.|.|||...|........|....|....||||.|||.|||||..|..||||.|||||||||||||||||.||.|||||

EVHLVESGGGLVQPGGSLKLSCAASGFTFSDYYMYWVRQTPEKRLEWVAYISNGGGSTYYPDTVKGRSTISRDTAKNTLYLQMSRLKSEDTAMYYCARPDGNYWYFDVWGTGTTVTVSS

^^^^^^^^ ^^^^^^^^ ^^^^^^^^^^^^

Therapeutic : Emapalumab

Best Alignment of Emapalumab heavy chain to a sequence from OAS

EVQLLESGGGLVQPGGSLRLSCAASGFTFSSYAMSWVRQAPGKGLEWVSAISGSGGSTYYADSVKGRFTISRDNSKNTLYLQMNSLRAEDTAVYYCAKDGSSGWYVPHWFDPWGQGTLVTVSS

||||||||||||||||||||||||||||||||||||||||||||||||||||||||||||||||||||||||||||||||||||||||||||||||||||||.||.|.|||||||||||||||

EVQLLESGGGLVQPGGSLRLSCAASGFTFSSYAMSWVRQAPGKGLEWVSAISGSGGSTYYADSVKGRFTISRDNSKNTLYLQMNSLRAEDTAVYYCAKDGSSSWYGPNWFDPWGQGTLVTVSS

^^^^^^^^ ^^^^^^^^ ^^^^^^^^^^^^^^^^

Best Alignment of Emapalumab light chain to a sequence from OAS

NFMLTQPHSVSESPGKTVTISCTRSSGSIASNYVQWYQQRPGSSPTTVIYEDNQRPSGVPDRFSGSIDSSSNSASLTISGLKTEDEADYYCQSYDGSNRWMFGGGTKLTVL

||||||||||||||||||||||||||||||||||||||||||||||||||||||||||||||||||||||||||||||||||||||||||||||||||||.||||||||||

NFMLTQPHSVSESPGKTVTISCTRSSGSIASNYVQWYQQRPGSSPTTVIYEDNQRPSGVPDRFSGSIDSSSNSASLTISGLKTEDEADYYCQSYDGSNRWVFGGGTKLTVL

^^^^^^^^ ^^^ ^^^^^^^^^^

Best Alignment of Emapalumab heavy chain CDRs to a sequence from OAS

EVQLLESGGGLVQPGGSLRLSCAASGFTFSSYAMSWVRQAPGKGLEWVSAISGSGGSTYYADSVKGRFTISRDNSKNTLYLQMNSLRAEDTAVYYCAKDGSSGWYVPHWFDPWGQGTLVTVSS

.............||||||||||||||||||||||||||||||||||||||||||||||||||||||||||||||||||||||||||||||||||||||||||||.|.|||||||||||||||

-------------PGGSLRLSCAASGFTFSSYAMSWVRQAPGKGLEWVSAISGSGGSTYYADSVKGRFTISRDNSKNTLYLQMNSLRAEDTAVYYCAKDGSSGWYTPGWFDPWGQGTLVTVSS

^^^^^^^^ ^^^^^^^^ ^^^^^^^^^^^^^^^^

Best Alignment of Emapalumab light chain CDRs to a sequence from OAS

NFMLTQPHSVSESPGKTVTISCTRSSGSIASNYVQWYQQRPGSSPTTVIYEDNQRPSGVPDRFSGSIDSSSNSASLTISGLKTEDEADYYCQSYDGSNRWMFGGGTKLTVL

|.|||||||||||||||||||||||||||||||||||||||||||||||||||||||||||||||.||.||||||||||||||.||||||||||||||||.||||||||||

NLMLTQPHSVSESPGKTVTISCTRSSGSIASNYVQWYQQRPGSSPTTVIYEDNQRPSGVPDRFSGPIDGSSNSASLTISGLKTGDEADYYCQSYDGSNRWVFGGGTKLTVL

^^^^^^^^ ^^^ ^^^^^^^^^^

Best Alignment of Emapalumab CDR-H3 to a sequence from OAS

EVQLLESGGGLVQPGGSLRLSCAASGFTFSSYAMSWVRQAPGKGLEWVSAISGSGGSTYYADSVKGRFTISRDNSKNTLYLQMNSLRAEDTAVYYCAKDGSSGWYVPHWFDPWGQGTLVTVSS

..............||||||||||||||||||.|.||||||||||||||.||.|....||||||||||||||||.||.|||||||||||||||||||.|.|||||||||||||||||||||||

--------------GGSLRLSCAASGFTFSSYSMNWVRQAPGKGLEWVSSISSSSSYIYYADSVKGRFTISRDNAKNSLYLQMNSLRAEDTAVYYCARDPSSGWYVPHWFDPWGQGTLVTVSS

^^^^^^^^ ^^^^^^^^ ^^^^^^^^^^^^^^^^

Therapeutic : Emibetuzumab

Best Alignment of Emibetuzumab heavy chain to a sequence from OAS

QVQLVQSGAEVKKPGASVKVSCKASGYTFTDYYMHWVRQAPGQGLEWMGRVNPNRRGTTYNQKFEGRVTMTTDTSTSTAYMELRSLRSDDTAVYYCARAN--WLDYWGQGTTVTVSS

||||||||||||||||||||||||||||||.|||||||||||||||||||.|||..||.|.|||.||||||||||||||||||||||||||||||||||...|.|||||||.|||||

QVQLVQSGAEVKKPGASVKVSCKASGYTFTGYYMHWVRQAPGQGLEWMGRINPNSGGTNYAQKFQGRVTMTTDTSTSTAYMELRSLRSDDTAVYYCARAWNYWGDYWGQGTLVTVSS

^^^^^^^^ ^^^^^^^^ ^^^^^^^^^^

Best Alignment of Emibetuzumab light chain to a sequence from OAS

DIQMTQSPSSLSASVGDRVTITCSVSSSVSSIYLHWYQQKPGKAPKLLIYSTSNLASGVPSRFSGSGSGTDFTLTISSLQPEDFATYYCQVYSGYPLTFGGGTKVEIK

|||||||||||||||||||||||..|.|..|.||.|||||||||||||||..|.|.||||||||||||||||||||||||||||||||||.|..||||||||||||||

DIQMTQSPSSLSASVGDRVTITCRASQSI-SSYLNWYQQKPGKAPKLLIYAASSLQSGVPSRFSGSGSGTDFTLTISSLQPEDFATYYCQQYNSYPLTFGGGTKVEIK

^^^^^^^ ^^^ ^^^^^^^^^

Best Alignment of Emibetuzumab heavy chain CDRs to a sequence from OAS

QVQLVQSGAEVKKPGASVKVSCKASGYTFTDYYMHWVRQAPGQGLEWMGRVNPNRRGTTYNQKFEGRVTMTTDTSTSTAYMELRSLRSDDTAVYYCARANWLDYWGQGTTVTVSS

.|||.|||.|..|||||||.||||||||||||||.||.|..|..|||.|..|||..||.|||||.|..|.|.|.|.||||||||||.|.|.|||||||||||||||||||.||||

EVQLQQSGPELVKPGASVKISCKASGYTFTDYYMNWVKQSHGKSLEWIGDINPNNGGTSYNQKFKGKATLTVDKSSSTAYMELRSLTSEDSAVYYCARANWLDYWGQGTTLTVSS

^^^^^^^^ ^^^^^^^^ ^^^^^^^^

Best Alignment of Emibetuzumab light chain CDRs to a sequence from OAS

DIQMTQSPSSLSASVGDRVTITCSVSSSVSSIYLHWYQQKPGKAPKLLIYSTSNLASGVPSRFSGSGSGTDFTLTISSLQPEDFATYYCQVYSGYPLTFGGGTKVEIK

....||||...|||.|..||.||..|||||||||||||||.|..|||.||||||||||||.|||||||||...|||||...||.||||||.|||||||||||||.|||

ENVLTQSPAIMSASPGEKVTMTCRASSSVSSIYLHWYQQKSGASPKLWIYSTSNLASGVPARFSGSGSGTSYSLTISSVEAEDAATYYCQQYSGYPLTFGGGTKLEIK

^^^^^^^ ^^^ ^^^^^^^^^

Best Alignment of Emibetuzumab CDR-H3 to a sequence from OAS

QVQLVQSGAEVKKPGASVKVSCKASGYTFTDYYMHWVRQAPGQGLEWMGRVNPNRRGTTYNQKFEGRVTMTTDTSTSTAYMELRSLRSDDTAVYYCARANWLDYWGQGTTVTVSS

.|||.|||.|..|||||||.||||||||||||||.||.|..|..|||.|..|||..||.|||||.|..|.|.|.|.||||||||||.|.|.|||||||||||||||||||.||||

EVQLQQSGPELVKPGASVKISCKASGYTFTDYYMNWVKQSHGKSLEWIGDINPNNGGTSYNQKFKGKATLTVDKSSSTAYMELRSLTSEDSAVYYCARANWLDYWGQGTTLTVSS

^^^^^^^^ ^^^^^^^^ ^^^^^^^^

Therapeutic : Enfortumab

Best Alignment of Enfortumab heavy chain to a sequence from OAS

EVQLVESGGGLVQPGGSLRLSCAASGFTFSSYNMNWVRQAPGKGLEWVSYISSSSSTIYYADSVKGRFTISRDNAKNSLSLQMNSLRDEDTAVYYCARAYYYGMDVWGQGTTVTVSS

||||||||||||||||||||||||||||||||.||||||||||||||||||||||||||||||||||||||||||||||.|||||||||||||||||||||||||||||||||||||

EVQLVESGGGLVQPGGSLRLSCAASGFTFSSYSMNWVRQAPGKGLEWVSYISSSSSTIYYADSVKGRFTISRDNAKNSLYLQMNSLRDEDTAVYYCARAYYYGMDVWGQGTTVTVSS

^^^^^^^^ ^^^^^^^^ ^^^^^^^^^^

Best Alignment of Enfortumab light chain to a sequence from OAS

DIQMTQSPSSVSASVGDRVTITCRASQGISGWLAWYQQKPGKAPKFLIYAASTLQSGVPSRFSGSGSGTDFTLTISSLQPEDFATYYCQQANSFPPTFGGGTKVEIK

||||||||||||||||||||||||||||||.||||||||||||||.|||||||||||||||||||||||||||||||||||||||||||||||||||||||||||||

DIQMTQSPSSVSASVGDRVTITCRASQGISSWLAWYQQKPGKAPKLLIYAASTLQSGVPSRFSGSGSGTDFTLTISSLQPEDFATYYCQQANSFPPTFGGGTKVEIK

^^^^^^ ^^^ ^^^^^^^^^

Best Alignment of Enfortumab heavy chain CDRs to a sequence from OAS

EVQLVESGGGLVQPGGSLRLSCAASGFTFSSYNMNWVRQAPGKGLEWVSYISSSSSTIYYADSVKGRFTISRDNAKNSLSLQMNSLRDEDTAVYYCARAYYYGMDVWGQGTTVTVSS

||||||||||||||||||||||||||||||||.||||||||||||||||||||||||||||||||||||||||||||||.|||||||||||||||||||||||||||||||||||||

EVQLVESGGGLVQPGGSLRLSCAASGFTFSSYSMNWVRQAPGKGLEWVSYISSSSSTIYYADSVKGRFTISRDNAKNSLYLQMNSLRDEDTAVYYCARAYYYGMDVWGQGTTVTVSS

^^^^^^^^ ^^^^^^^^ ^^^^^^^^^^

Best Alignment of Enfortumab light chain CDRs to a sequence from OAS

DIQMTQSPSSVSASVGDRVTITCRASQGISGWLAWYQQKPGKAPKFLIYAASTLQSGVPSRFSGSGSGTDFTLTISSLQPEDFATYYCQQANSFPPTFGGGTKVEIK

|||||||||||||||||||||||||||||||||||||||||||||.||||||.||||||||||||||||||||||||||||||||||||||||||||||.||..|||

DIQMTQSPSSVSASVGDRVTITCRASQGISGWLAWYQQKPGKAPKLLIYAASSLQSGVPSRFSGSGSGTDFTLTISSLQPEDFATYYCQQANSFPPTFGQGTRLEIK

^^^^^^ ^^^ ^^^^^^^^^

Best Alignment of Enfortumab CDR-H3 to a sequence from OAS

EVQLVESGGGLVQPGGSLRLSCAASGFTF--SSYNMNWVRQAPGKGLEWVSYISSSSSTIYYADSVKGRFTISRDNAKNSLSLQMNSLRDEDTAVYYCARAYYYGMDVWGQGTTVTVSS

.................|.|.|..||.......|...|.||.||.||||..||..|....||..|.|.|.|||.|..||..||...|....||||||||||||||||||||||||||||

--------------SETLSLTCTVSGGSISSGDYYWSWIRQPPGTGLEWIGYIYYS-GSTYYNPSLKSRVTISVDTSKNQFSLKLSSVTAADTAVYYCARAYYYGMDVWGQGTTVTVSS

^^^^^^^^^^ ^^^^^^^^ ^^^^^^^^^^

Therapeutic : Enokizumab

Best Alignment of Enokizumab heavy chain to a sequence from OAS

QVQLVQSGAEVKKPGSSVKVSCKASGGTFSYYWIEWVRQAPGQGLEWMGEILPGSGTTNPNEKFKGRVTITADESTSTAYMELSSLRSEDTAVYYCARADYYGS-DYVKFDYWGQGTLVTVSS

||||||||||||||||||||||||||||||.|.|.||||||||||||||.|.|..||.|...||.|||||||||||||||||||||||||||||||||||||.|..||.||||||||||||||

QVQLVQSGAEVKKPGSSVKVSCKASGGTFSSYAISWVRQAPGQGLEWMGRIIPIFGTANYAQKFQGRVTITADESTSTAYMELSSLRSEDTAVYYCARADYYDSSGYVFFDYWGQGTLVTVSS

^^^^^^^^ ^^^^^^^^ ^^^^^^^^^^^^^^^^

Best Alignment of Enokizumab light chain to a sequence from OAS

DIQMTQSPSSLSASVGDRVTITCKASQHVITHVTWYQQKPGKAPKLLIYGTSYSYSGVPSRFSGSGSGTDFTLTISSLQPEDFATYYCQQFYEYPLTFGGGTKVEIK

|||||||||||||||||||||||.|||.|.|.|.|||.|||||||||||..|...||||||||||||||||||||||||||||||||||||..||||||||||||||

DIQMTQSPSSLSASVGDRVTITCQASQSVSTWVAWYQHKPGKAPKLLIYKASSLQSGVPSRFSGSGSGTDFTLTISSLQPEDFATYYCQQFNSYPLTFGGGTKVEIK

^^^^^^ ^^^ ^^^^^^^^^

Best Alignment of Enokizumab heavy chain CDRs to a sequence from OAS

QVQLVQSGAEVKKPGSSVKVSCKASGGTFSYYWIEWVRQAPGQGLEWMGEILPGSGTTNPNEKFKGRVTITADESTSTAYMELSSLRSEDTAVYYCARADYYGSDYVKFDYWGQGTLVTVSS

.|||.|||||..|||.|||.||||.|.|||.||||||.|.||.||||.||||||||.||.||||||..|.|||.|..||||.||||.|||.|||||||.|||||.||.||||||||..||||

-VQLQQSGAELMKPGASVKISCKATGYTFSSYWIEWVKQRPGHGLEWIGEILPGSGSTNYNEKFKGKATFTADTSSNTAYMQLSSLTSEDSAVYYCARPDYYGSSYVSFDYWGQGTTLTVSS

^^^^^^^^ ^^^^^^^^ ^^^^^^^^^^^^^^^

Best Alignment of Enokizumab light chain CDRs to a sequence from OAS

DIQMTQSPSSLSASVGDRVTITCKASQHVITHVTWYQQKPGKAPKLLIYGTSYSYSGVPSRFSGSGSGTDFTLTISSLQPEDFATYYCQQFYEYPLTFGGGTKVEIK

|||||.|.|||.|||||||||.|.|||.|||...||||.||||||||..|.|....|||||||||||..||||||.|.||||||||.|||.|.||||.||||.||||

DIQMTHSSSSLAASVGDRVTIACRASQNVITYLAWYQQTPGKAPKLLLSGASTLQGGVPSRFSGSGSESDFTLTITSVQPEDFATYFCQQTYAYPLTCGGGTRVEIK

^^^^^^ ^^^ ^^^^^^^^^

Best Alignment of Enokizumab CDR-H3 to a sequence from OAS

QVQLVQSGAEVKKPGSSVKVSCKASGGTFSYYWIEWVRQAPGQGLEWMGEILPGSGTTNPNEKFKGRVTITADESTSTAYMELSSLRSEDTAVYYCARADYYGSDYVKFDYWGQGTLVTVSS

.......|||..|||.|||.||||||.||..||..||.|.|||||||.|||.|....||.|.||||..|.|.|.|.|||||.||||.|||.|||||||||||||.||.||||||||..||||

-------GAELVKPGASVKLSCKASGYTFTSYWMQWVKQRPGQGLEWIGEIDPSDSYTNYNQKFKGKATLTVDTSSSTAYMQLSSLTSEDSAVYYCARADYYGSSYVGFDYWGQGTTLTVSS

^^^^^^^^ ^^^^^^^^ ^^^^^^^^^^^^^^^

Therapeutic : Ensituximab

Best Alignment of Ensituximab heavy chain to a sequence from OAS

QVQLKESGPDLVAPSQSLSITCTVSGFSLSKFGVNWVRQPPGKGLEWLGVIWGDGSTSYNSGLISRLSISKENSKSQVFLKLNSLQADDTATYYCVKPGGDYWGHGTSVTVSS

|||||||||.|||||||||||||||||||...||.||||||||||||||||||||||.|.|.||||||||||||||||||||||||.||||||||.||||||||.||||||||

QVQLKESGPGLVAPSQSLSITCTVSGFSLTSYGVSWVRQPPGKGLEWLGVIWGDGSTNYHSALISRLSISKENSKSQVFLKLNSLQTDDTATYYCAKPGGDYWGQGTSVTVSS

^^^^^^^^ ^^^^^^^ ^^^^^^^

Best Alignment of Ensituximab light chain to a sequence from OAS

QVVLTQSPVIMSASPGEKVTMTCSASSSISYMYWYQQKPGTSPKRWIYDTSKLASGVPARFSGSGSGTSYSLTISNMEAGDAATYYCHQRDSYPWTFGGGTNLEIK

|.||||||.||||||||||||||||||||||||||||||||||||||||||||||||||||||||||||||||||.|||.||||||||||.||||||||||.||||

QIVLTQSPAIMSASPGEKVTMTCSASSSISYMYWYQQKPGTSPKRWIYDTSKLASGVPARFSGSGSGTSYSLTISSMEAEDAATYYCHQRSSYPWTFGGGTKLEIK

^^^^^ ^^^ ^^^^^^^^^

Best Alignment of Ensituximab heavy chain CDRs to a sequence from OAS

QVQLKESGPDLVAPSQSLSITCTVSGFSLSKFGVNWVRQPPGKGLEWLGVIWGDGSTSYNSGLISRLSISKENSKSQVFLKLNSLQADDTATYYCVKPGGDYWGHGTSVTVSS

.|.|.||||.|||||||||||||||||||...||.||||||||||||||||||||||.|.|.|||||||||.||||||||||||||.|.||||||.||||||||.||||||||

-VKLVESGPGLVAPSQSLSITCTVSGFSLTSYGVSWVRQPPGKGLEWLGVIWGDGSTNYHSALISRLSISKDNSKSQVFLKLNSLQTDVTATYYCAKPGGDYWGQGTSVTVSS

^^^^^^^^ ^^^^^^^ ^^^^^^^

Best Alignment of Ensituximab light chain CDRs to a sequence from OAS

QVVLTQSPVIMSASPGEKVTMTCSASSSISYMYWYQQKPGTSPKRWIYDTSKLASGVPARFSGSGSGTSYSLTISNMEAGDAATYYCHQRDSYPWTFGGGTNLEIK

|.||||||.|||||.|||||||||||||||||.||||||||||||||||||||||||||||||||||||||||||.|||.||||||||||.||||||||||.||||

QIVLTQSPAIMSASTGEKVTMTCSASSSISYMHWYQQKPGTSPKRWIYDTSKLASGVPARFSGSGSGTSYSLTISSMEAEDAATYYCHQRSSYPWTFGGGTKLEIK

^^^^^ ^^^ ^^^^^^^^^

Best Alignment of Ensituximab CDR-H3 to a sequence from OAS

QVQLKESGPDLVAPSQSLSITCTVSGFSLSKFGVNWVRQPPGKGLEWLGVIWGD---GSTSYNSGLISRLSISKENSKSQVFLKLNSLQADDTATYYCVKPGGDYWGHGTSVTVSS

.|||.|||..||.|..||...|..||||......|||||.|||||||...|........|.|......|..|....|.|...|..|.|...|||.||||.|||||||.||||||||

EVQLVESGGGLVQPKGSLKLSCAASGFSFNTYAMNWVRQAPGKGLEWVARIRSKSNKYATYYADSVKDRFTIYRDDSESMLYLQMNNLKTEDTAMYYCVSPGGDYWGQGTSVTVSS

^^^^^^^^ ^^^^^^^^^^ ^^^^^^^

Therapeutic : Epratuzumab

Best Alignment of Epratuzumab heavy chain to a sequence from OAS

QVQLVQSGAEVKKPGSSVKVSCKASGYTFTSYWLHWVRQAPGQGLEWIGYINPRNDYTEYNQNFKDKATITADESTNTAYMELSSLRSEDTAFYFCARRDITTFYWGQGTTVTVSS

||||.|||||..|||.|||.|||||||||||||.|||.|.|||||||||||||...||||||.||||||.|||.|..||||.||||.|||.|.|.|||.||||.|||||||.||||

QVQLKQSGAELAKPGASVKMSCKASGYTFTSYWMHWVKQRPGQGLEWIGYINPSTGYTEYNQKFKDKATLTADKSSSTAYMQLSSLTSEDSAVYYCARSDITTHYWGQGTTLTVSS

^^^^^^^^ ^^^^^^^^ ^^^^^^^^^

Best Alignment of Epratuzumab light chain to a sequence from OAS

DIQLTQSPSSLSASVGDRVTMSCKSSQSVLYSANHKNYLAWYQQKPGKAPKLLIYWASTRESGVPSRFSGSGSGTDFTFTISSLQPEDIATYYCHQYLSSWTFGGGTKLEIK

.|..|||||||..|.||.||||||||||||||.|.||||||||||||.|||||||||||||||||.||.|||||||||.||||.|.||.|.|||||||||||||||||||||

NIMMTQSPSSLAVSAGDKVTMSCKSSQSVLYSSNQKNYLAWYQQKPGQAPKLLIYWASTRESGVPDRFTGSGSGTDFTLTISSVQAEDLAVYYCHQYLSSWTFGGGTKLEIK

^^^^^^^^^^^^ ^^^ ^^^^^^^^

Best Alignment of Epratuzumab heavy chain CDRs to a sequence from OAS

QVQLVQSGAEVKKPGSSVKVSCKASGYTFTSYWLHWVRQAPGQGLEWIGYINPRNDYTEYNQNFKDKATITADESTNTAYMELSSLRSEDTAFYFCARRDITTFYWGQGTTVTVSS

.|||.|||||...||.|||.|||||||||||||.|||.|.|||||||||.|||...||.|.|.||.|||.|.|.|..||||.||||.|||.|.|.|||||.||||||||||.||||

-VQLQQSGAELVRPGTSVKLSCKASGYTFTSYWMHWVKQRPGQGLEWIGVINPSDSYTNYKQKFKGKATLTVDTSSSTAYMQLSSLTSEDSAVYYCARRDTTTFYWGQGTTLTVSS

^^^^^^^^ ^^^^^^^^ ^^^^^^^^^

Best Alignment of Epratuzumab light chain CDRs to a sequence from OAS

DIQLTQSPSSLSASVGDRVTMSCKSSQSVLYSANHKNYLAWYQQKPGKAPKLLIYWASTRESGVPSRFSGSGSGTDFTFTISSLQPEDIATYYCHQYLSSWTFGGGTKLEIK

.|..|||||||..|.|..||||||||||||||||.|||.|||||||...||||||||||||||||.||.|||||||||.||||.|.||.|.|||||||||||||||||||||

NIMMTQSPSSLAVSAGEKVTMSCKSSQSVLYSANQKNYVAWYQQKPAQSPKLLIYWASTRESGVPDRFTGSGSGTDFTLTISSVQAEDLAVYYCHQYLSSWTFGGGTKLEIK

^^^^^^^^^^^^ ^^^ ^^^^^^^^

Best Alignment of Epratuzumab CDR-H3 to a sequence from OAS

QVQLVQSGAEVKKPGSSVKVSCKASGYTFTSYWLHWVRQAPGQGLEWIGYINPRNDYTEYNQNFKDKATITADESTNTAYMELSSLRSEDTAFYFCARRDITTFYWGQGTTVTVSS

||||.|.|||..|||.|||.|||||||||||||..||.|.|||||||||.|.|....|.||..||.|||.|.|.|..||||.||||.|||.|.|.||||||||.|||||||.||||

QVQLKQPGAELVKPGASVKMSCKASGYTFTSYWITWVKQRPGQGLEWIGDIYPGSGSTNYNEKFKSKATLTVDTSSSTAYMQLSSLTSEDSAVYYCARRDITTDYWGQGTTLTVSS

^^^^^^^^ ^^^^^^^^ ^^^^^^^^^

Therapeutic : Eptinezumab

Best Alignment of Eptinezumab heavy chain to a sequence from OAS

EVQLVESGGGLVQPGGSLRLSCAVSGIDLSGYYMNWVRQAPGKGLEWVGVIGINGATYYASWAKGRFTISRDNSKTTVYLQMNSLRAEDTAVYFCARG---------DIWGQGTLVTVSS

||||||||||||||||||||||||||...|..||||||||||||||||.||...|.||||...||||||||||||.|.|||||||||||||||.||||.........|.|||||||||||

EVQLVESGGGLVQPGGSLRLSCAVSGFSVSSNYMNWVRQAPGKGLEWVSVIYSGGSTYYADSVKGRFTISRDNSKNTLYLQMNSLRAEDTAVYYCARGAGIVVVIHFDYWGQGTLVTVSS

^^^^^^^^ ^^^^^^^ ^^^^^^^^^^^^^^

Best Alignment of Eptinezumab light chain to a sequence from OAS

-QVLTQSPSSLSASVGDRVTINCQASQSVYHNTYLAWYQQKPGKVPKQLIYDASTLASGVPSRFSGSGSGTDFTLTISSLQPEDVATYYCLGSYDCTNGDCFVFGGGTKVEIK

....|||||||||||||||||.|.||||.....||||||||||||||.|||.||||.|||||||||||||||||||||||||||.|||||..|||........||||||||||

DIQMTQSPSSLSASVGDRVTITCRASQSI--SNYLAWYQQKPGKVPKLLIYAASTLQSGVPSRFSGSGSGTDFTLTISSLQPEDFATYYCQQSYD----TPLTFGGGTKVEIK

^^^^^^^^ ^^^ ^^^^^^^^^^^^^

Best Alignment of Eptinezumab heavy chain CDRs to a sequence from OAS

EVQLVESGGGLVQPGGSLRLSCAVSGIDLSGYYMNWVRQAPGKGLEWVGVIGINGATYYASWAKGRFTISRDNSKTTVYLQMNSLRAEDTAVYFCARGDIWGQGTLVTVSS

..||...|.||..|...|.|.|.|.|..|||||..|.||.|||||||.|.|...|.|.|......|.|||.|.||....|...|..|.|||||.|||||||||||.|||||

QMQLQQWGAGLLKPSETLSLTCGVHGGSLSGYYWSWIRQPPGKGLEWIGEINHSGSTNYNPSLESRVTISVDTSKNQFSLKLSSVTAADTAVYYCARGDIWGQGTMVTVSS

^^^^^^^^ ^^^^^^^ ^^^^^

Best Alignment of Eptinezumab light chain CDRs to a sequence from OAS

-QVLTQSPSSLSASVGDRVTINCQASQSVYHNTYLAWYQQKPGKVPKQLIYDASTLASGVPSRFSGSGSGTDFTLTISSLQPEDVATYYCLGSYDCTNGDCFVFGGGTKVEIK

..||||||..||.|.|.|.|..|.||||.....||||||..||..|..|||.||..|.|.|.||||||||||||||||||||||.|||||..||.........||.|||.|||

EIVLTQSPGTLSLSPGERATLSCRASQSLETSNYLAWYQKRPGQAPRLLIYNASSRATGIPDRFSGSGSGTDFTLTISSLQPEDFATYYCQQSYSTPPSIGYTFGQGTKLEIK

^^^^^^^^ ^^^ ^^^^^^^^^^^^^

Best Alignment of Eptinezumab CDR-H3 to a sequence from OAS

EVQLVESGGGLVQPGGSLRLSCAVSGIDLSGYYMNWVRQAPGKGLEWVGVIGIN-GATYYASWAKGRFTISRDNSKTTVYLQMNSLRAEDTAVYFCARGDIWGQGTLVTVSS

..............|.||..|||.||...|.|.|.|||||||||||||..|.......||....||||||||||.|...|||||||||||||||.|||||||||||.|||||

--------------GESLKISCAASGFTFSSYWMSWVRQAPGKGLEWVANIKQDGSEKYYVDSVKGRFTISRDNAKNSLYLQMNSLRAEDTAVYYCARGDIWGQGTMVTVSS

^^^^^^^^ ^^^^^^^^ ^^^^^

Therapeutic : Erenumab

Best Alignment of Erenumab heavy chain to a sequence from OAS

QVQLVESGGGVVQPGRSLRLSCAASGFTFSSFGMHWVRQAPGKGLEWVAVISFD-GSIKYSVDSVKGRFTISRDNSKNTLFLQMNSLRAEDTAVYYCARDRLNYYDSSGYYHYKYYGMAVWGQGTTVTVSS

|||||||||||||||||||||||||||||||.||||||||||||||||||||.|.....|..||||||||||||||||||.|||||||||||||||||||...||||||||.|.||||.||||||||||||

QVQLVESGGGVVQPGRSLRLSCAASGFTFSSYGMHWVRQAPGKGLEWVAVISYDGSNKYY-ADSVKGRFTISRDNSKNTLYLQMNSLRAEDTAVYYCARDLPYYYDSSGYYLYYYYGMDVWGQGTTVTVSS

^^^^^^^^ ^^^^^^^^ ^^^^^^^^^^^^^^^^^^^^^^^

Best Alignment of Erenumab light chain to a sequence from OAS

QSVLTQPPSVSAAPGQKVTISCSGSSSNIGNNYVSWYQQLPGTAPKLLIYDNNKRPSGIPDRFSGSKSGTSTTLGITGLQTGDEADYYCGTWDSRLSAVVFGGGTKLTVL

||||||||||||||||||||||||||||||||||||||||||||||||||||||||||||||||||||||||||||||||||||||||||||||.|||||||||||||||

QSVLTQPPSVSAAPGQKVTISCSGSSSNIGNNYVSWYQQLPGTAPKLLIYDNNKRPSGIPDRFSGSKSGTSTTLGITGLQTGDEADYYCGTWDSSLSAVVFGGGTKLTVL

^^^^^^^^ ^^^ ^^^^^^^^^^^

Best Alignment of Erenumab heavy chain CDRs to a sequence from OAS

QVQLVESGGGVVQPGRSLRLSCAASGFTFSSFGMHWVRQAPGKGLEWVAVISFDGSIKYSVDSVKGRFTISRDNSKNTLFLQMNSLRAEDTAVYYCARDRLNYYDSSGYYHYKYYGMAVWGQGTTVTVSS

..............|.||||||||||||.||..|.|||||||||||||.||...||..|..||||||||||||||||||.|||||||||||||||||||||.||||||||.|.||||.||||||||||||

--------------GGSLRLSCAASGFTVSSNYMSWVRQAPGKGLEWVSVIYSGGSTYY-ADSVKGRFTISRDNSKNTLYLQMNSLRAEDTAVYYCARDRLYYYDSSGYYYYYYYGMDVWGQGTTVTVSS

^^^^^^^^ ^^^^^^^ ^^^^^^^^^^^^^^^^^^^^^^^

Best Alignment of Erenumab light chain CDRs to a sequence from OAS

QSVLTQPPSVSAAPGQKVTISCSGSSSNIGNNYVSWYQQLPGTAPKLLIYDNNKRPSGIPDRFSGSKSGTSTTLGITGLQTGDEADYYCGTWDSRLSAVVFGGGTKLTVL

|||||||||||||||||||||||||||||||||||||||||||||||||||||||||||||||||||||||.||||.||||||||||||||||||||||||||||||||.

QSVLTQPPSVSAAPGQKVTISCSGSSSNIGNNYVSWYQQLPGTAPKLLIYDNNKRPSGIPDRFSGSKSGTSATLGIAGLQTGDEADYYCGTWDSRLSAVVFGGGTKLTVV

^^^^^^^^ ^^^ ^^^^^^^^^^^

Best Alignment of Erenumab CDR-H3 to a sequence from OAS

QVQLVESGGGVVQPGRSLRLSCAASGFTFSSFGMHWVRQAPGKGLEWVAVISFDGSIKYSVDSVKGRFTISRDNSKNTLFLQMNSLRAEDTAVYYCARDRLNYYDSSGYYHYKYYGMAVWGQGTTVTVSS

..............|.||||||||||||.||..|.|||||||||||||.||...||..|..||||||||||||||||||.|||||||||||||||||||||.||||||||.|.||||.||||||||||||

--------------GGSLRLSCAASGFTVSSNYMSWVRQAPGKGLEWVSVIYSGGSTYY-ADSVKGRFTISRDNSKNTLYLQMNSLRAEDTAVYYCARDRLYYYDSSGYYYYYYYGMDVWGQGTTVTVSS

^^^^^^^^ ^^^^^^^ ^^^^^^^^^^^^^^^^^^^^^^^

Therapeutic : Etaracizumab

Best Alignment of Etaracizumab heavy chain to a sequence from OAS

QVQLVESGGGVVQPGRSLRLSCAASGFTFSSYDMSWVRQAPGKGLEWVAKVSSGGGSTYYLDTVQGRFTISRDNSKNTLYLQMNSLRAEDTAVYYCARHLH-----GSFASWGQGTTVTVSS

||||||||||||||||||||||||||||||||.|.||||||||||||||...||.|||||.|.|.|||||||||||||||||||||||||||||||||||......|.|..|||||.|||||

QVQLVESGGGVVQPGRSLRLSCAASGFTFSSYGMHWVRQAPGKGLEWVAVIYSG-GSTYYADSVKGRFTISRDNSKNTLYLQMNSLRAEDTAVYYCARHLPAAIGMGAFDIWGQGTMVTVSS

^^^^^^^^ ^^^^^^^^ ^^^^^^^^^^^^^^^

Best Alignment of Etaracizumab light chain to a sequence from OAS

EIVLTQSPATLSLSPGERATLSCQASQSISNFLHWYQQRPGQAPRLLIRYRSQSISGIPARFSGSGSGTDFTLTISSLEPEDFAVYYCQQSGS-WPLTFGGGTKVEIK

|||||||||||||||||||||||.|||||.|||.||||||||||||||...|....||||||||||||||||||||||||||||||||||.||..|||||||||||||

EIVLTQSPATLSLSPGERATLSCRASQSIINFLAWYQQRPGQAPRLLIYDASNRAIGIPARFSGSGSGTDFTLTISSLEPEDFAVYYCQQYGSSPPLTFGGGTKVEIK

^^^^^^ ^^^ ^^^^^^^^^^

Best Alignment of Etaracizumab heavy chain CDRs to a sequence from OAS

QVQLVESGGGVVQPGRSLRLSCAASGFTFSSYDMSWVRQAPGKGLEWVAKVSSGGGSTYYLDTVQGRFTISRDNSKNTLYLQMNSLRAEDTAVYYCARHLHGSFASWGQGTTVTVSS

.||.||||||.|.||.||.||||||||.|||||||||||.|.|.|||||.||||||||||.|||.|||||||||.||||||||.||..||||.||||||..||||.|||||.||||.

EVQRVESGGGLVKPGGSLKLSCAASGFAFSSYDMSWVRQTPEKRLEWVAYVSSGGGSTYYPDTVKGRFTISRDNAKNTLYLQMSSLKSEDTAMYYCARHEDGSFAYWGQGTLVTVSA

^^^^^^^^ ^^^^^^^^ ^^^^^^^^^^

Best Alignment of Etaracizumab light chain CDRs to a sequence from OAS

EIVLTQSPATLSLSPGERATLSCQASQSISNFLHWYQQRPGQAPRLLIRYRSQSISGIPARFSGSGSGTDFTLTISSLEPEDFAVYYCQQSGSWPLTFGGGTKVEIK

.||.||||||||..||.|..|||.||||||||||||||.....||.||.|.||||.|.|.|||||||||||||.|.|.|.|||..|.||||.|||||||.|||.|.|

DIVMTQSPATLSVTPGDRVSLSCRASQSISNFLHWYQQKSHESPRFLIKYASQSIPGTPSRFSGSGSGTDFTLSINSVETEDFGIYFCQQSNSWPLTFGAGTKLELK

^^^^^^ ^^^ ^^^^^^^^^

Best Alignment of Etaracizumab CDR-H3 to a sequence from OAS

QVQLVESGGGVVQPGRSLRLSCAASGFTFSSYDMSWVRQAPGKGLEWVAKVSSGGGSTYYLDTVQGRFTISRDNSKNTLYLQMNSLRAEDTAVYYCARHLHGSFASWGQGTTVTVSS

.|.||||||..|.||.||.|||.|||||||.|.||||||.|.|.|||||..||.|......|.|.|||||||||..|||||||.|||.|||..|||||||.||||||||.|.||.|.

EVELVESGGDLVRPGGSLKLSCSASGFTFSTYSMSWVRQTPDKRLEWVATISSAGDYIFFPDSVKGRFTISRDNARNTLYLQMSSLRSEDTTMYYCARHLTGSFASWGQATLVTGSA

^^^^^^^^ ^^^^^^^^ ^^^^^^^^^^

Therapeutic : Etrolizumab

Best Alignment of Etrolizumab heavy chain to a sequence from OAS

EVQLVESGGGLVQPGGSLRLSCAASGFFITNNYWGWVRQAPGKGLEWVGYISYS-GSTSYNPSLKSRFTISRDTSKNTFYLQMNSLRAEDTAVYYCARTGSSGYFDFWGQGTLVTVSS

|||||||||||||||||||||||||||...|||..|||||||.|||||..||.|.|||.|..|.|.|||||||.||||.|||||||||||||||||||.|||.|||.|||||||||||

EVQLVESGGGLVQPGGSLRLSCAASGFTVSNNYMSWVRQAPGRGLEWVSAISGSGGSTYYADSVKGRFTISRDNSKNTLYLQMNSLRAEDTAVYYCAREGSSWYFDYWGQGTLVTVSS

^^^^^^^^ ^^^^^^^^ ^^^^^^^^^^^

Best Alignment of Etrolizumab light chain to a sequence from OAS

DIQMTQSPSSLSASVGDRVTITCRASESVDDLLHWYQQKPGKAPKLLIKYASQSISGVPSRFSGSGSGTDFTLTISSLQPEDFATYYCQQGNSLPNTFGQGTKVEIK

|||||||||||||||||||||||||||.|...||||||||||||||||..||...|||||||||||||||||||||||||||||||||||.||||.|||||||||||

DIQMTQSPSSLSASVGDRVTITCRASENVNNYLHWYQQKPGKAPKLLIYKASTLQSGVPSRFSGSGSGTDFTLTISSLQPEDFATYYCQQYNSLPRTFGQGTKVEIK

^^^^^^ ^^^ ^^^^^^^^^

Best Alignment of Etrolizumab heavy chain CDRs to a sequence from OAS

EVQLVESGGGLVQPGGSLRLSCAASGFFITNNYWGWVRQAPGKGLEWVGYISYSGSTSYNPSLKSRFTISRDTSKNTFYLQMNSLRAEDTAVYYCARTGSSGYFDFWGQGTLVTVSS

||||.|||.||..|...|.|.|...|..||..||.|.|..||..||..|||||||||.||||||||..|.||||||..|||.||...||||.|||||.|||||||.|||||..||||

EVQLQESGPGLAKPSQTLSLTCSVTGYSITSDYWNWIRKFPGNKLEYMGYISYSGSTYYNPSLKSRISITRDTSKNQYYLQLNSVTTEDTATYYCARWGSSGYFDYWGQGTTLTVSS

^^^^^^^^ ^^^^^^^ ^^^^^^^^^^^

Best Alignment of Etrolizumab light chain CDRs to a sequence from OAS

DIQMTQSPSSLSASVGDRVTITCRASESVDDLLHWYQQKPGKAPKLLIKYASQSISGVPSRFSGSGSGTDFTLTISSLQPEDFATYYCQQGNSLPNTFGQGTKVEIK

|||||||||.||.|||||||||||||.|||.||.|||.|||.||.|||..||...||||||||||||||.||||||||||.|.|||||||.||.|||||||||..||

DIQMTQSPSTLSVSVGDRVTITCRASQSVDSLLAWYQHKPGRAPTLLIYKASSLESGVPSRFSGSGSGTEFTLTISSLQPDDVATYYCQQYNSYPNTFGQGTKLDIK

^^^^^^ ^^^ ^^^^^^^^^

Best Alignment of Etrolizumab CDR-H3 to a sequence from OAS

EVQLVESGGGLVQPGGSLRLSCAASGFFITNNYWGWVRQAPGKGLEWVGYISYS-GSTSYNPSLKSRFTISRDTSKNTFYLQMNSLRAEDTAVYYCARTGSSGYFDFWGQGTLVTVSS

||||||||||.||||.||.|||.||||...|....|.|||||||||||..||.....|.|..|.|.||..|||..|.|.|||.||||.||||.||||||||||||||||.||.|||||

EVQLVESGGGPVQPGRSLKLSCVASGFIFSNYWMTWIRQAPGKGLEWVASISNTGDNTYYSDSVKGRFSLSRDNAKSTLYLQVNSLRSEDTATYYCARTGSSGYFDFWGPGTMVTVSS

^^^^^^^^ ^^^^^^^^ ^^^^^^^^^^^

Therapeutic : Evinacumab

Best Alignment of Evinacumab heavy chain to a sequence from OAS

EVQLVESGGGVIQPGGSLRLSCAASGFTFDDYAMNWVRQGPGKGLEWVSAISGDGGSTYYADSVKGRFTISRDNSKNSLYLQMNSLRAEDTAFFYCAKDLRNTIFGVVIPDAFDIWGQGTMVTVSS

|||||||||||.||||||||||||||||||||||.||||.|||||||||.|||||||||||||||||||||||||||||||||||||.||||..||||||..|....|.|.|||||||||||||||

EVQLVESGGGVVQPGGSLRLSCAASGFTFDDYAMHWVRQAPGKGLEWVSLISGDGGSTYYADSVKGRFTISRDNSKNSLYLQMNSLRTEDTALYYCAKDLLTTV---VTPYAFDIWGQGTMVTVSS

^^^^^^^^ ^^^^^^^^ ^^^^^^^^^^^^^^^^^^^

Best Alignment of Evinacumab light chain to a sequence from OAS

DIQMTQSPSTLSASVGDRVTITCRASQSIRSWLAWYQQKPGKAPKLLIYKASSLESGVPSRFSGSGSGTEFTLTISSLQPDDFATYYCQQYNSYSYTFGQGTKLEIK

|||||||||||||||||||||||||||||||||||||||||||||||||||||||||||||||||||||||||||||||||||||||||||||||||||||||||||

DIQMTQSPSTLSASVGDRVTITCRASQSIRSWLAWYQQKPGKAPKLLIYKASSLESGVPSRFSGSGSGTEFTLTISSLQPDDFATYYCQQYNSYSYTFGQGTKLEIK

^^^^^^ ^^^ ^^^^^^^^^

Best Alignment of Evinacumab heavy chain CDRs to a sequence from OAS

EVQLVESGGGVIQPGGSLRLSCAASGFTFDDYAMNWVRQGPGKGLEWVSAISGDGGSTYYADSVKGRFTISRDNSKNSLYLQMNSLRAEDTAFFYCAKDLRNTIFGVVIPDAFDIWGQGTMVTVSS

............||||||||||||||||||||||.||||.|||||||||.|||||||||||||||||||||||||||||||||||||.||||..|||||.|.|||||||.||||||||||||||||

------------QPGGSLRLSCAASGFTFDDYAMHWVRQAPGKGLEWVSLISGDGGSTYYADSVKGRFTISRDNSKNSLYLQMNSLRTEDTALYYCAKDYRGTIFGVVIMDAFDIWGQGTMVTVSS

^^^^^^^^ ^^^^^^^^ ^^^^^^^^^^^^^^^^^^^

Best Alignment of Evinacumab light chain CDRs to a sequence from OAS

DIQMTQSPSTLSASVGDRVTITCRASQSIRSWLAWYQQKPGKAPKLLIYKASSLESGVPSRFSGSGSGTEFTLTISSLQPDDFATYYCQQYNSYSYTFGQGTKLEIK

|||||.|.|||||||||||||||||||||||||||||||||||||||||||||||||||||||||||||||||||||||||||||||||||||||||||||||||||

DIQMTPSTSTLSASVGDRVTITCRASQSIRSWLAWYQQKPGKAPKLLIYKASSLESGVPSRFSGSGSGTEFTLTISSLQPDDFATYYCQQYNSYSYTFGQGTKLEIK

^^^^^^ ^^^ ^^^^^^^^^

Best Alignment of Evinacumab CDR-H3 to a sequence from OAS

EVQLVESGGGVIQPGGSLRLSCAASGFTFDDYAMNWVRQGPGKGLEWVSAISGDGGSTYYADSVKGRFTISRDNSKNSLYLQMNSLRAEDTAFFYCAKDLRNTIFGVVIPDAFDIWGQGTMVTVSS

..............|||||.|||||||||..|.|.||||.||||||||..||.||...|||||||||||||||||||.||||||||||||||..|||||||.||||||||||||||||||||||||

--------------GGSLRPSCAASGFTFSSYGMHWVRQAPGKGLEWVAVISYDGSNKYYADSVKGRFTISRDNSKNTLYLQMNSLRAEDTAVYYCAKDLRITIFGVVIPDAFDIWGQGTMVTVSS

^^^^^^^^ ^^^^^^^^ ^^^^^^^^^^^^^^^^^^^

Therapeutic : Evolocumab

Best Alignment of Evolocumab heavy chain to a sequence from OAS

EVQLVQSGAEVKKPGASVKVSCKASGYTLTSYGISWVRQAPGQGLEWMGWVSFYNGNTNYAQKLQGRGTMTTDPSTSTAYMELRSLRSDDTAVYYCARGY------------GMDVWGQGTTVTVSS

.|||||||||||||||||||||||||||.|||||||||||||||||||||.|.||||||||||||||.||||||||||||||||||||||||||||||||............|||||||||||||||

QVQLVQSGAEVKKPGASVKVSCKASGYTFTSYGISWVRQAPGQGLEWMGWISAYNGNTNYAQKLQGRVTMTTDPSTSTAYMELRSLRSDDTAVYYCARGYYGSGSYSRYYYYGMDVWGQGTTVTVSS

^^^^^^^^ ^^^^^^^^ ^^^^^^^^^^^^^^^^^^^^

Best Alignment of Evolocumab light chain to a sequence from OAS

ESALTQPASVSGSPGQSITISCTGTSSDVGGYNSVSWYQQHPGKAPKLMIYEVSNRPSGVSNRFSGSKSGNTASLTISGLQAEDEADYYCNSYTS--TSMVFGGGTKLTVL

.||||||||||||||||||||||||||||||||.||||||||||||||||||||||||||||||||||||||||||||||||||||||||.||||..||||||||||||||

QSALTQPASVSGSPGQSITISCTGTSSDVGGYNYVSWYQQHPGKAPKLMIYEVSNRPSGVSNRFSGSKSGNTASLTISGLQAEDEADYYCSSYTSSSTSMVFGGGTKLTVL

^^^^^^^^^ ^^^ ^^^^^^^^^^^

Best Alignment of Evolocumab heavy chain CDRs to a sequence from OAS

EVQLVQSGAEVKKPGASVKVSCKASGYTLTSYGISWVRQAPGQGLEWMGWVSFYNGNTNYAQKLQGRGTMTTDPSTSTAYMELRSLRSDDTAVYYCARGYGMDVWGQGTTVTVSS

..............||||||||||||||||||||||||||||||||||||.|.||||||||||||||.|||||.|||||||||||||||||||||||||||||||||||.||||.

--------------GASVKVSCKASGYTLTSYGISWVRQAPGQGLEWMGWISAYNGNTNYAQKLQGRVTMTTDTSTSTAYMELRSLRSDDTAVYYCARGYGMDVWGQGTLVTVS-

^^^^^^^^ ^^^^^^^^ ^^^^^^^^

Best Alignment of Evolocumab light chain CDRs to a sequence from OAS

ESALTQPASVSGSPGQSITISCTGTSSDVGGYNSVSWYQQHPGKAPKLMIYEVSNRPSGVSNRFSGSKSGNTASLTISGLQAEDEADYYCNSYTSTSMVFGGGTKLTVL

..||||||.|||||.|.||.|..|.||||||||.||||||||||||.||||||||||||||||||.|||||||||||||||||||||||||||||.|||||||||||||

QPALTQPATVSGSPAQAITTSFPGSSSDVGGYNYVSWYQQHPGKAPILMIYEVSNRPSGVSNRFSASKSGNTASLTISGLQAEDEADYYCNSYTSSSMVFGGGTKLTVL

^^^^^^^^^ ^^^ ^^^^^^^^^

Best Alignment of Evolocumab CDR-H3 to a sequence from OAS

EVQLVQSGAEVKKPGASVKVSCKASGYTLTSYGISWVRQAPGQGLEWMGWVSFYNGNTNYAQKLQGRGTMTTDPSTSTAYMELRSLRSDDTAVYYCARGYGMDVWGQGTTVTVSS

..............|.|.|.||.|||.|..||...|||||.|.||||........|.|.|.....||.|..........|....|||..||||||||||||||||||||||||||

--------------GESLKISCAASGFTFSSYDMHWVRQATGKGLEWVSAIGTA-GDTYYPGSVKGRFTISRENAKNSLYLQMNSLRAGDTAVYYCARGYGMDVWGQGTTVTVSS

^^^^^^^^ ^^^^^^^^ ^^^^^^^^

Therapeutic : Farletuzumab

Best Alignment of Farletuzumab heavy chain to a sequence from OAS

EVQLVESGGGVVQPGRSLRLSCSASGFTFSGYGLSWVRQAPGKGLEWVAMISSGGSYTYYADSVKGRFAISRDNAKNTLFLQMDSLRPEDTGVYFCARHGDD---------PAWFAYWGQGTPVTVSS

.|||||||||||||||||||||.|||||||.|||.||||||||||||||.||..||..||||||||||.|||||.||||.|||.|||.|||.||.|||.||..........|..|.||||||.|||||

QVQLVESGGGVVQPGRSLRLSCVASGFTFSSYGLHWVRQAPGKGLEWVAVISYDGSNKYYADSVKGRFTISRDNSKNTLYLQMNSLRAEDTAVYYCARDGDNDILTGYYRPPYYFDYWGQGTLVTVSS

^^^^^^^^ ^^^^^^^^ ^^^^^^^^^^^^^^^^^^^^^

Best Alignment of Farletuzumab light chain to a sequence from OAS

DIQLTQSPSSLSASVGDRVTITCSVSSSISSNNLHWYQQKPGKAPKPWIYGTSNLASGVPSRFSGSGSGTDYTFTISSLQPEDIATYYCQQWSSYPYMYTFGQGTKVEIK

|||.|||||||||||||||||||..|..|.||.|.|||||||||||..||..|||..||||||||||||||.|||||||||||||||||||..|||.|||||||||.|||

DIQMTQSPSSLSASVGDRVTITCQASQDI-SNYLNWYQQKPGKAPKLLIYDASNLETGVPSRFSGSGSGTDFTFTISSLQPEDIATYYCQQFNSYPPMYTFGQGTKLEIK

^^^^^^^ ^^^ ^^^^^^^^^^^

Best Alignment of Farletuzumab heavy chain CDRs to a sequence from OAS

EVQLVESGGGVVQPGRSLRLSCSASGFTFSGYGLSWVRQAPGKGLEWVAMISSGGSYTYYADSVKGRFAISRDNAKNTLFLQMDSLRPEDTGVYFCARHGDDPAWFAYWGQGTPVTVSS

||..|||||..|.||.||.|||.|||||||.||.|||||.|.|.|||||.||||||||||.|||||||.||||||||||.|||.||..|||..|.||||||||||||||||||.||||.

EVKVVESGGDLVKPGGSLKLSCAASGFTFSSYGMSWVRQTPDKRLEWVATISSGGSYTYYPDSVKGRFTISRDNAKNTLYLQMSSLKSEDTAMYYCARHGDDPAWFAYWGQGTLVTVSA

^^^^^^^^ ^^^^^^^^ ^^^^^^^^^^^^

Best Alignment of Farletuzumab light chain CDRs to a sequence from OAS

DIQLTQSPSSLSASVGDRVTITCSVSSSISSNNLHWYQQKPGKAPKPWIYGTSNLASGVPSRFSGSGSGTDYTFTISSLQPEDIATYYCQQWSSYPYMYTFGQGTKVEIK

.|.|||||....||.|..||.||||||||||.||||||||....||||||||||||||||.|||||||||.|..||||...||.||||||||||||.|||||.|||.|||

EIVLTQSPALMAASPGEKVTFTCSVSSSISSSNLHWYQQKSETSPKPWIYGTSNLASGVPVRFSGSGSGTSYSLTISSMEAEDAATYYCQQWSSYPLMYTFGGGTKLEIK

^^^^^^^ ^^^ ^^^^^^^^^^^

Best Alignment of Farletuzumab CDR-H3 to a sequence from OAS

EVQLVESGGGVVQPGRSLRLSCSASGFTFSGYGLSWVRQAPGKGLEWVAMISSGGSYTYYADSVKGRFAISRDNAKNTLFLQMDSLRPEDTGVYFCARHGDDPAWFAYWGQGTPVTVSS

|||.||||||.|.||.||.|||.|||||||.|..|||||.|.|.|||||.||.||..|||.|||||||.||||||||||.|||.|||.|||..|.||||||||||||||||||.||||.

EVQRVESGGGLVKPGGSLKLSCAASGFTFSSYTMSWVRQTPEKRLEWVATISGGGGNTYYPDSVKGRFTISRDNAKNTLYLQMSSLRSEDTALYYCARHGDDPAWFAYWGQGTLVTVSA

^^^^^^^^ ^^^^^^^^ ^^^^^^^^^^^^

Therapeutic : Fasinumab

Best Alignment of Fasinumab heavy chain to a sequence from OAS

QVQLVQSGAEVKKPGASVKVSCKVSGFTLTELSIHWVRQAPGKGLEWMGGFDPEDGETIYAQKFQGRVTMTEDTSTDTAYMELTSLRSEDTAVYYCSTIFGVVTNFDNWGQGTLVTVSS

||||||||||||||||||||||||||.||||||.|||||||||||||||||||||||||||||||||||||||||||||||||.||||||||||||.|.|||||.||.|||||||||||

QVQLVQSGAEVKKPGASVKVSCKVSGYTLTELSMHWVRQAPGKGLEWMGGFDPEDGETIYAQKFQGRVTMTEDTSTDTAYMELSSLRSEDTAVYYCATAFGVVTLFDYWGQGTLVTVSS

^^^^^^^^ ^^^^^^^^ ^^^^^^^^^^^^

Best Alignment of Fasinumab light chain to a sequence from OAS

DIQMTQSPSSLSASAGDRVTITCRASQAIRNDLGWYQQKPGKAPKRLIYAAFNLQSGVPSRFSGSGSGTEFTLTISSLQPEDLASYYCQQYNRYPWTFGQGTKVEIK

||||||||||||||.||||||||||||.|||||||||||.|||||||||||.||||||||||||||||||||||||||||||.|.|||||||.||||||||||||||

DIQMTQSPSSLSASVGDRVTITCRASQGIRNDLGWYQQKSGKAPKRLIYAASNLQSGVPSRFSGSGSGTEFTLTISSLQPEDFATYYCQQYNSYPWTFGQGTKVEIK

^^^^^^ ^^^ ^^^^^^^^^

Best Alignment of Fasinumab heavy chain CDRs to a sequence from OAS

QVQLVQSGAEVKKPGASVKVSCKVSGFTLTELSIHWVRQAPGKGLEWMGGFDPEDGETIYAQKFQGRVTMTEDTSTDTAYMELTSLRSEDTAVYYCSTIFGVVTNFDNWGQGTLVTVSS

................|||.||||||.||||||.|||||||||||||||||||||||||||||||||||||||||||||||||.||||||||||||.||||||||||.|||||||||||

----------------SVKASCKVSGYTLTELSMHWVRQAPGKGLEWMGGFDPEDGETIYAQKFQGRVTMTEDTSTDTAYMELSSLRSEDTAVYYCVTIFGVVTNFDYWGQGTLVTVSS

^^^^^^^^ ^^^^^^^^ ^^^^^^^^^^^^

Best Alignment of Fasinumab light chain CDRs to a sequence from OAS

DIQMTQSPSSLSASAGDRVTITCRASQAIRNDLGWYQQKPGKAPKRLIYAAFNLQSGVPSRFSGSGSGTEFTLTISSLQPEDLASYYCQQYNRYPWTFGQGTKVEIK

||||||||||||||.||||||||||||||||||.|.||||||||.|||.||..|||||||||||.|||||||||||||||.|.|.|||||||.||||||||||||||

DIQMTQSPSSLSASIGDRVTITCRASQAIRNDLDWFQQKPGKAPQRLISAASSLQSGVPSRFSGRGSGTEFTLTISSLQPDDFATYYCQQYNSYPWTFGQGTKVEIK

^^^^^^ ^^^ ^^^^^^^^^

Best Alignment of Fasinumab CDR-H3 to a sequence from OAS

QVQLVQSGAEVKKPGASVKVSCKVSGFTLTELSIHWVRQAPGKGLEWMGGFDPEDGETIYAQKFQGRVTMTEDTSTDTAYMELTSLRSEDTAVYYCSTIFGVVTNFDNWGQGTLVTVSS

................|...||..||||.......|.||||||||||............||....||.|...|......|....||..|||||||||||.||||.||||||||||||||

----------------SLRLSCAASGFTFSDHYMSWIRQAPGKGLEWISYISGSGRTIYYADSVKGRFTISRDNAKNSLYLQMNSLGAEDTAVYYCSTIVGVVTIFDNWGQGTLVTVSS

^^^^^^^^ ^^^^^^^^ ^^^^^^^^^^^^

Therapeutic : Fezakinumab

Best Alignment of Fezakinumab heavy chain to a sequence from OAS

QVQLVQSGAEVKKPGASVKVSCKASGYTFTNYYMHWVRQAPGQGLEWVGWINPYTGSAFYAQKFRGRVTMTRDTSISTAYMELSRLRSDDTAVYYCAREPEKF---DSDDSDVWGRGTLVTVSS

||||||||||||||||||||||||||||||.||||||||||||||||.|||||..|...|||||.||||||||||||||||||||||||||||||||||||.....|....||||.||.|||||

QVQLVQSGAEVKKPGASVKVSCKASGYTFTGYYMHWVRQAPGQGLEWMGWINPNSGGTNYAQKFQGRVTMTRDTSISTAYMELSRLRSDDTAVYYCAREPELEYPSDYYGMDVWGQGTTVTVSS

^^^^^^^^ ^^^^^^^^ ^^^^^^^^^^^^^^^^^

Best Alignment of Fezakinumab light chain to a sequence from OAS

QAVLTQPPSVSGAPGQRVTISCTGSSSNIGAGYGVHWYQQLPGTAPKLLIYGDSNRPSGVPDRFSGSKSGTSASLAITGLQAEDEADYYCQSYDNSLSGYVFGGGTQLTVL

|.|||||||||||||||||||||||||||||||.||||||||||||||||||.||||||||||||||||||||||||||||||||||||||||||||||.|||||||||||

QSVLTQPPSVSGAPGQRVTISCTGSSSNIGAGYDVHWYQQLPGTAPKLLIYGNSNRPSGVPDRFSGSKSGTSASLAITGLQAEDEADYYCQSYDNSLSGWVFGGGTQLTVL

^^^^^^^^^ ^^^ ^^^^^^^^^^^

Best Alignment of Fezakinumab heavy chain CDRs to a sequence from OAS

QVQLVQSGAEVKKPGASVKVSCKASGYTFTNYYMHWVRQAPGQGLEWVGWINPYTGSAFYAQKFRGRVTMTRDTSISTAYMELSRLRSDDTAVYYCAREPEKFDSDDSDVWGRGTLVTVSS

................||||||||||||||.||||||||||||||||.|.|||..||..|||||.||||||||||.||.|||||.|||..|||||||||...|.|||.||||.||.|||||

----------------SVKVSCKASGYTFTGYYMHWVRQAPGQGLEWMGIINPSGGSTSYAQKFQGRVTMTRDTSTSTVYMELSSLRSEETAVYYCARERDDFWSDDMDVWGKGTTVTVSS

^^^^^^^^ ^^^^^^^^ ^^^^^^^^^^^^^^

Best Alignment of Fezakinumab light chain CDRs to a sequence from OAS

QAVLTQPPSVSGAPGQRVTISCTGSSSNIGAGYGVHWYQQLPGTAPKLLIYGDSNRPSGVPDRFSGSKSGTSASLAITGLQAEDEADYYCQSYDNSLSGYVFGGGTQLTVL

|.||||.||||||||||||||||||||||||||..||||||||||||||||||||||||||||||||||||||||||||||||||||||||||||||||||||.||..|||

QSVLTQSPSVSGAPGQRVTISCTGSSSNIGAGYDIHWYQQLPGTAPKLLIYGDSNRPSGVPDRFSGSKSGTSASLAITGLQAEDEADYYCQSYDNSLSGYVFGTGTKVTVL

^^^^^^^^^ ^^^ ^^^^^^^^^^^

Best Alignment of Fezakinumab CDR-H3 to a sequence from OAS

QVQLVQSGAEVKKPGASVKVSCKASGYTF--TNYYMHWVRQAPGQGLEWVGWINPYTGSAFYAQKFRGRVTMTRDTSISTAYMELSRLRSDDTAVYYCAREPEKFDSDDSDVWGRGTLVTVSS

.....................|..||..|..|.||..||||.||.||||...|....||..|....|.||.|..||........||......||||||||.||.|.|||.||||.|.||||||

--------------SETLSLTCTVSGGSFASTTYYWGWVRQSPGKGLEWIAHIHYR-GSTYYKSSLRSRVIMSVDTFKNQFSLKLSSVTAAATAVYYCARDPEPFFSDDGDVWGQGILVTVSS

^^^^^^^^^^ ^^^^^^^^ ^^^^^^^^^^^^^^

Therapeutic : Ficlatuzumab

Best Alignment of Ficlatuzumab heavy chain to a sequence from OAS

QVQLVQPGAEVKKPGTSVKLSCKASGYTFTTYWMHWVRQAPGQGLEWIGEINPTNGHTNYNQKFQGRATLTVDKSTSTAYMELSSLRSEDTAVYYCARNYVG---SIFDYWGQGTLLTVSS

||||||.||||||||.|||.||||||||||.|.||||||||||||||.|.|||..|.|||.||||||.|.|.|.||||||||||||||||||||||||..|.....||||||||||.||||

QVQLVQSGAEVKKPGASVKVSCKASGYTFTGYYMHWVRQAPGQGLEWMGWINPNSGGTNYAQKFQGRVTITADESTSTAYMELSSLRSEDTAVYYCARSGVPYGDYIFDYWGQGTLVTVSS

^^^^^^^^ ^^^^^^^^ ^^^^^^^^^^^^^^

Best Alignment of Ficlatuzumab light chain to a sequence from OAS

DIVMTQSPDSLAMSLGERVTLNCKASENVVSYVSWYQQKPGQSPKLLIYGASNRESGVPDRFSGSGSATDFTLTISSVQAEDVADYHCGQSYNYPYTFGQGTKLEIK

.|||||||.|..||.||||||.|||||||..|||||||||||||||||||||||..||||||.|||||||||||||||||||.|||||||||.||||||.|||||||

NIVMTQSPKSMSMSVGERVTLSCKASENVGTYVSWYQQKPGQSPKLLIYGASNRYTGVPDRFTGSGSATDFTLTISSVQAEDLADYHCGQSYSYPYTFGGGTKLEIK

^^^^^^ ^^^ ^^^^^^^^^

Best Alignment of Ficlatuzumab heavy chain CDRs to a sequence from OAS

QVQLVQPGAEVKKPGTSVKLSCKASGYTFTTYWMHWVRQAPGQGLEWIGEINPTNGHTNYNQKFQGRATLTVDKSTSTAYMELSSLRSEDTAVYYCARNYVGSIFDYWGQGTLLTVSS

||||.|||.|..|||.||||||||||||||.||||||.|.|||||||||.|||.||.||||.||...||||||||.|||||.||||.|||.|||||||.|.|||||||||||.|||||

QVQLQQPGTELVKPGASVKLSCKASGYTFTSYWMHWVKQRPGQGLEWIGNINPSNGGTNYNEKFKSKATLTVDKSSSTAYMQLSSLTSEDSAVYYCARAYYGSIFDYWGQGTTLTVSS

^^^^^^^^ ^^^^^^^^ ^^^^^^^^^^^

Best Alignment of Ficlatuzumab light chain CDRs to a sequence from OAS

DIVMTQSPDSLAMSLGERVTLNCKASENVVSYVSWYQQKPGQSPKLLIYGASNRESGVPDRFSGSGSATDFTLTISSVQAEDVADYHCGQSYNYPYTFGQGTKLEIK

.|||||||.|..||.||||||.|||||||.|||||||||..|||||.|||||||..||||||.|||||||||||||||||||.|||||||||.||||||.|||||||

NIVMTQSPKSMSMSVGERVTLSCKASENVGSYVSWYQQKTEQSPKLVIYGASNRYTGVPDRFTGSGSATDFTLTISSVQAEDLADYHCGQSYSYPYTFGGGTKLEIK

^^^^^^ ^^^ ^^^^^^^^^

Best Alignment of Ficlatuzumab CDR-H3 to a sequence from OAS

QVQLVQPGAEVKKPGTSVKLSCKASGYTFTTYWMHWVRQAPGQGLEWIGEINPTNGHTNYNQKFQGRATLTVDKSTSTAYMELSSLRSEDTAVYYCARNYVGSIFDYWGQGTLLTVSS

||...|.|.....|..|....|..||...|.|..|||||.||.||||.|.|....|.|.||..|..|.....|.|.|.......||...|||.|||||||.|||||||||||.|||||

QVHVKQSGPGLVQPSQSLSITCTVSGFSLTSYGVHWVRQSPGKGLEWLGVIWSG-GSTDYNAAFISRLSISKDNSKSQVFFKMNSLQADDTAIYYCARNYYGSIFDYWGQGTTLTVSS

^^^^^^^^ ^^^^^^^^ ^^^^^^^^^^^

Therapeutic : Figitumumab

Best Alignment of Figitumumab heavy chain to a sequence from OAS

EVQLLESGGGLVQPGGSLRLSCTASGFTFSSYAMNWVRQAPGKGLEWVSAISGSGGTTFYADSVKGRFTISRDNSRTTLYLQMNSLRAEDTAVYYCAKDLGWSDS---------YYYYYGMDVWGQGTTVTVSS

||||||||||||||||||||||.|||||||||||.|||||||||||||||||||||.|.||||||||||||||||..|||||||||||||||||||||||||..|.........||||||||||||||||||||

EVQLLESGGGLVQPGGSLRLSCAASGFTFSSYAMSWVRQAPGKGLEWVSAISGSGGSTYYADSVKGRFTISRDNSKNTLYLQMNSLRAEDTAVYYCAKDLGWYSSSWFSGAIGPYYYYYGMDVWGQGTTVTVSS

^^^^^^^^ ^^^^^^^^ ^^^^^^^^^^^^^^^^^^^^^^^^^^^

Best Alignment of Figitumumab light chain to a sequence from OAS

DIQMTQFPSSLSASVGDRVTITCRASQGIRNDLGWYQQKPGKAPKRLIYAASRLHRGVPSRFSGSGSGTEFTLTISSLQPEDFATYYCLQHNSYPCSFGQGTKLEIK

||||||.|||||||||||||||||||||||||||||||||||||||||||||.|..|||||||||||||||||||||||||||||||||||||||||||||||||||

DIQMTQSPSSLSASVGDRVTITCRASQGIRNDLGWYQQKPGKAPKRLIYAASSLQSGVPSRFSGSGSGTEFTLTISSLQPEDFATYYCLQHNSYPCSFGQGTKLEIK

^^^^^^ ^^^ ^^^^^^^^^

Best Alignment of Figitumumab heavy chain CDRs to a sequence from OAS

EVQLLESGGGLVQPGGSLRLSCTASGFTFSSYAMNWVRQAPGKGLEWVSAISGSGGTTFYADSVKGRFTISRDNSRTTLYLQMNSLRAEDTAVYYCAKDLGWSDSYYYYYGMDVWGQGTTVTVSS

..............||||||||.|||||||||||.|||||||||||||||||||||.|.||||||||||||||||..||||||||||||||||||||||||.|.|||||||||||||||||||||

--------------GGSLRLSCAASGFTFSSYAMSWVRQAPGKGLEWVSAISGSGGSTYYADSVKGRFTISRDNSKNTLYLQMNSLRAEDTAVYYCAKDLGSSSSYYYYYGMDVWGQGTTVTVSS

^^^^^^^^ ^^^^^^^^ ^^^^^^^^^^^^^^^^^^

Best Alignment of Figitumumab light chain CDRs to a sequence from OAS

DIQMTQFPSSLSASVGDRVTITCRASQGIRNDLGWYQQKPGKAPKRLIYAASRLHRGVPSRFSGSGSGTEFTLTISSLQPEDFATYYCLQHNSYPCSFGQGTKLEIK

||.|||.||||||||||||||||||||||||||||||||||.||||||||||.||.||||||||.||||||||||||||.|||||||||||||||||||||||.|||

DIRMTQSPSSLSASVGDRVTITCRASQGIRNDLGWYQQKPGQAPKRLIYAASSLHSGVPSRFSGGGSGTEFTLTISSLQTEDFATYYCLQHNSYPCSFGQGTKVEIK

^^^^^^ ^^^ ^^^^^^^^^

Best Alignment of Figitumumab CDR-H3 to a sequence from OAS

EVQLLESGGGLVQPGGSLRLSCTASGFTFSSYAMNWVRQAPGKGLEWVSAISGSGGTTFYADSVKGRFTISRDNSRTTLYLQMNSLRAEDTAVYYCAKDLGWSDSYYYYYGMDVWGQGTTVTVSS

..............||||||||.|||||||||||.|||||||||||||||||||||.|.||||||||||||||||..||||||||||||||||||||||||.|.|||||||||||||||||||||

--------------GGSLRLSCAASGFTFSSYAMSWVRQAPGKGLEWVSAISGSGGSTYYADSVKGRFTISRDNSKNTLYLQMNSLRAEDTAVYYCAKDLGSSSSYYYYYGMDVWGQGTTVTVSS

^^^^^^^^ ^^^^^^^^ ^^^^^^^^^^^^^^^^^^

Therapeutic : Fletikumab

Best Alignment of Fletikumab heavy chain to a sequence from OAS

QVQLVQSGAEVKRPGASVKVSCKASGYTFTNDIIHWVRQAPGQRLEWMGWINAGYGNTQYSQNFQDRVSITRDTSASTAYMELISLRSEDTAVYYCAREPLWFGESSPHDYYGMDVWGQGTTVTVSS

||||||||||||.|||||||||||||||||....||||||||||||||||||||.|||.|||.||.||.||||||||||||||.||||||||||||||..||||||....|||||||||||||||||

QVQLVQSGAEVKKPGASVKVSCKASGYTFTSYAMHWVRQAPGQRLEWMGWINAGNGNTKYSQKFQGRVTITRDTSASTAYMELSSLRSEDTAVYYCARDLLWFGES-NYYYYGMDVWGQGTTVTVSS

^^^^^^^^ ^^^^^^^^ ^^^^^^^^^^^^^^^^^^^^

Best Alignment of Fletikumab light chain to a sequence from OAS

AIQLTQSPSSLSASVGDRVTITCRASQGISSALAWYQQKPGKAPKLLIYDASSLESGVPSRFSGSGSGTDFTLTISSLQPEDFATYYCQQFNSYPLTFGGGTKVEIK

|||||||||||||||||||||||||||||||||||||||||||||||||||||||||||||||||||||||||||||||||||||||||||||||||||||||||||

AIQLTQSPSSLSASVGDRVTITCRASQGISSALAWYQQKPGKAPKLLIYDASSLESGVPSRFSGSGSGTDFTLTISSLQPEDFATYYCQQFNSYPLTFGGGTKVEIK

^^^^^^ ^^^ ^^^^^^^^^

Best Alignment of Fletikumab heavy chain CDRs to a sequence from OAS

QVQLVQSGAEVKRPGASVKVSCKASGYTFTNDIIHWVRQAPGQRLEWMGWINAGYGNTQYSQNFQDRVSITRDTSASTAYMELISLRSEDTAVYYCAREPLWFGESSPHDYYGMDVWGQGTTVTVSS

................||||||||||||||....||||||||||||||||||||.|||.|||.||.||.||||||||||||||.|||||||||||||||.|||||.|||.||||||||||||||||.

----------------SVKVSCKASGYTFTSYAMHWVRQAPGQRLEWMGWINAGNGNTKYSQKFQGRVTITRDTSASTAYMELSSLRSEDTAVYYCAREGLWFGELSPHYYYGMDVWGQGTTVTVS-

^^^^^^^^ ^^^^^^^^ ^^^^^^^^^^^^^^^^^^^^

Best Alignment of Fletikumab light chain CDRs to a sequence from OAS

AIQLTQSPSSLSASVGDRVTITCRASQGISSALAWYQQKPGKAPKLLIYDASSLESGVPSRFSGSGSGTDFTLTISSLQPEDFATYYCQQFNSYPLTFGGGTKVEIK

|||||||||||||||||||||||||||||||||||||||||||||||||||||||||||||||||||||||||||||||||||||||||||||||||||.||||.||

AIQLTQSPSSLSASVGDRVTITCRASQGISSALAWYQQKPGKAPKLLIYDASSLESGVPSRFSGSGSGTDFTLTISSLQPEDFATYYCQQFNSYPLTFGPGTKVDIK

^^^^^^ ^^^ ^^^^^^^^^

Best Alignment of Fletikumab CDR-H3 to a sequence from OAS

QVQLVQSGAEVKRPGASVKVSCKASGYTFTNDIIHWVRQAPGQRLEWMGWINAGYGNTQYSQNFQDRVSITRDTSASTAYMELISLRSEDTAVYYCAREPLWFGESSPHDYYGMDVWGQGTTVTVSS

.....|||||.|.||.||||||||||.||||....||||||||.||.||.|...|....|...|..||.||.|...||.||||.||.||||||.|||||..|||||||||.||||||||||||.|||

-----QSGAEAKKPGSSVKVSCKASGGTFTNYAFSWVRQAPGQGLEGMGGIIPTYNSAHYARRFVGRVTITADEATSTVYMELSSLTSEDTAVCYCAREQRWFGESSPHDDYGMDVWGQGTTVIVSS

^^^^^^^^ ^^^^^^^^ ^^^^^^^^^^^^^^^^^^^^

Therapeutic : Foralumab

Best Alignment of Foralumab heavy chain to a sequence from OAS

QVQLVESGGGVVQPGRSLRLSCAASGFKFSGYGMHWVRQAPGKGLEWVAVIWYDGSKKYYVDSVKGRFTISRDNSKNTLYLQMNSLRAEDTAVYYCARQMGY-----------WHFDLWGRGTLVTVSS

|||||||||||||||||||||||||||.||.|||||||||||||||||||||||||.|||.|||||||||||||||||||||||||||||||||||||..||...........|.||||||||||||||

QVQLVESGGGVVQPGRSLRLSCAASGFTFSSYGMHWVRQAPGKGLEWVAVIWYDGSNKYYADSVKGRFTISRDNSKNTLYLQMNSLRAEDTAVYYCARESGYDFWSGYYGSSYWYFDLWGRGTLVTVSS

^^^^^^^^ ^^^^^^^^ ^^^^^^^^^^^^^^^^^^^^^^

Best Alignment of Foralumab light chain to a sequence from OAS

EIVLTQSPATLSLSPGERATLSCRASQSVSSYLAWYQQKPGQAPRLLIYDASNRATGIPARFSGSGSGTDFTLTISSLEPEDFAVYYCQQRSNWPPLTFGGGTKVEIK

||||||||||||||||||||||||||||||||||||||||||||||||||||||||||||||||||||||||||||||||||||||||||||||||||||||||||||

EIVLTQSPATLSLSPGERATLSCRASQSVSSYLAWYQQKPGQAPRLLIYDASNRATGIPARFSGSGSGTDFTLTISSLEPEDFAVYYCQQRSNWPPLTFGGGTKVEIK

^^^^^^ ^^^ ^^^^^^^^^^

Best Alignment of Foralumab heavy chain CDRs to a sequence from OAS

QVQLVESGGGVVQPGRSLRLSCAASGFKFSGYGMHWVRQAPGKGLEWVAVIWYDGSKKYYVDSVKGRFTISRDNSKNTLYLQMNSLRAEDTAVYYCARQMGYWHFDLWGRGTLVTVSS

.............||||||||||||||.||.|||||||||||||||||||||||||||||.||||||||||||||||||||||||||.||||||||||..|||.|||||||.||||||

-------------PGRSLRLSCAASGFTFSIYGMHWVRQAPGKGLEWVAVIWYDGSKKYYADSVKGRFTISRDNSKNTLYLQMNSLRVEDTAVYYCARGNGYWYFDLWGRGALVTVSS

^^^^^^^^ ^^^^^^^^ ^^^^^^^^^^^

Best Alignment of Foralumab light chain CDRs to a sequence from OAS

EIVLTQSPATLSLSPGERATLSCRASQSVSSYLAWYQQKPGQAPRLLIYDASNRATGIPARFSGSGSGTDFTLTISSLEPEDFAVYYCQQRSNWPPLTFGGGTKVEIK

|||||||||||||||||||||||||||||||||||||||||||||||||||||||||||||||||||||||||||||.||||||.|||||||||||||||||||||||

EIVLTQSPATLSLSPGERATLSCRASQSVSSYLAWYQQKPGQAPRLLIYDASNRATGIPARFSGSGSGTDFTLTISSIEPEDFAIYYCQQRSNWPPLTFGGGTKVEIK

^^^^^^ ^^^ ^^^^^^^^^^

Best Alignment of Foralumab CDR-H3 to a sequence from OAS

QVQLVESGGGVVQPGRSLRLSCAASGFKFSGYGMHWVRQAPGKGLEWVAVIWYDGSKKYYVDSVKGRFTISRDNSKNTLYLQMNSLRAEDTAVYYCARQMGYWHFDLWGRGTLVTVSS

.................|.|.|..||...||....|.||.|||||||...|.|......|..|.|.|.|.|.|.|||...|...|..|.||||||||||||||.||||||||||||||

--------------SETLSLTCTVSGGSISGHSWSWIRQPPGKGLEWIGYIYYS-GSANYGPSLKSRVTLSVDTSKNQFSLNLASVTAADTAVYYCARQMGYWYFDLWGRGTLVTVSS

^^^^^^^^ ^^^^^^^^ ^^^^^^^^^^^

Therapeutic : Foravirumab

Best Alignment of Foravirumab heavy chain to a sequence from OAS

QVQLVESGGGAVQPGRSLRLSCAASGFTFSSYGMHWVRQAPGKGLEWVAVILYDGSDKFYADSVKGRFTISRDNSKNTLYLQMNSLRAEDTAVYYCAKVAVAGTHFDYWGQGTLVTVSS

||||||||||.||||||||||||||||||||||||||||||||||||||||.||||.|.|||||||||||||||||||||||||||||||||||||||||..|.|||||||||||||||

QVQLVESGGGVVQPGRSLRLSCAASGFTFSSYGMHWVRQAPGKGLEWVAVIWYDGSNKYYADSVKGRFTISRDNSKNTLYLQMNSLRAEDTAVYYCAKVAPSGYHFDYWGQGTLVTVSS

^^^^^^^^ ^^^^^^^^ ^^^^^^^^^^^^

Best Alignment of Foravirumab light chain to a sequence from OAS

DIQMTQSPSSLSASVGDRVTITCRASQGIRNDLGWYQQKPGKAPKLLIYAASSLQSGVPSRFSGSGSGTDFTLTISSLQPEDFATYYCQQLNSYPPTFGGGTKVEIK

.||||||||||||||||||||||||||||||||||||||||||||||||||||||||||||||||||||||||||||||||||||||||||||||.|||||||||||

AIQMTQSPSSLSASVGDRVTITCRASQGIRNDLGWYQQKPGKAPKLLIYAASSLQSGVPSRFSGSGSGTDFTLTISSLQPEDFATYYCQQLNSYPLTFGGGTKVEIK

^^^^^^ ^^^ ^^^^^^^^^

Best Alignment of Foravirumab heavy chain CDRs to a sequence from OAS

QVQLVESGGGAVQPGRSLRLSCAASGFTFSSYGMHWVRQAPGKGLEWVAVILYDGSDKFYADSVKGRFTISRDNSKNTLYLQMNSLRAEDTAVYYCAKVAVAGTHFDYWGQGTLVTVSS

................|||||||||||||||||.|||||||||||||||||.||||.|.|||||||||||||||||||||||||||||||||||||||||||||.||||||||||||||

----------------SLRLSCAASGFTFSSYGTHWVRQAPGKGLEWVAVISYDGSNKYYADSVKGRFTISRDNSKNTLYLQMNSLRAEDTAVYYCAKVAVAGTDFDYWGQGTLVTVSS

^^^^^^^^ ^^^^^^^^ ^^^^^^^^^^^^

Best Alignment of Foravirumab light chain CDRs to a sequence from OAS

DIQMTQSPSSLSASVGDRVTITCRASQGIRNDLGWYQQKPGKAPKLLIYAASSLQSGVPSRFSGSGSGTDFTLTISSLQPEDFATYYCQQLNSYPPTFGGGTKVEIK

.|||||||||||.||||||||||||||||||||||.||||.|||..||||||||.||||||||.|||||.|||||.|||||||||||||||||||||||||||||||

-IQMTQSPSSLSPSVGDRVTITCRASQGIRNDLGWSQQKPVKAPNVLIYAASSLESGVPSRFSASGSGTEFTLTIGSLQPEDFATYYCQQLNSYPPTFGGGTKVEIK

^^^^^^ ^^^ ^^^^^^^^^

Best Alignment of Foravirumab CDR-H3 to a sequence from OAS

QVQLVESGGGAVQPGRSLRLSCAASGFTFSSYGMHWVRQAPGKGLEWVAVILYDGSDKFYADSVKGRFTISRDNSKNTLYLQMNSLRAEDTAVYYCAKVAVAGTHFDYWGQGTLVTVSS

..............|.||..||..||..|.||...||||.|||||||...|....||..|..|..|..|||.|.|..|.|||..||.|.|||.||||||||||||||||||||||||||

--------------GESLKISCKGSGYSFTSYWIGWVRQMPGKGLEWMGIIYPGDSDTRYSPSFQGQVTISADKSISTAYLQWSSLKASDTAMYYCAKVAVAGTHFDYWGQGTLVTVSS

^^^^^^^^ ^^^^^^^^ ^^^^^^^^^^^^

Therapeutic : Fremanezumab

Best Alignment of Fremanezumab heavy chain to a sequence from OAS

EVQLVESGGGLVQPGGSLRLSCAASGFTFSNYWISWVRQAPGKGLEWVAEIRSESDASATHYAEAVKGRFTISRDNAKNSLYLQMNSLRAEDTAVYYCLAYFDYG---LAIQNYWGQGTLVTVSS

||||||||||||||||||||||||||||||.||.|||||||||||||||.|......|...|...|||||||||||||||||||||||||||||||||...||||........||||||||||||

EVQLVESGGGLVQPGGSLRLSCAASGFTFSSYWMSWVRQAPGKGLEWVANIKQD--GSEKYYVDSVKGRFTISRDNAKNSLYLQMNSLRAEDTAVYYCARDFDYGGNSPSYFDYWGQGTLVTVSS

^^^^^^^^ ^^^^^^^^^^ ^^^^^^^^^^^^^^^^

Best Alignment of Fremanezumab light chain to a sequence from OAS

EIVLTQSPATLSLSPGERATLSCKASKRVTTYVSWYQQKPGQAPRLLIYGASNRYLGIPARFSGSGSGTDFTLTISSLEPEDFAVYYCSQSYN-YPYTFGQGTKLEIK

|||||||||||||||||||||||.||..||||..|||||||||||||||.||||..||||||||||||||||||||||||||||||||.|..|..|||||||||||||

EIVLTQSPATLSLSPGERATLSCRASQSVTTYLAWYQQKPGQAPRLLIYDASNRATGIPARFSGSGSGTDFTLTISSLEPEDFAVYYCQQRSNWPPYTFGQGTKLEIK

^^^^^^ ^^^ ^^^^^^^^^^

Best Alignment of Fremanezumab heavy chain CDRs to a sequence from OAS

EVQLVESGGGLVQPGGSLRLSCAASGFTFSNYWISWVRQAPGKGLEWVAEIRSESDASATHYAEAVKGRFTISRDNAKNSLYLQMNSLRAEDTAVYYCLAYFDYGLAIQNYWGQGTLVTVSS

..|||||||||||||||..|||.||||||||||..||||.|.|||||||.||..||..||||||.||||||||||..|.|.|||||.||||||..|||.||.||.||...|||||||||||.

--QLVESGGGLVQPGGSMKLSCVASGFTFSNYWMNWVRQSPAKGLEWVAQIRLKSDNYATHYAESVKGRFTISRDDSKSSVYLQMNNLRAEDTGIYYCTAYYDYDLAWFAYWGQGTLVTVSA

^^^^^^^^ ^^^^^^^^^^ ^^^^^^^^^^^^^

Best Alignment of Fremanezumab light chain CDRs to a sequence from OAS

EIVLTQSPATLSLSPGERATLSCKASKRVTTYVSWYQQKPGQAPRLLIYGASNRYLGIPARFSGSGSGTDFTLTISSLEPEDFAVYYCSQSYNYPYTFGQGTKLEIK

.||.||||...|.|.|||..|.|..||.|.|||.||||||.|.|.||||||||||.|.|.||.||||.|.|||||||...||.|.|.|.|||.||||||.|||||||

-IVMTQSPKSMSMSVGERDSLICQSSKNVRTYVHWYQQKPEQSPKLLIYGASNRYTGVPDRFTGSGSATYFTLTISSVQAEDLADYHCRQSYSYPYTFGAGTKLEIK

^^^^^^ ^^^ ^^^^^^^^^

Best Alignment of Fremanezumab CDR-H3 to a sequence from OAS

EVQLVESGGGLVQPGGSLRLSCAASGFTFSNYWISWVRQAPGKGLEWVAEIRSESDASATHYAEAVKGRFTISRDNAKNSLYLQMNSLRAEDTAVYYCLAYFDYGLAIQNYWGQGTLVTVSS

................||||||.||||||..|..||||||||.|.|||..|.......|..||..|||||||.||.||||.||..||||||||||.||||..|||...|.||||||||||||

----------------SLRLSCKASGFTFRTYAMSWVRQAPGQGPEWVSDISTS--GTAKYYAHTVKGRFTITRDDAKNSPYLRINSLRAEDTAVFYCLAGPDYGDYGQDYWGQGTLVTVSS

^^^^^^^^ ^^^^^^^^^^ ^^^^^^^^^^^^^

Therapeutic : Fresolimumab

Best Alignment of Fresolimumab heavy chain to a sequence from OAS

QVQLVQSGAEVKKPGSSVKVSCKASGYTFSSNVISWVRQAPGQGLEWMGGVIPIVDIANYAQRFKGRVTITADESTSTTYMELSSLRSEDTAVYYCASTLGLV-----LDAMDYWGQGTLVTVSS

||||||||||||||||||||||||||.||||..|||||||||||||||||.|||...|||||.|.|||||||||||||.||||||||||||||||||.|||.......||..|||||||||||||

QVQLVQSGAEVKKPGSSVKVSCKASGGTFSSYAISWVRQAPGQGLEWMGGIIPIFGTANYAQKFQGRVTITADESTSTAYMELSSLRSEDTAVYYCARTLGYSGYDPDLDNFDYWGQGTLVTVSS

^^^^^^^^ ^^^^^^^^ ^^^^^^^^^^^^^^^^^^

Best Alignment of Fresolimumab light chain to a sequence from OAS

ETVLTQSPGTLSLSPGERATLSCRASQSLGSSYLAWYQQKPGQAPRLLIYGASSRAPGIPDRFSGSGSGTDFTLTISRLEPEDFAVYYCQQYADSPITFGQGTRLEIK

||||||||||||||||||||||||||||..||||||||||||||||||||||||||.|||||||||||||||||||||||||||||||||||..||||||||||||||

ETVLTQSPGTLSLSPGERATLSCRASQSVSSSYLAWYQQKPGQAPRLLIYGASSRATGIPDRFSGSGSGTDFTLTISRLEPEDFAVYYCQQYGSSPITFGQGTRLEIK

^^^^^^^ ^^^ ^^^^^^^^^

Best Alignment of Fresolimumab heavy chain CDRs to a sequence from OAS

QVQLVQSGAEVKKPGSSVKVSCKASGYTFSSNVISWVRQAPGQGLEWMGGVIPIVDIANYAQRFKGRVTITADESTSTTYMELSSLRSEDTAVYYCASTLGLVLDAMDYWGQGTLVTVSS

................||||||||||.||||..|||||||||||||||||.|||..||||||.|.||||||||.||||.||||||||.|||||||||||.|..|||.|.|||||.|||||

----------------SVKVSCKASGGTFSSYAISWVRQAPGQGLEWMGGIIPILGIANYAQKFQGRVTITADKSTSTAYMELSSLRAEDTAVYYCASTYGDYLDAFDIWGQGTMVTVSS

^^^^^^^^ ^^^^^^^^ ^^^^^^^^^^^^^

Best Alignment of Fresolimumab light chain CDRs to a sequence from OAS

ETVLTQSPGTLSLSPGERATLSCRASQSLGSSYLAWYQQKPGQAPRLLIYGASSRAPGIPDRFSGSGSGTDFTLTISRLEPEDFAVYYCQQYADSPITFGQGTRLEIK

|.||||||||||||||||||||||||||||||||||||||||||||||||||||||.|||||||||||||||||||.||||||||||||||||.||.|||||||..||

EIVLTQSPGTLSLSPGERATLSCRASQSLGSSYLAWYQQKPGQAPRLLIYGASSRATGIPDRFSGSGSGTDFTLTINRLEPEDFAVYYCQQYASSPWTFGQGTRVDIK

^^^^^^^ ^^^ ^^^^^^^^^

Best Alignment of Fresolimumab CDR-H3 to a sequence from OAS

QVQLVQSGAEVKKPGSSVKVSCKASGYTFSSNVISWVRQAPGQGLEWMGGVIPIVDIANYAQRFKGRVTITADESTSTTYMELSSLRSEDTAVYYCASTLGLVLDAMDYWGQGTLVTVSS

.|.||.||.|..|||.|||.|||||||.|.|....||.|.||.||||.|...|.....||...|||..|.|||....|.||.||.|.||...||.|||||.|||.|||||||||.|||||

-VKLVESGPELVKPGASVKISCKASGYAFTSSWVNWVKQRPGKGLEWIGLIYPGGGATNYNGKFKGQATLTADKYSRTAYMQLSRLTSEASVVYFCASTLRLVLYAMDYWGQGTSVTVSS

^^^^^^^^ ^^^^^^^^ ^^^^^^^^^^^^^

Therapeutic : Fulranumab

Best Alignment of Fulranumab heavy chain to a sequence from OAS

EVQLVESGGGLVQPGGSLRLSCAASGFTLRSYSMNWVRQAPGKGLEWVSYISRSSHTIFYADSVKGRFTISRDNAKNSLYLQMDSLRDEDTAMYYCARVYSSGWHVSDYFDYWGQGILVTVSS

||||||||||||||||||||||||||||..||||||||||||||||||||||.||.||.||||||||||||||||||||||||.||||||||.|||||.|||||.|..||||||||.||||||

EVQLVESGGGLVQPGGSLRLSCAASGFTFSSYSMNWVRQAPGKGLEWVSYISSSSSTIYYADSVKGRFTISRDNAKNSLYLQMNSLRDEDTAVYYCARGYSSGWYVVYYFDYWGQGTLVTVSS

^^^^^^^^ ^^^^^^^^ ^^^^^^^^^^^^^^^^

Best Alignment of Fulranumab light chain to a sequence from OAS

AIQLTQSPSSLSASVGDRVTITCRASQGISSALAWYQQKPGKAPKLLIYDASSLESGVPSRFSGSGSGTDFTLTISSLQPEDFATYYCQQFNSYPLTFGGGTKVEIK

|||||||||||||||||||||||||||||||||||||||||||||||||||||||||||||||||||||||||||||||||||||||||||||||||||||||||||

AIQLTQSPSSLSASVGDRVTITCRASQGISSALAWYQQKPGKAPKLLIYDASSLESGVPSRFSGSGSGTDFTLTISSLQPEDFATYYCQQFNSYPLTFGGGTKVEIK

^^^^^^ ^^^ ^^^^^^^^^

Best Alignment of Fulranumab heavy chain CDRs to a sequence from OAS

EVQLVESGGGLVQPGGSLRLSCAASGFTLRSYSMNWVRQAPGKGLEWVSYISRSSHTIFYADSVKGRFTISRDNAKNSLYLQMDSLRDEDTAMYYCARVYSSGWHVSDYFDYWGQGILVTVSS

..............||||||||||||||..||||||||||||||||||||||.||.||.||||||||||||||||||||||||.|||.||||.|||||.|||||..||||||||||.||||||

--------------GGSLRLSCAASGFTFSSYSMNWVRQAPGKGLEWVSYISSSSSTIYYADSVKGRFTISRDNAKNSLYLQMNSLRAEDTAVYYCARAYSSGWYPSDYFDYWGQGTLVTVSS

^^^^^^^^ ^^^^^^^^ ^^^^^^^^^^^^^^^^

Best Alignment of Fulranumab light chain CDRs to a sequence from OAS

AIQLTQSPSSLSASVGDRVTITCRASQGISSALAWYQQKPGKAPKLLIYDASSLESGVPSRFSGSGSGTDFTLTISSLQPEDFATYYCQQFNSYPLTFGGGTKVEIK

|||||||||||||||||||||||||||||||||||||||||||||||||||||||||||||||||||||||||||||||||||||||||||||||||||.||||.||

AIQLTQSPSSLSASVGDRVTITCRASQGISSALAWYQQKPGKAPKLLIYDASSLESGVPSRFSGSGSGTDFTLTISSLQPEDFATYYCQQFNSYPLTFGPGTKVDIK

^^^^^^ ^^^ ^^^^^^^^^

Best Alignment of Fulranumab CDR-H3 to a sequence from OAS

EVQLVESGGGLVQPGGSLRLSCAASGFTL--RSYSMNWVRQAPGKGLEWVSYISRSSHTIFYADSVKGRFTISRDNAKNSLYLQMDSLRDEDTAMYYCARVYSSGWHVSDYFDYWGQGILVTVSS

.................|.|.|..||......||...|.||.|||||||...|..|.....|..|.|.|.|||.|..||...|...|....|||.|||||||||||.|||||||||||.||||||

--------------SETLSLTCTVSGGSISSSSYYWGWIRQPPGKGLEWIGSIYYS-GSTYYNPSLKSRVTISVDTSKNQFSLKLSSVTAADTAVYYCARVYSSGWYVSDYFDYWGQGTLVTVSS

^^^^^^^^^^ ^^^^^^^^ ^^^^^^^^^^^^^^^^

Therapeutic : Futuximab

Best Alignment of Futuximab heavy chain to a sequence from OAS

EVQLQQPGSELVRPGASVKLSCKASGYTFTSYWMHWVKQRPGQGLEWIGNIYPGSRSTNYDEKFKSKATLTVDTSSSTAYMQLSSLTSEDSAVYYCTRNGDYYVSSGDAMDYWGQGTSVTVSS

.||||||||||||||||||||||||||||||||||||||||||||||||||||||.||||||||||||||||||||||||||||||||||||||||||.|.||.||.||||||||||||||||

QVQLQQPGSELVRPGASVKLSCKASGYTFTSYWMHWVKQRPGQGLEWIGNIYPGSGSTNYDEKFKSKATLTVDTSSSTAYMQLSSLTSEDSAVYYCTRRGSYYGSSYDAMDYWGQGTSVTVSS

^^^^^^^^ ^^^^^^^^ ^^^^^^^^^^^^^^^^

Best Alignment of Futuximab light chain to a sequence from OAS

DIQMTQTTSSLSASLGDRVTISCRTSQDIGNYLNWYQQKPDGTVKLLIYYTSRLHSGVPSRFSGSGSGTDFSLTINNVEQEDVATYFCQHYNTVPPTFGGGTKLEIK

||||||||||||||||||||||||.||||.||||||||||||||||||||||||||||||||||||||||.||||.|.|||||||||||..||.|||||||||||||

DIQMTQTTSSLSASLGDRVTISCRASQDISNYLNWYQQKPDGTVKLLIYYTSRLHSGVPSRFSGSGSGTDYSLTISNLEQEDVATYFCQQGNTLPPTFGGGTKLEIK

^^^^^^ ^^^ ^^^^^^^^^

Best Alignment of Futuximab heavy chain CDRs to a sequence from OAS

EVQLQQPGSELVRPGASVKLSCKASGYTFTSYWMHWVKQRPGQGLEWIGNIYPGSRSTNYDEKFKSKATLTVDTSSSTAYMQLSSLTSEDSAVYYCTRNGDYYVSSGDAMDYWGQGTSVTVSS

.||||||||||||||||||||||||||||||||||||||||||||||||||||||.||||||||||||||||||||||||||||||||||||||||||.|.||.|||||||||||||||||||

-VQLQQPGSELVRPGASVKLSCKASGYTFTSYWMHWVKQRPGQGLEWIGNIYPGSGSTNYDEKFKSKATLTVDTSSSTAYMQLSSLTSEDSAVYYCTRWGYYYGSSGDAMDYWGQGTSVTVSS

^^^^^^^^ ^^^^^^^^ ^^^^^^^^^^^^^^^^

Best Alignment of Futuximab light chain CDRs to a sequence from OAS

DIQMTQTTSSLSASLGDRVTISCRTSQDIGNYLNWYQQKPDGTVKLLIYYTSRLHSGVPSRFSGSGSGTDFSLTINNVEQEDVATYFCQHYNTVPPTFGGGTKLEIK

||||||..||||||.|||.||.||.||||.|||.||||||....|.||||||.|..|||||||||.||||..|||.....||.|||.||||||.|||||||||.|.|

DIQMTQSPSSLSASVGDRITITCRASQDINNYLSWYQQKPGKAPKPLIYYTSSLETGVPSRFSGSRSGTDYTLTISSLQPEDIATYYCQHYNTLPPTFGGGTKGELK

^^^^^^ ^^^ ^^^^^^^^^

Best Alignment of Futuximab CDR-H3 to a sequence from OAS

EVQLQQPGSELVRPGASVKLSCKASGYTFTSYWMHWVKQRPGQGLEWIGNIYPGSRSTNYDEKFKSKATLTVDTSSSTAYMQLSSLTSEDSAVYYCTRNGDYYVSSGDAMDYWGQGTSVTVSS

.|||||.|..|.|||||||.|||.||||||||||||||||||.||||||.||||...|.|..|||.||.||..||.|||||.|||||.||||||||||.||||.|||.|||||||||||||||

-VQLQQSGTVLARPGASVKMSCKTSGYTFTSYWMHWVKQRPGPGLEWIGAIYPGNSDTSYNQKFKGKAKLTAVTSASTAYMELSSLTNEDSAVYYCTRSGDYYGSSGGAMDYWGQGTSVTVSS

^^^^^^^^ ^^^^^^^^ ^^^^^^^^^^^^^^^^

Therapeutic : Galcanezumab

Best Alignment of Galcanezumab heavy chain to a sequence from OAS

QVQLVQSGAEVKKPGSSVKVSCKASGYTFGNYWMQWVRQAPGQGLEWMGAIYEGTGKTVYIQKFADRVTITADKSTSTAYMELSSLRSEDTAVYYCARLSDY-VSGFGYWGQGTTVTVSS

|||||||||||||||.|||||||||||||..|.|.||||||||||||||.|....|.|.|.|||..||||||||||||||||||||||||||||||||||.|....|.||||||.|||||

QVQLVQSGAEVKKPGASVKVSCKASGYTFTSYYMHWVRQAPGQGLEWMGIINPSGGSTSYAQKFQGRVTITADKSTSTAYMELSSLRSEDTAVYYCARLSGYNKYYFDYWGQGTLVTVSS

^^^^^^^^ ^^^^^^^^ ^^^^^^^^^^^^^

Best Alignment of Galcanezumab light chain to a sequence from OAS

DIQMTQSPSSLSASVGDRVTITCRASKDISKYLNWYQQKPGKAPKLLIYYTSGYHSGVPSRFSGSGSGTDFTLTISSLQPEDFATYYCQQGDALPPTFGGGTKVEIK

||||||||||||||||||||||||||..||.||||||||||||||||||..|...|||||||||||||||||||||||||||||||||||.|.||||||||||||||

DIQMTQSPSSLSASVGDRVTITCRASQSISSYLNWYQQKPGKAPKLLIYAASSLQSGVPSRFSGSGSGTDFTLTISSLQPEDFATYYCQQYDNLPPTFGGGTKVEIK

^^^^^^ ^^^ ^^^^^^^^^

Best Alignment of Galcanezumab heavy chain CDRs to a sequence from OAS

QVQLVQSGAEVKKPGSSVKVSCKASGYTFGNYWMQWVRQAPGQGLEWMGAIYEGTGKTVYIQKFADRVTITADKSTSTAYMELSSLRSEDTAVYYCARLSDYVSGFGYWGQGTTVTVSS

||||.|||||...||.|||.|||||||||.|||..|..|.||.||||.|.||.|.|.|.|..||....|.|||||.|||||..|||.|||.|.|||||||||..||.|||||||.||||

QVQLQQSGAELVRPGTSVKMSCKASGYTFTNYWIGWAKQRPGHGLEWIGDIYPGGGYTNYNEKFKGKATLTADKSSSTAYMQFSSLTSEDSAIYYCARLSDYDGGFDYWGQGTTLTVSS

^^^^^^^^ ^^^^^^^^ ^^^^^^^^^^^^

Best Alignment of Galcanezumab light chain CDRs to a sequence from OAS

DIQMTQSPSSLSASVGDRVTITCRASKDISKYLNWYQQKPGKAPKLLIYYTSGYHSGVPSRFSGSGSGTDFTLTISSLQPEDFATYYCQQGDALPPTFGGGTKVEIK

||||||..||||||.||||||.||||.|||||||||||||....||||||||..||||||||||||||||..||||.|..||.|||.||||.|||.|||.|||.|||

DIQMTQTTSSLSASLGDRVTISCRASQDISKYLNWYQQKPDGTVKLLIYYTSRLHSGVPSRFSGSGSGTDYSLTISNLEQEDIATYFCQQGNALPLTFGAGTKLEIK

^^^^^^ ^^^ ^^^^^^^^^

Best Alignment of Galcanezumab CDR-H3 to a sequence from OAS

QVQLVQSGAEVKKPGSSVKVSCKASGYTFGNYWMQWVRQAPGQGLEWMGAIYEGTGKTVYIQKFADRVTITADKSTSTAYMELSSLRSEDTAVYYCARLSDYVSGFGYWGQGTTVTVSS

................|...||.|||.|...|.|.|||||||.||||...|..|.....|......|.||..|......|....|.|.||||||||||..|||||||||||||.|||||

----------------SLRLSCAASGFTLSSYSMNWVRQAPGKGLEWVASITTGGSYKYYGDSVKGRFTISRDNAKNSLYLQMNSMRAEDTAVYYCARSGDYVSGFGYWGQGTLVTVSS

^^^^^^^^ ^^^^^^^^ ^^^^^^^^^^^^

Therapeutic : Galiximab

Best Alignment of Galiximab heavy chain to a sequence from OAS

QVQLQESGPGLVKPSETLSLTCAVSGGSISGGYGWGWIRQPPGKGLEWIGSFYSSSGNTYYNPSLKSQVTISTDTSKNQFSLKLNSMTAADTAVYYCVRDRLFSVVGMVYNNWFDVWGPGVLVTVSS

||||||||||||||||||||||||||||.|||||||||||||||||||||..||.||||||||||||.||||||||||||||||.|.||||||||||.|...........|||||||||||||||||

QVQLQESGPGLVKPSETLSLTCAVSGGSVSGGYGWGWIRQPPGKGLEWIGTIYSGSGNTYYNPSLKSRVTISTDTSKNQFSLKLTSVTAADTAVYYCAREPFSRY---SWNNWFDVWGPGVLVTVSS

^^^^^^^^^ ^^^^^^^^ ^^^^^^^^^^^^^^^^^^^

Best Alignment of Galiximab light chain to a sequence from OAS

ESALTQPPSVSGAPGQKVTISCTGSTSNIGGYDLHWYQQLPGTAPKLLIYDINKRPSGISDRFSGSKSGTAASLAITGLQTEDEADYYCQSYDSSLNAQVFGGGTRLTVL

.|.||||||||||||||||||||||.|||||||.|||||||||||||||||.||||||||||||||||||.|||||||||||||||||||||||||||||||||||||||

QSVLTQPPSVSGAPGQKVTISCTGSSSNIGGYDVHWYQQLPGTAPKLLIYDNNKRPSGISDRFSGSKSGTSASLAITGLQTEDEADYYCQSYDSSLNAQVFGGGTRLTVL

^^^^^^^^ ^^^ ^^^^^^^^^^^

Best Alignment of Galiximab heavy chain CDRs to a sequence from OAS

QVQLQESGPGLVKPSETLSLTCAVSGGSISGGYGWGWIRQPPGKGLEWIGSFYSSSGNTYYNPSLKSQVTISTDTSKNQFSLKLNSMTAADTAVYYCVRDRLFSVVGMVYNNWFDVWGPGVLVTVSS

|||||||||||.|||||||||||||||||||||.|.||||||||||||||.....||||.|.|||||.||||.|||||||||.|.|.||||||||||.|||.....|..|.||.||||||||.||||

QVQLQESGPGLLKPSETLSLTCAVSGGSISGGYAWSWIRQPPGKGLEWIGFIGGYSGNTDYDPSLKSRVTISKDTSKNQFSLNLSSVTAADTAVYYCARDRGYCSGGVCYGNWVDVWGPGVLATVSS

^^^^^^^^^ ^^^^^^^^ ^^^^^^^^^^^^^^^^^^^

Best Alignment of Galiximab light chain CDRs to a sequence from OAS

ESALTQPPSVSGAPGQKVTISCTGSTSNIGGYDLHWYQQLPGTAPKLLIYDINKRPSGISDRFSGSKSGTAASLAITGLQTEDEADYYCQSYDSSLNAQVFGGGTRLTVL

.|.||||||||||||||||||||||.|||||||.||||||||||||||||||||||||.|||||||||||.|||||||||||||||||||||||||||.|||.||.||||

QSVLTQPPSVSGAPGQKVTISCTGSSSNIGGYDVHWYQQLPGTAPKLLIYDINKRPSGVSDRFSGSKSGTSASLAITGLQTEDEADYYCQSYDSSLNAHVFGSGTKLTVL

^^^^^^^^ ^^^ ^^^^^^^^^^^

Best Alignment of Galiximab CDR-H3 to a sequence from OAS

QVQLQESGPGLVKPSETLSLTCAVSGGSISGGYGWGWIRQPPGKGLEWIGSFYSSSGNTYYNPSLKSQVTISTDTSKNQFSLKLNSMTAADTAVYYCVRDRLFSVVGMVYNNWFDVWGPGVLVTVSS

.....................|..||......|...|.||.||.||||.|........|.|.......||..|||..|.....|.|....|||||||||..|..|||.|.|||||.||.|.||||||

----------------SVKVSCKASGYRF-STYSITWVRQAPGQGLEWMGWISPYNRDTKYARKFQGRVTMTTDTITNTAYMELRSLRSDDTAVYYCVRGALVGVVGGVQNNWFDPWGQGTLVTVSS

^^^^^^^^^ ^^^^^^^^ ^^^^^^^^^^^^^^^^^^^

Therapeutic : Ganitumab

Best Alignment of Ganitumab heavy chain to a sequence from OAS

QVQLQESGPGLVKPSGTLSLTCAVSGGSISSSNWWSWVRQPPGKGLEWIGEIYHSGSTNYNPSLKSRVTISVDKSKNQFSLKLSSVTAADTAVYYCARWTGR-TDAFDIWGQGTMVTVSS

||||||||||||||||||||||||||||||||||||||||||||||||||||||||||||||||||||||||||||||||||||||||||||||||||.|.|..||||||||||||||||

QVQLQESGPGLVKPSGTLSLTCAVSGGSISSSNWWSWVRQPPGKGLEWIGEIYHSGSTNYNPSLKSRVTISVDKSKNQFSLKLSSVTAADTAVYYCARDTEREGDAFDIWGQGTMVTVSS

^^^^^^^^^ ^^^^^^^ ^^^^^^^^^^^^^

Best Alignment of Ganitumab light chain to a sequence from OAS

DVVMTQSPLSLPVTPGEPASISCRSSQSLLHSNGYNYLDWYLQKPGQSPQLLIYLGSNRASGVPDRFSGSGSGTDFTLKISRVEAEDVGVYYCMQGTHWPLTFGQGTKVEIK

|.||||||||||||||||||||||||||||||||||||||||||||||||||||||||||||||||||||||||||||||||||||||||||||||||||||||||||||||

DIVMTQSPLSLPVTPGEPASISCRSSQSLLHSNGYNYLDWYLQKPGQSPQLLIYLGSNRASGVPDRFSGSGSGTDFTLKISRVEAEDVGVYYCMQGTHWPLTFGQGTKVEIK

^^^^^^^^^^^ ^^^ ^^^^^^^^^

Best Alignment of Ganitumab heavy chain CDRs to a sequence from OAS

QVQLQESGPGLVKPSGTLSLTCAVSGGSISSSNWWSWVRQPPGKGLEWIGEIYHSGSTNYNPSLKSRVTISVDKSKNQFSLKLSSVTAADTAVYYCARWTGRTDAFDIWGQGTMVTVSS

..............|.|||||||||||||||||||||||||||||||||||||||||||||||||||||||||||||||||||||||||||||||||||.||.||||||||||||||||

--------------SETLSLTCAVSGGSISSSNWWSWVRQPPGKGLEWIGEIYHSGSTNYNPSLKSRVTISVDKSKNQFSLKLSSVTAADTAVYYCARWPGRYDAFDIWGQGTMVTVSS

^^^^^^^^^ ^^^^^^^ ^^^^^^^^^^^^

Best Alignment of Ganitumab light chain CDRs to a sequence from OAS

DVVMTQSPLSLPVTPGEPASISCRSSQSLLHSNGYNYLDWYLQKPGQSPQLLIYLGSNRASGVPDRFSGSGSGTDFTLKISRVEAEDVGVYYCMQGTHWPLTFGQGTKVEIK

|.||||||||||||||||||||||||||||||||||||||||||||||||||||||||||||||||||||||||||||||..||||||||||||||||||||||.|||||||

DIVMTQSPLSLPVTPGEPASISCRSSQSLLHSNGYNYLDWYLQKPGQSPQLLIYLGSNRASGVPDRFSGSGSGTDFTLKITSVEAEDVGVYYCMQGTHWPLTFGGGTKVEIK

^^^^^^^^^^^ ^^^ ^^^^^^^^^

Best Alignment of Ganitumab CDR-H3 to a sequence from OAS

QVQLQESGPGLVKPSGTLSLTCAVSGGSIS-SSNWWSWVRQPPGKGLEWIGEIYHSGSTNYNPSLKSRVTISVDKSKNQFSLKLSSVTAADTAVYYCARWTGRTDAFDIWGQGTMVTVSS

..............|.||||||.|||||||.||..|.|.|||||||||.||.||.||||.||||||||||||||.||.||.|||||||||||||||||||||.|||||||||||||||||

--------------SETLSLTCTVSGGSISSSSYYWGWLRQPPGKGLEGIGSIYYSGSTYYNPSLKSRVTISVDTSKYQFYLKLSSVTAADTAVYYCARWTGGTDAFDIWGQGTMVTVSS

^^^^^^^^^^ ^^^^^^^ ^^^^^^^^^^^^

Therapeutic : Gantenerumab

Best Alignment of Gantenerumab heavy chain to a sequence from OAS

QVELVESGGGLVQPGGSLRLSCAASGFTFSSYAMSWVRQAPGKGLEWVSAINASGTRTYYADSVKGRFTISRDNSKNTLYLQMNSLRAEDTAVYYCARGKGNTHKPYGYVRYFDVWGQGTLVTVSS

.|.||||||||||||||||||||||||||||||||||||||||||||||||..||..|||||||||||||||||||||||||||||||||||||||||||..|.....|..|||.|||||||||||

EVQLVESGGGLVQPGGSLRLSCAASGFTFSSYAMSWVRQAPGKGLEWVSAISGSGGSTYYADSVKGRFTISRDNSKNTLYLQMNSLRAEDTAVYYCARGKPDTR---TYKYYFDYWGQGTLVTVSS

^^^^^^^^ ^^^^^^^^ ^^^^^^^^^^^^^^^^^^^

Best Alignment of Gantenerumab light chain to a sequence from OAS

DIVLTQSPATLSLSPGERATLSCRASQSVSSSYLAWYQQKPGQAPRLLIYGASSRATGVPARFSGSGSGTDFTLTISSLEPEDFATYYCLQIYNMPITFGQGTKVEIK

.|||||||||||||||||||||||||||||||||||||||||||||||||||||||||.||||||||||||||||||||||||||||||.|.||.|.|||||||||||

EIVLTQSPATLSLSPGERATLSCRASQSVSSSYLAWYQQKPGQAPRLLIYGASSRATGIPARFSGSGSGTDFTLTISSLEPEDFATYYCQQNYNAPWTFGQGTKVEIK

^^^^^^^ ^^^ ^^^^^^^^^

Best Alignment of Gantenerumab heavy chain CDRs to a sequence from OAS

QVELVESGGGLVQPGGSLRLSCAASGFTFSSYAMSWVRQAPGKGLEWVSAINASGTRTYYADSVKGRFTISRDNSKNTLYLQMNSLRAEDTAVYYCARGKGNTHKPYGYVRYFDVWGQGTLVTVSS

.............||||||||||||||||||||||||||||||||||||||.|||.||..||||.||||||||||.||||||||||||||||.||||......|.|.||.|||||||.||||.|||

-------------PGGSLRLSCAASGFTFSSYAMSWVRQAPGKGLEWVSAISASGSRTHFADSVAGRFTISRDNSRNTLYLQMNSLRAEDTAIYYCAKDRVYSHDPSGYPRYFDVWGRGTLVAVSS

^^^^^^^^ ^^^^^^^^ ^^^^^^^^^^^^^^^^^^^

Best Alignment of Gantenerumab light chain CDRs to a sequence from OAS

DIVLTQSPATLSLSPGERATLSCRASQSVSSSYLAWYQQKPGQAPRLLIYGASSRATGVPARFSGSGSGTDFTLTISSLEPEDFATYYCLQIYNMPITFGQGTKVEIK

.|||||||.||||||||||||||||||||||||||||.||||||||||.||||.||||.|||||||||||.||||||||..||||||||||.||.||||||||..|||

EIVLTQSPGTLSLSPGERATLSCRASQSVSSSYLAWYRQKPGQAPRLLMYGASTRATGIPARFSGSGSGTEFTLTISSLQSEDFATYYCLQDYNYPITFGQGTRLEIK

^^^^^^^ ^^^ ^^^^^^^^^

Best Alignment of Gantenerumab CDR-H3 to a sequence from OAS

QVELVESGGGLVQPGGSLRLSCAASGFTFSSYAMSWVRQAPGKGLEWVSAINASGTRTYYADSVKGRFTISRDNSKNTLYLQMNSLRAEDTAVYYCARGKGNTHKPYGYVRYFDVWGQGTLVTVSS

.................|.|.||..|...|.|..||.||.|||||||...||.|...|.|..|.|.|.|||.|.|||...|...|..|.||||||||||.|.....||||||||.||.||||||||

--------------SETLSLTCAVYGGSYSGYYWSWIRQPPGKGLEWIGEINHS-GSTNYNPSLKSRVTISVDTSKNQFSLKLSSVTAADTAVYYCARGRGRSGYSYGYVRYFDLWGRGTLVTVSS

^^^^^^^^ ^^^^^^^^ ^^^^^^^^^^^^^^^^^^^

Therapeutic : Gedivumab

Best Alignment of Gedivumab heavy chain to a sequence from OAS

EVQLVESGGGVVQPGKSLRLSCAASGLTFSSYAVHWVRQAPGKGLEWVTLISYDGANQYYADSVKGRFTISRDNSKNTVYLQMNSLRPEDTAVYYCAVPGPVFGI--FPPWSYFDNWGQGILVTVSS

.||||||||||||||.||||||||||.||||||.||||||||||||||..|||||.|.||||||||||||||||||||.||||||||.|||||||||......|...|||.||||.||||.||||||

QVQLVESGGGVVQPGRSLRLSCAASGFTFSSYAMHWVRQAPGKGLEWVAVISYDGSNKYYADSVKGRFTISRDNSKNTLYLQMNSLRAEDTAVYYCARDAYGWGGYDFPPRSYFDYWGQGTLVTVSS

^^^^^^^^ ^^^^^^^^ ^^^^^^^^^^^^^^^^^^^^

Best Alignment of Gedivumab light chain to a sequence from OAS

EIVLTQSPATLSVSPGERATLSCRASQVISHNLAWYQQKPGQAPRLLIYGASTRASGIPARFSGSGSGTDYTLTITSLQSEDFAVYYCQHYSNWPPRLTFGGGTKVEIK

|||.|||||||||||||||||||||||..|.||||||||||||||||||||||||.||||||||||||||.||||||||||||||||||.|||||||||||||||||||

EIVMTQSPATLSVSPGERATLSCRASQSVSSNLAWYQQKPGQAPRLLIYGASTRATGIPARFSGSGSGTDFTLTITSLQSEDFAVYYCQQYSNWPPRLTFGGGTKVEIK

^^^^^^ ^^^ ^^^^^^^^^^^

Best Alignment of Gedivumab heavy chain CDRs to a sequence from OAS

EVQLVESGGGVVQPGKSLRLSCAASGLTFSSYAVHWVRQAPGKGLEWVTLISYDGANQYYADSVKGRFTISRDNSKNTVYLQMNSLRPEDTAVYYCAVPGPVFGIFPPWSYFDNWGQGILVTVSS

..............|.||||||||||.||||||.||||||||||||||.||||||.|.||||||||||||||||||||.||||||||.|||||||||.||..||.|.|..|||.||||.||||||

--------------GRSLRLSCAASGFTFSSYAMHWVRQAPGKGLEWVALISYDGSNKYYADSVKGRFTISRDNSKNTLYLQMNSLRAEDTAVYYCARPGSWFGEFIPGYYFDYWGQGTLVTVSS

^^^^^^^^ ^^^^^^^^ ^^^^^^^^^^^^^^^^^^

Best Alignment of Gedivumab light chain CDRs to a sequence from OAS

EIVLTQSPATLSVSPGERATLSCRASQVISHNLAWYQQKPGQAPRLLIYGASTRASGIPARFSGSGSGTDYTLTITSLQSEDFAVYYCQHYSNWPPRLTFGGGTKVEIK

|||.||||.||||||||.|||||||||.||.|||||||||||.||||.|||||||.||||||.||||||..||||.|.||.|.||||||||.|||||.|||.||...||

EIVMTQSPDTLSVSPGETATLSCRASQSISNNLAWYQQKPGQSPRLLFYGASTRATGIPARFRGSGSGTEFTLTISSIQSGDCAVYYCQHYNNWPPRITFGQGTRLAIK

^^^^^^ ^^^ ^^^^^^^^^^^

Best Alignment of Gedivumab CDR-H3 to a sequence from OAS

EVQLVESGGGVVQPGKSLRLSCAASGLTFSSYAVHWVRQAPGKGLEWVTLISYDGANQYYADSVKGRFTISRDNSKNTVYLQMNSLRPEDTAVYYCAVPGPVFGIFPPWSYFDNWGQGILVTVSS

.............||.||..||||||.||..||.||||||||.||||||.|||.|...||||||||||||||||||.|.||||.|||.||.||||||..||.|||.|..|.||.||||..|.|||

-------------PGESLKISCAASGFTFNNYAMHWVRQAPGQGLEWVTVISYNGNDKYYADSVKGRFTISRDNSKDTLYLQMRSLRAEDSAVYYCATGGPIFGIRPITSAFDIWGQGTMVSVSS

^^^^^^^^ ^^^^^^^^ ^^^^^^^^^^^^^^^^^^

Therapeutic : Gemtuzumab

Best Alignment of Gemtuzumab heavy chain to a sequence from OAS

EVQLVQSGAEVKKPGSSVKVSCKASGYTITDSNIHWVRQAPGQSLEWIGYIYPYNGGTDYNQKFKNRATLTVDNPTNTAYMELSSLRSEDTAFYYCVNGNPWLAYWGQGTLVTVSS

||||..||.|..|||.|||.||||||||.||.||||..|.||||||||||||||||||.||||||||||||||...|||||.|.||.|||.|.|||.||..|.||||||||||||.

EVQLQESGPELVKPGASVKISCKASGYTFTDNNIHWLKQSPGQSLEWIGYIYPYNGGTGYNQKFKNRATLTVDDSSNTAYMDLRSLTSEDSAVYYCANGYAWFAYWGQGTLVTVSA

^^^^^^^^ ^^^^^^^^ ^^^^^^^^^

Best Alignment of Gemtuzumab light chain to a sequence from OAS

DIQLTQSPSTLSASVGDRVTITCRASESLDNYGIRFLTWFQQKPGKAPKLLMYAASNQGSGVPSRFSGSGSGTEFTLTISSLQPDDFATYYCQQTKEVPWSFGQGTKVEVK

|||.||||||||||||||||||||||.|........|||.|||||||||||.|.|||..|||||||||||||||||||||||||||||||||||.|..||.||||||||||

DIQMTQSPSTLSASVGDRVTITCRASQSI----SSWLTWYQQKPGKAPKLLIYKASNLESGVPSRFSGSGSGTEFTLTISSLQPDDFATYYCQQYKSYPWTFGQGTKVEVK

^^^^^^^^^^ ^^^ ^^^^^^^^^

Best Alignment of Gemtuzumab heavy chain CDRs to a sequence from OAS

EVQLVQSGAEVKKPGSSVKVSCKASGYTITDSNIHWVRQAPGQSLEWIGYIYPYNGGTDYNQKFKNRATLTVDNPTNTAYMELSSLRSEDTAFYYCVNGNPWLAYWGQGTLVTVSS

.|||.|||.|..|||.|||.||||||||.||.|.|||.|..|.|||||||||||||||.||||||..|||||||...||||||.||.|||.|.|||.|||.|.||||||||||||.

-VQLQQSGPELVKPGASVKISCKASGYTFTDYNMHWVKQSHGKSLEWIGYIYPYNGGTGYNQKFKSKATLTVDNSSSTAYMELRSLTSEDSAVYYCANGNAWFAYWGQGTLVTVSA

^^^^^^^^ ^^^^^^^^ ^^^^^^^^^

Best Alignment of Gemtuzumab light chain CDRs to a sequence from OAS

DIQLTQSPSTLSASVGDRVTITCRASESLDNYGIRFLTWFQQKPGKAPKLLMYAASNQGSGVPSRFSGSGSGTEFTLTISSLQPDDFATYYCQQTKEVPWSFGQGTKVEVK

||.|||||..|..|.|.|.||.||||||.|||||||..|||||.|..||||.|||||||||||.|||||||||.|.|.|.....||.|.|.|||.|||||.||.|||.|.|

DIVLTQSPASLAVSLGQRATISCRASESVDNYGIRFMNWFQQKTGQPPKLLIYAASNQGSGVPARFSGSGSGTDFSLNIHPMEEDDTAMYFCQQSKEVPWTFGGGTKLEIK

^^^^^^^^^^ ^^^ ^^^^^^^^^

Best Alignment of Gemtuzumab CDR-H3 to a sequence from OAS

EVQLVQSGAEVKKPGSSVKVSCKASGYTITDSNIHWVRQAPGQSLEWIGYIYPYNGGTDYNQKFKNRATLTVDNPTNTAYMELSSLRSEDTAFYYCVNGNPWLAYWGQGTLVTVSS

.|||.|||||..|||.|||.||.|||..|.|...|||.|.|.|.|||||.|.|.||.|.|..||...||.|.|...||||..||||.|||||.|||||||||.||||||||||||.

-VQLQQSGAELVKPGASVKLSCTASGFNIKDTYMHWVKQRPEQGLEWIGRIDPANGNTKYDPKFQGKATITADTSSNTAYLQLSSLTSEDTAVYYCVNGNPWFAYWGQGTLVTVSA

^^^^^^^^ ^^^^^^^^ ^^^^^^^^^

Therapeutic : Gevokizumab

Best Alignment of Gevokizumab heavy chain to a sequence from OAS

QVQLQESGPGLVKPSQTLSLTCSFSGFSLSTSGMGVGWIRQPSGKGLEWLAHIWWDGDESYNPSLKSRLTISKDTSKNQVSLKITSVTAADTAVYFCARNRYDPPWFVDWGQGTLVTVSS

||.|.|||||..||||||||||||||||||||||||||||||||||||||||||||.|..|||||||.||||||||.|||.||||||..||||.|.|||.|||.|||..||||||||||.

QVTLKESGPGILKPSQTLSLTCSFSGFSLSTSGMGVGWIRQPSGKGLEWLAHIWWDDDKYYNPSLKSQLTISKDTSRNQVFLKITSVDTADTATYYCARRRYDGPWFAYWGQGTLVTVSA

^^^^^^^^^^ ^^^^^^^ ^^^^^^^^^^^^

Best Alignment of Gevokizumab light chain to a sequence from OAS

DIQMTQSTSSLSASVGDRVTITCRASQDISNYLSWYQQKPGKAVKLLIYYTSKLHSGVPSRFSGSGSGTDYTLTISSLQQEDFATYFCLQGKMLPWTFGQGTKLEIK

|||||||.|||||||||||||||||||.|||||||||||||||.|||||..|.|.|||||||||||||||.||||||||.||||||.|||.|..|||||||||.|||

DIQMTQSPSSLSASVGDRVTITCRASQGISNYLSWYQQKPGKAPKLLIYDASTLQSGVPSRFSGSGSGTDFTLTISSLQPEDFATYYCLQYKSDPWTFGQGTKVEIK

^^^^^^ ^^^ ^^^^^^^^^

Best Alignment of Gevokizumab heavy chain CDRs to a sequence from OAS

QVQLQESGPGLVKPSQTLSLTCSFSGFSLSTSGMGVGWIRQPSGKGLEWLAHIWWDGDESYNPSLKSRLTISKDTSKNQVSLKITSVTAADTAVYFCARNRYDPPWFVDWGQGTLVTVSS

||.|.|||||..||||||||||||||||||||||||||||||||||||||||||||.|..|||||||.||||||||.|||.||||||..||||.|.|||.|||.|||..||||||||||.

QVTLKESGPGILKPSQTLSLTCSFSGFSLSTSGMGVGWIRQPSGKGLEWLAHIWWDDDKYYNPSLKSQLTISKDTSRNQVFLKITSVDTADTATYYCARRRYDGPWFAYWGQGTLVTVSA

^^^^^^^^^^ ^^^^^^^ ^^^^^^^^^^^^

Best Alignment of Gevokizumab light chain CDRs to a sequence from OAS

DIQMTQSTSSLSASVGDRVTITCRASQDISNYLSWYQQKPGKAVKLLIYYTSKLHSGVPSRFSGSGSGTDYTLTISSLQQEDFATYFCLQGKMLPWTFGQGTKLEIK

|||||..||.|||..||||||.|||||||||||.||||||...|||||||||..||||||.||||||||||.||||.|.|||.|||||.||.|||||||.|||||||

DIQMTWPTSPLSAPLGDRVTISCRASQDISNYLNWYQQKPDGTVKLLIYYTSRIHSGVPSTFSGSGSGTDYSLTISNLEQEDIATYFCQQGNMLPWTFGGGTKLEIK

^^^^^^ ^^^ ^^^^^^^^^

Best Alignment of Gevokizumab CDR-H3 to a sequence from OAS

QVQLQESGPGLVKPSQTLSLTCSFSGFSLSTSGMGVGWIRQPSGKGLEWLAHIWWDGDESYNPSLKSRLTISKDTSKNQVSLKITSVTAADTAVYFCARNRYDPPWFVDWGQGTLVTVSS

.|||.||||.|||||||||||||..|.|.........|||...|..||....|...|...||||||||..|..||||||..|...||||.|||.|.|||||||..|||.||||||||||.

EVQLVESGPSLVKPSQTLSLTCSVTGDSI--TSGYWNWIRKFPGNKLEYMGYINYSGNTYYNPSLKSRISITRDTSKNQFYLQLNSVTADDTATYYCARNRYDEAWFVYWGQGTLVTVSP

^^^^^^^^^^ ^^^^^^^ ^^^^^^^^^^^^

Therapeutic : Girentuximab

Best Alignment of Girentuximab heavy chain to a sequence from OAS

DVKLVESGGGLVKLGGSLKLSCAASGFTFSNYYMSWVRQTPEKRLELVAAINSDGGITYYLDTVKGRFTISRDNAKNTLYLQMSSLKSEDTALFYCARHRSG---YFSMDYWGQGTSVTVSS

||||||||||||||||||||||||||||||.||||||||||||||||||||||.||.|||.||||||||||||||||||||||||||||||||.||||||.|...|..||||||||||||||

DVKLVESGGGLVKLGGSLKLSCAASGFTFSSYYMSWVRQTPEKRLELVAAINSNGGSTYYPDTVKGRFTISRDNAKNTLYLQMSSLKSEDTALYYCARHRDGNYDYYAMDYWGQGTSVTVSS

^^^^^^^^ ^^^^^^^^ ^^^^^^^^^^^^^^^

Best Alignment of Girentuximab light chain to a sequence from OAS

DIVMTQSQRFMSTTVGDRVSITCKASQNVVSAVAWYQQKPGQSPKLLIYSASNRYTGVPDRFTGSGSGTDFTLTISNMQSEDLADFFCQQYSNYPWTFGGGTKLEIK

||||||||.||||||||||||||||||||..||||||||||||||||||||||||||||||||||||||||||||||||||||||.||||||.||||||||||||||

DIVMTQSQKFMSTTVGDRVSITCKASQNVGTAVAWYQQKPGQSPKLLIYSASNRYTGVPDRFTGSGSGTDFTLTISNMQSEDLADYFCQQYSSYPWTFGGGTKLEIK

^^^^^^ ^^^ ^^^^^^^^^

Best Alignment of Girentuximab heavy chain CDRs to a sequence from OAS

DVKLVESGGGLVKLGGSLKLSCAASGFTFSNYYMSWVRQTPEKRLELVAAINSDGGITYYLDTVKGRFTISRDNAKNTLYLQMSSLKSEDTALFYCARHRSGYFSMDYWGQGTSVTVSS

.|.||||.||||..|.|.||||.|||||||.|||.||||.|||.||.||.||.||..|||||..|.||.||||||||.||||||||.|.|||..||||.|.||||||||||||||||||

EVHLVESEGGLVQPGSSMKLSCTASGFTFSDYYMAWVRQVPEKGLEWVANINYDGSSTYYLDSLKSRFIISRDNAKNILYLQMSSLQSDDTATYYCARERGGYFSMDYWGQGTSVTVSS

^^^^^^^^ ^^^^^^^^ ^^^^^^^^^^^^

Best Alignment of Girentuximab light chain CDRs to a sequence from OAS

DIVMTQSQRFMSTTVGDRVSITCKASQNVVSAVAWYQQKPGQSPKLLIYSASNRYTGVPDRFTGSGSGTDFTLTISNMQSEDLADFFCQQYSNYPWTFGGGTKLEIK

||||||||.||||.|||||..||||||||.|.||||.|||||||..||.|||.||.|||||||||||||||||||.|.||||||..|||||||||||||||||||||

DIVMTQSQKFMSTSVGDRVTVTCKASQNVGSNVAWYEQKPGQSPTALIHSASYRYSGVPDRFTGSGSGTDFTLTINNVQSEDLAEYFCQQYSNYPWTFGGGTKLEIK

^^^^^^ ^^^ ^^^^^^^^^

Best Alignment of Girentuximab CDR-H3 to a sequence from OAS

DVKLVESGGGLVKLGGSLKLSCAASGFTFSNYYMSWVRQTPEKRLELVAAINSDGGITYYLDTVKGRFTISRDNAKNTLYLQMSSLKSEDTALFYCARHRSGYFSMDYWGQGTSVTVSS

.||||||||.|||.||||||||||||||||.|.||||||||.||||.||.|.|.|..|||.|.|||||||||||||||||||||||||||||..||||||.||||||||||||||||||

EVKLVESGGDLVKPGGSLKLSCAASGFTFSSYGMSWVRQTPDKRLEWVATISSGGSYTYYPDSVKGRFTISRDNAKNTLYLQMSSLKSEDTAMYYCARHRDGYFSMDYWGQGTSVTVSS

^^^^^^^^ ^^^^^^^^ ^^^^^^^^^^^^

Therapeutic : Glembatumumab

Best Alignment of Glembatumumab heavy chain to a sequence from OAS

QVQLQESGPGLVKPSQTLSLTCTVSGGSISSFNYYWSWIRHHPGKGLEWIGYIYYSGSTYSNPSLKSRVTISVDTSKNQFSLTLSSVTAADTAVYYCARGYNW------NYFDYWGQGTLVTVSS

|||||||||||||||||||||||||||||||..|||||||.|||||||||||||||||||.|||||||||||||||||||||.||||||||||||||||||........||||||||||||||||

QVQLQESGPGLVKPSQTLSLTCTVSGGSISSGGYYWSWIRQHPGKGLEWIGYIYYSGSTYYNPSLKSRVTISVDTSKNQFSLKLSSVTAADTAVYYCARGYLSYYTRIRNYFDYWGQGTLVTVSS

^^^^^^^^^^ ^^^^^^^ ^^^^^^^^^^^^^^^^^

Best Alignment of Glembatumumab light chain to a sequence from OAS

EIVMTQSPATLSVSPGERATLSCRASQSVDNNLVWYQQKPGQAPRLLIYGASTRATGIPARFSGSGSGTEFTLTISSLQSEDFAVYYCQQYNNWPPWTFGQGTKVEIK

|||||||||||||||||||||||||||||.||||||||||||||||||||||||||||||||||||||||||||||||||||||||||||||||||||||||||||||

EIVMTQSPATLSVSPGERATLSCRASQSVSNNLVWYQQKPGQAPRLLIYGASTRATGIPARFSGSGSGTEFTLTISSLQSEDFAVYYCQQYNNWPPWTFGQGTKVEIK

^^^^^^ ^^^ ^^^^^^^^^^

Best Alignment of Glembatumumab heavy chain CDRs to a sequence from OAS

QVQLQESGPGLVKPSQTLSLTCTVSGGSISSFNYYWSWIRHHPGKGLEWIGYIYYSGSTYSNPSLKSRVTISVDTSKNQFSLTLSSVTAADTAVYYCARGYNWNYFDYWGQGTLVTVSS

..............|.|||||||||||||||..|||.|||..|||||||||||||||||..|||||||||||||||||||||.||||||||||||||||||||||||||||||||||||

--------------SETLSLTCTVSGGSISSSSYYWGWIRQPPGKGLEWIGYIYYSGSTNYNPSLKSRVTISVDTSKNQFSLKLSSVTAADTAVYYCARGYNWNYFDYWGQGTLVTVSS

^^^^^^^^^^ ^^^^^^^ ^^^^^^^^^^^

Best Alignment of Glembatumumab light chain CDRs to a sequence from OAS

EIVMTQSPATLSVSPGERATLSCRASQSVDNNLVWYQQKPGQAPRLLIYGASTRATGIPARFSGSGSGTEFTLTISSLQSEDFAVYYCQQYNNWPPWTFGQGTKVEIK

||||||||||||||||||||||||||||||||..|||||||||||||||||||||||||||||||||||||||||||.||||||||||||||||||||||||||||||

EIVMTQSPATLSVSPGERATLSCRASQSVDNNFAWYQQKPGQAPRLLIYGASTRATGIPARFSGSGSGTEFTLTISSRQSEDFAVYYCQQYNNWPPWTFGQGTKVEIK

^^^^^^ ^^^ ^^^^^^^^^^

Best Alignment of Glembatumumab CDR-H3 to a sequence from OAS

QVQLQESGPGLVKPSQTLSLTCTVSGGSISSFNYYWSWIRHHPGKGLEWIGYIYYS-GSTYSNPSLKSRVTISVDTSKNQFSLTLSSVTAADTAVYYCARGYNWNYFDYWGQGTLVTVSS

.....................|..||.......|...|.|..||..|||.|.|....|.|........||||..|||.......|.|....||||||||||||||||||.||||||||||

----------------SVKVSCKASGYTF--TTYAIHWVRQAPGQRLEWMGWINAGNGNTKYAQKFQGRVTITRDTSASTAYMELRSLRSEDTAVYYCARGYNWNYFDYCGQGTLVTVSS

^^^^^^^^^^ ^^^^^^^^ ^^^^^^^^^^^

Therapeutic : Golimumab

Best Alignment of Golimumab heavy chain to a sequence from OAS

QVQLVESGGGVVQPGRSLRLSCAASGFIFSSYAMHWVRQAPGNGLEWVAFMSYDGSNKKYADSVKGRFTISRDNSKNTLYLQMNSLRAEDTAVYYCARDRGIAAGGNYYYYGMDVWGQGTTVTVSS

|||||||||||||||||||||||||||.||||||||||||||.||||||..|||||||.|||||||||||||||||||||||||||||||||||||||||||||.|..||||||||||||||||||

QVQLVESGGGVVQPGRSLRLSCAASGFTFSSYAMHWVRQAPGKGLEWVAVISYDGSNKYYADSVKGRFTISRDNSKNTLYLQMNSLRAEDTAVYYCARDRGIAAAGLRYYYGMDVWGQGTTVTVSS

^^^^^^^^ ^^^^^^^^ ^^^^^^^^^^^^^^^^^^^

Best Alignment of Golimumab light chain to a sequence from OAS

EIVLTQSPATLSLSPGERATLSCRASQSVYSYLAWYQQKPGQAPRLLIYDASNRATGIPARFSGSGSGTDFTLTISSLEPEDFAVYYCQQRSNWPPFTFGPGTKVDIK

|||||||||||||||||||||||||||||.||||||||||||||||||||||||||||||||||||||||||||||||||||||||||||||||||||||||||||||

EIVLTQSPATLSLSPGERATLSCRASQSVSSYLAWYQQKPGQAPRLLIYDASNRATGIPARFSGSGSGTDFTLTISSLEPEDFAVYYCQQRSNWPPFTFGPGTKVDIK

^^^^^^ ^^^ ^^^^^^^^^^

Best Alignment of Golimumab heavy chain CDRs to a sequence from OAS

QVQLVESGGGVVQPGRSLRLSCAASGFIFSSYAMHWVRQAPGNGLEWVAFMSYDGSNKKYADSVKGRFTISRDNSKNTLYLQMNSLRAEDTAVYYCARDRGIAAGGNYYYYGMDVWGQGTTVTVSS

................|||||||||||.||||||||||||||.||||||..|||||||.|||||||||||||||||||||||||||||.|||||||||||||||.|.|||||||||||||||||||

----------------SLRLSCAASGFTFSSYAMHWVRQAPGKGLEWVAVISYDGSNKYYADSVKGRFTISRDNSKNTLYLQMNSLRAADTAVYYCARDRGIAAAGYYYYYGMDVWGQGTTVTVSS

^^^^^^^^ ^^^^^^^^ ^^^^^^^^^^^^^^^^^^^

Best Alignment of Golimumab light chain CDRs to a sequence from OAS

EIVLTQSPATLSLSPGERATLSCRASQSVYSYLAWYQQKPGQAPRLLIYDASNRATGIPARFSGSGSGTDFTLTISSLEPEDFAVYYCQQRSNWPPFTFGPGTKVDIK

|||||||||||||||||||||||||||||.||||||||||||||||||||||||||||||||||||||||||||||||||||||||||||||||||||||.||||.||

EIVLTQSPATLSLSPGERATLSCRASQSVSSYLAWYQQKPGQAPRLLIYDASNRATGIPARFSGSGSGTDFTLTISSLEPEDFAVYYCQQRSNWPPFTFGGGTKVEIK

^^^^^^ ^^^ ^^^^^^^^^^

Best Alignment of Golimumab CDR-H3 to a sequence from OAS

QVQLVESGGGVVQPGRSLRLSCAASGFIFSSYAMHWVRQAPGNGLEWVAFMSYDGSNKKYADSVKGRFTISRDNSKNTLYLQMNSLRAEDTAVYYCARDRGIAAGGNYYYYGMDVWGQGTTVTVSS

.............|...|.|.|..||...|||...|.||.||.||||.....|......|..|.|.|.|||.|.|||...|...|..|.|||||||||||||||.||||||||||||||||||||.

-------------PSETLSLTCTVSGGSISSYYWSWIRQPPGKGLEWIGYIYYS-GSTNYNPSLKSRVTISVDTSKNQFSLKLSSVTAADTAVYYCARDRGIAAAGNYYYYGMDVWGQGTTVTVS-

^^^^^^^^ ^^^^^^^^ ^^^^^^^^^^^^^^^^^^^

Therapeutic : Guselkumab

Best Alignment of Guselkumab heavy chain to a sequence from OAS

EVQLVQSGAEVKKPGESLKISCKGSGYSFSNYWIGWVRQMPGKGLEWMGIIDPSNSYTRYSPSFQGQVTISADKSISTAYLQWSSLKASDTAMYYCARWYY----KPFDVWGQGTLVTVSS

|||||||||||||||||||||||||||||.|||||||||||||||||||||||..|.|||||||||||||||||||||||||||||||||||||||||..|.....|||.|||||||||||

EVQLVQSGAEVKKPGESLKISCKGSGYSFTNYWIGWVRQMPGKGLEWMGIIDPGDSDTRYSPSFQGQVTISADKSISTAYLQWSSLKASDTAMYYCARHSYSNYAPPFDYWGQGTLVTVSS

^^^^^^^^ ^^^^^^^^ ^^^^^^^^^^^^^^

Best Alignment of Guselkumab light chain to a sequence from OAS

QSVLTQPPSVSGAPGQRVTISCTGSSSNIGSGYDVHWYQQLPGTAPKLLIYGNSKRPSGVPDRFSGSKSGTSASLAITGLQSEDEADYYCASWTDGLSLVVFGGGTKLTVL

||||||||||||||||||||||||||||||.|||||||||||||||||||||||.||||||||||||||||||||||||||.|||||||||||.|.||.||||||||||||

QSVLTQPPSVSGAPGQRVTISCTGSSSNIGAGYDVHWYQQLPGTAPKLLIYGNSNRPSGVPDRFSGSKSGTSASLAITGLQAEDEADYYCASWDDSLSGVVFGGGTKLTVL

^^^^^^^^^ ^^^ ^^^^^^^^^^^

Best Alignment of Guselkumab heavy chain CDRs to a sequence from OAS

EVQLVQSGAEVKKPGESLKISCKGSGYSFSNYWIGWVRQMPGKGLEWMGIIDPSNSYTRYSPSFQGQVTISADKSISTAYLQWSSLKASDTAMYYCARWYYKPFDVWGQGTLVTVSS

..............||||||||||||||||||||.||||||||||||||.||||.|||.|||||||.|||||||||||||||||||||||||||||||.||...|||||||.|||||

--------------GESLKISCKGSGYSFSNYWISWVRQMPGKGLEWMGRIDPSDSYTNYSPSFQGHVTISADKSISTAYLQWSSLKASDTAMYYCARLYYYAMDVWGQGTTVTVSS

^^^^^^^^ ^^^^^^^^ ^^^^^^^^^^

Best Alignment of Guselkumab light chain CDRs to a sequence from OAS

QSVLTQPPSVSGAPGQRVTISCTGSSSNIGSGYDVHWYQQLPGTAPKLLIYGNSKRPSGVPDRFSGSKSGTSASLAITGLQSEDEADYYCASWTDGLSLVVFGGGTKLTVL

||||||||||||||||||||||||||||||.||||||||||||.||||||||||.||||||||||||||||||||||||||.|||||||||||.|.||.||||||||||||

QSVLTQPPSVSGAPGQRVTISCTGSSSNIGAGYDVHWYQQLPGAAPKLLIYGNSNRPSGVPDRFSGSKSGTSASLAITGLQAEDEADYYCASWDDSLSGVVFGGGTKLTVL

^^^^^^^^^ ^^^ ^^^^^^^^^^^

Best Alignment of Guselkumab CDR-H3 to a sequence from OAS

EVQLVQSGAEVKKPGESLKISCKGSGYSFSNYWIGWVRQMPGKGLEWMGIIDPSNSYTRYSPSFQGQVTISADKSISTAYLQWSSLKASDTAMYYCARWYYKPFDVWGQGTLVTVSS

.|||.|.|||...||.|.|.|||.|||.|..||..||.|.||.||||.|.|.||.|.|.|...|....|...|||.||||.|.|||...|.|.||||||||.||||||.||.|||||

QVQLQQPGAELVRPGSSVKLSCKASGYTFTSYWMDWVKQRPGQGLEWIGNIYPSDSETHYNQKFKDKATLTVDKSSSTAYMQLSSLTSEDSAVYYCARWYYGPFDVWGTGTTVTVSS

^^^^^^^^ ^^^^^^^^ ^^^^^^^^^^

Therapeutic : Ianalumab

Best Alignment of Ianalumab heavy chain to a sequence from OAS

QVQLQQSGPGLVKPSQTLSLTCAISGDSVSSNSAAWGWIRQSPGRGLEWLGRIYYRSKWYNSYAVSVKSRITINPDTSKNQFSLQLNSVTPEDTAVYYCARYQWVP--KIGVFDSWGQGTLVTVSS

||||||||||||||||||||||||||||||||||||.||||||.||||||||.||||||||.|||||||||||||||||||||||||||||||||||||||..|......||||.|||||||||||

QVQLQQSGPGLVKPSQTLSLTCAISGDSVSSNSAAWNWIRQSPSRGLEWLGRTYYRSKWYNDYAVSVKSRITINPDTSKNQFSLQLNSVTPEDTAVYYCARESWGGGWYKGVFDYWGQGTLVTVSS

^^^^^^^^^^ ^^^^^^^^^ ^^^^^^^^^^^^^^^^

Best Alignment of Ianalumab light chain to a sequence from OAS

DIVLTQSPATLSLSPGERATLSCRASQFILPEYLSWYQQKPGQAPRLLIYGSSSRATGVPARFSGSGSGTDFTLTISSLEPEDFAVYYCQQFYSSPLTFGQGTKVEIK

|||||||||||||||||||||||||||.....||.||||||||||||||||.||||||||.||||||||||||||||||||||||||||||..|||.||||||.||||

DIVLTQSPATLSLSPGERATLSCRASQSV-SSYLAWYQQKPGQAPRLLIYGASSRATGVPDRFSGSGSGTDFTLTISSLEPEDFAVYYCQQYGSSPYTFGQGTRVEIK

^^^^^^^ ^^^ ^^^^^^^^^

Best Alignment of Ianalumab heavy chain CDRs to a sequence from OAS

QVQLQQSGPGLVKPSQTLSLTCAISGDSVSSNSAAWGWIRQSPGRGLEWLGRIYYRSKWYNSYAVSVKSRITINPDTSKNQFSLQLNSVTPEDTAVYYCARYQWVPKIGVFDSWGQGTLVTVSS

.....................|||||||||||||||.||||||.||||||||.||||||||.|||||.|||||||||||.|||||||||||||||||||||.|..||.|.||.|||||||||||

---------------------CAISGDSVSSNSAAWNWIRQSPSRGLEWLGRTYYRSKWYNDYAVSVESRITINPDTSKIQFSLQLNSVTPEDTAVYYCARSQITPKLGWFDPWGQGTLVTVSS

^^^^^^^^^^ ^^^^^^^^^ ^^^^^^^^^^^^^^

Best Alignment of Ianalumab light chain CDRs to a sequence from OAS

DIVLTQSPATLSLSPGERATLSCRASQFILPEYLSWYQQKPGQAPRLLIYGSSSRATGVPARFSGSGSGTDFTLTISSLEPEDFAVYYCQQFYSSPLTFGQGTKVEIK

.|||||||.||||||||||||||||||.||..||.||||||||||||||||||.||||||.|||||||||.||||||.||||||||||||||..||||||.|||.|||

EIVLTQSPGTLSLSPGERATLSCRASQTILRSYLAWYQQKPGQAPRLLIYGSSNRATGVPDRFSGSGSGTEFTLTISRLEPEDFAVYYCQQFETSPLTFGGGTKLEIK

^^^^^^^ ^^^ ^^^^^^^^^

Best Alignment of Ianalumab CDR-H3 to a sequence from OAS

QVQLQQSGPGLVKPSQTLSLTCAISGDSVSSNSAAWGWIRQSPGRGLEWLGRIYYRSKWYNSYAVSVKSRITINPDTSKNQFSLQLNSVTPEDTAVYYCARYQWVPKIGVFDSWGQGTLVTVSS

..............|.||||||..||.|.||.|..||||||.||.||||.|.|||.......|..|.|||.||..|||||||||.|.|||..|||||||||.|||..|||||.|||||||||||

--------------SETLSLTCTVSGGSISSSSYYWGWIRQPPGKGLEWIGSIYYS--GSTYYNPSLKSRVTISVDTSKNQFSLKLSSVTAADTAVYYCARDQWVATIGVFDYWGQGTLVTVSS

^^^^^^^^^^ ^^^^^^^^^ ^^^^^^^^^^^^^^

Therapeutic : Ibalizumab

Best Alignment of Ibalizumab heavy chain to a sequence from OAS

QVQLQQSGPEVVKPGASVKMSCKASGYTFTSYVIHWVRQKPGQGLDWIGYINPYNDGTDYDEKFKGKATLTSDTSTSTAYMELSSLRSEDTAVYYCAREKDNYATGAWFAYWGQGTLVTVSS

.|||||||||.||||||||||||||||||||||.|||.|||||||.||||||||||||.|.||||||||||||.|.||||||||||.|||.||||||||.|....||||||||||||||||.

EVQLQQSGPELVKPGASVKMSCKASGYTFTSYVMHWVKQKPGQGLEWIGYINPYNDGTKYNEKFKGKATLTSDKSSSTAYMELSSLTSEDSAVYYCAREWDYD--GAWFAYWGQGTLVTVSA

^^^^^^^^ ^^^^^^^^ ^^^^^^^^^^^^^^^

Best Alignment of Ibalizumab light chain to a sequence from OAS

DIVMTQSPDSLAVSLGERVTMNCKSSQSLLYSTNQKNYLAWYQQKPGQSPKLLIYWASTRESGVPDRFSGSGSGTDFTLTISSVQAEDVAVYYCQQYYSYRTFGGGTKLEIK

||||.|||.|||||.||.|||.||||||||||.|||||||||||||||||||||||||||||||||||.|||||||||||||||||||.|||||||||||||||||||||||

DIVMSQSPSSLAVSVGEKVTMSCKSSQSLLYSSNQKNYLAWYQQKPGQSPKLLIYWASTRESGVPDRFTGSGSGTDFTLTISSVQAEDLAVYYCQQYYSYRTFGGGTKLEIK

^^^^^^^^^^^^ ^^^ ^^^^^^^^

Best Alignment of Ibalizumab heavy chain CDRs to a sequence from OAS

QVQLQQSGPEVVKPGASVKMSCKASGYTFTSYVIHWVRQKPGQGLDWIGYINPYNDGTDYDEKFKGKATLTSDTSTSTAYMELSSLRSEDTAVYYCAREKDNYATGAWFAYWGQGTLVTVSS

.|||||||||.||||||||||||||||||||||.|||.|||||||.||||||||||||.|.||||||||||||.|.||||||||||.|||.|||||||..|.|.|||||||||||||||||.

EVQLQQSGPELVKPGASVKMSCKASGYTFTSYVMHWVKQKPGQGLEWIGYINPYNDGTKYNEKFKGKATLTSDKSSSTAYMELSSLTSEDSAVYYCARGGDYYGTGAWFAYWGQGTLVTVSA

^^^^^^^^ ^^^^^^^^ ^^^^^^^^^^^^^^^

Best Alignment of Ibalizumab light chain CDRs to a sequence from OAS

DIVMTQSPDSLAVSLGERVTMNCKSSQSLLYSTNQKNYLAWYQQKPGQSPKLLIYWASTRESGVPDRFSGSGSGTDFTLTISSVQAEDVAVYYCQQYYSYRTFGGGTKLEIK

||.|.|||.|||||.||..||.|.||||||||.||||||||||||||||||||||||||||||.||||.|||||||||||||||.|||.|||||||||||||||.|||||.|

DIAMLQSPSSLAVSVGEKATMSCNSSQSLLYSSNQKNYLAWYQQKPGQSPKLLIYWASTRESGIPDRFTGSGSGTDFTLTISSVKAEDLAVYYCQQYYSYRTFGAGTKLELK

^^^^^^^^^^^^ ^^^ ^^^^^^^^

Best Alignment of Ibalizumab CDR-H3 to a sequence from OAS

QVQLQQSGPEVVKPGASVKMSCKASGYTFTSYVIHWVRQKPGQGLDWIGYINPYNDGTDYDEKFKGKATLTSDTSTSTAYMELSSLRSEDTAVYYCAREKDNYATGAWFAYWGQGTLVTVSS

.|||||.|||.||||||||.|||||||.||.|..|||.|.....|.|||.|||||..|.|...||.||.||.|.|.|||||||.||.|||.||||||||.|.||.||||||||||||||||.

-VQLQQFGPELVKPGASVKISCKASGYSFTGYYMHWVKQSHVKSLEWIGRINPYNGATSYNQNFKDKASLTVDKSSSTAYMELHSLTSEDSAVYYCAREEDYYASGAWFAYWGQGTLVTVSA

^^^^^^^^ ^^^^^^^^ ^^^^^^^^^^^^^^^

Therapeutic : Icrucumab

Best Alignment of Icrucumab heavy chain to a sequence from OAS

QAQVVESGGGVVQSGRSLRLSCAASGFAFSSYGMHWVRQAPGKGLEWVAVIWYDGSNKYYADSVRGRFTISRDNSENTLYLQMNSLRAEDTAVYYCARDHYGSGVH---HYFYYGLDVWGQGTTVTVSS

|.|.|||||||||.|||||||||||||.||||||||||||||||||||||||||||||||||||.||||||||||.|||||||||||||||||||||||.||||.....||.|||.|||||||||||||

QVQLVESGGGVVQPGRSLRLSCAASGFTFSSYGMHWVRQAPGKGLEWVAVIWYDGSNKYYADSVKGRFTISRDNSKNTLYLQMNSLRAEDTAVYYCARDYYGSGSYYSFHYYYYGVDVWGQGTTVTVSS

^^^^^^^^ ^^^^^^^^ ^^^^^^^^^^^^^^^^^^^^^^

Best Alignment of Icrucumab light chain to a sequence from OAS

EIVLTQSPGTLSLSPGERATLSCRASQSVSSSYLAWYQQKPGQAPRLLIYGASSRATGIPDRFSGSGSGTDFTLTISRLEPEDFAVYYCQQYGSSPLTFGGGTKVEIK

||||||||||||||||||||||||||||||||||||||||||||||||||||||||||||||||||||||||||||||||||||||||||||||||||||||||||||

EIVLTQSPGTLSLSPGERATLSCRASQSVSSSYLAWYQQKPGQAPRLLIYGASSRATGIPDRFSGSGSGTDFTLTISRLEPEDFAVYYCQQYGSSPLTFGGGTKVEIK

^^^^^^^ ^^^ ^^^^^^^^^

Best Alignment of Icrucumab heavy chain CDRs to a sequence from OAS

QAQVVESGGGVVQSGRSLRLSCAASGFAFSSYGMHWVRQAPGKGLEWVAVIWYDGSNKYYADSVRGRFTISRDNSENTLYLQMNSLRAEDTAVYYCARDHYGSGVHHYFYYGLDVWGQGTTVTVSS

|.|.|||||||||.|||||||||||||.||||||||||||||||||||||||||||||||||||.||||||||||.||||||||||||||||||||||||||||...|.|||.|||||||||||||

QVQLVESGGGVVQPGRSLRLSCAASGFTFSSYGMHWVRQAPGKGLEWVAVIWYDGSNKYYADSVKGRFTISRDNSKNTLYLQMNSLRAEDTAVYYCARDHYGSGHALYYYYGMDVWGQGTTVTVSS

^^^^^^^^ ^^^^^^^^ ^^^^^^^^^^^^^^^^^^^

Best Alignment of Icrucumab light chain CDRs to a sequence from OAS

EIVLTQSPGTLSLSPGERATLSCRASQSVSSSYLAWYQQKPGQAPRLLIYGASSRATGIPDRFSGSGSGTDFTLTISRLEPEDFAVYYCQQYGSSPLTFGGGTKVEIK

|||||||||||||||||||||||||||||||||||||||||||||||||||||||||||||||||||||.||||||||||||||||||||||||||||||.||..|||

EIVLTQSPGTLSLSPGERATLSCRASQSVSSSYLAWYQQKPGQAPRLLIYGASSRATGIPDRFSGSGSGADFTLTISRLEPEDFAVYYCQQYGSSPLTFGQGTRLEIK

^^^^^^^ ^^^ ^^^^^^^^^

Best Alignment of Icrucumab CDR-H3 to a sequence from OAS

QAQVVESGGGVVQSGRSLRLSCAASGFAFSSYGMHWVRQAPGKGLEWVAVIWYDGSNKYYADSVRGRFTISRDNSENTLYLQMNSLRAEDTAVYYCARDHYGSGVHHYFYYGLDVWGQGTTVTVSS

..............|.||..|||||||.||||.|.|||||||||||||..|...||..||||||.|||||||||..|.||||||||||||||||||||||||||..||.|||.|||||||||||||

--------------GESLKISCAASGFTFSSYSMNWVRQAPGKGLEWVSYISSSGSTIYYADSVKGRFTISRDNAKNSLYLQMNSLRAEDTAVYYCARDHYGSGSYHYYYYGMDVWGQGTTVTVSS

^^^^^^^^ ^^^^^^^^ ^^^^^^^^^^^^^^^^^^^

Therapeutic : Idarucizumab

Best Alignment of Idarucizumab heavy chain to a sequence from OAS

QVQLQESGPGLVKPSETLSLTCTVSGFSLTSYIVDWIRQPPGKGLEWIGVIWAGGSTGYNSALRSRVSITKDTSKNQFSLKLSSVTAADTAVYYCASAAYYSY-------YNYDGFAYWGQGTLVTVSS

||||||||||||||||||||||||||.|..||...||||||||||||||.|...|||.||..|.|||.|..||||||||||||||||||||||||||..|.|........|.||.|.||||||||||||

QVQLQESGPGLVKPSETLSLTCTVSGGSISSYYWSWIRQPPGKGLEWIGYIYYSGSTNYNPSLKSRVTISVDTSKNQFSLKLSSVTAADTAVYYCASSGYCSGGSLCGYSYGYDYFDYWGQGTLVTVSS

^^^^^^^^ ^^^^^^^ ^^^^^^^^^^^^^^^^^^^^^^^

Best Alignment of Idarucizumab light chain to a sequence from OAS

DVVMTQSPLSLPVTLGQPASISCKSSQSLLYTDGKTYLYWFLQRPGQSPRRLIYLVSKLDSGVPDRFSGSGSGTDFTLKISRVEAEDVGVYYCLQSTHFPHTFGGGTKVEIK

||||||.||.|.||.|||||||||||||||||.|||||.|.||||||||.|||||||||||||||||||||||||||||||||||||.||||||||||||||||||||.|||

DVVMTQTPLTLSVTIGQPASISCKSSQSLLYTNGKTYLNWLLQRPGQSPKRLIYLVSKLDSGVPDRFSGSGSGTDFTLKISRVEAEDLGVYYCLQSTHFPHTFGGGTKLEIK

^^^^^^^^^^^ ^^^ ^^^^^^^^^

Best Alignment of Idarucizumab heavy chain CDRs to a sequence from OAS

QVQLQESGPGLVKPSETLSLTCTVSGFSLTSYIVDWIRQPPGKGLEWIGVIWAGGSTGYNSALRSRVSITKDTSKNQFSLKLSSVTAADTAVYYCASAAYYSYYNYDGFAYWGQGTLVTVSS

|||||.||||||.||..||.||||||||||||.|||.||.|||||||.||||.||||.|||||.||.||.||.||.|..||..|....|||.|||||.||||||.||.|||||||||||||.

QVQLQQSGPGLVAPSQSLSITCTVSGFSLTSYGVDWVRQSPGKGLEWLGVIWGGGSTNYNSALKSRLSISKDNSKSQVFLKMNSLQTDDTAMYYCASSAYYSYYSYDPFAYWGQGTLVTVSA

^^^^^^^^ ^^^^^^^ ^^^^^^^^^^^^^^^^

Best Alignment of Idarucizumab light chain CDRs to a sequence from OAS

DVVMTQSPLSLPVTLGQPASISCKSSQSLLYTDGKTYLYWFLQRPGQSPRRLIYLVSKLDSGVPDRFSGSGSGTDFTLKISRVEAEDVGVYYCLQSTHFPHTFGGGTKVEIK

||||||.||.|.||.|||||||||||||||||.|||||.|.||||||||.|||||||||||||||||||||||||||||||||||||.||||||||||||||||.|||.|||

DVVMTQTPLTLSVTIGQPASISCKSSQSLLYTNGKTYLNWLLQRPGQSPKRLIYLVSKLDSGVPDRFSGSGSGTDFTLKISRVEAEDLGVYYCLQSTHFPHTFGSGTKLEIK

^^^^^^^^^^^ ^^^ ^^^^^^^^^

Best Alignment of Idarucizumab CDR-H3 to a sequence from OAS

QVQLQESGPGLVKPSETLSLTCTVSGFSLTSYIVDWIRQPPGKGLEWIGVIWAG-GSTGYNSALRSRVSITKDTSKNQFSLKLSSVTAADTAVYYCASAAYYSYYNYDGFAYWGQGTLVTVSS

|||||..|..||||.....|.|..||...|||...|..|.||.||||||.|....|||.||....|....|.|.|.......|||.|..|.|||||||.||||||.||||||||||||||||.

QVQLQQPGAELVKPGASVKLSCKASGYTFTSYWMHWVKQRPGQGLEWIGMIHPNSGSTNYNEKFKSKATLTVDKSSSTAYMQLSSLTSEDSAVYYCASEAYYSYYSYDGFAYWGQGTLVTVSA

^^^^^^^^ ^^^^^^^^ ^^^^^^^^^^^^^^^^

Therapeutic : Imgatuzumab

Best Alignment of Imgatuzumab heavy chain to a sequence from OAS

QVQLVQSGAEVKKPGSSVKVSCKASGFTFTDYKIHWVRQAPGQGLEWMGYFNPNSGYSTYAQKFQGRVTITADKSTSTAYMELSSLRSEDTAVYYCARLSPGG----YYVMDAWGQGTTVTVSS

|||||||||||||||.||||||||||.|||.|..|||||||||||||||..|||||...|||||||||||||||||||||||||||||||||||||||.|..|....||.||.|||||||||||

QVQLVQSGAEVKKPGASVKVSCKASGYTFTGYYMHWVRQAPGQGLEWMGWINPNSGGTNYAQKFQGRVTITADKSTSTAYMELSSLRSEDTAVYYCARGSVRGVIDYYYGMDVWGQGTTVTVSS

^^^^^^^^ ^^^^^^^^ ^^^^^^^^^^^^^^^^^

Best Alignment of Imgatuzumab light chain to a sequence from OAS

DIQMTQSPSSLSASVGDRVTITCRASQGINNYLNWYQQKPGKAPKRLIYNTNNLQTGVPSRFSGSGSGTEFTLTISSLQPEDFATYYCLQHN-SFPTFGQGTKLEIK

|||||||||||||||||||||||||||||.|.|.|||||||||||||||.|.|||.||||||||||||||||||||||||||||||||||||.|..|||||||.|||

DIQMTQSPSSLSASVGDRVTITCRASQGIRNGLGWYQQKPGKAPKRLIYATSNLQSGVPSRFSGSGSGTEFTLTISSLQPEDFATYYCLQHNSSPRTFGQGTKVEIK

^^^^^^ ^^^ ^^^^^^^^^

Best Alignment of Imgatuzumab heavy chain CDRs to a sequence from OAS

QVQLVQSGAEVKKPGSSVKVSCKASGFTFTDYKIHWVRQAPGQGLEWMGYFNPNSGYSTYAQKFQGRVTITADKSTSTAYMELSSLRSEDTAVYYCARLSPGGYYVMDAWGQGTTVTVSS

|||||||||||.|||.||||||||||.|||||..|||||||||||||||..|||||...||||||||||.|.|.|.||||||||.|||.|||||||||||..|||.||.||.||||||||

QVQLVQSGAEVQKPGASVKVSCKASGYTFTDYYMHWVRQAPGQGLEWMGWINPNSGGTNYAQKFQGRVTMTRDTSISTAYMELSRLRSDDTAVYYCARLSGDGYYYMDVWGKGTTVTVSS

^^^^^^^^ ^^^^^^^^ ^^^^^^^^^^^^^

Best Alignment of Imgatuzumab light chain CDRs to a sequence from OAS

DIQMTQSPSSLSASVGDRVTITCRASQGINNYLNWYQQKPGKAPKRLIYNTNNLQTGVPSRFSGSGSGTEFTLTISSLQPEDFATYYCLQHNSFPTFGQGTKLEIK

|||||||||||||||||||||||||||||||||||||||.|||||.|.|.||.|..|||||||||||||||||||.||||||||||||.|.||.||||.|||.|||

DIQMTQSPSSLSASVGDRVTITCRASQGINNYLNWYQQKQGKAPKLLNYYTNRLERGVPSRFSGSGSGTEFTLTIGSLQPEDFATYYCQQYNSLPTFGGGTKVEIK

^^^^^^ ^^^ ^^^^^^^^

Best Alignment of Imgatuzumab CDR-H3 to a sequence from OAS

QVQLVQSGAEVKKPGSSVKVSCKASGFTFTDYKIHWVRQAPGQGLEWMGYFNPNSGYSTYAQKFQGRVTITADKSTSTAYMELSSLRSEDTAVYYCARLSPGGYYVMDAWGQGTTVTVSS

.||||.|......||.|.|.||.||||||..|...||||||..||||.....|..|...|.....||.||..|...||.|....||||||||.||.|||||.|||||||||||..|||||

EVQLVESDGGLVQPGRSLKLSCAASGFTFSNYDMAWVRQAPTKGLEWVASISPSGGSTYYRDSVKGRFTISRDNAKSTLYLQMDSLRSEDTATYYGARLSPTGYYVMDAWGQGASVTVSS

^^^^^^^^ ^^^^^^^^ ^^^^^^^^^^^^^

Therapeutic : Inclacumab

Best Alignment of Inclacumab heavy chain to a sequence from OAS

EVQLVESGGGLVRPGGSLRLSCAASGFTFSNYDMHWVRQATGKGLEWVSAITAAGDIYYPGSVKGRFTISRENAKNSLYLQMNSLRAGDTAVYYCARGRYSGSGSYYNDWFDPWGQGTLVTVSS

||||||||||||.|||||||||||||||||.||||||||||||||||||||..|||.|||||||||||||||||||||||||||||||||||||||||.||||...|..|||||||||||||||

EVQLVESGGGLVQPGGSLRLSCAASGFTFSSYDMHWVRQATGKGLEWVSAIGTAGDTYYPGSVKGRFTISRENAKNSLYLQMNSLRAGDTAVYYCARGSYSGS---YRNWFDPWGQGTLVTVSS

^^^^^^^^ ^^^^^^^ ^^^^^^^^^^^^^^^^^^

Best Alignment of Inclacumab light chain to a sequence from OAS

EIVLTQSPATLSLSPGERATLSCRASQSVSSYLAWYQQKPGQAPRLLIYDASNRATGIPARFSGSGSGTDFTLTISSLEPEDFAVYYCQQRSNWPLTFGGGTKVEIK

|||||||||||||||||||||||||||||||||||||||||||||||||||||||||||||||||||||||||||||||||||||||||||||||||||||||||||

EIVLTQSPATLSLSPGERATLSCRASQSVSSYLAWYQQKPGQAPRLLIYDASNRATGIPARFSGSGSGTDFTLTISSLEPEDFAVYYCQQRSNWPLTFGGGTKVEIK

^^^^^^ ^^^ ^^^^^^^^^

Best Alignment of Inclacumab heavy chain CDRs to a sequence from OAS

EVQLVESGGGLVRPGGSLRLSCAASGFTFSNYDMHWVRQATGKGLEWVSAITAAGDIYYPGSVKGRFTISRENAKNSLYLQMNSLRAGDTAVYYCARGRYSGSGSYYNDWFDPWGQGTLVTVSS

..............||||||||||||||||.||||||||||||||||||||..|||.||||||||||||||.|||||||||||||||.|||||||||.||.|||||||..||||||||||||||

--------------GGSLRLSCAASGFTFSSYDMHWVRQATGKGLEWVSAIGTAGDTYYPGSVKGRFTISRDNAKNSLYLQMNSLRAEDTAVYYCARDRYYGSGSYYNVPFDPWGQGTLVTVSS

^^^^^^^^ ^^^^^^^ ^^^^^^^^^^^^^^^^^^

Best Alignment of Inclacumab light chain CDRs to a sequence from OAS

EIVLTQSPATLSLSPGERATLSCRASQSVSSYLAWYQQKPGQAPRLLIYDASNRATGIPARFSGSGSGTDFTLTISSLEPEDFAVYYCQQRSNWPLTFGGGTKVEIK

||||||||||||||||||||||||||||||||||||||||||||||||||||||||||||||||||.||||||||||||||||||||||||||||||||||||||||

EIVLTQSPATLSLSPGERATLSCRASQSVSSYLAWYQQKPGQAPRLLIYDASNRATGIPARFSGSGPGTDFTLTISSLEPEDFAVYYCQQRSNWPLTFGGGTKVEIK

^^^^^^ ^^^ ^^^^^^^^^

Best Alignment of Inclacumab CDR-H3 to a sequence from OAS

EVQLVESGGGLVRPGGSLRLSCAASGFTFSNYDMHWVRQATGKGLEWVSAITAA-GDIYYPGSVKGRFTISRENAKNSLYLQMNSLRAGDTAVYYCARGRYSGSGSYYNDWFDPWGQGTLVTVSS

.............|||||||||||||||||.|.|||||||.|||||||..|.......||..||||||||||.|.||.||||||||||.||||||||||||.|||||||.|||||||||||||||

-------------PGGSLRLSCAASGFTFSSYGMHWVRQAPGKGLEWVAFIRYDGSNKYYADSVKGRFTISRDNSKNTLYLQMNSLRAEDTAVYYCARGRYYGSGSYYNFWFDPWGQGTLVTVSS

^^^^^^^^ ^^^^^^^^ ^^^^^^^^^^^^^^^^^^

Therapeutic : Indatuximab

Best Alignment of Indatuximab heavy chain to a sequence from OAS

QVQLQQSGSELMMPGASVKISCKATGYTFSNYWIEWVKQRPGHGLEWIGEILPGTGRTIYNEKFKGKATFTADISSNTVQMQLSSLTSEDSAVYYCARRDYYGN-FYYAMDYWGQGTSVTVSS

||||||||.|||.|||||||||||||||||.|||||||||||||||||||||||.|.|.||||||||||||||.||||..|||||||||||||||||||.|||...|||||||||||||||||

QVQLQQSGAELMKPGASVKISCKATGYTFSSYWIEWVKQRPGHGLEWIGEILPGSGSTNYNEKFKGKATFTADTSSNTAYMQLSSLTSEDSAVYYCARRGYYGSTHYYAMDYWGQGTSVTVSS

^^^^^^^^ ^^^^^^^^ ^^^^^^^^^^^^^^^^

Best Alignment of Indatuximab light chain to a sequence from OAS

DIQMTQSTSSLSASLGDRVTISCSASQGINNYLNWYQQKPDGTVELLIYYTSTLQSGVPSRFSGSGSGTDYSLTISNLEPEDIGTYYCQQYSKLPRTFGGGTKLEIK

||||||.||||||||||||||||||||||.||||||||||||||.|||||||.||||||||||||||||||||||||||||||.|||||||||||||||||||||||

DIQMTQTTSSLSASLGDRVTISCSASQGISNYLNWYQQKPDGTVKLLIYYTSSLQSGVPSRFSGSGSGTDYSLTISNLEPEDIATYYCQQYSKLPRTFGGGTKLEIK

^^^^^^ ^^^ ^^^^^^^^^

Best Alignment of Indatuximab heavy chain CDRs to a sequence from OAS

QVQLQQSGSELMMPGASVKISCKATGYTFSNYWIEWVKQRPGHGLEWIGEILPGTGRTIYNEKFKGKATFTADISSNTVQMQLSSLTSEDSAVYYCARRDYYGNFYYAMDYWGQGTSVTVSS

.|||||||.|.|.||||||.|||||||||..|||||||||||||||||||||||||.|.||.|||.|||.|.|.||.|..|||.||||||||||||||.|||||||||||||||||||||||

-VQLQQSGDEVMKPGASVKLSCKATGYTFTDYWIEWVKQRPGHGLEWIGEILPGTGSTTYNQKFKAKATLTVDKSSSTAYMQLKSLTSEDSAVYYCARYDYYGNFYYAMDYWGQGTSVTVSS

^^^^^^^^ ^^^^^^^^ ^^^^^^^^^^^^^^^

Best Alignment of Indatuximab light chain CDRs to a sequence from OAS

DIQMTQSTSSLSASLGDRVTISCSASQGINNYLNWYQQKPDGTVELLIYYTSTLQSGVPSRFSGSGSGTDYSLTISNLEPEDIGTYYCQQYSKLPRTFGGGTKLEIK

||||||.|||||.|||||||||||||||||||||||||.|||||.|||||||.|.||||||||||||||||||||||||||||.|||||||||||.|||.|||||||

DIQMTQTTSSLSVSLGDRVTISCSASQGINNYLNWYQQNPDGTVKLLIYYTSSLHSGVPSRFSGSGSGTDYSLTISNLEPEDIATYYCQQYSKLPFTFGSGTKLEIK

^^^^^^ ^^^ ^^^^^^^^^

Best Alignment of Indatuximab CDR-H3 to a sequence from OAS

QVQLQQSGSELMMPGASVKISCKATGYTFSNYWIEWVKQRPGHGLEWIGEILPGTGRTIYNEKFKGKATFTADISSNTVQMQLSSLTSEDSAVYYCARRDYYGNFYYAMDYWGQGTSVTVSS

||||..||.||..||||||.||||.||||..|.|.||.|||..||||||.|.||.|.|.||||||||||.|.|.||.|..||||.|||||||||.|||||||||||||||||||||||||||

QVQLKESGAELVKPGASVKLSCKASGYTFTSYDINWVRQRPEQGLEWIGWIFPGDGSTKYNEKFKGKATLTTDKSSSTAYMQLSRLTSEDSAVYFCARRDYYGNFYYAMDYWGQGTSVTVSS

^^^^^^^^ ^^^^^^^^ ^^^^^^^^^^^^^^^

Therapeutic : Indusatumab

Best Alignment of Indusatumab heavy chain to a sequence from OAS

QVQLQQWGAGLLKPSETLSLTCAVFGGSFSGYYWSWIRQPPGKGLEWIGEINHRGNTNDNPSLKSRVTISVDTSKNQFALKLSSVTAADTAVYYCARERGYT--YGNFDHWGQGTLVTVSS

||||||||||||||||||||||||.||||||||||||||||||||||||||||.|.||.|||||||||||||||||||.|||||||||||||||||||||.|..|.|||.|||||||||||

QVQLQQWGAGLLKPSETLSLTCAVYGGSFSGYYWSWIRQPPGKGLEWIGEINHSGSTNYNPSLKSRVTISVDTSKNQFSLKLSSVTAADTAVYYCARERGITGYYTNFDYWGQGTLVTVSS

^^^^^^^^ ^^^^^^^ ^^^^^^^^^^^^^^^

Best Alignment of Indusatumab light chain to a sequence from OAS

EIVMTQSPATLSVSPGERATLSCRASQSVSRNLAWYQQKPGQAPRLLIYGASTRATGIPARFSGSGSGTEFTLTIGSLQSEDFAVYYCQQYKTWPRTFGQGTNVEIK

||||||||||||||||||||||||||||||.||||||||||||||||||||||||||||||||||||||||||||||||||||||||||||..|||||||||.||||

EIVMTQSPATLSVSPGERATLSCRASQSVSSNLAWYQQKPGQAPRLLIYGASTRATGIPARFSGSGSGTEFTLTIGSLQSEDFAVYYCQQYNNWPRTFGQGTKVEIK

^^^^^^ ^^^ ^^^^^^^^^

Best Alignment of Indusatumab heavy chain CDRs to a sequence from OAS

QVQLQQWGAGLLKPSETLSLTCAVFGGSFSGYYWSWIRQPPGKGLEWIGEINHRGNTNDNPSLKSRVTISVDTSKNQFALKLSSVTAADTAVYYCARERGYTYGNFDHWGQGTLVTVSS

..............||||||||||.||||||||||||||||||||||||||||.|.||.|||||||||.|||||||||.||||||||||||||||||||||.||.||.|||||||||||

--------------SETLSLTCAVYGGSFSGYYWSWIRQPPGKGLEWIGEINHSGSTNYNPSLKSRVTVSVDTSKNQFSLKLSSVTAADTAVYYCARERGYSYGYFDYWGQGTLVTVSS

^^^^^^^^ ^^^^^^^ ^^^^^^^^^^^^^

Best Alignment of Indusatumab light chain CDRs to a sequence from OAS

EIVMTQSPATLSVSPGERATLSCRASQSVSRNLAWYQQKPGQAPRLLIYGASTRATGIPARFSGSGSGTEFTLTIGSLQSEDFAVYYCQQYKTWPRTFGQGTNVEIK

||||||||||||||||||||||||||||||||||||||||||||||||||||.|||||||.|||.||||||||||..|..||.||||.|||||||||.|.||.||.|

EIVMTQSPATLSVSPGERATLSCRASQSVSRNLAWYQQKPGQAPRLLIYGASNRATGIPAGFSGGGSGTEFTLTITRLEPEDLAVYYWQQYKTWPRTLGHGTKVEVK

^^^^^^ ^^^ ^^^^^^^^^

Best Alignment of Indusatumab CDR-H3 to a sequence from OAS

QVQLQQWGAGLLKPSETLSLTCAVFGGSFSGYYWSWIRQPPGKGLEWIGEINHR-GNTNDNPSLKSRVTISVDTSKNQFALKLSSVTAADTAVYYCARERGYTYGNFDHWGQGTLVTVSS

.................|.|.||..|..||.|..||.||.|||||||...|...........|.|.|.|||.|..||...|...|..|.|||||||||||||.|||||.|||||||||||

--------------GGSLRLSCAASGFTFSSYWMSWVRQAPGKGLEWVANIKQDGSEKYYVDSVKGRFTISRDNAKNSLYLQMNSLRAEDTAVYYCARERGYSYGNFDYWGQGTLVTVSS

^^^^^^^^ ^^^^^^^^ ^^^^^^^^^^^^^

Therapeutic : Inebilizumab

Best Alignment of Inebilizumab heavy chain to a sequence from OAS

EVQLVESGGGLVQPGGSLRLSCAASGFTFSSSWMNWVRQAPGKGLEWVGRIYPG--DGDTNYNVKFKGRFTISRDDSKNSLYLQMNSLKTEDTAVYYCARSGFITTVRDFDYWGQGTLVTVSS

||||||||||||||||||||||||||||||..|||||||||||||||||||.....||.|.|....|||||||||||||.||||||||||||||||||||.......|.||||||||||||||

EVQLVESGGGLVQPGGSLRLSCAASGFTFSNAWMNWVRQAPGKGLEWVGRIKSKTDDGTTDYATPVKGRFTISRDDSKNTLYLQMNSLKTEDTAVYYCAREAA----RRFDYWGQGTLVTVSS

^^^^^^^^ ^^^^^^^^^^ ^^^^^^^^^^^^^^

Best Alignment of Inebilizumab light chain to a sequence from OAS

EIVLTQSPDFQSVTPKEKVTITCRASESVDTFGISFMNWFQQKPDQSPKLLIHEASNQGSGVPSRFSGSGSGTDFTLTINSLEAEDAATYYCQQSKEVPFTFGGGTKVEIK

||||||||||||||||||||||||||.|......|..||.||||||||||||..||..|||||||||||||||||||||||||||||||||||||...|.|||||||||||

EIVLTQSPDFQSVTPKEKVTITCRASQSI----GSSLNWYQQKPDQSPKLLIKYASQYGSGVPSRFSGSGSGTDFTLTINSLEAEDAATYYCQQSSSLPITFGGGTKVEIK

^^^^^^^^^^ ^^^ ^^^^^^^^^

Best Alignment of Inebilizumab heavy chain CDRs to a sequence from OAS

EVQLVESGGGLVQPGGSLRLSCAASGFTFSSSWMNWVRQAPGKGLEWVGRIYPGDGDTNYNVKFKGRFTISRDDSKNSLYLQMNSLKTEDTAVYYCARSGFITTVRDFDYWGQGTLVTVSS

.|||.|||..||.||.|...||.|||..|||||||||.|.|||||||.|||||||||||||.||||..|...|.|....|.|..||..||.|||.||||||||||.|||||||||..||||

QVQLKESGPELVKPGASVKISCKASGYAFSSSWMNWVKQRPGKGLEWIGRIYPGDGDTNYNGKFKGKATLTADKSSSTAYMQLSSLTSEDSAVYFCARSGFITTVVDFDYWGQGTTLTVSS

^^^^^^^^ ^^^^^^^^ ^^^^^^^^^^^^^^

Best Alignment of Inebilizumab light chain CDRs to a sequence from OAS

EIVLTQSPDFQSVTPKEKVTITCRASESVDTFGISFMNWFQQKPDQSPKLLIHEASNQGSGVPSRFSGSGSGTDFTLTINSLEAEDAATYYCQQSKEVPFTFGGGTKVEIK

.|||||||....|......||.|||||||||.||||||||||||.|.|||||..|||||||||.|||||||||||.|.|...|..|.|.|.||||||||||||.|||.|||

DIVLTQSPASLAVSLWQRATIPCRASESVDTYGISFMNWFQQKPGQPPKLLIYAASNQGSGVPARFSGSGSGTDFSLNIHPMEEDDTAMYFCQQSKEVPFTFGSGTKLEIK

^^^^^^^^^^ ^^^ ^^^^^^^^^

Best Alignment of Inebilizumab CDR-H3 to a sequence from OAS

EVQLVESGGGLVQPGGSLRLSCAASGFTFSSSWMNWVRQAPGKGLEWVGRIYPGDGDTNYNVKFKGRFTISRDDSKNSLYLQMNSLKTEDTAVYYCARSGFITTVRDFDYWGQGTLVTVSS

.|||...|..||.||.|..|||.|||.||.|.||.||.|.||.||||.|.|.|.|..||||.||||..|...|......|.|..||..||.|||||||.|||||||||||||.||..||||

QVQLQQPGAELVKPGASVKLSCKASGYTFTSYWMQWVKQRPGQGLEWIGEIDPSDSYTNYNQKFKGKATLTGDTASSTAYMQLSSLTSEDSAVYYCARGGFITTVRDFDYWGKGTTLTVSS

^^^^^^^^ ^^^^^^^^ ^^^^^^^^^^^^^^

Therapeutic : Infliximab

Best Alignment of Infliximab heavy chain to a sequence from OAS

EVKLEESGGGLVQPGGSMKLSCVASGFIFSNHWMNWVRQSPEKGLEWVAEIRSKSINSATHYAESVKGRFTISRDDSKSAVYLQMTDLRTEDTGVYYCSRNYYGSTYDYWGQGTTLTVSS

|||||||||||||||||||||||||||.|||.||||||||||||||||||||.||.|.|||||||||||||||||||||.|||||..||.||||||||.|.||||..|||||||||||||

EVKLEESGGGLVQPGGSMKLSCVASGFTFSNYWMNWVRQSPEKGLEWVAEIRLKSNNYATHYAESVKGRFTISRDDSKSSVYLQMNNLRAEDTGVYYCTRHYYGSSFDYWGQGTTLTVSS

^^^^^^^^ ^^^^^^^^^^ ^^^^^^^^^^^

Best Alignment of Infliximab light chain to a sequence from OAS

DILLTQSPAILSVSPGERVSFSCRASQFVGSSIHWYQQRTNGSPRLLIKYASESMSGIPSRFSGSGSGTDFTLSINTVESEDIADYYCQQSHSWPFTFGSGTNLEVK

|||||||||||||||||||||||||||..|.|||||||||||||||||||||||.|||||||||||||||||||||.|||||||||||||||||||||||||.||.|

DILLTQSPAILSVSPGERVSFSCRASQSIGTSIHWYQQRTNGSPRLLIKYASESISGIPSRFSGSGSGTDFTLSINSVESEDIADYYCQQSHSWPFTFGSGTKLEIK

^^^^^^ ^^^ ^^^^^^^^^

Best Alignment of Infliximab heavy chain CDRs to a sequence from OAS

EVKLEESGGGLVQPGGSMKLSCVASGFIFSNHWMNWVRQSPEKGLEWVAEIRSKSINSATHYAESVKGRFTISRDDSKSAVYLQMTDLRTEDTGVYYCSRNYYGSTYDYWGQGTTLTVSS

|||||||||||||||||||||||||||.|||.|||||||||||||||||.||.||.||||.|||||.||||||||||||.|||||..||.||||.|||.|.|||||.|||||||||||||

EVKLEESGGGLVQPGGSMKLSCVASGFTFSNYWMNWVRQSPEKGLEWVAQIRLKSDNSATPYAESVQGRFTISRDDSKSSVYLQMNNLRAEDTGIYYCTRSYYGSTFDYWGQGTTLTVSS

^^^^^^^^ ^^^^^^^^^^ ^^^^^^^^^^^

Best Alignment of Infliximab light chain CDRs to a sequence from OAS

DILLTQSPAILSVSPGERVSFSCRASQFVGSSIHWYQQRTNGSPRLLIKYASESMSGIPSRFSGSGSGTDFTLSINTVESEDIADYYCQQSHSWPFTFGSGTNLEVK

...........|||.||.....|.|.|.||||....||.....|.|||..||....|||.||.||||||.|||.|.....||.|.||||||||||.|||.||..|.|

-----------SVSAGESSTPVCGAGQSVGSSVALFQQKPGQAPSLLIYGASTRATGIPARFGGSGSGTEFTLTISSLQAEDFAVYYCQQSHSWPVTFGQGTRVEIK

^^^^^^ ^^^ ^^^^^^^^^

Best Alignment of Infliximab CDR-H3 to a sequence from OAS

EVKLEESGGGLVQPGGSMKLSCVASGFIFSNHWMNWVRQSPEKGLEWVAEIRSKSINSATHYAESVKGRFTISRDDSKSAVYLQMTDLRTEDTGVYYCSRNYYGSTYDYWGQGTTLTVSS

...|...|..|..||...|.||.|||..|....|.||.|.|.|||.|...|..........||...||||..|...|.|..|||...|..|||..|.||||||||.||||||||||||||

QIQLVQYGPELKKPGETVKISCKASGYTFTTYGMSWVKQAPGKGLKWMGWINTY--AGVPTYADDFKGRFAFSLETSASTAYLQINNLKNEDTATYFCSRNYYGSSYDYWGQGTTLTVSS

^^^^^^^^ ^^^^^^^^^^ ^^^^^^^^^^^

Therapeutic : Inotuzumab

Best Alignment of Inotuzumab heavy chain to a sequence from OAS

EVQLVQSGAEVKKPGASVKVSCKASGYRFTNYWIHWVRQAPGQGLEWIGGINPGNNYATYRRKFQGRVTMTADTSTSTVYMELSSLRSEDTAVYYCTREGYGN------YGAWFAYWGQGTLVTVSS

.||||||||||||||||||||||||||.||.|..|||||||||||||.|.|||.....||..|||||||||.||||||||||||||||||||||||.|||.|.......|.|.|.||||||||||||

QVQLVQSGAEVKKPGASVKVSCKASGYTFTSYYMHWVRQAPGQGLEWMGIINPSGGSTTYAQKFQGRVTMTRDTSTSTVYMELSSLRSEDTAVYYCAREGDGYYDFSGYYYAPFQYWGQGTLVTVSS

^^^^^^^^ ^^^^^^^^ ^^^^^^^^^^^^^^^^^^^^

Best Alignment of Inotuzumab light chain to a sequence from OAS

DVQVTQSPSSLSASVGDRVTITCRSSQSLANSYGNTFLSWYLHKPGKAPQLLIYGISNRFSGVPDRFSGSGSGTDFTLTISSLQPEDFATYYCLQGTHQPYTFGQGTKVEIK

||.|||.|.||..|.||.|.|.||||||||||||||.||||||||||.||||||||||||||||||||||||||||||.||...|||...||||||||||||||.|||.|||

DVVVTQTPLSLPVSFGDQVSISCRSSQSLANSYGNTYLSWYLHKPGKSPQLLIYGISNRFSGVPDRFSGSGSGTDFTLKISTIKPEDLGMYYCLQGTHQPYTFGGGTKLEIK

^^^^^^^^^^^ ^^^ ^^^^^^^^^

Best Alignment of Inotuzumab heavy chain CDRs to a sequence from OAS

EVQLVQSGAEVKKPGASVKVSCKASGYRFTNYWIHWVRQAPGQGLEWIGGINPGNNYATYRRKFQGRVTMTADTSTSTVYMELSSLRSEDTAVYYCTREGYGNYGAWFAYWGQGTLVTVSS

||||.|||.....||||||.|||||||.||.||.|||.|.|||||||||.|.|||....|..||.|....||.|||||.|||||||..||.|||||||||||||||||||||||||||||.

EVQLQQSGTVLARPGASVKMSCKASGYTFTSYWMHWVKQRPGQGLEWIGAIYPGNSDTSYNQKFKGKAKLTAVTSTSTAYMELSSLTNEDSAVYYCTREGYGNYGAWFAYWGQGTLVTVSA

^^^^^^^^ ^^^^^^^^ ^^^^^^^^^^^^^^

Best Alignment of Inotuzumab light chain CDRs to a sequence from OAS

DVQVTQSPSSLSASVGDRVTITCRSSQSLANSYGNTFLSWYLHKPGKAPQLLIYGISNRFSGVPDRFSGSGSGTDFTLTISSLQPEDFATYYCLQGTHQPYTFGQGTKVEIK

||.|||.|.||..|.||.|.|.||||||||||||||||||||||||..||.|||||||||||||||||||||||||||.||...|||...||||||||||.|||.|||.|||

DVVVTQTPLSLPVSFGDQVSISCRSSQSLANSYGNTFLSWYLHKPGQSPQFLIYGISNRFSGVPDRFSGSGSGTDFTLKISTIKPEDLGMYYCLQGTHQPFTFGSGTKLEIK

^^^^^^^^^^^ ^^^ ^^^^^^^^^

Best Alignment of Inotuzumab CDR-H3 to a sequence from OAS

EVQLVQSGAEVKKPGASVKVSCKASGYRFTNYWIHWVRQAPGQGLEWIGGINPGNNYATYRRKFQGRVTMTADTSTSTVYMELSSLRSEDTAVYYCTREGYGNYGAWFAYWGQGTLVTVSS

||||.|||.....||||||.|||||||.||.||.|||.|.|||||||||.|.|||....|..||.|....||.|||||.|||||||..||.|||||||||||||||||||||||||||||.

EVQLQQSGTVLARPGASVKMSCKASGYTFTSYWMHWVKQRPGQGLEWIGAIYPGNSDTSYNQKFKGKAKLTAVTSTSTAYMELSSLTNEDSAVYYCTREGYGNYGAWFAYWGQGTLVTVSA

^^^^^^^^ ^^^^^^^^ ^^^^^^^^^^^^^^

Therapeutic : Intetumumab

Best Alignment of Intetumumab heavy chain to a sequence from OAS

QVQLVESGGGVVQPGRSRRLSCAASGFTFSRYTMHWVRQAPGKGLEWVAVISFDGSNKYYVDSVKGRFTISRDNSENTLYLQVNILRAEDTAVYYCAREARG----SYAFDIWGQGTMVTVSS

|||||||||||||||||.||||||||||||.|.|||||||||||||||||||.|||||||.||||||||||||||.||||||.|.|||||||||||||||||....|.|||||||||||||||

QVQLVESGGGVVQPGRSLRLSCAASGFTFSSYAMHWVRQAPGKGLEWVAVISYDGSNKYYADSVKGRFTISRDNSKNTLYLQMNSLRAEDTAVYYCAREARGYGDYSDAFDIWGQGTMVTVSS

^^^^^^^^ ^^^^^^^^ ^^^^^^^^^^^^^^^^

Best Alignment of Intetumumab light chain to a sequence from OAS

EIVLTQSPATLSLSPGERATLSCRASQSVSSYLAWYQQKPGQAPRLLIYDASNRATGIPARFSGSGSGTDFTLTISSLEPEDFAVYYCQQRSNWPPFTFGPGTKVDIK

||||||||||||||||||||||||||||||||||||||||||||||||||||||||||||||||||||||||||||||||||||||||||||||||||||||||||||

EIVLTQSPATLSLSPGERATLSCRASQSVSSYLAWYQQKPGQAPRLLIYDASNRATGIPARFSGSGSGTDFTLTISSLEPEDFAVYYCQQRSNWPPFTFGPGTKVDIK

^^^^^^ ^^^ ^^^^^^^^^^

Best Alignment of Intetumumab heavy chain CDRs to a sequence from OAS

QVQLVESGGGVVQPGRSRRLSCAASGFTFSRYTMHWVRQAPGKGLEWVAVISFDGSNKYYVDSVKGRFTISRDNSENTLYLQVNILRAEDTAVYYCAREARGSYAFDIWGQGTMVTVSS

..............|.|.||||||||||||.|.|||||||||||||||||||.|||||||.||||||||||||||.||||||.|.||||||||||||||.|||||||||||||||||||

--------------GGSLRLSCAASGFTFSSYAMHWVRQAPGKGLEWVAVISYDGSNKYYADSVKGRFTISRDNSKNTLYLQMNSLRAEDTAVYYCARETRGSYAFDIWGQGTMVTVSS

^^^^^^^^ ^^^^^^^^ ^^^^^^^^^^^^

Best Alignment of Intetumumab light chain CDRs to a sequence from OAS

EIVLTQSPATLSLSPGERATLSCRASQSVSSYLAWYQQKPGQAPRLLIYDASNRATGIPARFSGSGSGTDFTLTISSLEPEDFAVYYCQQRSNWPPFTFGPGTKVDIK

||||||||||||||||||||||||||||||||||||||||||||||||||||||||||||||||||||||||||||||||||||||||||||||||||||.||||.||

EIVLTQSPATLSLSPGERATLSCRASQSVSSYLAWYQQKPGQAPRLLIYDASNRATGIPARFSGSGSGTDFTLTISSLEPEDFAVYYCQQRSNWPPFTFGGGTKVEIK

^^^^^^ ^^^ ^^^^^^^^^^

Best Alignment of Intetumumab CDR-H3 to a sequence from OAS

QVQLVESGGGVVQPGRSRRLSCAASGFTFSRYTMHWVRQAPGKGLEWVAVISFDGSNKYYVDSVKGRFTISRDNSENTLYLQVNILRAEDTAVYYCAREARGSYAFDIWGQGTMVTVSS

................|.||||||||||||.|.|||||||.|||||||..|.......||..||||||||||.|..|.||||.|.|||.|||||||||||||.||||||||||||||||

----------------SLRLSCAASGFTFSSYDMHWVRQATGKGLEWVSAIGTA-GDTYYPGSVKGRFTISRENAKNSLYLQMNSLRAGDTAVYYCAREARGGYAFDIWGQGTMVTVSS

^^^^^^^^ ^^^^^^^^ ^^^^^^^^^^^^

Therapeutic : Ipilimumab

Best Alignment of Ipilimumab heavy chain to a sequence from OAS

QVQLVESGGGVVQPGRSLRLSCAASGFTFSSYTMHWVRQAPGKGLEWVTFISYDGNNKYYADSVKGRFTISRDNSKNTLYLQMNSLRAEDTAIYYCARTGWL--------GPFDYWGQGTLVTVSS

||||||||||||||||||||||||||||||||.||||||||||||||||.|||||.||||||||||||||||||||||||||||||||||||.|||||.|..........||||||||||||||||

QVQLVESGGGVVQPGRSLRLSCAASGFTFSSYAMHWVRQAPGKGLEWVTVISYDGSNKYYADSVKGRFTISRDNSKNTLYLQMNSLRAEDTAVYYCARAGLGDYGDYRRPGPFDYWGQGTLVTVSS

^^^^^^^^ ^^^^^^^^ ^^^^^^^^^^^^^^^^^^^

Best Alignment of Ipilimumab light chain to a sequence from OAS

EIVLTQSPGTLSLSPGERATLSCRASQSVGSSYLAWYQQKPGQAPRLLIYGAFSRATGIPDRFSGSGSGTDFTLTISRLEPEDFAVYYCQQYGSSPWTFGQGTKVEIK

|||||||||||||||||||||||||||||||||||||||||||||||||||||||||||||||||||||||||||||||||||||.||||||||||||||||||||||

EIVLTQSPGTLSLSPGERATLSCRASQSVGSSYLAWYQQKPGQAPRLLIYGAFSRATGIPDRFSGSGSGTDFTLTISRLEPEDFALYYCQQYGSSPWTFGQGTKVEIK

^^^^^^^ ^^^ ^^^^^^^^^

Best Alignment of Ipilimumab heavy chain CDRs to a sequence from OAS

QVQLVESGGGVVQPGRSLRLSCAASGFTFSSYTMHWVRQAPGKGLEWVTFISYDGNNKYYADSVKGRFTISRDNSKNTLYLQMNSLRAEDTAIYYCARTGWLGPFDYWGQGTLVTVSS

..............|.||||||||||||||||.|||||||||||||||..|||||.||||||||||||||||||||||||||||||||||||.||||||.||||||||||||||||||

--------------GGSLRLSCAASGFTFSSYAMHWVRQAPGKGLEWVAVISYDGSNKYYADSVKGRFTISRDNSKNTLYLQMNSLRAEDTAVYYCARTLWLGPFDYWGQGTLVTVSS

^^^^^^^^ ^^^^^^^^ ^^^^^^^^^^^

Best Alignment of Ipilimumab light chain CDRs to a sequence from OAS

EIVLTQSPGTLSLSPGERATLSCRASQSVGSSYLAWYQQKPGQAPRLLIYGAFSRATGIPDRFSGSGSGTDFTLTISRLEPEDFAVYYCQQYGSSPWTFGQGTKVEIK

|||||||||||||||||||||||||||||||||||||||||||||||||||||||||||||||||||||||||||||||||||||.||||||||||||||||||||||

EIVLTQSPGTLSLSPGERATLSCRASQSVGSSYLAWYQQKPGQAPRLLIYGAFSRATGIPDRFSGSGSGTDFTLTISRLEPEDFALYYCQQYGSSPWTFGQGTKVEIK

^^^^^^^ ^^^ ^^^^^^^^^

Best Alignment of Ipilimumab CDR-H3 to a sequence from OAS

QVQLVESGGGVVQPGRSLRLSCAASGFTFSSYTMHWVRQAPGKGLEWVTFISYDGNNKYYADSVKGRFTISRDNSKNTLYLQMNSLRAEDTAIYYCARTGWLGPFDYWGQGTLVTVSS

....................||.|||.|||||...|||||||.||||...|........||....||.||..|.|..|.|....|||.||||.||||||.||||||||||||||||||

------------------KISCKASGGTFSSYAISWVRQAPGQGLEWMGGIIPIFGTANYAQKFQGRVTITADESTSTAYMELSSLRSEDTAVYYCARTQWLGPFDYWGQGTLVTVSS

^^^^^^^^ ^^^^^^^^ ^^^^^^^^^^^

Therapeutic : Iratumumab

Best Alignment of Iratumumab heavy chain to a sequence from OAS

QVQLQQWGAGLLKPSETLSLTCAVYGGSFSAYYWSWIRQPPGKGLEWIGDINHGGGTNYNPSLKSRVTISVDTSKNQFSLKLNSVTAADTAVYYCASL------TAYWGQGSLVTVSS

||||||||||||||||||||||||||||||.||||||||||||||||||.|||.|.||||||||||||||||||||||||||||||||||||||||||.......||||||.||||||

QVQLQQWGAGLLKPSETLSLTCAVYGGSFSGYYWSWIRQPPGKGLEWIGEINHSGSTNYNPSLKSRVTISVDTSKNQFSLKLNSVTAADTAVYYCASLFMIVEDMAYWGQGTLVTVSS

^^^^^^^^ ^^^^^^^ ^^^^^^^^^^^^

Best Alignment of Iratumumab light chain to a sequence from OAS

DIQMTQSPTSLSASVGDRVTITCRASQGISSWLTWYQQKPEKAPKSLIYAASSLQSGVPSRFSGSGSGTDFTLTISSLQPEDFATYYCQQYDSYPITFGQGTRLEIK

||||||||.||||||||||||||||||||||||.|||||||||||||||||||||||||||||||||||||||||||||||||||||||||||||||||||||||||

DIQMTQSPSSLSASVGDRVTITCRASQGISSWLAWYQQKPEKAPKSLIYAASSLQSGVPSRFSGSGSGTDFTLTISSLQPEDFATYYCQQYDSYPITFGQGTRLEIK

^^^^^^ ^^^ ^^^^^^^^^

Best Alignment of Iratumumab heavy chain CDRs to a sequence from OAS

QVQLQQWGAGLLKPSETLSLTCAVYGGSFSAYYWSWIRQPPGKGLEWIGDINHGGGTNYNPSLKSRVTISVDTSKNQFSLKLNSVTAADTAVYYCASLTAYWGQGSLVTVSS

.............|||||||||||||||||.||||||||||||||||||.|||||.||||||||||||||||||||||||||.|||||||||||||.||||||||.||||||

-------------PSETLSLTCAVYGGSFSGYYWSWIRQPPGKGLEWIGEINHGGSTNYNPSLKSRVTISVDTSKNQFSLKLSSVTAADTAVYYCARLTAYWGQGTLVTVSS

^^^^^^^^ ^^^^^^^ ^^^^^^

Best Alignment of Iratumumab light chain CDRs to a sequence from OAS

DIQMTQSPTSLSASVGDRVTITCRASQGISSWLTWYQQKPEKAPKSLIYAASSLQSGVPSRFSGSGSGTDFTLTISSLQPEDFATYYCQQYDSYPITFGQGTRLEIK

|.|.||||.|.||||||||||||||||||||||.|||||||||||||||||||||||||||||||||||||||||||||||||||||||||||||||||||||||||

DSQLTQSPSSPSASVGDRVTITCRASQGISSWLAWYQQKPEKAPKSLIYAASSLQSGVPSRFSGSGSGTDFTLTISSLQPEDFATYYCQQYDSYPITFGQGTRLEIK

^^^^^^ ^^^ ^^^^^^^^^

Best Alignment of Iratumumab CDR-H3 to a sequence from OAS

QVQLQQWGAGLLKPSETLSLTCAVYGGSFSAYYWSWIRQPPGKGLEWIGDINHGGGTNYNPSLKSRVTISVDTSKNQFSLKLNSVTAADTAVYYCASLTAYWGQGSLVTVSS

.............|||||||||.|.|||.|.||||||||||||||||||.|...|.||||||||||||||||||||||||||.||||||||||||||||||||||.||||||

-------------PSETLSLTCTVSGGSISSYYWSWIRQPPGKGLEWIGYIYYSGSTNYNPSLKSRVTISVDTSKNQFSLKLSSVTAADTAVYYCASLTAYWGQGTLVTVSS

^^^^^^^^ ^^^^^^^ ^^^^^^

Therapeutic : Isatuximab

Best Alignment of Isatuximab heavy chain to a sequence from OAS

QVQLVQSGAEVAKPGTSVKLSCKASGYTFTDYWMQWVKQRPGQGLEWIGTIYPGDGDTGYAQKFQGKATLTADKSSKTVYMHLSSLASEDSAVYYCARGDYYG---SNSLDYWGQGTSVTVSS

||||.|||||.|.||.||||||||||||||.||||||||||||||||||.||||||||.|.|||.|||||||||||.|.||.|||||||||||||||||||||...|...|||||||||||||

QVQLQQSGAELARPGASVKLSCKASGYTFTSYWMQWVKQRPGQGLEWIGAIYPGDGDTRYTQKFKGKATLTADKSSSTAYMQLSSLASEDSAVYYCARGDYYGSSYSYAMDYWGQGTSVTVSS

^^^^^^^^ ^^^^^^^^ ^^^^^^^^^^^^^^^^

Best Alignment of Isatuximab light chain to a sequence from OAS

DIVMTQSHLSMSTSLGDPVSITCKASQDVSTVVAWYQQKPGQSPRRLIYSASYRYIGVPDRFTGSGAGTDFTFTISSVQAEDLAVYYCQQHYSPPYTFGGGTKLEIK

||||||||..||||.||.|||||||||||||.||||||||||||.||||||||||.||||||||||.||||||||||||||||||||||||||.|||||||||||||

DIVMTQSHKFMSTSVGDRVSITCKASQDVSTAVAWYQQKPGQSPKRLIYSASYRYTGVPDRFTGSGSGTDFTFTISSVQAEDLAVYYCQQHYSTPYTFGGGTKLEIK

^^^^^^ ^^^ ^^^^^^^^^

Best Alignment of Isatuximab heavy chain CDRs to a sequence from OAS

QVQLVQSGAEVAKPGTSVKLSCKASGYTFTDYWMQWVKQRPGQGLEWIGTIYPGDGDTGYAQKFQGKATLTADKSSKTVYMHLSSLASEDSAVYYCARGDYYGSNSLDYWGQGTSVTVSS

.|||..||||.|.||.||||||||||||||.||||||||||||||||||.||||||||.|.|||.|||||||||||.|.||.|||||||||||||||||||||||..|||||||..||||

-VQLQESGAELARPGASVKLSCKASGYTFTSYWMQWVKQRPGQGLEWIGAIYPGDGDTRYTQKFKGKATLTADKSSSTAYMQLSSLASEDSAVYYCARGDYYGSNYFDYWGQGTTLTVSS

^^^^^^^^ ^^^^^^^^ ^^^^^^^^^^^^^

Best Alignment of Isatuximab light chain CDRs to a sequence from OAS

DIVMTQSHLSMSTSLGDPVSITCKASQDVSTVVAWYQQKPGQSPRRLIYSASYRYIGVPDRFTGSGAGTDFTFTISSVQAEDLAVYYCQQHYSPPYTFGGGTKLEIK

||||||||..||||.||.||||||||||||||||||||||||||..|||||||||.|||.||||||.||||||||||||||||||||||||||.|||||||||||||

DIVMTQSHKFMSTSVGDRVSITCKASQDVSTVVAWYQQKPGQSPKLLIYSASYRYTGVPGRFTGSGSGTDFTFTISSVQAEDLAVYYCQQHYSTPYTFGGGTKLEIK

^^^^^^ ^^^ ^^^^^^^^^

Best Alignment of Isatuximab CDR-H3 to a sequence from OAS

QVQLVQSGAEVAKPGTSVKLSCKASGYTF--TDYWMQWVKQRPGQGLEWIGTIYPGDGDTGYAQKFQGKATLTADKSSKTVYMHLSSLASEDSAVYYCARGDYYGSNSLDYWGQGTSVTVSS

...................|.|..||.......|...|..|.||.||||||.||...|.|.|........|...|.|.......|||....|.|||||||||||||.|||||||||.|||||

----------------TLSLTCTVSGGSISSGGYYWSWIRQHPGKGLEWIGYIYYS-GSTYYNPSLKSRVTISVDTSKNQFSLKLSSVTAADTAVYYCARGDYYGSGSLDYWGQGTLVTVSS

^^^^^^^^^^ ^^^^^^^^ ^^^^^^^^^^^^^

Therapeutic : Itolizumab

Best Alignment of Itolizumab heavy chain to a sequence from OAS

EVQLVESGGGLVKPGGSLKLSCAASGFKFSRYAMSWVRQAPGKRLEWVATISSGGSYIYYPDSVKGRFTISRDNVKNTLYLQMSSLRSEDTAMYYCARRDYD--LDYFDSWGQGTLVTVSS

|||||||||||||||||||||||||||.||.||||||||.|.|||||||||||||||.||||||||||||||||.|||||||||||||||||||||||||||...||||.|||||..||||

EVQLVESGGGLVKPGGSLKLSCAASGFTFSSYAMSWVRQTPEKRLEWVATISSGGSYTYYPDSVKGRFTISRDNAKNTLYLQMSSLRSEDTAMYYCARRDYDGSSDYFDYWGQGTTLTVSS

^^^^^^^^ ^^^^^^^^ ^^^^^^^^^^^^^^

Best Alignment of Itolizumab light chain to a sequence from OAS

DIQMTQSPSSLSASVGDRVTITCKASRDIRSYLTWYQQKPGKAPKTLIYYATSLADGVPSRFSGSGSGQDYSLTISSLESDDTATYYCLQHGESPFTLGSGTKLEIK

||.||||||||.||.|.|||||||||.||.|||.||||||.|.||||||||||||||||||||||||||||||||||||||||||||||||||||||.|||||||||

DIKMTQSPSSLYASLGERVTITCKASQDIKSYLSWYQQKPWKSPKTLIYYATSLADGVPSRFSGSGSGQDYSLTISSLESDDTATYYCLQHGESPFTFGSGTKLEIK

^^^^^^ ^^^ ^^^^^^^^^

Best Alignment of Itolizumab heavy chain CDRs to a sequence from OAS

EVQLVESGGGLVKPGGSLKLSCAASGFKFSRYAMSWVRQAPGKRLEWVATISSGGSYIYYPDSVKGRFTISRDNVKNTLYLQMSSLRSEDTAMYYCARRDYDLDYFDSWGQGTLVTVSS

||||.||||||||||||||||||||||.||.||||||||.|.|||||||||||||||.||||||||||||||||.||||||||||||||||||||||||||||.|||.|||||..||||

EVQLQESGGGLVKPGGSLKLSCAASGFTFSSYAMSWVRQTPEKRLEWVATISSGGSYTYYPDSVKGRFTISRDNAKNTLYLQMSSLRSEDTAMYYCARRDYDLYYFDYWGQGTTLTVSS

^^^^^^^^ ^^^^^^^^ ^^^^^^^^^^^^

Best Alignment of Itolizumab light chain CDRs to a sequence from OAS

DIQMTQSPSSLSASVGDRVTITCKASRDIRSYLTWYQQKPGKAPKTLIYYATSLADGVPSRFSGSGSGQDYSLTISSLESDDTATYYCLQHGESPFTLGSGTKLEIK

||.|||||||..||.|.||||||||||||.|||.||||||.|.||||||||||||||||||||||||||||||||||||||||||||||||||||.|.|.|||||||

DIKMTQSPSSMYASLGERVTITCKASRDIKSYLSWYQQKPWKSPKTLIYYATSLADGVPSRFSGSGSGQDYSLTISSLESDDTATYYCLQHGESPPTFGGGTKLEIK

^^^^^^ ^^^ ^^^^^^^^^

Best Alignment of Itolizumab CDR-H3 to a sequence from OAS

EVQLVESGGGLVKPGGSLKLSCAASGFKF-SRYAMSWVRQAPGKRLEWVATISSGGSYIYYPDSVKGRFTISRDNVKNTLYLQMSSLRSEDTAMYYCARRDYDLDYFDSWGQGTLVTVSS

||||||||.|||||..||.|.|........|.||..|.||.||..|||...||.......|..|.|.|..|.||..||...||..|...||||.|||||||||.||||.|||||..||||

EVQLVESGPGLVKPSQSLSLTCTVTSYSITSDYAWNWIRQFPGNKLEWMGYISYS-GSTSYNPSLKSRISITRDTSKNQFFLQLNSVTTEDTATYYCARRDYDYDYFDYWGQGTTLTVSS

^^^^^^^^^ ^^^^^^^^ ^^^^^^^^^^^^

Therapeutic : Ixekizumab

Best Alignment of Ixekizumab heavy chain to a sequence from OAS

QVQLVQSGAEVKKPGSSVKVSCKASGYSFTDYHIHWVRQAPGQGLEWMGVINPMYGTTDYNQRFKGRVTITADESTSTAYMELSSLRSEDTAVYYCARYDYF-----TGTGVYWGQGTLVTVSS

|||||||||||||||.|||||||||||.||.|.||||||||||||||||.|||..|||.|.|.|.|||||||||||||||||||||||||||||||||..||......|...||||||||||||

QVQLVQSGAEVKKPGASVKVSCKASGYTFTGYYIHWVRQAPGQGLEWMGWINPIFGTTNYAQKFQGRVTITADESTSTAYMELSSLRSEDTAVYYCARDQYFGSGSLIGRFDYWGQGTLVTVSS

^^^^^^^^ ^^^^^^^^ ^^^^^^^^^^^^^^^^^

Best Alignment of Ixekizumab light chain to a sequence from OAS

DIVMTQTPLSLSVTPGQPASISCRSSRSLVHSRGNTYLHWYLQKPGQSPQLLIYKVSNRFIGVPDRFSGSGSGTDFTLKISRVEAEDVGVYYCSQSTHLPFTFGQGTKLEIK

|||||||||||||||||||||.||||.|||||.|.|||.|||||||||||||||.||.||.||||||||||||||||||||||||||||||||.|.||||.|||||||||||

DIVMTQTPLSLSVTPGQPASIFCRSSQSLVHSDGSTYLYWYLQKPGQSPQLLIYEVSKRFSGVPDRFSGSGSGTDFTLKISRVEAEDVGVYYCMQGTHLPDTFGQGTKLEIK

^^^^^^^^^^^ ^^^ ^^^^^^^^^

Best Alignment of Ixekizumab heavy chain CDRs to a sequence from OAS

QVQLVQSGAEVKKPGSSVKVSCKASGYSFTDYHIHWVRQAPGQGLEWMGVINPMYGTTDYNQRFKGRVTITADESTSTAYMELSSLRSEDTAVYYCARYDYFTGTGVYWGQGTLVTVSS

.|||.|||.|..|||.|||.||||||||||||...||.|..|..|||.|||||.||||.|||.|||..|.|.|.|.|||||.|.||.|||.||||||||||..||..||||||..||||

EVQLQQSGPELVKPGASVKISCKASGYSFTDYNMNWVKQSNGKSLEWIGVINPNYGTTSYNQKFKGKATLTVDQSSSTAYMQLNSLTSEDSAVYYCARYDYDRGTFDYWGQGTTLTVSS

^^^^^^^^ ^^^^^^^^ ^^^^^^^^^^^^

Best Alignment of Ixekizumab light chain CDRs to a sequence from OAS

DIVMTQTPLSLSVTPGQPASISCRSSRSLVHSRGNTYLHWYLQKPGQSPQLLIYKVSNRFIGVPDRFSGSGSGTDFTLKISRVEAEDVGVYYCSQSTHLPFTFGQGTKLEIK

|||.||..|||.|..|..||||||||.|||||.||||||||||||||||.||||||||||.||||||||||||||||||||||||||.|||.||||||||||||.|||||||

DIVLTQSTLSLPVSLGDQASISCRSSQSLVHSNGNTYLHWYLQKPGQSPKLLIYKVSNRFSGVPDRFSGSGSGTDFTLKISRVEAEDLGVYFCSQSTHLPFTFGSGTKLEIK

^^^^^^^^^^^ ^^^ ^^^^^^^^^

Best Alignment of Ixekizumab CDR-H3 to a sequence from OAS

QVQLVQSGAEVKKPGSSVKVSCKASGYSFTDYHIHWVRQAPGQGLEWMGVINPMYGTTDYNQRFKGRVTITADESTSTAYMELSSLRSEDTAVYYCARYDYFTGTGVYWGQGTLVTVSS

.....||||||||||.|.|.|||.||||||.|.|.||||.||.||||||.|.|....|.|...|.|.|||.||.|.||||...|||...|||.||||||||..|.||||||||||||||

-----QSGAEVKKPGESLKISCKGSGYSFTNYWIAWVRQMPGKGLEWMGIIYPGDSDTRYSPSFQGQVTISADKSISTAYLQWSSLKASDTAMYYCARYDYDSGGGVYWGQGTLVTVSS

^^^^^^^^ ^^^^^^^^ ^^^^^^^^^^^^

Therapeutic : Lacnotuzumab

Best Alignment of Lacnotuzumab heavy chain to a sequence from OAS

QVQLQESGPGLVKPSQTLSLTCTVSDYSITSDYAWNWIRQFPGKGLEWMGYISYSGSTSYNPSLKSRITISRDTSKNQFSLQLNSVTAADTAVYYCASFDYA-HAMDYWGQGTTVTVSS

.|||||||||||||||.|||||||..|||||||||||||||||..|||||||||||||||||||||||.|.||||||||.|||||||..|||.|||||.|||..|||||||||.|||||

DVQLQESGPGLVKPSQSLSLTCTVTGYSITSDYAWNWIRQFPGNKLEWMGYISYSGSTSYNPSLKSRISITRDTSKNQFFLQLNSVTTEDTATYYCASYDYAYYAMDYWGQGTSVTVSS

^^^^^^^^^ ^^^^^^^ ^^^^^^^^^^^^

Best Alignment of Lacnotuzumab light chain to a sequence from OAS

DIVLTQSPAFLSVTPGEKVTFTCQASQSIGTSIHWYQQKTDQAPKLLIKYASESISGIPSRFSGSGSGTDFTLTISSVEAEDAADYYCQQINSWPTTFGGGTKLEIK

||.||||||.|||.|||.|.|.|.||||||||||||||.|...|.||||||||||||||||||||||||||||||.|||.||.|||||||.||||||||||||||||

DILLTQSPAILSVSPGERVSFSCRASQSIGTSIHWYQQRTNDSPRLLIKYASESISGIPSRFSGSGSGTDFTLTINSVESEDIADYYCQQSNSWPTTFGGGTKLEIK

^^^^^^ ^^^ ^^^^^^^^^

Best Alignment of Lacnotuzumab heavy chain CDRs to a sequence from OAS

QVQLQESGPGLVKPSQTLSLTCTVSDYSITSDYAWNWIRQFPGKGLEWMGYISYSGSTSYNPSLKSRITISRDTSKNQFSLQLNSVTAADTAVYYCASFDYAHAMDYWGQGTTVTVSS

.|.|||||||||||||.|||||||..|||||||||||||||||..|||||||||||||||||||||||.|.||||||||.|||||||..|||.|||||.|||.|||||||||.|||||

EVKLQESGPGLVKPSQSLSLTCTVTGYSITSDYAWNWIRQFPGNKLEWMGYISYSGSTSYNPSLKSRISITRDTSKNQFFLQLNSVTTEDTATYYCASYDYAYAMDYWGQGTSVTVSS

^^^^^^^^^ ^^^^^^^ ^^^^^^^^^^^

Best Alignment of Lacnotuzumab light chain CDRs to a sequence from OAS

DIVLTQSPAFLSVTPGEKVTFTCQASQSIGTSIHWYQQKTDQAPKLLIKYASESISGIPSRFSGSGSGTDFTLTISSVEAEDAADYYCQQINSWPTTFGGGTKLEIK

||.||||||.|||.|||.|.|.|.||||||||||||||.|...|.||||||||||||||||||||||||||||.|.|||.||.|.|||||.||||||||.|||||||

DILLTQSPAILSVSPGERVSFSCRASQSIGTSIHWYQQRTNGSPRLLIKYASESISGIPSRFSGSGSGTDFTLSINSVESEDIAEYYCQQSNSWPTTFGSGTKLEIK

^^^^^^ ^^^ ^^^^^^^^^

Best Alignment of Lacnotuzumab CDR-H3 to a sequence from OAS

QVQLQESGPGLVKPSQTLSLTCTVSDYSITSDYAWNWIRQFPGKGLEWMGYISYS-GSTSYNPSLKSRITISRDTSKNQFSLQLNSVTAADTAVYYCASFDYAHAMDYWGQGTTVTVSS

|||||..|..||||.......|..|.|.....|...|..|.||.||||.|.|....|||.||...||..|...|||......||.|.|..|.|||||||||||.|||||||||.|||||

QVQLQQPGAELVKPGASVKMSCKASGYTF-TSYWITWVKQRPGQGLEWIGDIYPGSGSTNYNEKFKSKATLTVDTSSSTAYNQLSSLTTGDSAVYYCASFDYAYAMDYWGQGTSVTVSS

^^^^^^^^^ ^^^^^^^^ ^^^^^^^^^^^

Therapeutic : Lampalizumab

Best Alignment of Lampalizumab heavy chain to a sequence from OAS

EVQLVQSGPELKKPGASVKVSCKASGYTFTNYGMNWVRQAPGQGLEWMGWINTYTGETTYADDFKGRFVFSLDTSVSTAYLQISSLKAEDTAVYYCEREG---GVNNWGQGTLVTVSS

.|||||||.|||||||||||||||||||||.|.||||||||||||||||||||.||.||||..|.|||||||||||||||||||||||||||||||.|||....|..|||||||||||

QVQLVQSGSELKKPGASVKVSCKASGYTFTSYAMNWVRQAPGQGLEWMGWINTNTGNTTYAQGFTGRFVFSLDTSVSTAYLQISSLKAEDTAVYYCAREGGFRSVDYWGQGTLVTVSS

^^^^^^^^ ^^^^^^^^ ^^^^^^^^^^^

Best Alignment of Lampalizumab light chain to a sequence from OAS

DIQVTQSPSSLSASVGDRVTITCITSTDIDDDMNWYQQKPGKVPKLLISGGNTLRPGVPSRFSGSGSGTDFTLTISSLQPEDVATYYCLQSDSLPYTFGQGTKVEIK

|||.|||||||||||||||||||..|.||.....||||||||||||||.|..||..||||||||||||||||||||||||||||||||.|.|.||||||||||.|||

DIQMTQSPSSLSASVGDRVTITCRASQDISNYLAWYQQKPGKVPKLLIYGASTLQSGVPSRFSGSGSGTDFTLTISSLQPEDVATYYCQQYDNLPYTFGQGTKLEIK

^^^^^^ ^^^ ^^^^^^^^^

Best Alignment of Lampalizumab heavy chain CDRs to a sequence from OAS

EVQLVQSGPELKKPGASVKVSCKASGYTFTNYGMNWVRQAPGQGLEWMGWINTYTGETTYADDFKGRFVFSLDTSVSTAYLQISSLKAEDTAVYYCEREGGVNNWGQGTLVTVSS

..|||||||||||||..||.|||||||||||||||||.||||.||.|||||||||||.||||||||||.|||.||.|||||||..||.||||.|...||||...|||||..||||

QIQLVQSGPELKKPGETVKISCKASGYTFTNYGMNWVKQAPGKGLKWMGWINTYTGEPTYADDFKGRFAFSLETSASTAYLQINNLKNEDTATYFWAREGGTDYWGQGTTLTVSS

^^^^^^^^ ^^^^^^^^ ^^^^^^^^

Best Alignment of Lampalizumab light chain CDRs to a sequence from OAS

DIQVTQSPSSLSASVGDRVTITCITSTDIDDDMNWYQQKPGKVPKLLISGGNTLRPGVPSRFSGSGSGTDFTLTISSLQPEDVATYYCLQSDSLPYTFGQGTKVEIK

...|||||.|||...|..|||.|||.|||||||||||||||..||||||||||||||||||||.||.||||..||.....||||.||||||||.|||||.|||.|||

ETTVTQSPASLSVATGEKVTIRCITGTDIDDDMNWYQQKPGEPPKLLISGGNTLRPGVPSRFSSSGYGTDFVFTIDNTLSEDVADYYCLQSDSMPYTFGGGTKLEIK

^^^^^^ ^^^ ^^^^^^^^^

Best Alignment of Lampalizumab CDR-H3 to a sequence from OAS

EVQLVQSGPELKKPGASVKVSCKASGYTFTNYGMNWVRQAPGQGLEWMGWINTYTGETTYADDFKGRFVFSLDTSVSTAYLQISSLKAEDTAVYYCEREGGVNNWGQGTLVTVSS

.....................|...|..|..|...|.||.||.||||.|.||...|.|......|.|...|.|||.....|..||..|.|||||||.||||||.||.||.|||||

----------------TLSLTCAVYGGSFSGYYWSWIRQPPGKGLEWIGEINHS-GSTNSKPSLKSRVTISVDTSKNQFSLILSSVTAADTAVYYCAREGGVNVWGKGTTVTVSS

^^^^^^^^ ^^^^^^^^ ^^^^^^^^

Therapeutic : Lanadelumab

Best Alignment of Lanadelumab heavy chain to a sequence from OAS

EVQLLESGGGLVQPGGSLRLSCAASGFTFSHYIMMWVRQAPGKGLEWVSGIYSSGGITVYADSVKGRFTISRDNSKNTLYLQMNSLRAEDTAVYYCAYRRIGVP-----RRDEFDIWGQGTMVTVSS

||||||||||||||||||||||||||||||.|.|.||||||||||||||||..|||.|.||||||||||||||||||||||||||||||||||||||...|||.......|..||||||||||||||

EVQLLESGGGLVQPGGSLRLSCAASGFTFSSYAMSWVRQAPGKGLEWVSGISGSGGSTYYADSVKGRFTISRDNSKNTLYLQMNSLRAEDTAVYYCAKDSIGVITFGGVNRGSFDIWGQGTMVTVSS

^^^^^^^^ ^^^^^^^^ ^^^^^^^^^^^^^^^^^^^^

Best Alignment of Lanadelumab light chain to a sequence from OAS

DIQMTQSPSTLSASVGDRVTITCRASQSISSWLAWYQQKPGKAPKLLIYKASTLESGVPSRFSGSGSGTEFTLTISSLQPDDFATYYCQQYNTYWTFGQGTKVEIK

||||||||||||||||||||||||||||||||||||||||||||||||||||||||||||||||||||||||||||||||||||||||||||||||||||||||||

DIQMTQSPSTLSASVGDRVTITCRASQSISSWLAWYQQKPGKAPKLLIYKASTLESGVPSRFSGSGSGTEFTLTISSLQPDDFATYYCQQYNTYWTFGQGTKVEIK

^^^^^^ ^^^ ^^^^^^^^

Best Alignment of Lanadelumab heavy chain CDRs to a sequence from OAS

EVQLLESGGGLVQPGGSLRLSCAASGFTFSHYIMMWVRQAPGKGLEWVSGIYSSGGITVYADSVKGRFTISRDNSKNTLYLQMNSLRAEDTAVYYCAYRRIGVPRRDEFDIWGQGTMVTVSS

................||||||||||||||.|.|.||||||||||||||.|..|||.|.||||||||||||||||||||||||||||||||||||||.||||...||.||||||||||||||

----------------SLRLSCAASGFTFSSYAMSWVRQAPGKGLEWVSVISGSGGTTYYADSVKGRFTISRDNSKNTLYLQMNSLRAEDTAVYYCAKRRIGGVVRDAFDIWGQGTMVTVSS

^^^^^^^^ ^^^^^^^^ ^^^^^^^^^^^^^^^

Best Alignment of Lanadelumab light chain CDRs to a sequence from OAS

DIQMTQSPSTLSASVGDRVTITCRASQSISSWLAWYQQKPGKAPKLLIYKASTLESGVPSRFSGSGSGTEFTLTISSLQPDDFATYYCQQYNTYWTFGQGTKVEIK

|||||||||||||||||||||.||||||||||||||||||||||||||||||.|||||||||||||||||||||||||||||.|||||||||||||||||||||||

DIQMTQSPSTLSASVGDRVTIACRASQSISSWLAWYQQKPGKAPKLLIYKASSLESGVPSRFSGSGSGTEFTLTISSLQPDDLATYYCQQYNTYWTFGQGTKVEIK

^^^^^^ ^^^ ^^^^^^^^

Best Alignment of Lanadelumab CDR-H3 to a sequence from OAS

EVQLLESGGGLVQPGGSLRLSCAASGFTFSHYIMMWVRQAPGKGLEWVSGIYSSGGITVYADSVKGRFTISRDNSKNTLYLQMNSLRAEDTAVYYCAYRRIGVPRRDEFDIWGQGTMVTVSS

.............|||||||||||||||||.|.|.|||||||||||||..|...|....|.|||||||||||||.||.|||||||||||||||||||..||||||.|.||||||||||||||

------------RPGGSLRLSCAASGFTFSSYWMSWVRQAPGKGLEWVANIKQDGSEKYYVDSVKGRFTISRDNAKNSLYLQMNSLRAEDTAVYYCARVRIGVPRWDAFDIWGQGTMVTVSS

^^^^^^^^ ^^^^^^^^ ^^^^^^^^^^^^^^^

Therapeutic : Landogrozumab

Best Alignment of Landogrozumab heavy chain to a sequence from OAS

EVQLVESGGGLVQPGGSLRLSCAASGLTFSRYPMSWVRQAPGKGLVWVSAITSSGGSTYYSDTVKGRFTISRDNAKNTLYLQMNSLRAEDTAVYYCARL---------------PDYWGQGTLVTVSS

||||||||||||||||||||||||||.|||.|.||||||||||||.|||||..|||||||.|.|||||||||||.||||||||||||||||||||||||...............||||||||||||||

EVQLVESGGGLVQPGGSLRLSCAASGFTFSSYAMSWVRQAPGKGLEWVSAISGSGGSTYYADSVKGRFTISRDNSKNTLYLQMNSLRAEDTAVYYCARLEFFIAAAGTLVPPSVPDYWGQGTLVTVSS

^^^^^^^^ ^^^^^^^^ ^^^^^^^^^^^^^^^^^^^^^

Best Alignment of Landogrozumab light chain to a sequence from OAS

EIVLTQSPGTLSLSPGERATLSCRASSSVSSSYLHWYQQKPGQAPRLLIYSTSNLVAGIPDRFSGSGSGTDFTLTISRLEPEDFAVYYCQHHSGYHFTFGGGTKVEIK

||||||||||||||||||||||||||.|||||||.|||||||||||||||.|||..||||||||||||||||||||||||||||||||||...|...|||||||||||

EIVLTQSPGTLSLSPGERATLSCRASQSVSSSYLAWYQQKPGQAPRLLIYGTSNRAAGIPDRFSGSGSGTDFTLTISRLEPEDFAVYYCQQYDGSPLTFGGGTKVEIK

^^^^^^^ ^^^ ^^^^^^^^^

Best Alignment of Landogrozumab heavy chain CDRs to a sequence from OAS

EVQLVESGGGLVQPGGSLRLSCAASGLTFSRYPMSWVRQAPGKGLVWVSAITSSGGSTYYSDTVKGRFTISRDNAKNTLYLQMNSLRAEDTAVYYCARLPDYWGQGTLVTVSS

|||||||||.|||||.||.|||.|||.||..|.|.|.|..|||||.||..||||||||.|.|.||||.||||||||.||||||||||.||.|.|||||||||||||..|||||

EVQLVESGGDLVQPGRSLKLSCVASGFTFNNYWMTWIRHVPGKGLEWVASITSSGGSTNYRDSVKGRLTISRDNAKSTLYLQMNSLRSEDMATYYCARLPDYWGQGVMVTVSS

^^^^^^^^ ^^^^^^^^ ^^^^^^

Best Alignment of Landogrozumab light chain CDRs to a sequence from OAS

EIVLTQSPGTLSLSPGERATLSCRASSSVSSSYLHWYQQKPGQAPRLLIYSTSNLVAGIPDRFSGSGSGTDFTLTISRLEPEDFAVYYCQHHSGYHFTFGGGTKVEIK

|.||||||...|.|||...|..||||||||||||||||||.|..|.|.|||||||..|.|.|||||||||...||||..|.||.|.||||..||||||||.|||.|||

ENVLTQSPAIMSASPGDKVTMTCRASSSVSSSYLHWYQQKSGASPKLWIYSTSNLASGVPARFSGSGSGTSYSLTISSVEAEDAATYYCQQYSGYHFTFGSGTKLEIK

^^^^^^^ ^^^ ^^^^^^^^^

Best Alignment of Landogrozumab CDR-H3 to a sequence from OAS

EVQLVESGGGLVQPGGSLRLSCAASGLTFSRYPMSWVRQAPGKGLVWVSAITSSGGSTYYSDTVKGRFTISRDNAKNTLYLQMNSLRAEDTAVYYCARLPDYWGQGTLVTVSS

..............|.||..||.|||.||..|...|||||.|.||.|........|.|.|.....||.|..|.....|.|....|||.|||||||||||||||||||||||||

--------------GESLKISCKASGYTFTSYDINWVRQATGQGLEWMGWMNPNSGNTGYAQKFQGRVTMTRNTSISTAYMELSSLRSEDTAVYYCARLPDYWGQGTLVTVSS

^^^^^^^^ ^^^^^^^^ ^^^^^^

Therapeutic : Lebrikizumab

Best Alignment of Lebrikizumab heavy chain to a sequence from OAS

QVTLRESGPALVKPTQTLTLTCTVSGFSL--SAYSVNWIRQPPGKALEWLAMIWGDGKIVYNSALKSRLTISKDTSKNQVVLTMTNMDPVDTATYYCAGDGYY----PYAMDNWGQGSLVTVSS

|||||||||||||||||||||||.|||||..|...|.||||||||||||||.|..||...||..||.|||||||||||||||||||||||||||||||...||....|.|.|.||||..|||||

QVTLRESGPALVKPTQTLTLTCTFSGFSLSTSGMCVSWIRQPPGKALEWLARIDWDGDKYYNTSLKTRLTISKDTSKNQVVLTMTNMDPVDTATYYCARIHYYDSSGPRAFDIWGQGTMVTVSS

^^^^^^^^^^ ^^^^^^^ ^^^^^^^^^^^^^^^^

Best Alignment of Lebrikizumab light chain to a sequence from OAS

DIVMTQSPDSLSVSLGERATINCRASKSVDSYGNSFMHWYQQKPGQPPKLLIYLASNLESGVPDRFSGSGSGTDFTLTISSLQAEDVAVYYCQQNNEDPRTFGGGTKVEIK

|||.||||.||.||||.||||.||||.||||||||||||||||||||||||||.|||||||||.|||||||.||||||||...|.|||.|||||.||||||||||||.|||

DIVLTQSPASLAVSLGQRATISCRASESVDSYGNSFMHWYQQKPGQPPKLLIYRASNLESGVPARFSGSGSRTDFTLTISPVEADDVATYYCQQSNEDPRTFGGGTKLEIK

^^^^^^^^^^ ^^^ ^^^^^^^^^

Best Alignment of Lebrikizumab heavy chain CDRs to a sequence from OAS

QVTLRESGPALVKPTQTLTLTCTVSGFSLSAYSVNWIRQPPGKALEWLAMIWGDGKIVYNSALKSRLTISKDTSKNQVVLTMTNMDPVDTATYYCAGDGYYPYAMDNWGQGSLVTVSS

||.|.||||.||.|.|.|..|||||||||..|.|.|.||||||.||||..|||||...|.|||.|||.||||.||.||.|........||||||||.|||||||||.||||..|||||

QVQLKESGPGLVAPSQSLSITCTVSGFSLTSYGVSWVRQPPGKGLEWLGVIWGDGSTNYHSALISRLSISKDNSKSQVFLKLNSLQTDDTATYYCAKDGYYPYAMDYWGQGTSVTVSS

^^^^^^^^ ^^^^^^^ ^^^^^^^^^^^^

Best Alignment of Lebrikizumab light chain CDRs to a sequence from OAS

DIVMTQSPDSLSVSLGERATINCRASKSVDSYGNSFMHWYQQKPGQPPKLLIYLASNLESGVPDRFSGSGSGTDFTLTISSLQAEDVAVYYCQQNNEDPRTFGGGTKVEIK

.||.||||.||.||||.||||.||||.||||||||||||||||||||||||||||||||||||.|||||||.|||||||....|.|.|.||||||||||||||||||.|||

NIVPTQSPASLAVSLGQRATISCRASESVDSYGNSFMHWYQQKPGQPPKLLIYLASNLESGVPARFSGSGSRTDFTLTIDPVEADDAATYYCQQNNEDPRTFGGGTKLEIK

^^^^^^^^^^ ^^^ ^^^^^^^^^

Best Alignment of Lebrikizumab CDR-H3 to a sequence from OAS

QVTLRESGPALVKPTQTLTLTCTVSGFSLSAYSVNWIRQPPGKALEWLAMIWGD---GKIVYNSALKSRLTISKDTSKNQVVLTMTNMDPVDTATYYCAGDGYYPYAMDNWGQGSLVTVSS

.|.|.|||..||.|...|.|.|..|||....|...|.|||||||||||..|..........|....|.|...|.|||.......|......|.|.||.|||||||||||.||||..|||||

EVELVESGGDLVQPGGSLRLSCATSGFTFTDYYMSWVRQPPGKALEWLGVIRNKANGYTTEYSASVKGRFPCSRDTSQSILYRQMDTLRAEDGASYYRAGDGYYPYAMDYWGQGASVTVSS

^^^^^^^^ ^^^^^^^^^^ ^^^^^^^^^^^^

Therapeutic : Lenzilumab

Best Alignment of Lenzilumab heavy chain to a sequence from OAS

QVQLVQSGAEVKKPGASVKVSCKASGYSFTNYYIHWVRQAPGQRLEWMGWINAGNGNTKYSQKFQGRVTITRDTSASTAYMELSSLRSEDTAVYYCVRRQRF--------PYYFDYWGQGTLVTVSS

|||||||||||||||||||||||||||.||.||.||||||||||||||||||||||||||||||||||||||||||||||||||||||||||||||.|.||..........||||||||||||||||

QVQLVQSGAEVKKPGASVKVSCKASGYTFTGYYMHWVRQAPGQRLEWMGWINAGNGNTKYSQKFQGRVTITRDTSASTAYMELSSLRSEDTAVYYCARDQRKGTAAGSPIAYYFDYWGQGTLVTVSS

^^^^^^^^ ^^^^^^^^ ^^^^^^^^^^^^^^^^^^^^

Best Alignment of Lenzilumab light chain to a sequence from OAS

EIVLTQSPATLSVSPGERATLSCRASQSVGTNVAWYQQKPGQAPRVLIYSTSSRATGITDRFSGSGSGTDFTLTISRLEPEDFAVYYCQQFNKSPLTFGGGTKVEIK

||||||||||||||||||||||||||||||.|.||||||||||||.|||..|||||||.|||||||||||||||||||||||||||||||...||||||||||||||

EIVLTQSPATLSVSPGERATLSCRASQSVGNNLAWYQQKPGQAPRLLIYGASSRATGIPDRFSGSGSGTDFTLTISRLEPEDFAVYYCQQYSSSPLTFGGGTKVEIK

^^^^^^ ^^^ ^^^^^^^^^

Best Alignment of Lenzilumab heavy chain CDRs to a sequence from OAS

QVQLVQSGAEVKKPGASVKVSCKASGYSFTNYYIHWVRQAPGQRLEWMGWINAGNGNTKYSQKFQGRVTITRDTSASTAYMELSSLRSEDTAVYYCVRRQRFPYYFDYWGQGTLVTVSS

................|||||||||||.||||..|||||||||.||||||||||||||||||||||||||||||||.|||||||||||||||||||.|...||||||||||||||||||

----------------SVKVSCKASGYTFTNYAMHWVRQAPGQWLEWMGWINAGNGNTKYSQKFQGRVTITRDTSARTAYMELSSLRSEDTAVYYCARGFDFPYYFDYWGQGTLVTVSS

^^^^^^^^ ^^^^^^^^ ^^^^^^^^^^^^

Best Alignment of Lenzilumab light chain CDRs to a sequence from OAS

EIVLTQSPATLSVSPGERATLSCRASQSVGTNVAWYQQKPGQAPRVLIYSTSSRATGITDRFSGSGSGTDFTLTISRLEPEDFAVYYCQQFNKSPLTFGGGTKVEIK

|||||||||||||..|||.||||||||||.||.||||||||||||.|.||.|.|||||..|||||||||.||||||.|..||.||||||||||.|||||||||||||

EIVLTQSPATLSVAEGERTTLSCRASQSVNTNLAWYQQKPGQAPRLLLYSASTRATGIPARFSGSGSGTEFTLTISSLQSEDVAVYYCQQFNKWPLTFGGGTKVEIK

^^^^^^ ^^^ ^^^^^^^^^

Best Alignment of Lenzilumab CDR-H3 to a sequence from OAS

QVQLVQSGAEVKKPGASVKVSCKASGYSFTNYYIHWVRQAPGQRLEWMGWINAGNGNTKYSQKFQGRVTITRDTSASTAYMELSSLRSEDTAVYYCVRRQRFPYYFDYWGQGTLVTVSS

.....................|..||.|.......|.||.||..|||.|.|....|...|.......|||..|||........||....||||||||||.|.|||||||||||||||||

----------------TLSLTCTVSGVSISDHSWGWIRQPPGKGLEWIGYISYS-GRSNYNPPLKSQVTISLDTSKNQVSLKVSSVTAADTAVYYCVRRFRDPYYFDYWGQGTLVTVSS

^^^^^^^^ ^^^^^^^^ ^^^^^^^^^^^^

Therapeutic : Lifastuzumab

Best Alignment of Lifastuzumab heavy chain to a sequence from OAS

EVQLVESGGGLVQPGGSLRLSCAASGFSFSDFAMSWVRQAPGKGLEWVATIGRVAFHTYYPDSMKGRFTISRDNSKNTLYLQMNSLRAEDTAVYYCARHRGFD---VGHFDFWGQGTLVTVSS

|||||||||||||||||||||||||||.||..||||||||||||||||..|......|||.||.||||||||||||||||||||||||||||||||||.||.|....|.||.|||||||||||

EVQLVESGGGLVQPGGSLRLSCAASGFTFSSYAMSWVRQAPGKGLEWVSAISGSGGSTYYADSVKGRFTISRDNSKNTLYLQMNSLRAEDTAVYYCARDRGPDYGDYGWFDYWGQGTLVTVSS

^^^^^^^^ ^^^^^^^^ ^^^^^^^^^^^^^^^^

Best Alignment of Lifastuzumab light chain to a sequence from OAS

DIQMTQSPSSLSASVGDRVTITCRSSETLVHSSGNTYLEWYQQKPGKAPKLLIYRVSNRFSGVPSRFSGSGSGTDFTLTISSLQPEDFATYYCFQGSFNPLTFGQGTKVEIK

||||||||||||||||||||||||.|.|......||||.|||||||||||||||..|...|||||||||||||||||||||||||||||||||.|.|..|.|||||||||||

DIQMTQSPSSLSASVGDRVTITCRASQTI-----NTYLNWYQQKPGKAPKLLIYAASSLQSGVPSRFSGSGSGTDFTLTISSLQPEDFATYYCQQSSSTPWTFGQGTKVEIK

^^^^^^^^^^^ ^^^ ^^^^^^^^^

Best Alignment of Lifastuzumab heavy chain CDRs to a sequence from OAS

EVQLVESGGGLVQPGGSLRLSCAASGFSFSDFAMSWVRQAPGKGLEWVATIGRVAFHTYYPDSMKGRFTISRDNSKNTLYLQMNSLRAEDTAVYYCARHRGFDVGHFDFWGQGTLVTVSS

................|||||||||||.||||.|.||||||||||||...|...|.||.|.||..||||.||||..|.|||||||||.||||||||||.||...|||||||||.||.|||

----------------SLRLSCAASGFTFSDFYMTWVRQAPGKGLEWISDISDTAAHTNYADSVRGRFTVSRDNAHNSLYLQMNSLRGEDTAVYYCARGRGDGFGHFDFWGQGRLVVVSS

^^^^^^^^ ^^^^^^^^ ^^^^^^^^^^^^^

Best Alignment of Lifastuzumab light chain CDRs to a sequence from OAS

DIQMTQSPSSLSASVGDRVTITCRSSETLVHSSGNTYLEWYQQKPGKAPKLLIYRVSNRFSGVPSRFSGSGSGTDFTLTISSLQPEDFATYYCFQGSFNPLTFGQGTKVEIK

.....|.|.||..|.||...|.||||..||||||||||.||.||||..||||||.|||||||||.|||||||||||||.||....||...|||||||..|||||.|||.|.|

-----QTPLSLPVSLGDQAAISCRSSLSLVHSSGNTYLHWYLQKPGQSPKLLIYKVSNRFSGVPDRFSGSGSGTDFTLKISRVEAEDLGVYYCFQGSHVPLTFGAGTKLELK

^^^^^^^^^^^ ^^^ ^^^^^^^^^

Best Alignment of Lifastuzumab CDR-H3 to a sequence from OAS

EVQLVESGGGLVQPGGSLRLSCAASGFSF-SDFAMSWVRQAPGKGLEWVATIGRVAFHTYYPDSMKGRFTISRDNSKNTLYLQMNSLRAEDTAVYYCARHRGFDVGHFDFWGQGTLVTVSS

.................|.|.|..||.|.......||||||||||||||.........|.|..|.|.|.|.|.|.|.|...|...||.|.||.||.||||||..||.||||||||||||||

--------------SQTLSLTCTVSGVSVTTTNWWSWVRQAPGKGLEWVGEVYQD-GSTNYNPSLKSRVTVSLDKSNNKFSLKLTSLTAADTGVYFCARHRGATVGPFDFWGQGTLVTVSS

^^^^^^^^^ ^^^^^^^^ ^^^^^^^^^^^^^

Therapeutic : Ligelizumab

Best Alignment of Ligelizumab heavy chain to a sequence from OAS

QVQLVQSGAEVMKPGSSVKVSCKASGYTFSWYWLEWVRQAPGHGLEWMGEIDPGTFTTNYNEKFKARVTFTADTSTSTAYMELSSLRSEDTAVYYCARFSHFSGSNYDYFDYWGQGTLVTVSS

|||||||||||.||||||||||||||.|||.|.|.|||||||.||||||.|.|...|.||..||..|||.|||.||||||||||||||||||||||||....|||.|.|||||||||||||||

QVQLVQSGAEVKKPGSSVKVSCKASGGTFSSYALSWVRQAPGQGLEWMGGIIPIFGTANYAQKFQGRVTITADESTSTAYMELSSLRSEDTAVYYCARVGIYSGSYYHYFDYWGQGTLVTVSS

^^^^^^^^ ^^^^^^^^ ^^^^^^^^^^^^^^^^

Best Alignment of Ligelizumab light chain to a sequence from OAS

EIVMTQSPATLSVSPGERATLSCRASQSIGTNIHWYQQKPGQAPRLLIYYASESISGIPARFSGSGSGTEFTLTISSLQSEDFAVYYCQQSWSWPTTFGGGTKVEIK

|||||||||||||||||||||||||||||..|..|||||||||||||||.||....||||||||||||||||||||||||||||||||||..|||.|||||||||||

EIVMTQSPATLSVSPGERATLSCRASQSISSNLAWYQQKPGQAPRLLIYGASTRATGIPARFSGSGSGTEFTLTISSLQSEDFAVYYCQQYNSWPLTFGGGTKVEIK

^^^^^^ ^^^ ^^^^^^^^^

Best Alignment of Ligelizumab heavy chain CDRs to a sequence from OAS

QVQLVQSGAEVMKPGSSVKVSCKASGYTFSWYWLEWVRQAPGHGLEWMGEIDPGTFTTNYNEKFKARVTFTADTSTSTAYMELSSLRSEDTAVYYCARFSHFSGSNYDYFDYWGQGTLVTVSS

.|||.|.|||...||||||.|||||||||..|||.||.|.|..|.||.|.|||....|.||.|||...|.|.|.|.|||||.||||.|||.||||||||||..||.|||||||||||..||||

-VQLQQPGAELVRPGSSVKLSCKASGYTFTSYWLHWVKQRPIQGHEWIGNIDPSDSETHYNQKFKDKATLTVDKSSSTAYMQLSSLTSEDSAVYYCARFSHYYGSSYDYFDYWGQGTTLTVSS

^^^^^^^^ ^^^^^^^^ ^^^^^^^^^^^^^^^^

Best Alignment of Ligelizumab light chain CDRs to a sequence from OAS

EIVMTQSPATLSVSPGERATLSCRASQSIGTNIHWYQQKPGQAPRLLIYYASESISGIPARFSGSGSGTEFTLTISSLQSEDFAVYYCQQSWSWPTTFGGGTKVEIK

.|..|||||.||||||||...|||||||||||||||||.....|||||.|||||.||||.|||||||||.|||.|.|..|||.|.||||||.|||.|||||||.|||

DILLTQSPAILSVSPGERVSFSCRASQSIGTNIHWYQQRTNGSPRLLIKYASESSSGIPSRFSGSGSGTDFTLSINSVESEDIADYYCQQSNSWPYTFGGGTKLEIK

^^^^^^ ^^^ ^^^^^^^^^

Best Alignment of Ligelizumab CDR-H3 to a sequence from OAS

QVQLVQSGAEVMKPGSSVKVSCKASGYTFSWYWLEWVRQAPGHGLEWMGEIDPGTFTTNYNEKFKARVTFTADTSTSTAYMELSSLRSEDTAVYYCARFSHFSGSNYDYFDYWGQGTLVTVSS

.|||.|.|||...||||||.|||||||||..|||.||.|.|..|.||.|.|||....|.||.|||...|.|.|.|.|||||.||||.|||.||||||||||..||.|||||||||||..||||

-VQLQQPGAELVRPGSSVKLSCKASGYTFTSYWLHWVKQRPIQGHEWIGNIDPSDSETHYNQKFKDKATLTVDKSSSTAYMQLSSLTSEDSAVYYCARFSHYYGSSYDYFDYWGQGTTLTVSS

^^^^^^^^ ^^^^^^^^ ^^^^^^^^^^^^^^^^

Therapeutic : Lintuzumab

Best Alignment of Lintuzumab heavy chain to a sequence from OAS

QVQLVQSGAEVKKPGSSVKVSCKASGYTFTDYNMHWVRQAPGQGLEWIGYIYPYNGGTGYNQKFKSKATITADESTNTAYMELSSLRSEDTAVYYCARGRP--AMDYWGQGTLVTVSS

|||||||||||||||||||||||||||||.||.||||||||||||||.|.|.|.||||.|.|||....||||||||.|||||||||||||||||||||.|...|.|||||||||||||

QVQLVQSGAEVKKPGSSVKVSCKASGYTFIDYYMHWVRQAPGQGLEWMGWINPNNGGTNYAQKFQGRVTITADESTSTAYMELSSLRSEDTAVYYCARLRSTSALDYWGQGTLVTVSS

^^^^^^^^ ^^^^^^^^ ^^^^^^^^^^^

Best Alignment of Lintuzumab light chain to a sequence from OAS

DIQMTQSPSSLSASVGDRVTITCRASESVDNYGISFMNWFQQKPGKAPKLLIYAASNQGSGVPSRFSGSGSGTDFTLTISSLQPDDFATYYCQQSKEVPWTFGQGTKVEIK

||||||||||||||||||||||||||.||.....||.||.||||||||||||||||...|||||||||||||||||||||||||||||||.||||...|||||||||||||

DIQMTQSPSSLSASVGDRVTITCRASQSV----SSFLNWYQQKPGKAPKLLIYAASRLQSGVPSRFSGSGSGTDFTLTISSLQPDDFATYSCQQSLVLPWTFGQGTKVEIK

^^^^^^^^^^ ^^^ ^^^^^^^^^

Best Alignment of Lintuzumab heavy chain CDRs to a sequence from OAS

QVQLVQSGAEVKKPGSSVKVSCKASGYTFTDYNMHWVRQAPGQGLEWIGYIYPYNGGTGYNQKFKSKATITADESTNTAYMELSSLRSEDTAVYYCARGRPAMDYWGQGTLVTVSS

.|||.|||.|..|||.|||.|||||||||||||||||.|..|..|||||||||||||||||||||||||.|.|.|..||||||.||.|||.|||||||||.|||||||||.|||||

-VQLQQSGPELVKPGASVKISCKASGYTFTDYNMHWVKQSHGKSLEWIGYIYPYNGGTGYNQKFKSKATLTVDNSSSTAYMELRSLTSEDSAVYYCARGRDAMDYWGQGTSVTVSS

^^^^^^^^ ^^^^^^^^ ^^^^^^^^^

Best Alignment of Lintuzumab light chain CDRs to a sequence from OAS

DIQMTQSPSSLSASVGDRVTITCRASESVDNYGISFMNWFQQKPGKAPKLLIYAASNQGSGVPSRFSGSGSGTDFTLTISSLQPDDFATYYCQQSKEVPWTFGQGTKVEIK

||..||||.||..|.|.|.||.|||||||||||||||||||||||..||||||||||||||||.|||||||||||.|.|.....||.|.|.||||||||||||.|||....

DIVLTQSPASLAVSLGQRATISCRASESVDNYGISFMNWFQQKPGQPPKLLIYAASNQGSGVPARFSGSGSGTDFSLNIHPMEEDDTAMYFCQQSKEVPWTFGGGTKLKSN

^^^^^^^^^^ ^^^ ^^^^^^^^^

Best Alignment of Lintuzumab CDR-H3 to a sequence from OAS

QVQLVQSGAEVKKPGSSVKVSCKASGYTFTDYNMHWVRQAPGQGLEWIGYIYPYNGGTGYNQKFKSKATITADESTNTAYMELSSLRSEDTAVYYCARGRPAMDYWGQGTLVTVSS

.|||.|.|||...||||||.||||||||||.|.|.||.|.|||||||||.|||....|.||||.|.|||.|.|.|..||||.||||.|||.||||||||||||||.||||.|||||

-VQLQQPGAELVRPGSSVKLSCKASGYTFTSYWMDWVKQRPGQGLEWIGNIYPSDSETHYNQKVKDKATLTVDKSSSTAYMQLSSLTSEDSAVYYCARGRPAMDYRGQGTSVTVSS

^^^^^^^^ ^^^^^^^^ ^^^^^^^^^

Therapeutic : Lirilumab

Best Alignment of Lirilumab heavy chain to a sequence from OAS

QVQLVQSGAEVKKPGSSVKVSCKASGGTFSFYAISWVRQAPGQGLEWMGGFIPIFGAANYAQKFQGRVTITADESTSTAYMELSSLRSDDTAVYYCARIPSGSY-----YYDYDMDVWGQGTTVTVSS

||||||||||||||||||||||||||||||.|||||||||||||||||||.|||||.|||||||||||||||||||||||||||||||.||||||||||.||||.....||.|.||||||||||||||

QVQLVQSGAEVKKPGSSVKVSCKASGGTFSSYAISWVRQAPGQGLEWMGGIIPIFGTANYAQKFQGRVTITADESTSTAYMELSSLRSEDTAVYYCARIRSGSYYNENYYYYYGMDVWGQGTTVTVSS

^^^^^^^^ ^^^^^^^^ ^^^^^^^^^^^^^^^^^^^^^

Best Alignment of Lirilumab light chain to a sequence from OAS

EIVLTQSPVTLSLSPGERATLSCRASQSVSSYLAWYQQKPGQAPRLLIYDASNRATGIPARFSGSGSGTDFTLTISSLEPEDFAVYYCQQRSNWMYTFGQGTKLEIK

||||||||.||||||||||||||||||||||||||||||||||||||||||||||||||||||||||||||||||||||||||||||||||||||||||||||||||

EIVLTQSPATLSLSPGERATLSCRASQSVSSYLAWYQQKPGQAPRLLIYDASNRATGIPARFSGSGSGTDFTLTISSLEPEDFAVYYCQQRSNWMYTFGQGTKLEIK

^^^^^^ ^^^ ^^^^^^^^^

Best Alignment of Lirilumab heavy chain CDRs to a sequence from OAS

QVQLVQSGAEVKKPGSSVKVSCKASGGTFSFYAISWVRQAPGQGLEWMGGFIPIFGAANYAQKFQGRVTITADESTSTAYMELSSLRSDDTAVYYCARIPSGSYYYDYDMDVWGQGTTVTVSS

..............|.||||||||||||||.|||||||||||||||||||.||||||||||||||||||||||||||||||||.||||.|||||||||..||||||.|||||||.||||||||

--------------GASVKVSCKASGGTFSSYAISWVRQAPGQGLEWMGGIIPIFGAANYAQKFQGRVTITADESTSTAYMELRSLRSEDTAVYYCARAHSGSYYYYYDMDVWGKGTTVTVSS

^^^^^^^^ ^^^^^^^^ ^^^^^^^^^^^^^^^^

Best Alignment of Lirilumab light chain CDRs to a sequence from OAS

EIVLTQSPVTLSLSPGERATLSCRASQSVSSYLAWYQQKPGQAPRLLIYDASNRATGIPARFSGSGSGTDFTLTISSLEPEDFAVYYCQQRSNWMYTFGQGTKLEIK

||||||||.||||||||||||||||||||||||||||||||||||||||||||||||||||||||||||||||||||||||||||||||||||||||||||||||||

EIVLTQSPATLSLSPGERATLSCRASQSVSSYLAWYQQKPGQAPRLLIYDASNRATGIPARFSGSGSGTDFTLTISSLEPEDFAVYYCQQRSNWMYTFGQGTKLEIK

^^^^^^ ^^^ ^^^^^^^^^

Best Alignment of Lirilumab CDR-H3 to a sequence from OAS

QVQLVQSGAEVKKPGSSVKVSCKASGGTF--SFYAISWVRQAPGQGLEWMGGFIPIFGAANYAQKFQGRVTITADESTSTAYMELSSLRSDDTAVYYCARIPSGSYYYDYDMDVWGQGTTVTVSS

.....................|..|||......|...|.||.||.||||.|..........|......||||..|.|.......|.|....|||||||||..||||||||||||||.||.|||||

----------------TLSLTCTVSGGSISSIGYFWTWIRQHPGKGLEWIGYKYCS-ESTDYNPSLKSRVTISVDTSENQFSLKLRSVTAADTAVYYCARGYSGSYYYDYDMDVWGKGTSVTVSS

^^^^^^^^^^ ^^^^^^^^ ^^^^^^^^^^^^^^^^

Therapeutic : Lodelcizumab

Best Alignment of Lodelcizumab heavy chain to a sequence from OAS

QVQLVQSGAEVKKPGASVKVSCKASGYTFSTMYMSWVRQAPGQGLEWMGRIDPANEHTNYAQKFQGRVTMTRDTSISTAYMELSRLTSDDTAVYYCARSYYY------YNMDYWGQGTLVTVSS

|||||||||||||||||||||||||||||...|..||||||||||||||||.|....|||||||||||||||||||||||||||||.|||||||||||.|||......|..|||||||||||||

QVQLVQSGAEVKKPGASVKVSCKASGYTFTGYYIQWVRQAPGQGLEWMGRINPNSGGTNYAQKFQGRVTMTRDTSISTAYMELSRLRSDDTAVYYCARVYYYDSSGYYYYFDYWGQGTLVTVSS

^^^^^^^^ ^^^^^^^^ ^^^^^^^^^^^^^^^^^

Best Alignment of Lodelcizumab light chain to a sequence from OAS

QIVLTQSPATLSVSPGERATLSCRASQSV--SYMHWYQQKPGQAPRLLIYGVFRRATGIPDRFSGSGSGTDFTLTIGRLEPEDFAVYYCLQWSS-DPPTFGQGTKLEIK

.||||||||||||||||||||||||||||..||..|||||||||||||||||..||||||||||||||||||||||.||||||||||||.|..|..|.|||||||||||

EIVLTQSPATLSVSPGERATLSCRASQSVSSSYLAWYQQKPGQAPRLLIYGVSTRATGIPDRFSGSGSGTDFTLTISRLEPEDFAVYYCQQYGSSPPYTFGQGTKLEIK

^^^^^^^ ^^^ ^^^^^^^^^^

Best Alignment of Lodelcizumab heavy chain CDRs to a sequence from OAS

QVQLVQSGAEVKKPGASVKVSCKASGYTFSTMYMSWVRQAPGQGLEWMGRIDPANEHTNYAQKFQGRVTMTRDTSISTAYMELSRLTSDDTAVYYCARSYYYYNMDYWGQGTLVTVSS

.|||.|.|||..|||||||.|||||||||....|.||.|.|.|||||.|||||||..|.|..||||..|.|.|||..|||..||.|||.||||||||||.|||.||||||||.|||||

-VQLQQPGAELVKPGASVKLSCKASGYTFTSYWMHWVKQRPEQGLEWIGRIDPANGNTKYDPKFQGKATITADTSSNTAYLQLSSLTSEDTAVYYCARSGYYYAMDYWGQGTSVTVSS

^^^^^^^^ ^^^^^^^^ ^^^^^^^^^^^

Best Alignment of Lodelcizumab light chain CDRs to a sequence from OAS

QIVLTQSPATLSVSPGERATLSCRASQSVSYMHWYQQKPGQAPRLLIYGVFRRATGIPDRFSGSGSGTDFTLTIGRLEPEDFAVYYCLQWSSDPPTFGQGTKLEIK

|||||||||..|.||||..|..|.||.|||||.|||||||..|||||||....|.|.|.|||||||||...|||.|.|.||.|.|||.||||.|||||.|||||||

QIVLTQSPAIMSASPGEKVTMTCSASSSVSYMYWYQQKPGSSPRLLIYGTSNLASGVPVRFSGSGSGTSYSLTISRMEAEDAATYYCQQWSSYPPTFGGGTKLEIK

^^^^^ ^^^ ^^^^^^^^^

Best Alignment of Lodelcizumab CDR-H3 to a sequence from OAS

QVQLVQSGAEVKKPGASVKVSCKASGYTF-STMYMSWVRQAPGQGLEWMGRIDPANEHTNYAQKFQGRVTMTRDTSISTAYMELSRLTSDDTAVYYCARSYYYYNMDYWGQGTLVTVSS

.|||..||....||..|....|...||...|.....|.||.||..|||||.|......|.|......|...|||.|.....|.|...|..|||.||||||||||.||||||||.|||||

EVQLQESGPGLVKPSQSLSLTCTVTGYSITSDYAWNWIRQFPGNKLEWMGYISYS-GNTSYNPSLKSRISITRDQSKNQFFMQLNSVTTEDTATYYCARSYYYYAMDYWGQGTSVTVSS

^^^^^^^^^ ^^^^^^^^ ^^^^^^^^^^^

Therapeutic : Lorvatuzumab

Best Alignment of Lorvatuzumab heavy chain to a sequence from OAS

QVQLVESGGGVVQPGRSLRLSCAASGFTFSSFGMHWVRQAPGKGLEWVAYISSGSFTIYYADSVKGRFTISRDNSKNTLYLQMNSLRAEDTAVYYCARMRKG--YAMDYWGQGTLVTVSS

.||||||||||||||||||||||||||||||.||||||||||||||||.||||.|.||||||||||||||||||.||.||||||||||||||||||||.|.|...|.|||||||||||||

-VQLVESGGGVVQPGRSLRLSCAASGFTFSSYGMHWVRQAPGKGLEWVSYISSSSSTIYYADSVKGRFTISRDNAKNSLYLQMNSLRAEDTAVYYCARDRVGAIGAFDYWGQGTLVTVSS

^^^^^^^^ ^^^^^^^^ ^^^^^^^^^^^^^

Best Alignment of Lorvatuzumab light chain to a sequence from OAS

DVVMTQSPLSLPVTLGQPASISCRSSQIIIHSDGNTYLEWFQQRPGQSPRRLIYKVSNRFSGVPDRFSGSGSGTDFTLKISRVEAEDVGVYYCFQGSH-VPHTFGQGTKVEIK

|||||||||||||||||||||||||||...||||||||.||||||||||||||||||||.|||||||||||||||||||||||||||||||||.||||..|.|||||||||||

DVVMTQSPLSLPVTLGQPASISCRSSQSLVHSDGNTYLNWFQQRPGQSPRRLIYKVSNRDSGVPDRFSGSGSGTDFTLKISRVEAEDVGVYYCMQGSHFPPWTFGQGTKVEIK

^^^^^^^^^^^ ^^^ ^^^^^^^^^^

Best Alignment of Lorvatuzumab heavy chain CDRs to a sequence from OAS

QVQLVESGGGVVQPGRSLRLSCAASGFTFSSFGMHWVRQAPGKGLEWVAYISSGSFTIYYADSVKGRFTISRDNSKNTLYLQMNSLRAEDTAVYYCARMRKGYAMDYWGQGTLVTVSS

.|||||||||.||||.|..||||||||||||||||||||||.|||||||||||||.||||||.|||||||||||.||||.||..|||.||||.|.|||.|.|||||||||||..||||

EVQLVESGGGLVQPGGSRKLSCAASGFTFSSFGMHWVRQAPEKGLEWVAYISSGSSTIYYADTVKGRFTISRDNPKNTLFLQKTSLRSEDTAMYDCARWRYGYAMDYWGQGTSLTVSS

^^^^^^^^ ^^^^^^^^ ^^^^^^^^^^^

Best Alignment of Lorvatuzumab light chain CDRs to a sequence from OAS

DVVMTQSPLSLPVTLGQPASISCRSSQIIIHSDGNTYLEWFQQRPGQSPRRLIYKVSNRFSGVPDRFSGSGSGTDFTLKISRVEAEDVGVYYCFQGSHVPHTFGQGTKVEIK

||||||...||||..|...|..|||.|.||||.|||||||..|.||..|..|||||||||.||||||||||||||||||||||||||.||||||||||||.|||.|||.|||

DVVMTQTAHSLPVSPGDQVSMPCRSGQSIIHSNGNTYLEWYLQKPGEYPKLLIYKVSNRFPGVPDRFSGSGSGTDFTLKISRVEAEDLGVYYCFQGSHVPWTFGRGTKLEIK

^^^^^^^^^^^ ^^^ ^^^^^^^^^

Best Alignment of Lorvatuzumab CDR-H3 to a sequence from OAS

QVQLVESGGGVVQPGRSLRLSCAASGFTF--SSFGMHWVRQAPGKGLEWVAYISSGSFTIYYADSVKGRFTISRDNSKNTLYLQMNSLRAEDTAVYYCARMRKGYAMDYWGQGTLVTVSS

||.|.|||.|..||...|.|.|..|||....|..|..|.||..||||||.|.|........|....|.|.|||.|.|.|...|...|....|||.|||||||||||||||||||.|||||

QVTLKESGPGILQPSQTLSLTCSFSGFSLSTSGMGVGWIRQPSGKGLEWLAHIWWD-DDKRYNPALKSRLTISKDTSSNQVFLKIASVDTADTATYYCARMRKGYAMDYWGQGTSVTVSS

^^^^^^^^^^ ^^^^^^^^ ^^^^^^^^^^^

Therapeutic : Lumiliximab

Best Alignment of Lumiliximab heavy chain to a sequence from OAS

EVQLVESGGGLAKPGGSLRLSCAASGFRFTFNNYYMDWVRQAPGQGLEWVSRISSSGDPTWYADSVKGRFTISRENANNTLFLQMNSLRAEDTAVYYCASLTT----GSDSWGQGVLVTVSS

.||||||||||.|||||||||||||||.|....|||.|.|||||.||||.|.|||||..|.|||||||||||||.||.|.|||||||||||||||||||||||....|.|.||||.||||||

QVQLVESGGGLVKPGGSLRLSCAASGFSF--SDYYMSWIRQAPGKGLEWLSYISSSGTYTTYADSVKGRFTISRDNAKNSLFLQMNSLRAEDTAVYYCASLTTVVTPGLDYWGQGTLVTVSS

^^^^^^^^^^ ^^^^^^^^ ^^^^^^^^^^^^^

Best Alignment of Lumiliximab light chain to a sequence from OAS

DIQMTQSPSSLSASVGDRVTITCRASQDIRYYLNWYQQKPGKAPKLLIYVASSLQSGVPSRFSGSGSGTEFTLTVSSLQPEDFATYYCLQVYSTPRTFGQGTKVEIK

|||||||||||||||||||||||||||.|..||||||||||||||||||.||||||||||||||||||||||||.|||||||||||||||.||||||||||||||||

DIQMTQSPSSLSASVGDRVTITCRASQSISSYLNWYQQKPGKAPKLLIYAASSLQSGVPSRFSGSGSGTEFTLTISSLQPEDFATYYCLQSYSTPRTFGQGTKVEIK

^^^^^^ ^^^ ^^^^^^^^^

Best Alignment of Lumiliximab heavy chain CDRs to a sequence from OAS

EVQLVESGGGLAKPGGSLRLSCAASGFRFTFNNYYMDWVRQAPGQGLEWVSRISSSGDPTWYADSVKGRFTISRENANNTLFLQMNSLRAEDTAVYYCASLTTGSDSWGQGVLVTVSS

...............|||.||||.|||.|....||..|.|||||.||||||.|||||....|||||||||||||.||.|.|.|||||||||||||||||.||||.|.||||.||||||

--------------WGSLCLSCAVSGFSFSSSGYYLGWIRQAPGKGLEWVSYISSSGSTIYYADSVKGRFTISRDNAKNSLYLQMNSLRAEDTAVYYCARLTTGFDYWGQGTLVTVSS

^^^^^^^^^^ ^^^^^^^^ ^^^^^^^^^

Best Alignment of Lumiliximab light chain CDRs to a sequence from OAS

DIQMTQSPSSLSASVGDRVTITCRASQDIRYYLNWYQQKPGKAPKLLIYVASSLQSGVPSRFSGSGSGTEFTLTVSSLQPEDFATYYCLQVYSTPRTFGQGTKVEIK

||||||||||||||.||||||||||||.||.||||||.||||||||||.|||.||||||||||||||||.||||.|||||||||||||||.||||||||||||||||

DIQMTQSPSSLSASEGDRVTITCRASQSIRNYLNWYQEKPGKAPKLLINVASILQSGVPSRFSGSGSGTDFTLTISSLQPEDFATYYCLQSYSTPRTFGQGTKVEIK

^^^^^^ ^^^ ^^^^^^^^^

Best Alignment of Lumiliximab CDR-H3 to a sequence from OAS

EVQLVESGGGLAKPGGSLRLSCAASGFRFTFNNYYMDWVRQAPGQGLEWVSRISSSGDPTWYADSVKGRFTISRENANNTLFLQMNSLRAEDTAVYYCASLTTGSDSWGQGVLVTVSS

||||.|||..|.|||.|...||.|||..|....|.|.||.|..|..|||...|......|.|....||..|........|...|.|||..||.||||||||||||.|||||...||||

EVQLQESGPELVKPGASVKISCKASGYSF--TDYNMNWVKQSNGKSLEWIGVINPNYGTTSYNQKFKGKATLTVDQSSSTAYMQLNSLTSEDSAVYYCASLTTGSSSWGQGTTHTVSS

^^^^^^^^^^ ^^^^^^^^ ^^^^^^^^^

Therapeutic : Margetuximab

Best Alignment of Margetuximab heavy chain to a sequence from OAS

QVQLQQSGPELVKPGASLKLSCTASGFNIKDTYIHWVKQRPEQGLEWIGRIYPTNGYTRYDPKFQDKATITADTSSNTAYLQVSRLTSEDTAVYYCSRWGGDG--FYAMDYWGQGASVTVSS

.|||||||.||||||||.|||||||||||||||.|||||||||||||||||.|.||.|.||||||.||||||||||||||||.|.|||||||||||.|.||||..||||||||||.||||||

EVQLQQSGAELVKPGASVKLSCTASGFNIKDTYMHWVKQRPEQGLEWIGRIDPANGNTKYDPKFQGKATITADTSSNTAYLQLSSLTSEDTAVYYCARGGGDGYPFYAMDYWGQGTSVTVSS

^^^^^^^^ ^^^^^^^^ ^^^^^^^^^^^^^^^

Best Alignment of Margetuximab light chain to a sequence from OAS

DIVMTQSHKFMSTSVGDRVSITCKASQDVNTAVAWYQQKPGHSPKLLIYSASFRYTGVPDRFTGSRSGTDFTFTISSVQAEDLAVYYCQQHYTTPPTFGGGTKVEIK

|||||||||||||||||||||||||||||.|||||||||||.||||||||||.||||||||||||.||||||||||||||||||||||||||.||||||||||.|||

DIVMTQSHKFMSTSVGDRVSITCKASQDVSTAVAWYQQKPGQSPKLLIYSASYRYTGVPDRFTGSGSGTDFTFTISSVQAEDLAVYYCQQHYSTPPTFGGGTKLEIK

^^^^^^ ^^^ ^^^^^^^^^

Best Alignment of Margetuximab heavy chain CDRs to a sequence from OAS

QVQLQQSGPELVKPGASLKLSCTASGFNIKDTYIHWVKQRPEQGLEWIGRIYPTNGYTRYDPKFQDKATITADTSSNTAYLQVSRLTSEDTAVYYCSRWGGDGFYAMDYWGQGASVTVSS

.|.|..||.||||||||.|||||||||||||||.|||||||||||||||||.|.||.|.||||||.||||||||||||||||.|.||||||||||..||||||.|||||||||.||||||

EVMLVESGAELVKPGASVKLSCTASGFNIKDTYMHWVKQRPEQGLEWIGRIDPANGNTKYDPKFQGKATITADTSSNTAYLQLSSLTSEDTAVYYGARWGGDGVYAMDYWGQGPSVTVSS

^^^^^^^^ ^^^^^^^^ ^^^^^^^^^^^^^

Best Alignment of Margetuximab light chain CDRs to a sequence from OAS

DIVMTQSHKFMSTSVGDRVSITCKASQDVNTAVAWYQQKPGHSPKLLIYSASFRYTGVPDRFTGSRSGTDFTFTISSVQAEDLAVYYCQQHYTTPPTFGGGTKVEIK

||.|||||||||||||||||||||||||||||||||||||||||||||||||||.||||||||||.|||||||||.||||||||||||||||.||||||||||.|||

DIMMTQSHKFMSTSVGDRVSITCKASQDVNTAVAWYQQKPGHSPKLLIYSASFRCTGVPDRFTGSGSGTDFTFTINSVQAEDLAVYYCQQHYSTPPTFGGGTKLEIK

^^^^^^ ^^^ ^^^^^^^^^

Best Alignment of Margetuximab CDR-H3 to a sequence from OAS

QVQLQQSGPELVKPGASLKLSCTASGFNIKDTYIHWVKQRPEQGLEWIGRIYPTNGYTRYDPKFQDKATITADTSSNTAYLQVSRLTSEDTAVYYCSRWGGDGFYAMDYWGQGASVTVSS

||||||.|.|||.||||.||||.|||.........||||||||||||||||.|.....||..||.|||..|.|.||.|||.|.|.|||||.||||||||||..||||||||||.||||||

QVQLQQPGAELVRPGASVKLSCKASGYTFTNYWMNWVKQRPEQGLEWIGRIDPYDSEIRYNQKFKDKAILTVDKSSRTAYMQLSSLTSEDSAVYYCSRWGGITFYAMDYWGQGTSVTVSS

^^^^^^^^ ^^^^^^^^ ^^^^^^^^^^^^^

Therapeutic : Matuzumab

Best Alignment of Matuzumab heavy chain to a sequence from OAS

QVQLVQSGAEVKKPGASVKVSCKASGYTFTSHWMHWVRQAPGQGLEWIGEFNPSNGRTNYNEKFKSKATMTVDTSTNTAYMELSSLRSEDTAVYYCASRDYDYDGRYFDYWGQGTLVTVSS

||||.|||||..|||||||.|||||||||||.|||||.|.||||||||||.||||||||||||||||||.|||.|..||||.||||.|||.|||||||.||||||.|||.||.||.|||||

QVQLQQSGAELVKPGASVKLSCKASGYTFTSYWMHWVKQRPGQGLEWIGEINPSNGRTNYNEKFKSKATLTVDKSSSTAYMQLSSLTSEDSAVYYCASVDYDYDGGYFDVWGAGTTVTVSS

^^^^^^^^ ^^^^^^^^ ^^^^^^^^^^^^^^

Best Alignment of Matuzumab light chain to a sequence from OAS

DIQMTQSPSSLSASVGDRVTITCSASSSV-TYMYWYQQKPGKAPKLLIYDTSNLASGVPSRFSGSGSGTDYTFTISSLQPEDIATYYCQQWSSHIFTFGQGTKVEIK

|||||||||||||||||||||||.||..|..|..|||||||||||||||..|||.|||||||||||||||||||||||||||.|||||||..|...|||||||||||

DIQMTQSPSSLSASVGDRVTITCRASENVNNYLNWYQQKPGKAPKLLIYKASNLQSGVPSRFSGSGSGTDYTFTISSLQPEDVATYYCQQHNSYPRTFGQGTKVEIK

^^^^^^ ^^^ ^^^^^^^^^

Best Alignment of Matuzumab heavy chain CDRs to a sequence from OAS

QVQLVQSGAEVKKPGASVKVSCKASGYTFTSHWMHWVRQAPGQGLEWIGEFNPSNGRTNYNEKFKSKATMTVDTSTNTAYMELSSLRSEDTAVYYCASRDYDYDGRYFDYWGQGTLVTVSS

||||.|.|||..|||||||.|||||||||||.|||||.|.||||||||||.||||||||||||||||||.|||.|..||||.||||.|||.|||||||.||||||.|||.||.||.|||||

QVQLQQPGAELVKPGASVKLSCKASGYTFTSYWMHWVKQRPGQGLEWIGEINPSNGRTNYNEKFKSKATLTVDKSSSTAYMQLSSLTSEDSAVYYCASVDYDYDGGYFDVWGAGTTVTVSS

^^^^^^^^ ^^^^^^^^ ^^^^^^^^^^^^^^

Best Alignment of Matuzumab light chain CDRs to a sequence from OAS

DIQMTQSPSSLSASVGDRVTITCSASSSVTYMYWYQQKPGKAPKLLIYDTSNLASGVPSRFSGSGSGTDYTFTISSLQPEDIATYYCQQWSSHIFTFGQGTKVEIK

.|..||||....||.|..||.|||||||||||.|||||.|..||..|||||.||||||.|||||||||.|..||||...||.||||||||||..||||.|||.|||

QIVLTQSPAIMPASPGEKVTMTCSASSSVTYMHWYQQKSGTSPKRWIYDTSKLASGVPARFSGSGSGTSYSLTISSMEAEDAATYYCQQWSSNPFTFGSGTKLEIK

^^^^^ ^^^ ^^^^^^^^^

Best Alignment of Matuzumab CDR-H3 to a sequence from OAS

QVQLVQSGAEVKKPGASVKVSCKASGYTFTSHWMHWVRQAPGQGLEWIGEFNPSNGRTNYNEKFKSKATMTVDTSTNTAYMELSSLRSEDTAVYYCASRDYDYDGRYFDYWGQGTLVTVSS

.||.|.||....|||.|.|.||.|||.||....||||||||..||||............|....|...|...|...||......||||||||.||||||||||||.|||||||||..||||

EVQRVESGGGLVKPGGSLKLSCAASGFTFSDYGMHWVRQAPEKGLEWVAYISSGSSTIYYADTVKGRFTISRDNAKNTLFLQMTSLRSEDTAMYYCASRDYDYDGGYFDYWGQGTTLTVSS

^^^^^^^^ ^^^^^^^^ ^^^^^^^^^^^^^^

Therapeutic : Mavrilimumab

Best Alignment of Mavrilimumab heavy chain to a sequence from OAS

QVQLVQSGAEVKKPGASVKVSCKVSGYTLTELSIHWVRQAPGKGLEWMGGFDPEENEIVYAQRFQGRVTMTEDTSTDTAYMELSSLRSEDTAVYYCAIVGSFS-PLTLGLWGQGTMVTVSS

|||||||||||||||||||||||||||||||||.||||||||||||||||||||..|..|||.||||||||||||||||||||||||||||||||||.|||.|..|.||.|||||.|||||

QVQLVQSGAEVKKPGASVKVSCKVSGYTLTELSMHWVRQAPGKGLEWMGGFDPEDGETIYAQKFQGRVTMTEDTSTDTAYMELSSLRSEDTAVYYCARVGSSSWYLGLGYWGQGTLVTVSS

^^^^^^^^ ^^^^^^^^ ^^^^^^^^^^^^^^

Best Alignment of Mavrilimumab light chain to a sequence from OAS

QSVLTQPPSVSGAPGQRVTISCTGSGSNIGAPYDVSWYQQLPGTAPKLLIYHNNKRPSGVPDRFSGSKSGTSASLAITGLQAEDEADYYCATVEAGLSGSVFGGGTKLTVL

|||||||||||||||||||||||||.|||||||||.|||||||||||||||.||.|||||||||||||||||||||||||||||||||||.|....|||||||||||||||

QSVLTQPPSVSGAPGQRVTISCTGSSSNIGAPYDVHWYQQLPGTAPKLLIYGNNNRPSGVPDRFSGSKSGTSASLAITGLQAEDEADYYCQTYDNSLSGSVFGGGTKLTVL

^^^^^^^^^ ^^^ ^^^^^^^^^^^

Best Alignment of Mavrilimumab heavy chain CDRs to a sequence from OAS

QVQLVQSGAEVKKPGASVKVSCKVSGYTLTELSIHWVRQAPGKGLEWMGGFDPEENEIVYAQRFQGRVTMTEDTSTDTAYMELSSLRSEDTAVYYCAIVGSFSPLTLGLWGQGTMVTVSS

................|||||||||||||||||.||||||||||||||||||||..|..|||.||||||||||||||||||.|||||||||||||||..||.|.||||.|||||.|||||

----------------SVKVSCKVSGYTLTELSMHWVRQAPGKGLEWMGGFDPEDGETIYAQKFQGRVTMTEDTSTDTAYMVLSSLRSEDTAVYYCATDGSGSYLTLGYWGQGTLVTVSS

^^^^^^^^ ^^^^^^^^ ^^^^^^^^^^^^^

Best Alignment of Mavrilimumab light chain CDRs to a sequence from OAS

QSVLTQPPSVSGAPGQRVTISCTGSGSNIGAPYDVSWYQQLPGTAPKLLIYHNNKRPSGVPDRFSGSKSGTSASLAITGLQAEDEADYYCATVEAGLSGSVFGGGTKLTVL

|||||||||||||||||||||||||.|||||||||.||.||||.|||||||.||.||||.|||||||||||||.|.|||||..|||||||||...||||.|||||||||||

QSVLTQPPSVSGAPGQRVTISCTGSRSNIGAPYDVHWYKQLPGLAPKLLIYENNERPSGIPDRFSGSKSGTSATLDITGLQTGDEADYYCATWDDGLSGRVFGGGTKLTVL

^^^^^^^^^ ^^^ ^^^^^^^^^^^

Best Alignment of Mavrilimumab CDR-H3 to a sequence from OAS

QVQLVQSGAEVKKPGASVKVSCKVSGYTLTELSIHWVRQAPGKGLEWMGGFDPEENEIVYAQRFQGRVTMTEDTSTDTAYMELSSLRSEDTAVYYCAIVGSFSPLTLGLWGQGTMVTVSS

................|||||||.||||.|...|..||||||.||||||...|......|||.||.|||.|.|.|..|||||||||||||||.||||...|.||||||.|||||.|||||

----------------SVKVSCKASGYTFTSYGISRVRQAPGQGLEWMGWITPFNGNTNYAQKFQDRVTITRDRSMSTAYMELSSLRSEDTAMYYCAGGDSSSPLTLGYWGQGTLVTVSS

^^^^^^^^ ^^^^^^^^ ^^^^^^^^^^^^^

Therapeutic : Mepolizumab

Best Alignment of Mepolizumab heavy chain to a sequence from OAS

QVTLRESGPALVKPTQTLTLTCTVSGFSL--TSYSVHWVRQPPGKGLEWLGVIWASGGTDYNSALMSRLSISKDTSRNQVVLTMTNMDPVDTATYYCARDPPSS----LLRLDYWGRGTPVTVSS

|||||||||||||||||||||||.|||||......|.|.||||||.||||.||.......||..|..|||||||||.||||||||||||||||||||||....|.......|||||.||.|||||

QVTLRESGPALVKPTQTLTLTCTFSGFSLNTDEMCVSWIRQPPGKALEWLAVIHRDDNKFYNTSLKTRLSISKDTSKNQVVLTMTNMDPVDTATYYCARIRNYSGYNGYGGLDYWGQGTLVTVSS

^^^^^^^^^^ ^^^^^^^ ^^^^^^^^^^^^^^^^^

Best Alignment of Mepolizumab light chain to a sequence from OAS

DIVMTQSPDSLAVSLGERATINCKSSQSLLNSGNQKNYLAWYQQKPGQPPKLLIYGASTRESGVPDRFSGSGSGTDFTLTISSLQAEDVAVYYCQNVHSFPFTFGGGTKLEIK

||||||||||||||||||||||||||||.|.|.|.||||||||||||||||||||.|||||||||||||||||||||||||||||||||||||||..||.|||||.|||||||

DIVMTQSPDSLAVSLGERATINCKSSQSVLYSSNNKNYLAWYQQKPGQPPKLLIYWASTRESGVPDRFSGSGSGTDFTLTISSLQAEDVAVYYCQQDHSPPFTFGQGTKLEIK

^^^^^^^^^^^^ ^^^ ^^^^^^^^^

Best Alignment of Mepolizumab heavy chain CDRs to a sequence from OAS

QVTLRESGPALVKPTQTLTLTCTVSGFSLTSYSVHWVRQPPGKGLEWLGVIWASGGTDYNSALMSRLSISKDTSRNQVVLTMTNMDPVDTATYYCARDPPSSLLRLDYWGRGTPVTVSS

||.|.||||.||.|.|.|..||||||||||||.||||||||||||||||||||.|.|.||||||||||||||.|..||.|.|......|.|.||||||.|..||||||||.||.|||||

QVQLKESGPGLVAPSQSLSITCTVSGFSLTSYGVHWVRQPPGKGLEWLGVIWAGGSTNYNSALMSRLSISKDNSKSQVCLKMNSLQIDDPAMYYCARDAPLLLLRLDYWGQGTSVTVSS

^^^^^^^^ ^^^^^^^ ^^^^^^^^^^^^^

Best Alignment of Mepolizumab light chain CDRs to a sequence from OAS

DIVMTQSPDSLAVSLGERATINCKSSQSLLNSGNQKNYLAWYQQKPGQPPKLLIYGASTRESGVPDRFSGSGSGTDFTLTISSLQAEDVAVYYCQNVHSFPFTFGGGTKLEIK

||||||||.||.||.||..|..||||||||||||||||||||||||||||||||||||||||||||||.||||||||||||||.||||.||||||||||.|||||.|||||||

DIVMTQSPSSLSVSAGEKVTMSCKSSQSLLNSGNQKNYLAWYQQKPGQPPKLLIYGASTRESGVPDRFTGSGSGTDFTLTISSVQAEDLAVYYCQNVHSYPFTFGSGTKLEIK

^^^^^^^^^^^^ ^^^ ^^^^^^^^^

Best Alignment of Mepolizumab CDR-H3 to a sequence from OAS

QVTLRESGPALVKPTQTLTLTCTVSGFSLTSYSVHWVRQPPGKGLEWLGVIWAS-GGTDYNSALMSRLSISKDTSRNQVVLTMTNMDPVDTATYYCARDPPSSLLRLDYWGRGTPVTVSS

.............|...|.|.|..||||...|..|||||.|||||||...||.......|......|..||.|.|.|...|.|......|||.||||||||||.|.|||||.||||||||

-------------PGGSLRLSCAASGFSFSDYGMHWVRQAPGKGLEWVAFIWYDGNNIKYADSVKGRFTISRDNSKNTMYLQMNTLRVEDTAVYYCARDPPSSGLHLDYWGQGTPVTVSS

^^^^^^^^ ^^^^^^^^ ^^^^^^^^^^^^^

Therapeutic : Mirikizumab

Best Alignment of Mirikizumab heavy chain to a sequence from OAS

QVQLVQSGAEVKKPGSSVKVSCKASGYKFTRYVMHWVRQAPGQGLEWMGYINPYNDGTNYNEKFKGRVTITADKSTSTAYMELSSLRSEDTAVYYCARNW---------------DTGLWGQGTTVTVSS

|||||||||||||||||||||||||||.||.|.||||||||||||||||.|||...||||..||.|||||||||||||||||||||||||||||||||.....................|||||||||||

QVQLVQSGAEVKKPGSSVKVSCKASGYTFTGYYMHWVRQAPGQGLEWMGWINPNSGGTNYAQKFQGRVTITADKSTSTAYMELSSLRSEDTAVYYCARERIAAAAKSSDHHYYYYGMDVWGQGTTVTVSS

^^^^^^^^ ^^^^^^^^ ^^^^^^^^^^^^^^^^^^^^^^^

Best Alignment of Mirikizumab light chain to a sequence from OAS

DIQMTQSPSSLSASVGDRVTITCKASDHILKFLTWYQQKPGKAPKLLIYGATSLETGVPSRFSGSGSGTDFTLTISSLQPEDFATYYCQMYWSTPFTFGGGTKVEIK

|||||||||||||||||||||||.||..|...|.|||||||||||||||.|.|||||||||||||||||||||||||||||||||||||.|.|||.|||||||||||

DIQMTQSPSSLSASVGDRVTITCRASQGISSYLNWYQQKPGKAPKLLIYAASSLETGVPSRFSGSGSGTDFTLTISSLQPEDFATYYCQQYDSTPITFGGGTKVEIK

^^^^^^ ^^^ ^^^^^^^^^

Best Alignment of Mirikizumab heavy chain CDRs to a sequence from OAS

QVQLVQSGAEVKKPGSSVKVSCKASGYKFTRYVMHWVRQAPGQGLEWMGYINPYNDGTNYNEKFKGRVTITADKSTSTAYMELSSLRSEDTAVYYCARNWDTGLWGQGTTVTVSS

.|||.|||.|..|||.|||.|||||||||||||||||.|.|||||||.||||||||||.|||||||..|.|.|||.||||||||||.|||.||||||.|||...|||.|.||||.

EVQLQQSGPELVKPGASVKMSCKASGYKFTRYVMHWVKQKPGQGLEWIGYINPYNDGTQYNEKFKGKATLTSDKSSSTAYMELSSLTSEDSAVYYCAPNWDSAYWGQWTLVTVSA

^^^^^^^^ ^^^^^^^^ ^^^^^^^^

Best Alignment of Mirikizumab light chain CDRs to a sequence from OAS

DIQMTQSPSSLSASVGDRVTITCKASDHILKFLTWYQQKPGKAPKLLIYGATSLETGVPSRFSGSGSGTDFTLTISSLQPEDFATYYCQMYWSTPFTFGGGTKVEIK

|||||||.|.||.|.|.||||||||||||.|.|.|||||||.||.|||.|||||||||||||||||||...||.|.|||.||.||||||.|||||.|||||||.|||

DIQMTQSSSYLSVSLGGRVTITCKASDHINKWLAWYQQKPGNAPRLLISGATSLETGVPSRFSGSGSGKNYTLNITSLQTEDVATYYCQQYWSTPYTFGGGTKLEIK

^^^^^^ ^^^ ^^^^^^^^^

Best Alignment of Mirikizumab CDR-H3 to a sequence from OAS

QVQLVQSGAEVKKPGSSVKVSCKASGYKFTRYVMHWVRQAPGQGLEWMGYINPYNDGTNYNEKFKGRVTITADKSTSTAYMELSSLRSEDTAVYYCARNWDTGLWGQGTTVTVSS

................|||||||||||.||.|..|||||||||...||||||.....|.|......|||||.|.|.||.||.||.|.|||||||||||.||||||||||.|||||

---------------ASVKVSCKASGYIFTNYAIHWVRQAPGQRPQWMGYINTAIGNTKYSQSLQDRVTITSDTSASTVYMHLSRLTSEDTAVYYCARDWDTGLWGQGTLVTVSS

^^^^^^^^ ^^^^^^^^ ^^^^^^^^

Therapeutic : Mirvetuximab

Best Alignment of Mirvetuximab heavy chain to a sequence from OAS

QVQLVQSGAEVVKPGASVKISCKASGYTFTGYFMNWVKQSPGQSLEWIGRIHPYDGDTFYNQKFQGKATLTVDKSSNTAHMELLSLTSEDFAVYYCTRYDGS---RAMDYWGQGTTVTVSS

.|||.|||.|.||||||||||||||||.||||||||||||.||||||||||.||.|||||||||.|||||||||||.|||||||||||||||||||.||.||....|||||||||.|||||

-VQLQQSGPELVKPGASVKISCKASGYSFTGYFMNWVKQSHGQSLEWIGRINPYNGDTFYNQKFKGKATLTVDKSSSTAHMELLSLTSEDFAVYYCARYYGSSYDYAMDYWGQGTSVTVSS

^^^^^^^^ ^^^^^^^^ ^^^^^^^^^^^^^^

Best Alignment of Mirvetuximab light chain to a sequence from OAS

DIVLTQSPLSLAVSLGQPAIISCKASQSVSFAGTSLMHWYHQKPGQQPRLLIYRASNLEAGVPDRFSGSGSKTDFTLTISPVEAEDAATYYCQQSREYPYTFGGGTKLEIK

||||||||.||||||||.||||||||||||||||||||||.|||||||.||||||||||||||.|||||||.|||||.|.|||..||||||||||||||.|||||||||||

DIVLTQSPASLAVSLGQRAIISCKASQSVSFAGTSLMHWYQQKPGQQPKLLIYRASNLEAGVPTRFSGSGSRTDFTLNIHPVEEDDAATYYCQQSREYPRTFGGGTKLEIK

^^^^^^^^^^ ^^^ ^^^^^^^^^

Best Alignment of Mirvetuximab heavy chain CDRs to a sequence from OAS

QVQLVQSGAEVVKPGASVKISCKASGYTFTGYFMNWVKQSPGQSLEWIGRIHPYDGDTFYNQKFQGKATLTVDKSSNTAHMELLSLTSEDFAVYYCTRYDGSRAMDYWGQGTTVTVSS

.|||.|.|.|.||||||||||||||||.|||||||||.||.|.||||||||.||||||||||||.|||||||||||.||||||.||.|||.|||||.||.|..|||||||||.|||||

-VQLQQPGPELVKPGASVKISCKASGYSFTGYFMNWVMQSHGKSLEWIGRINPYDGDTFYNQKFKGKATLTVDKSSSTAHMELRSLASEDSAVYYCARYYGQGAMDYWGQGTSVTVSS

^^^^^^^^ ^^^^^^^^ ^^^^^^^^^^^

Best Alignment of Mirvetuximab light chain CDRs to a sequence from OAS

DIVLTQSPLSLAVSLGQPAIISCKASQSVSFAGTSLMHWYHQKPGQQPRLLIYRASNLEAGVP-DRFSGSGSKTDFTLTISPVEAEDAATYYCQQSREYPYTFGGGTKLEIK

||||||||.||||||||.||||||||||||||||||||||.|||||||.||||||||||||.........|........|.|||..||||||||||||||||||||||||||

DIVLTQSPASLAVSLGQRAIISCKASQSVSFAGTSLMHWYQQKPGQQPKLLIYRASNLEAGGSYQGLVAVGLGQTSPSNIHPVEEDDAATYYCQQSREYPYTFGGGTKLEIK

^^^^^^^^^^ ^^^ ^^^^^^^^^

Best Alignment of Mirvetuximab CDR-H3 to a sequence from OAS

QVQLVQSGAEVVKPGASVKISCKASGYTFTGYFMNWVKQSPGQSLEWIGRIHPYDGDTFYNQKFQGKATLTVDKSSNTAHMELLSLTSEDFAVYYCTRYDGSRAMDYWGQGTTVTVSS

||||.|||||.|.|||||..||||||||||.|.|.||||.|...|||||.|.|..|.|.|||||.|||.||.||||.||.|||.||||||.|||||||||.|||||||||||.|||||

QVQLQQSGAELVRPGASVALSCKASGYTFTDYEMHWVKQTPVHGLEWIGAIDPETGGTAYNQKFKGKAILTADKSSSTAYMELRSLTSEDSAVYYCTRYDSSRAMDYWGQGTSVTVSS

^^^^^^^^ ^^^^^^^^ ^^^^^^^^^^^

Therapeutic : Modotuximab

Best Alignment of Modotuximab heavy chain to a sequence from OAS

QVQLQQPGAELVEPGGSVKLSCKASGYTFTSHWMHWVKQRPGQGLEWIGEINPSSGRNNYNEKFKSKATLTVDKSSSTAYMQFSSLTSEDSAVYYCVRYYGYDEAMDYWGQGTSVTVSS

||||||||||||.||.|||||||||||||||.||||||||||||||||||||||.||.||||||||||||||||||||||||.|||||||||||||.|||||.||||||||||||||||

QVQLQQPGAELVKPGASVKLSCKASGYTFTSYWMHWVKQRPGQGLEWIGEINPSNGRTNYNEKFKSKATLTVDKSSSTAYMQLSSLTSEDSAVYYCARYYGY-EAMDYWGQGTSVTVSS

^^^^^^^^ ^^^^^^^^ ^^^^^^^^^^^^

Best Alignment of Modotuximab light chain to a sequence from OAS

DIVMTQAAFSNPVTLGTSASISCRSSKSLLHSNGITYLYWYLQKPGQSPQLLIYQMSNLASGVPDRFSSSGSGTDFTLRISRVEAEDVGVYYCAQNLELPYTFGGGTKLEIK

||||||||||||||||||||||||||||||||||||||||||||||||||||||||||||||||||||||||||||||||||||||||||||||||||||||||||||||||

DIVMTQAAFSNPVTLGTSASISCRSSKSLLHSNGITYLYWYLQKPGQSPQLLIYQMSNLASGVPDRFSSSGSGTDFTLRISRVEAEDVGVYYCAQNLELPYTFGGGTKLEIK

^^^^^^^^^^^ ^^^ ^^^^^^^^^

Best Alignment of Modotuximab heavy chain CDRs to a sequence from OAS

QVQLQQPGAELVEPGGSVKLSCKASGYTFTSHWMHWVKQRPGQGLEWIGEINPSSGRNNYNEKFKSKATLTVDKSSSTAYMQFSSLTSEDSAVYYCVRYYGYDEAMDYWGQGTSVTVSS

.|||||.||||..||.|||||||||||||||.|||||||||||||||||.||||||...||.|||.||||||.|||||||.|.||||.|||||..|.|.||||||||||||||||.|||

-VQLQQSGAELAKPGASVKLSCKASGYTFTSYWMHWVKQRPGQGLEWIGYINPSSGYTKYNQKFKDKATLTVEKSSSTAYIQLSSLTYEDSAVSSCARVYGYDEAMDYWGQGTSVAVSS

^^^^^^^^ ^^^^^^^^ ^^^^^^^^^^^^

Best Alignment of Modotuximab light chain CDRs to a sequence from OAS

DIVMTQAAFSNPVTLGTSASISCRSSKSLLHSNGITYLYWYLQKPGQSPQLLIYQMSNLASGVPDRFSSSGSGTDFTLRISRVEAEDVGVYYCAQNLELPYTFGGGTKLEIK

.|.||.||...||.|..||||||.||||||||||||||||||||||||||||||||||||||||||||||||||||||||||||||||||||||||||||||||||||||||

-IAMTPAACCSPVSLLPSASISCGSSKSLLHSNGITYLYWYLQKPGQSPQLLIYQMSNLASGVPDRFSSSGSGTDFTLRISRVEAEDVGVYYCAQNLELPYTFGGGTKLEIK

^^^^^^^^^^^ ^^^ ^^^^^^^^^

Best Alignment of Modotuximab CDR-H3 to a sequence from OAS

QVQLQQPGAELVEPGGSVKLSCKASGYTFTSHWMHWVKQRPGQGLEWIGEINPS--SGRNNYNEKFKSKATLTVDKSSSTAYMQFSSLTSEDSAVYYCVRYYGYDEAMDYWGQGTSVTVSS

.|.|...|..||.||||..|||.|||.|||...|.||.|.||..|||.|.|..........|....|...|...|.|.|..|.|...|...|||.|||.||||||||||||||||||||||

EVKLVESGGGLVQPGGSLSLSCAASGFTFTDYYMSWVRQPPGKALEWLGFIRNKANGYTTEYSASVKGRFTISRDNSQSILYLQMNALRADDSATYYCARYYGYDEAMDYWGQGTSVTVSS

^^^^^^^^ ^^^^^^^^^^ ^^^^^^^^^^^^

Therapeutic : Mogamulizumab

Best Alignment of Mogamulizumab heavy chain to a sequence from OAS

EVQLVESGGDLVQPGRSLRLSCAASGFIFSNYGMSWVRQAPGKGLEWVATISSASTYSYYPDSVKGRFTISRDNAKNSLYLQMNSLRVEDTALYYCGRHSDG-NFAFGYWGQGTLVTVSS

||||.||||.|||||.|||||||||||.||.|.||||||||||||||||||||.|.|.||.||||||||||||||||||||||||||.||||.|||.|..||.||...||||||||||||

EVQLLESGGGLVQPGGSLRLSCAASGFTFSSYAMSWVRQAPGKGLEWVATISSSSSYIYYTDSVKGRFTISRDNAKNSLYLQMNSLRAEDTAVYYCARSRDGYNFDIDYWGQGTLVTVSS

^^^^^^^^ ^^^^^^^^ ^^^^^^^^^^^^^

Best Alignment of Mogamulizumab light chain to a sequence from OAS

DVLMTQSPLSLPVTPGEPASISCRSSRNIVHINGDTYLEWYLQKPGQSPQLLIYKVSNRFSGVPDRFSGSGSGTDFTLKISRVEAEDVGVYYCFQGSLLPWTFGQGTKVEIK

||.|||||||||||||||||||||||.....|||.|||||||||||||||||||.||||.|||||||||||||||||||||||||||||||||.||...|||||||||||||

DVVMTQSPLSLPVTPGEPASISCRSSQSLMPINGYTYLEWYLQKPGQSPQLLIYLVSNRASGVPDRFSGSGSGTDFTLKISRVEAEDVGVYYCMQGLQIPWTFGQGTKVEIK

^^^^^^^^^^^ ^^^ ^^^^^^^^^

Best Alignment of Mogamulizumab heavy chain CDRs to a sequence from OAS

EVQLVESGGDLVQPGRSLRLSCAASGFIFSNYGMSWVRQAPGKGLEWVATISSASTYSYYPDSVKGRFTISRDNAKNSLYLQMNSLRVEDTALYYCGRHSDGNFAFGYWGQGTLVTVSS

||||||||||||.||.||.||||||||.||.||||||||.|.|.|||||||||...|||||||||||||||||||||.|||||.||..||||.|||.||.||||.|.||||||..||||

EVQLVESGGDLVKPGGSLKLSCAASGFTFSSYGMSWVRQTPDKRLEWVATISSGGSYSYYPDSVKGRFTISRDNAKNTLYLQMSSLKSEDTAMYYCARHEDGNFHFDYWGQGTTLTVSS

^^^^^^^^ ^^^^^^^^ ^^^^^^^^^^^^

Best Alignment of Mogamulizumab light chain CDRs to a sequence from OAS

DVLMTQSPLSLPVTPGEPASISCRSSRNIVHINGDTYLEWYLQKPGQSPQLLIYKVSNRFSGVPDRFSGSGSGTDFTLKISRVEAEDVGVYYCFQGSLLPWTFGQGTKVEIK

||.|||.||.|||..|..||||||||..|||.|||||||||||||||||.|||||||||||||||||||.|||||||||||||||||.|||||||||..|||||.|||.|||

DVVMTQTPLFLPVRLGDHASISCRSSQSIVHSNGDTYLEWYLQKPGQSPKLLIYKVSNRFSGVPDRFSGGGSGTDFTLKISRVEAEDLGVYYCFQGSHVPWTFGGGTKLEIK

^^^^^^^^^^^ ^^^ ^^^^^^^^^

Best Alignment of Mogamulizumab CDR-H3 to a sequence from OAS

EVQLVESGGDLVQPGRSLRLSCAASGFIFSNYGMSWVRQAPGKGLEWVATISSASTYSYYPDSVKGRFTISRDNAKNSLYLQMNSLRVEDTALYYCGRHSDGNFAFGYWGQGTLVTVSS

|||.|||||.||.||.||.||||||||.||.|.||||||.|.|.|||||||||...|.||||||.||||||||||||.|.|||..||.||.|.|||.|.|||||.|||||||||||||.

EVQGVESGGGLVKPGGSLKLSCAASGFTFSSYAMSWVRQTPEKRLEWVATISSGGSYTYYPDSVEGRFTISRDNAKNTLCLQMSGLRSEDAAMYYCARGSDGNFPFGYWGQGTLVTVSA

^^^^^^^^ ^^^^^^^^ ^^^^^^^^^^^^

Therapeutic : Motavizumab

Best Alignment of Motavizumab heavy chain to a sequence from OAS

QVTLRESGPALVKPTQTLTLTCTFSGFSLSTAGMSVGWIRQPPGKALEWLADIWWDDKKHYNPSLKDRLTISKDTSKNQVVLKVTNMDPADTATYYCARDMIF-NFYFDVWGQGTTVTVSS

|||||||||||||||||||||||||||||||.||||.||||||||||||||.|.|||.|.||.|||.|||||||||||||||..|||||.|||||||||.......|||.|||||.|||||

QVTLRESGPALVKPTQTLTLTCTFSGFSLSTSGMSVSWIRQPPGKALEWLALIDWDDAKYYNTSLKTRLTISKDTSKNQVVLTMTNMDPVDTATYYCARSYGGRTYYFDYWGQGTLVTVSS

^^^^^^^^^^ ^^^^^^^ ^^^^^^^^^^^^^

Best Alignment of Motavizumab light chain to a sequence from OAS

DIQMTQSPSTLSASVGDRVTITCSASSRV-GYMHWYQQKPGKAPKLLIYDTSKLASGVPSRFSGSGSGTEFTLTISSLQPDDFATYYCFQGSGYPFTFGGGTKVEIK

|||||||||||||||||||||||.||.|......||||||||||||||||.|.|||||||||||||||||||||||||||||||||||.|..|||.|||.|||||||

DIQMTQSPSTLSASVGDRVTITCRASQRFTTWLAWYQQKPGKAPKLLIYDVSSLASGVPSRFSGSGSGTEFTLTISSLQPDDFATYYCQQYDGYPWTFGQGTKVEIK

^^^^^^ ^^^ ^^^^^^^^^

Best Alignment of Motavizumab heavy chain CDRs to a sequence from OAS

QVTLRESGPALVKPTQTLTLTCTFSGFSLSTAGMSVGWIRQPPGKALEWLADIWWDDKKHYNPSLKDRLTISKDTSKNQVVLKVTNMDPADTATYYCARDMIFNFYFDVWGQGTTVTVSS

||||.||||..|.|.|||.|||..|||||||.||.|||||||.||.|||||.|||||.|.||..||..||||||||||||.||....|.||||||||||...|.|||||||.||||||||

QVTLKESGPGIVQPSQTLGLTCSCSGFSLSTSGMGVGWIRQPSGKGLEWLAHIWWDDDKYYNTALKSGLTISKDTSKNQVFLKIASVDTADTATYYCARISLFIFYFDVWGAGTTVTVSS

^^^^^^^^^^ ^^^^^^^ ^^^^^^^^^^^^

Best Alignment of Motavizumab light chain CDRs to a sequence from OAS

DIQMTQSPSTLSASVGDRVTITCSASSRVGYMHWYQQKPGKAPKLLIYDTSKLASGVPSRFSGSGSGTEFTLTISSLQPDDFATYYCFQGSGYPFTFGGGTKVEIK

....||||...|||.|..||.||||||||.||||||||....|||.|||||.|||||..||||||||....|||||....|.||||||||||||.|||||||.|||

ENVLTQSPAIMSASPGEKVTMTCSASSRVSYMHWYQQKSSTSPKLWIYDTSILASGVTGRFSGSGSGNSYSLTISSMEAEDVATYYCFQGSGYPYTFGGGTKLEIK

^^^^^ ^^^ ^^^^^^^^^

Best Alignment of Motavizumab CDR-H3 to a sequence from OAS

QVTLRESGPALVKPTQTLTLTCTFSGFSLSTAGMSVGWIRQPPGKALEWLADIWWD-DKKHYNPSLKDRLTISKDTSKNQVVLKVTNMDPADTATYYCARDMIFNFYFDVWGQGTTVTVSS

||.|..||..||.|.......|..||...........|..|.|...|||...|........||...|...|...|.|..............|.|.|.||||.|||.||||||.||||||||

QVQLQQSGAELVRPGASVKMSCKASGYTF--TSYNMHWVKQTPRQGLEWIGAIYPGNGDTSYNQKFKGKATLTVDKSSSTAYMQLSSLTSEDSAVYFCARDGIFNWYFDVWGTGTTVTVSS

^^^^^^^^^^ ^^^^^^^^ ^^^^^^^^^^^^

Therapeutic : Muromonab

Best Alignment of Muromonab heavy chain to a sequence from OAS

QVQLQQSGAELARPGASVKMSCKASGYTFTRYTMHWVKQRPGQGLEWIGYINPSRGYTNYNQKFKDKATLTTDKSSSTAYMQLSSLTSEDSAVYYCARYYDDHYCLDYWGQGTTLTVSS

||||||||||||||||||||||||||||||.|||||||||||||||||||||||.||||||||||||||||.||||||||||||||||||||||||||.||..|.||||||||||||||

QVQLQQSGAELARPGASVKMSCKASGYTFTSYTMHWVKQRPGQGLEWIGYINPSSGYTNYNQKFKDKATLTADKSSSTAYMQLSSLTSEDSAVYYCARPYDG-YYLDYWGQGTTLTVSS

^^^^^^^^ ^^^^^^^^ ^^^^^^^^^^^^

Best Alignment of Muromonab light chain to a sequence from OAS

QIVLTQSPAIMSASPGEKVTMTCSASSSVSYMNWYQQKSGTSPKRWIYDTSKLASGVPAHFRGSGSGTSYSLTISGMEAEDAATYYCQQWSSNPFTFGSGTKLEIK

|||||||||||||||||||||||||||||||||||||||||||||||||||||||||||.|.|||||||||||||.||||||||||||||||||||||||||||||

QIVLTQSPAIMSASPGEKVTMTCSASSSVSYMNWYQQKSGTSPKRWIYDTSKLASGVPARFSGSGSGTSYSLTISSMEAEDAATYYCQQWSSNPFTFGSGTKLEIK

^^^^^ ^^^ ^^^^^^^^^

Best Alignment of Muromonab heavy chain CDRs to a sequence from OAS

QVQLQQSGAELARPGASVKMSCKASGYTFTRYTMHWVKQRPGQGLEWIGYINPSRGYTNYNQKFKDKATLTTDKSSSTAYMQLSSLTSEDSAVYYCARYYDDHYCLDYWGQGTTLTVSS

|.|||.|..|||||||||.||||||||||||||||||.||||||||||||||||.|||.||||.|||.|||.||||||||||||||||||.||||||||||..|..||||||||.|..|

QLQLQHSASELARPGASVQMSCKASGYTFTRYTMHWVQQRPGQGLEWIGYINPSSGYTEYNQKLKDKTTLTADKSSSTAYMQLSSLTSEDYAVYYCARYYDYDYAMDYWGQGTTDTDTS

^^^^^^^^ ^^^^^^^^ ^^^^^^^^^^^^

Best Alignment of Muromonab light chain CDRs to a sequence from OAS

QIVLTQSPAIMSASPGEKVTMTCSASSSVSYMNWYQQKSGTSPKRWIYDTSKLASGVPAHFRGSGSGTSYSLTISGMEAEDAATYYCQQWSSNPFTFGSGTKLEIK

||||||||||||||||||||||||||||||||.||||||||||||||||||||||||||.|.|||||||||||||.||||||||||||||||||||||.|||||.|

QIVLTQSPAIMSASPGEKVTMTCSASSSVSYMHWYQQKSGTSPKRWIYDTSKLASGVPARFSGSGSGTSYSLTISSMEAEDAATYYCQQWSSNPFTFGAGTKLELK

^^^^^ ^^^ ^^^^^^^^^

Best Alignment of Muromonab CDR-H3 to a sequence from OAS

QVQLQQSGAELARPGASVKMSCKASGYTFTRYTMHWVKQRPGQGLEWIGYINPS--RGYTNYNQKFKDKATLTTDKSSSTAYMQLSSLTSEDSAVYYCARYYDDHYCLDYWGQGTTLTVSS

.|....||..|..||.|...||.|||.|||.|.|.||.|.||..|||.|.|........|.|....|...|...|.|.|..|.|...|..||||.||||||.||.||||||||||||||||

EVKVVESGGGLVQPGGSLSLSCAASGFTFTDYYMSWVRQPPGKALEWLGFIRNKANGYTTEYSASVKGRFTISRDNSQSILYLQMNALRAEDSATYYCARYLDDGYCLDYWGQGTTLTVSS

^^^^^^^^ ^^^^^^^^^^ ^^^^^^^^^^^^

Therapeutic : Natalizumab

Best Alignment of Natalizumab heavy chain to a sequence from OAS

QVQLVQSGAEVKKPGASVKVSCKASGFNIKDTYIHWVRQAPGQRLEWMGRIDPANGYTKYDPKFQGRVTITADTSASTAYMELSSLRSEDTAVYYCAREGYYGN-YGVYAMDYWGQGTLVTVSS

||||||||||||||||||||||||||........|||||||||||||||.|...||.|||..|||||||||.||||||||||||||||||||||||||||||....|.|..|||||||||||||

QVQLVQSGAEVKKPGASVKVSCKASGYTFTSYAMHWVRQAPGQRLEWMGWINAGNGNTKYSQKFQGRVTITRDTSASTAYMELSSLRSEDTAVYYCAREGYYYDSSGYYPLDYWGQGTLVTVSS

^^^^^^^^ ^^^^^^^^ ^^^^^^^^^^^^^^^^^

Best Alignment of Natalizumab light chain to a sequence from OAS

DIQMTQSPSSLSASVGDRVTITCKTSQDINKYMAWYQQTPGKAPRLLIHYTSALQPGIPSRFSGSGSGRDYTFTISSLQPEDIATYYCLQYDNLWTFGQGTKVEIK

||||||||||||||.|..|||||||||||||||||||..|||.|||||||||.||||||||||||||||||.|.||.|.|||||||||||||||||||.|||.|||

DIQMTQSPSSLSASLGGKVTITCKTSQDINKYMAWYQHKPGKGPRLLIHYTSTLQPGIPSRFSGSGSGRDYSFSISNLEPEDIATYYCLQYDNLWTFGGGTKLEIK

^^^^^^ ^^^ ^^^^^^^^

Best Alignment of Natalizumab heavy chain CDRs to a sequence from OAS

QVQLVQSGAEVKKPGASVKVSCKASGFNIKDTYIHWVRQAPGQRLEWMGRIDPANGYTKYDPKFQGRVTITADTSASTAYMELSSLRSEDTAVYYCAREGYYGNYGVYAMDYWGQGTLVTVSS

.|||.|||||..|||||||.||.||||||||||.|||.|.|.|.|||.||||||||.|||.|||||..|||||||..|||..||||.|||||.|||||.|||||||.||||||||||.|||||

-VQLQQSGAELVKPGASVKLSCTASGFNIKDTYMHWVKQRPEQGLEWIGRIDPANGNTKYAPKFQGKATITADTSSNTAYLQLSSLTSEDTAIYYCARTGYYGNYGGYAMDYWGQGTSVTVSS

^^^^^^^^ ^^^^^^^^ ^^^^^^^^^^^^^^^^

Best Alignment of Natalizumab light chain CDRs to a sequence from OAS

DIQMTQSPSSLSASVGDRVTITCKTSQDINKYMAWYQQTPGKAPRLLIHYTSALQPGIPSRFSGSGSGRDYTFTISSLQPEDIATYYCLQYDNLWTFGQGTKVEIK

.|||.||||||.||.|..||||||.|||||||.||||..|||.|||||||||.||||||||||||||||||.|.||.|.|||||||||||||||||||.|||.|||

-IQMIQSPSSLPASLGGKVTITCKASQDINKYIAWYQHKPGKGPRLLIHYTSTLQPGIPSRFSGSGSGRDYSFSISNLEPEDIATYYCLQYDNLWTFGGGTKLEIK

^^^^^^ ^^^ ^^^^^^^^

Best Alignment of Natalizumab CDR-H3 to a sequence from OAS

QVQLVQSGAEVKKPGASVKVSCKASGFNIKDTYIHWVRQAPGQRLEWMGRIDPANGYTKYDPKFQGRVTITADTSASTAYMELSSLRSEDTAVYYCAREGYYGNYGVYAMDYWGQGTLVTVSS

||||..||.....|..|....|..|||........||||.||..|||.|.|....|.|.|......|..|..|.|.|.......||...|||.||||||||||||||||||||||||.|||||

QVQLKESGPGLVAPSQSLSITCTVSGFSLTGYGVNWVRQPPGKGLEWLGMIWGD-GSTDYNSALKSRLSISKDNSKSQVFLKMNSLQTDDTARYYCAREGYYGNYGVYAMDYWGQGTSVTVSS

^^^^^^^^ ^^^^^^^^ ^^^^^^^^^^^^^^^^

Therapeutic : Necitumumab

Best Alignment of Necitumumab heavy chain to a sequence from OAS

QVQLQESGPGLVKPSQTLSLTCTVSGGSISSGDYYWSWIRQPPGKGLEWIGYIYYSGSTDYNPSLKSRVTMSVDTSKNQFSLKVNSVTAADTAVYYCARVSIFGVGTFDYWGQGTLVTVSS

|||||||||||||||||||||||||||||||||||||||||||||||||||||||||||.||||||||||.||||||||||||..||||||||||||||..||||.|||||||||||||||

QVQLQESGPGLVKPSQTLSLTCTVSGGSISSGDYYWSWIRQPPGKGLEWIGYIYYSGSTYYNPSLKSRVTISVDTSKNQFSLKLSSVTAADTAVYYCARATIFGVDTFDYWGQGTLVTVSS

^^^^^^^^^^ ^^^^^^^ ^^^^^^^^^^^^^

Best Alignment of Necitumumab light chain to a sequence from OAS

EIVMTQSPATLSLSPGERATLSCRASQSVSSYLAWYQQKPGQAPRLLIYDASNRATGIPARFSGSGSGTDFTLTISSLEPEDFAVYYCHQYGS-TPLTFGGGTKAEIK

|||.||||||||||||||||||||||||||||||||||||||||||||||||||||||||||||||||||||||||||||||||||||.||||..|||||||||.|||

EIVLTQSPATLSLSPGERATLSCRASQSVSSYLAWYQQKPGQAPRLLIYDASNRATGIPARFSGSGSGTDFTLTISSLEPEDFAVYYCQQYGSSPPLTFGGGTKVEIK

^^^^^^ ^^^ ^^^^^^^^^^

Best Alignment of Necitumumab heavy chain CDRs to a sequence from OAS

QVQLQESGPGLVKPSQTLSLTCTVSGGSISSGDYYWSWIRQPPGKGLEWIGYIYYSGSTDYNPSLKSRVTMSVDTSKNQFSLKVNSVTAADTAVYYCARVSIFGVGTFDYWGQGTLVTVSS

..............|.||||||||||||||||.||||||||.|||||||||||||||||.||||||||||.||||||||||||..|||||||||||||||.||||||||||||||||||||

--------------SETLSLTCTVSGGSISSGGYYWSWIRQHPGKGLEWIGYIYYSGSTYYNPSLKSRVTISVDTSKNQFSLKLSSVTAADTAVYYCARVTIFGVGTFDYWGQGTLVTVSS

^^^^^^^^^^ ^^^^^^^ ^^^^^^^^^^^^^

Best Alignment of Necitumumab light chain CDRs to a sequence from OAS

EIVMTQSPATLSLSPGERATLSCRASQSVSSYLAWYQQKPGQAPRLLIYDASNRATGIPARFSGSGSGTDFTLTISSLEPEDFAVYYCHQYGSTPLTFGGGTKAEIK

|||.|||||||||||||||||||||||||||||||||||||||||||||||||||||||.||||.|||||||||||.|||||||||||.||||||||||||||.|||

EIVLTQSPATLSLSPGERATLSCRASQSVSSYLAWYQQKPGQAPRLLIYDASNRATGIPDRFSGGGSGTDFTLTISRLEPEDFAVYYCQQYGSTPLTFGGGTKVEIK

^^^^^^ ^^^ ^^^^^^^^^

Best Alignment of Necitumumab CDR-H3 to a sequence from OAS

QVQLQESGPGLVKPSQTLSLTCTVSGGSISSGDYYWSWIRQPPGKGLEWIGYIYYSGSTDYNPSLKSRVTMSVDTSKNQFSLKVNSVTAADTAVYYCARVSIFGVGTFDYWGQGTLVTVSS

.............||.|||||||||||||....||||||||||||||||||||||||||.||||||||||.||||||||||||..||||||||||||||||||||.|||||||||||||||

-------------PSETLSLTCTVSGGSI--SSYYWSWIRQPPGKGLEWIGYIYYSGSTNYNPSLKSRVTISVDTSKNQFSLKLSSVTAADTAVYYCARVSIFGVVTFDYWGQGTLVTVSS

^^^^^^^^^^ ^^^^^^^ ^^^^^^^^^^^^^

Therapeutic : Nimotuzumab

Best Alignment of Nimotuzumab heavy chain to a sequence from OAS

QVQLQQSGAEVKKPGSSVKVSCKASGYTFTNYYIYWVRQAPGQGLEWIGGINPTSGGSNFNEKFKTRVTITADESSTTAYMELSSLRSEDTAFYFCTRQGLWFD----SDGRGFDFWGQGTTVTVSS

||||.||||||||||.||||||||||||||.|||.||||||||||||.|.|||.|||.|...||..|||||||||..|||||||||||||||.|.|.|...|......|..|.||.|||||.|||||

QVQLVQSGAEVKKPGASVKVSCKASGYTFTGYYIHWVRQAPGQGLEWMGRINPNSGGTNYAQKFQGRVTITADESTSTAYMELSSLRSEDTAVYYCARGRCWGRYYDSSGYRAFDIWGQGTMVTVSS

^^^^^^^^ ^^^^^^^^ ^^^^^^^^^^^^^^^^^^^^

Best Alignment of Nimotuzumab light chain to a sequence from OAS

DIQMTQSPSSLSASVGDRVTITCRSSQNIVHSNGNTYLDWYQQTPGKAPKLLIYKVSNRFSGVPSRFSGSGSGTDFTFTISSLQPEDIATYYCFQYSH---VPWTFGQGTKLQIT

||||||||||||||||||||||||.||||......|||.||||.||||||||||..||..|||||||||||||||||||||||||||||||||.||......|.||||||.|.|.

DIQMTQSPSSLSASVGDRVTITCRASQNI-----ATYLNWYQQKPGKAPKLLIYVASNLHSGVPSRFSGSGSGTDFTFTISSLQPEDIATYYCQQYDNLPRPPITFGQGTRLEIK

^^^^^^^^^^^ ^^^ ^^^^^^^^^^^^

Best Alignment of Nimotuzumab heavy chain CDRs to a sequence from OAS

QVQLQQSGAEVKKPGSSVKVSCKASGYTFTNYYIYWVRQAPGQGLEWIGGINPTSGGSNFNEKFKTRVTITADESSTTAYMELSSLRSEDTAFYFCTRQGLWFDSDGRGFDFWGQGTTVTVSS

||||.||||||||||.||||||||||||||.||..||||||||||||.|.|||.|||.|...||..|||.|.|.|..|||||||.|||.|||.|.|.|....|||.|||||.|||||.|||||

QVQLVQSGAEVKKPGASVKVSCKASGYTFTGYYMHWVRQAPGQGLEWMGWINPNSGGTNYAQKFQGRVTMTRDTSISTAYMELSRLRSDDTAVYYCARDTPPFDSSGRGFDYWGQGTLVTVSS

^^^^^^^^ ^^^^^^^^ ^^^^^^^^^^^^^^^^

Best Alignment of Nimotuzumab light chain CDRs to a sequence from OAS

DIQMTQSPSSLSASVGDRVTITCRSSQNIVHSNGNTYLDWYQQTPGKAPKLLIYKVSNRFSGVPSRFSGSGSGTDFTFTISSLQPEDIATYYCFQYSHVPWTFGQGTKLQIT

|..|||.|.||..|.||...|.||||||||||||||||.||.|.||..||||||||||||||||.||||||||||||..||....||...|||||.||||||||.||||.|.

DVLMTQTPLSLPVSLGDQASISCRSSQNIVHSNGNTYLEWYLQKPGQSPKLLIYKVSNRFSGVPDRFSGSGSGTDFTLKISRVEAEDLGVYYCFQGSHVPWTFGGGTKLEIK

^^^^^^^^^^^ ^^^ ^^^^^^^^^

Best Alignment of Nimotuzumab CDR-H3 to a sequence from OAS

QVQLQQSGAEVKKPGSSVKVSCKASGYTFTNYYIYWVRQAPGQGLEWIGGINPTSGGSNFNEKFKTRVTITADESSTTAYMELSSLRSEDTAFYFCTRQGLWFDSDGRGFDFWGQGTTVTVSS

.|||||.||...||.......|..||..|.|....|.||.||.||||||.......|.........|.||..|.........|.|....|||.|.|||.|||...|.||||.|||||.|||||

-VQLQQWGAGLLKPSETLSLTCAVSGGSFSNSLWTWIRQPPGKGLEWIGDVSLD-RGTKNVPSLQSRITISMDRTKNQFSLRLKSVTAADTATYYCTRLGLWGYYDARGFDYWGQGTLVTVSS

^^^^^^^^ ^^^^^^^^ ^^^^^^^^^^^^^^^^

Therapeutic : Nivolumab

Best Alignment of Nivolumab heavy chain to a sequence from OAS

QVQLVESGGGVVQPGRSLRLDCKASGITFSNSGMHWVRQAPGKGLEWVAVIWYDGSKRYYADSVKGRFTISRDNSKNTLFLQMNSLRAEDTAVYYCATN-------DDYWGQGTLVTVSS

||||||||||||||||||||.|.|||.||.|.|||||||||||||||||||||||||.||||||.|||||||||||||||||||||||||||||||||.........|||||||||||||

QVQLVESGGGVVQPGRSLRLSCVASGFTFRNYGMHWVRQAPGKGLEWVAVIWYDGSKKYYADSVGGRFTISRDNSKNTLFLQMNSLRAEDTAVYYCATVSKSSSWYTDYWGQGTLVTVSS

^^^^^^^^ ^^^^^^^^ ^^^^^^^^^^^^^

Best Alignment of Nivolumab light chain to a sequence from OAS

EIVLTQSPATLSLSPGERATLSCRASQSVSSYLAWYQQKPGQAPRLLIYDASNRATGIPARFSGSGSGTDFTLTISSLEPEDFAVYYCQQSSNWPRTFGQGTKVEIK

|||||||||||||||||||||||||||||||||||||||||||||||||||||||||||||||||||||||||||||||||||||||||||||||||||||||||||

EIVLTQSPATLSLSPGERATLSCRASQSVSSYLAWYQQKPGQAPRLLIYDASNRATGIPARFSGSGSGTDFTLTISSLEPEDFAVYYCQQSSNWPRTFGQGTKVEIK

^^^^^^ ^^^ ^^^^^^^^^

Best Alignment of Nivolumab heavy chain CDRs to a sequence from OAS

QVQLVESGGGVVQPGRSLRLDCKASGITFSNSGMHWVRQAPGKGLEWVAVIWYDGSKRYYADSVKGRFTISRDNSKNTLFLQMNSLRAEDTAVYYCATNDDYWGQGTLVTVSS

................||||.|.||||||||.||||||||||||||||||||||||.||||||||||||||||||||||.||||.|||||||||||..|.|||||||||||||

---------------GSLRLSCAASGITFSNYGMHWVRQAPGKGLEWVAVIWYDGSNRYYADSVKGRFTISRDNSKNTLYLQMNGLRAEDTAVYYCTSNFDYWGQGTLVTVSS

^^^^^^^^ ^^^^^^^^ ^^^^^^

Best Alignment of Nivolumab light chain CDRs to a sequence from OAS

EIVLTQSPATLSLSPGERATLSCRASQSVSSYLAWYQQKPGQAPRLLIYDASNRATGIPARFSGSGSGTDFTLTISSLEPEDFAVYYCQQSSNWPRTFGQGTKVEIK

||||.||||||||||||||||||||||||||||||||||||||||||||||||||||||||.|||||||.||||||||||||||||||||||||||||||||.||||

EIVLAQSPATLSLSPGERATLSCRASQSVSSYLAWYQQKPGQAPRLLIYDASNRATGIPARVSGSGSGTEFTLTISSLEPEDFAVYYCQQSSNWPRTFGQGTRVEIK

^^^^^^ ^^^ ^^^^^^^^^

Best Alignment of Nivolumab CDR-H3 to a sequence from OAS

QVQLVESGGGVVQPGRSLRLDCKASGITFSNSGMHWVRQAPGKGLEWVAVIWYDGSKRYYADSVKGRFTISRDNSKNTLFLQMNSLRAEDTAVYYCATNDDYWGQGTLVTVSS

.|||..||...|.||.|....|||||.||....|.||.|..||.|||...|........|....||..|...|.|..|......||..||.||||||||||||||||..||||

-VQLQQSGPELVKPGASVKISCKASGYTFTDYYMNWVKQSHGKSLEWIGDINPNNGGTSYNQKFKGKATLTVDKSSSTAYMELRSLTSEDSAVYYCATNDDYWGQGTTLTVSS

^^^^^^^^ ^^^^^^^^ ^^^^^^

Therapeutic : Obiltoxaximab

Best Alignment of Obiltoxaximab heavy chain to a sequence from OAS

QVQLQQSGPELKKPGASVKVSCKDSGYAFSSSWMNWVRQAPGQGLEWIGRIYPGDGDTNYNGKFQGRVTITADKSSSTAYMELSSLRSEDTAVYFCARSGLL---RYAMDYWGQGTLVTVSS

|||||||||||.|||||||.|||.|||||||||||||.|.||.|||||||||||||||||||||.|..|.|||||||||||.||||.|||.|||||||||||...|||||||||||.|||||

QVQLQQSGPELVKPGASVKISCKASGYAFSSSWMNWVKQRPGKGLEWIGRIYPGDGDTNYNGKFKGKATLTADKSSSTAYMQLSSLTSEDSAVYFCARSGLLLRSRYAMDYWGQGTSVTVSS

^^^^^^^^ ^^^^^^^^ ^^^^^^^^^^^^^^^

Best Alignment of Obiltoxaximab light chain to a sequence from OAS

DIQMTQSPSSLSASVGDRVTITCRASQDIRNYLNWYQQKPGKAVKLLIYYTSRLLPGVPSRFSGSGSGTDYSLTISSQEQEDIGTYFCQQGNTLPWTFGQGTKVEIR

||||||..||||||.||||||.|||||||.||||||||||..||||||||||||..||||||||||||||||||||..|||||.|||||||||||||||.|||.||.

DIQMTQITSSLSASLGDRVTISCRASQDISNYLNWYQQKPDGAVKLLIYYTSRLHSGVPSRFSGSGSGTDYSLTISNLEQEDIATYFCQQGNTLPWTFGGGTKLEIK

^^^^^^ ^^^ ^^^^^^^^^

Best Alignment of Obiltoxaximab heavy chain CDRs to a sequence from OAS

QVQLQQSGPELKKPGASVKVSCKDSGYAFSSSWMNWVRQAPGQGLEWIGRIYPGDGDTNYNGKFQGRVTITADKSSSTAYMELSSLRSEDTAVYFCARSGLLRYAMDYWGQGTLVTVSS

||||..|||||.|||||||.|||.||||||||||.||.|.||||||||||||||||||||||||.|..|.||||||.||||.||||.|||.||||.|||||||||||||||||.|||||

QVQLKESGPELVKPGASVKISCKASGYAFSSSWMHWVKQRPGQGLEWIGRIYPGDGDTNYNGKFKGKATLTADKSSNTAYMPLSSLTSEDSAVYFFARSGLLRYAMDYWGQGTSVTVSS

^^^^^^^^ ^^^^^^^^ ^^^^^^^^^^^^

Best Alignment of Obiltoxaximab light chain CDRs to a sequence from OAS

DIQMTQSPSSLSASVGDRVTITCRASQDIRNYLNWYQQKPGKAVKLLIYYTSRLLPGVPSRFSGSGSGTDYSLTISSQEQEDIGTYFCQQGNTLPWTFGQGTKVEIR

|||.||..||||||.||||||.||||||||||||||||||...|||||||||||..||||||||||||||||||||..|||||.||||||||||||||..|||.|..

DIQLTQTTSSLSASLGDRVTISCRASQDIRNYLNWYQQKPDGTVKLLIYYTSRLHSGVPSRFSGSGSGTDYSLTISNLEQEDIATYFCQQGNTLPWTFSGGTKLEVK

^^^^^^ ^^^ ^^^^^^^^^

Best Alignment of Obiltoxaximab CDR-H3 to a sequence from OAS

QVQLQQSGPELKKPGASVKVSCKDSGYAFSSSWMNWVRQAPGQGLEWIGRIYPGDGDTNYNGKFQGRVTITADKSSSTAYMELSSLRSEDTAVYFCARSGLLRYAMDYWGQGTLVTVSS

.|||..||..|..||.|.|.||..||..|||..|.||||||..||||......|.....|.....||.||..|....|......||||||||.|.||||||||||||||||||.|||||

EVQLVESGGGLVQPGGSRKLSCAASGFTFSSFGMHWVRQAPEKGLEWVAYVSSGSSTIYYADTVKGRFTISRDNPKNTLFLQMTSLRSEDTAMYYCARSGLLRYAMDYWGQGTSVTVSS

^^^^^^^^ ^^^^^^^^ ^^^^^^^^^^^^

Therapeutic : Obinutuzumab

Best Alignment of Obinutuzumab heavy chain to a sequence from OAS

QVQLVQSGAEVKKPGSSVKVSCKASGYAFSYSWINWVRQAPGQGLEWMGRIFPGDGDTDYNGKFKGRVTITADKSTSTAYMELSSLRSEDTAVYYCARNVFD--GYWLVYWGQGTLVTVSS

||||||||||||||||||||||||||..|....||||||||||||||||||.|..|.|.|..||.|||||||||||||||||||||||||||||||||..||..||...||||||||||||

QVQLVQSGAEVKKPGSSVKVSCKASGGTF-RYAINWVRQAPGQGLEWMGRIIPIFGTTNYAQKFQGRVTITADKSTSTAYMELSSLRSEDTAVYYCARGPFDNSGYYSFYWGQGTLVTVSS

^^^^^^^^ ^^^^^^^^ ^^^^^^^^^^^^^^

Best Alignment of Obinutuzumab light chain to a sequence from OAS

DIVMTQTPLSLPVTPGEPASISCRSSKSLLHSNGITYLYWYLQKPGQSPQLLIYQMSNLVSGVPDRFSGSGSGTDFTLKISRVEAEDVGVYYCAQNLELPYTFGGGTKVEIK

||||||...|.|||.|..|||||||||||||||||||||||||||||||||||||||||.||||||||||||||||||.|||||||||||||||||||||||||||||.|||

DIVMTQAAFSNPVTLGTSASISCRSSKSLLHSNGITYLYWYLQKPGQSPQLLIYQMSNLASGVPDRFSGSGSGTDFTLRISRVEAEDVGVYYCAQNLELPYTFGGGTKLEIK

^^^^^^^^^^^ ^^^ ^^^^^^^^^

Best Alignment of Obinutuzumab heavy chain CDRs to a sequence from OAS

QVQLVQSGAEVKKPGSSVKVSCKASGYAFSYSWINWVRQAPGQGLEWMGRIFPGDGDTDYNGKFKGRVTITADKSTSTAYMELSSLRSEDTAVYYCARNVFDGYWLVYWGQGTLVTVSS

||||.|||.|..|||.|||.||||||||||.||.|||.|.|||||||.|||.||||||.|||||||..|.|||||.|||||.||||.|.|.|||.|||.|.||.|.||||||||||||.

QVQLQQSGPELVKPGASVKISCKASGYAFSSSWMNWVKQRPGQGLEWIGRIYPGDGDTNYNGKFKGKATLTADKSSSTAYMHLSSLTSVDSAVYFCARCVYDGSWFVYWGQGTLVTVSA

^^^^^^^^ ^^^^^^^^ ^^^^^^^^^^^^

Best Alignment of Obinutuzumab light chain CDRs to a sequence from OAS

DIVMTQTPLSLPVTPGEPASISCRSSKSLLHSNGITYLYWYLQKPGQSPQLLIYQMSNLVSGVPDRFSGSGSGTDFTLKISRVEAEDVGVYYCAQNLELPYTFGGGTKVEIK

.|.||......||.....|||||.|||||||||||||||||||||||||||||||||||.||||||||.|||||||||.|||||||||||||||||||||||||||||.|||

-IAMTPAACCSPVSLLPSASISCGSSKSLLHSNGITYLYWYLQKPGQSPQLLIYQMSNLASGVPDRFSSSGSGTDFTLRISRVEAEDVGVYYCAQNLELPYTFGGGTKLEIK

^^^^^^^^^^^ ^^^ ^^^^^^^^^

Best Alignment of Obinutuzumab CDR-H3 to a sequence from OAS

QVQLVQSGAEVKKPGSSVKVSCKASGYAFSYSWINWVRQAPGQGLEWMGRIFPGDGDTDYNGKFKGRVTITADKSTSTAYMELSSLRSEDTAVYYCARNVFDGYWLVYWGQGTLVTVSS

.|||.|||....|||.|||.|||||||.|.....|||.|..|..|||.|.|.|..|.|..|.||||..|.|.|||.|||||||.||.|||.||.||||||||||.|.||||||..||||

EVQLQQSGPVLVKPGASVKMSCKASGYTFTDYYMNWVKQSHGKSLEWIGVINPYNGGTSSNQKFKGKATLTVDKSSSTAYMELNSLKSEDSAVFYCARNVFDGYYLDYWGQGTTLTVSS

^^^^^^^^ ^^^^^^^^ ^^^^^^^^^^^^

Therapeutic : Ocrelizumab

Best Alignment of Ocrelizumab heavy chain to a sequence from OAS

EVQLVESGGGLVQPGGSLRLSCAASGYTFTSYNMHWVRQAPGKGLEWVGAIYPGNGDTSYNQKFKGRFTISVDKSKNTLYLQMNSLRAEDTAVYYCARVVYYSN--SYWYFDVWGQGTLVTVSS

||||||||||||||||||||||||||.||.||.|||||||||||||||..||.|.|.|.|....|||||||.|.|||||||||||||||||||||||||||..|.....|||.|||||||||||

EVQLVESGGGLVQPGGSLRLSCAASGFTFSSYAMHWVRQAPGKGLEWVSVIYSG-GSTYYADSVKGRFTISRDNSKNTLYLQMNSLRAEDTAVYYCARVVYGGNVFPSYYFDYWGQGTLVTVSS

^^^^^^^^ ^^^^^^^^ ^^^^^^^^^^^^^^^^^

Best Alignment of Ocrelizumab light chain to a sequence from OAS

DIQMTQSPSSLSASVGDRVTITCRASSSV-SYMHWYQQKPGKAPKPLIYAPSNLASGVPSRFSGSGSGTDFTLTISSLQPEDFATYYCQQWSFNPPTFGQGTKVEIK

||||||||||||||||||||.|||||.|..||..|||||||||||.||||.|||.|||||||||||||||||||||||||||||||||||...||||||||||||||

DIQMTQSPSSLSASVGDRVTFTCRASQSIISYLNWYQQKPGKAPKLLIYAASNLQSGVPSRFSGSGSGTDFTLTISSLQPEDFATYYCQQSYSNPPTFGQGTKVEIK

^^^^^^ ^^^ ^^^^^^^^^

Best Alignment of Ocrelizumab heavy chain CDRs to a sequence from OAS

EVQLVESGGGLVQPGGSLRLSCAASGYTFTSYNMHWVRQAPGKGLEWVGAIYPGNGDTSYNQKFKGRFTISVDKSKNTLYLQMNSLRAEDTAVYYCARVVYYSNSYWYFDVWGQGTLVTVSS

...|..||..||.||.|...||.||||||||||||||.|.|..||||.||||||||||||||||||..|..||||..|.|.|..||..||.|||.|||..|||||||||||||.||.|||||

---LQQSGAELVRPGASVKMSCKASGYTFTSYNMHWVKQTPRQGLEWIGAIYPGNGDTSYNQKFKGKATLTVDKSSSTAYMQLSSLTSEDSAVYFCARSDYYSNSYWYFDVWGTGTTVTVSS

^^^^^^^^ ^^^^^^^^ ^^^^^^^^^^^^^^^

Best Alignment of Ocrelizumab light chain CDRs to a sequence from OAS

DIQMTQSPSSLSASVGDRVTITCRASSSVSYMHWYQQKPGKAPKPLIYAPSNLASGVPSRFSGSGSGTDFTLTISSLQPEDFATYYCQQWSFNPPTFGQGTKVEIK

.|..||||...|||.|..||.||.||||||||||||||.|..|...|||||..|.|||.|||||||||...|||||...||.|||||||||.||||||.|||.|.|

QIVLTQSPAIMSASPGEKVTMTCSASSSVSYMHWYQQKSGPSPTSWIYAPSQRACGVPARFSGSGSGTSYSLTISSMEAEDAATYYCQQWSSNPPTFGAGTKLELK

^^^^^ ^^^ ^^^^^^^^^

Best Alignment of Ocrelizumab CDR-H3 to a sequence from OAS

EVQLVESGGGLVQPGGSLRLSCAASGYTFTSYNMHWVRQAPGKGLEWVGAIYPGNGDTSYNQKFKGRFTISVDKSKNTLYLQMNSLRAEDTAVYYCARVVYYSNSYWYFDVWGQGTLVTVSS

.|||..||..||.||.|...||.||||.|.|..|.||.|.||.||||.|.||||.|||.||.|||...|...|||..|.|.|..||..||.|||.|||.||||||||||||||.||.||||.

QVQLQQSGPELVKPGASVKISCKASGYAFSSSWMNWVKQRPGQGLEWIGRIYPGDGDTNYNGKFKCKATLTADKSSSTAYMQRSSLTSEDSAVYFCARSVYYSNSYWYFDVWGTGTTVTVSP

^^^^^^^^ ^^^^^^^^ ^^^^^^^^^^^^^^^

Therapeutic : Ofatumumab

Best Alignment of Ofatumumab heavy chain to a sequence from OAS

EVQLVESGGGLVQPGRSLRLSCAASGFTFNDYAMHWVRQAPGKGLEWVSTISWNSGSIGYADSVKGRFTISRDNAKKSLYLQMNSLRAEDTALYYCAKDIQYGN-------YYYGMDVWGQGTTVTVSS

|||||||||||||||||||||||||||||.|||||||||||||||||||.||||||||||||||||||||||||||.|||||||||||||||||||||||.|.........||||||||||||||||||

EVQLVESGGGLVQPGRSLRLSCAASGFTFDDYAMHWVRQAPGKGLEWVSGISWNSGSIGYADSVKGRFTISRDNAKNSLYLQMNSLRAEDTALYYCAKDIGYCSSTSCPRPYYYGMDVWGQGTTVTVSS

^^^^^^^^ ^^^^^^^^ ^^^^^^^^^^^^^^^^^^^^^^

Best Alignment of Ofatumumab light chain to a sequence from OAS

EIVLTQSPATLSLSPGERATLSCRASQSVSSYLAWYQQKPGQAPRLLIYDASNRATGIPARFSGSGSGTDFTLTISSLEPEDFAVYYCQQRSNWPITFGQGTRLEIK

|||||||||||||||||||||||||||||||||||||||||||||||||||||||||||||||||||||||||||||||||||||||||||||||||||||||||||

EIVLTQSPATLSLSPGERATLSCRASQSVSSYLAWYQQKPGQAPRLLIYDASNRATGIPARFSGSGSGTDFTLTISSLEPEDFAVYYCQQRSNWPITFGQGTRLEIK

^^^^^^ ^^^ ^^^^^^^^^

Best Alignment of Ofatumumab heavy chain CDRs to a sequence from OAS

EVQLVESGGGLVQPGRSLRLSCAASGFTFNDYAMHWVRQAPGKGLEWVSTISWNSGSIGYADSVKGRFTISRDNAKKSLYLQMNSLRAEDTALYYCAKDIQYGNYYYGMDVWGQGTTVTVSS

................|||||||||||||.|||||||||||||||||||.||||||||||||||||||||||||||.||||||||||||.|||||||||||.|.||||||||||||||||||

----------------SLRLSCAASGFTFDDYAMHWVRQAPGKGLEWVSGISWNSGSIGYADSVKGRFTISRDNAKNSLYLQMNSLRAEGTALYYCAKDIQAGYYYYGMDVWGQGTTVTVSS

^^^^^^^^ ^^^^^^^^ ^^^^^^^^^^^^^^^

Best Alignment of Ofatumumab light chain CDRs to a sequence from OAS

EIVLTQSPATLSLSPGERATLSCRASQSVSSYLAWYQQKPGQAPRLLIYDASNRATGIPARFSGSGSGTDFTLTISSLEPEDFAVYYCQQRSNWPITFGQGTRLEIK

|||||||||||||||||||||||||||||||||||||||||||||||||||||||||||||||||||||||||||||||||||||||||||||||||||||||||||

EIVLTQSPATLSLSPGERATLSCRASQSVSSYLAWYQQKPGQAPRLLIYDASNRATGIPARFSGSGSGTDFTLTISSLEPEDFAVYYCQQRSNWPITFGQGTRLEIK

^^^^^^ ^^^ ^^^^^^^^^

Best Alignment of Ofatumumab CDR-H3 to a sequence from OAS

EVQLVESGGGLVQPGRSLRLSCAASGFTFNDYAMHWVRQAPGKGLEWVSTISWNSGSIGYADSVKGRFTISRDNAKKSLYLQMNSLRAEDTALYYCAKDIQYGNYYYGMDVWGQGTTVTVSS

..............|.|||||||||||||..|.|||||||||||||.|..||.......|.|||||||||||||.|..||||||||||||||.|||||||.||||||||||||.||||||||

--------------GGSLRLSCAASGFTFSSYGMHWVRQAPGKGLESVADISYDGSNKYYVDSVKGRFTISRDNSKNTLYLQMNSLRAEDTAVYYCAKDIAYGNYYYGMDVWGKGTTVTVSS

^^^^^^^^ ^^^^^^^^ ^^^^^^^^^^^^^^^

Therapeutic : Olaratumab

Best Alignment of Olaratumab heavy chain to a sequence from OAS

QLQLQESGPGLVKPSETLSLTCTVSGGSINSSSYYWGWLRQSPGKGLEWIGSFFYTGSTYYNPSLRSRLTISVDTSKNQFSLMLSSVTAADTAVYYCARQSTYYYGSGNYYGWFDRWDQGTLVTVSS

|||||||||||||||||||||||||||||.||||||||.||.||||||||||..|.|||||||||.||.|||||||||||||.||||||||||||||||..|||||||.|||.||.|.|||||||||

QLQLQESGPGLVKPSETLSLTCTVSGGSISSSSYYWGWIRQPPGKGLEWIGSIYYSGSTYYNPSLKSRVTISVDTSKNQFSLKLSSVTAADTAVYYCARGRTYYYGSGSYYGLFDYWGQGTLVTVSS

^^^^^^^^^^ ^^^^^^^ ^^^^^^^^^^^^^^^^^^^

Best Alignment of Olaratumab light chain to a sequence from OAS

EIVLTQSPATLSLSPGERATLSCRASQSVSSYLAWYQQKPGQAPRLLIYDASNRATGIPARFSGSGSGTDFTLTISSLEPEDFAVYYCQQRSNWPPAFGQGTKVEIK

|||||||||||||||||||||||||||||||||||||||||||||||||||||||||||||||||||||||||||||||||||||||||||||||||||||||||||

EIVLTQSPATLSLSPGERATLSCRASQSVSSYLAWYQQKPGQAPRLLIYDASNRATGIPARFSGSGSGTDFTLTISSLEPEDFAVYYCQQRSNWPPAFGQGTKVEIK

^^^^^^ ^^^ ^^^^^^^^^

Best Alignment of Olaratumab heavy chain CDRs to a sequence from OAS

QLQLQESGPGLVKPSETLSLTCTVSGGSINSSSYYWGWLRQSPGKGLEWIGSFFYTGSTYYNPSLRSRLTISVDTSKNQFSLMLSSVTAADTAVYYCARQSTYYYGSGNYYGWFDRWDQGTLVTVSS

..............||||||||.||||||.||||||||.||.|||||||||...|.|||||||||.||.|||||||||||||.||||||||||||||||.|.||||||.||||||.|.|||||||||

--------------SETLSLTCAVSGGSISSSSYYWGWIRQPPGKGLEWIGGIYYSGSTYYNPSLKSRVTISVDTSKNQFSLKLSSVTAADTAVYYCARHSPYYYGSGSYYGWFDPWGQGTLVTVSS

^^^^^^^^^^ ^^^^^^^ ^^^^^^^^^^^^^^^^^^^

Best Alignment of Olaratumab light chain CDRs to a sequence from OAS

EIVLTQSPATLSLSPGERATLSCRASQSVSSYLAWYQQKPGQAPRLLIYDASNRATGIPARFSGSGSGTDFTLTISSLEPEDFAVYYCQQRSNWPPAFGQGTKVEIK

|||||||||||||||||||||||||||||||||||||||||||||||||||||||||||||||||||||||||||||||||||||||||||||||||||.|||||||

EIVLTQSPATLSLSPGERATLSCRASQSVSSYLAWYQQKPGQAPRLLIYDASNRATGIPARFSGSGSGTDFTLTISSLEPEDFAVYYCQQRSNWPPAFGGGTKVEIK

^^^^^^ ^^^ ^^^^^^^^^

Best Alignment of Olaratumab CDR-H3 to a sequence from OAS

QLQLQESGPGLVKPSETLSLTCTVSGGSINSSSYYWGWLRQSPGKGLEWIGSFFYTGSTYYNPSLRSRLTISVDTSKNQFSLMLSSVTAADTAVYYCARQSTYYYGSGNYYGWFDRWDQGTLVTVSS

.............||||||||||||||||..|||||.|.||.|||||||||...|.|||.|||||.||.|||||||||||||.||||||||||||||||.||||||||.|||.||.|.|||.|||||

-------------PSETLSLTCTVSGGSI--SSYYWSWIRQPPGKGLEWIGYIYYSGSTNYNPSLKSRVTISVDTSKNQFSLKLSSVTAADTAVYYCARDSTYYYGSGSYYGAFDIWGQGTMVTVSS

^^^^^^^^^^ ^^^^^^^ ^^^^^^^^^^^^^^^^^^^

Therapeutic : Olendalizumab

Best Alignment of Olendalizumab heavy chain to a sequence from OAS

QVQLVQSGAEVKKPGASVKVSCKASGYTFTDYSMDWVRQAPGQGLEWMGAIHLNTGYTNYNQKFKGRVTMTRDTSTSTVYMELSSLRSEDTAVYYCARGFYDGYSPMDYWGQGTTVTVSS

||||||||||||||||||||||||||||||.|.|.||||||||||||||.|..|.|.|||.|||.|||||||||||||||||||||||||||||||||.|.||||..|||||||.|||||

QVQLVQSGAEVKKPGASVKVSCKASGYTFTGYYMHWVRQAPGQGLEWMGWINPNSGGTNYAQKFQGRVTMTRDTSTSTVYMELSSLRSEDTAVYYCARDFLDGYSGFDYWGQGTLVTVSS

^^^^^^^^ ^^^^^^^^ ^^^^^^^^^^^^^

Best Alignment of Olendalizumab light chain to a sequence from OAS

DIQMTQSPSSLSASVGDRVTITCRASESVDSYGNSFMHWYQQKPGKAPKLLIYRASNLESGVPSRFSGSGSGTDFTLTISSLQPEDFATYYCQQSNEDPYTFGGGTKVEIK

||||||||||||||||||||||||||.||....||..||||||||||||||.|.||||.||||||||||||||||||||||||||||||||||||...|||||.|||||||

DIQMTQSPSSLSASVGDRVTITCRASQSV----NSYLHWYQQKPGKAPKLLMYAASNLQSGVPSRFSGSGSGTDFTLTISSLQPEDFATYYCQQSYSTPYTFGQGTKVEIK

^^^^^^^^^^ ^^^ ^^^^^^^^^

Best Alignment of Olendalizumab heavy chain CDRs to a sequence from OAS

QVQLVQSGAEVKKPGASVKVSCKASGYTFTDYSMDWVRQAPGQGLEWMGAIHLNTGYTNYNQKFKGRVTMTRDTSTSTVYMELSSLRSEDTAVYYCARGFYDGYSPMDYWGQGTTVTVSS

.|||.|.|||..|||||||.||||||||||.|.|.||.|.|||||||.|.||.|.|.||||.|||...|.|.|.|.||.||.||||.|||.||||||||.|||||.||||||||.|||||

-VQLQQPGAELVKPGASVKLSCKASGYTFTSYWMHWVKQRPGQGLEWIGMIHPNSGSTNYNEKFKSKATLTVDKSSSTAYMQLSSLTSEDSAVYYCARGDYDGYSFMDYWGQGTSVTVSS

^^^^^^^^ ^^^^^^^^ ^^^^^^^^^^^^^

Best Alignment of Olendalizumab light chain CDRs to a sequence from OAS

DIQMTQSPSSLSASVGDRVTITCRASESVDSYGNSFMHWYQQKPGKAPKLLIYRASNLESGVPSRFSGSGSGTDFTLTISSLQPEDFATYYCQQSNEDPYTFGGGTKVEIK

||..||||.||..|.|.|.||.|||||||||||||||||||||||..||||||||||||||.|.|||||||.|||||||......|.||||||||||||||||||||.|||

DIVLTQSPVSLAVSLGQRATISCRASESVDSYGNSFMHWYQQKPGQPPKLLIYRASNLESGIPARFSGSGSRTDFTLTINPVEADDVATYYCQQSNEDPYTFGGGTKLEIK

^^^^^^^^^^ ^^^ ^^^^^^^^^

Best Alignment of Olendalizumab CDR-H3 to a sequence from OAS

QVQLVQSGAEVKKPGASVKVSCKASGYTFTDYSMDWVRQAPGQGLEWMGAIHLNTGYTNYNQKFKGRVTMTRDTSTSTVYMELSSLRSEDTAVYYCARGFYDGYSPMDYWGQGTTVTVSS

.|||.|||....|||||||.||||||||||||.|.||.|..||.|||.|.|....|.|.||.||||..|.|.|.|.||.||||.||.|||.||||||||||||||.||||||||.|||||

EVQLQQSGPVLVKPGASVKMSCKASGYTFTDYYMNWVKQSHGQSLEWIGVINPYNGVTSYNPKFKGKATLTVDKSSSTAYMELNSLTSEDSAVYYCARGFYDGYSFMDYWGQGTSVTVSS

^^^^^^^^ ^^^^^^^^ ^^^^^^^^^^^^^

Therapeutic : Olokizumab

Best Alignment of Olokizumab heavy chain to a sequence from OAS

EVQLVESGGGLVQPGGSLRLSCAASGFNFNDYFMNWVRQAPGKGLEWVAQMRNKNYQYGTYYAESLEGRFTISRDDSKNSLYLQMNSLKTEDTAVYYCARESYY---GFTSYWGQGTLVTVSS

|||||||||||||||||||||||||||.|..|.|||||||||||||||...|||...|.|.||.|..|||||||||||||||||||||||||||||||||..||...|...||||||||||||

EVQLVESGGGLVQPGGSLRLSCAASGFTFSSYEMNWVRQAPGKGLEWVGRTRNKANSYTTEYAASVKGRFTISRDDSKNSLYLQMNSLKTEDTAVYYCARVDYYDSSGWYDYWGQGTLVTVSS

^^^^^^^^ ^^^^^^^^^^ ^^^^^^^^^^^^^^

Best Alignment of Olokizumab light chain to a sequence from OAS

DIQMTQSPSSLSASVGDRVTITCQASQDIGISLSWYQQKPGKAPKLLIYNANNLADGVPSRFSGSGSGTDFTLTISSLQPEDFATYYCLQHNSAPYTFGQGTKLEIK

|||||||||||||||||||||||||||||...|.|||||||||||||||.|.||..|||||||||||||.|||||||||||||||||||||||.|||||||||||||

DIQMTQSPSSLSASVGDRVTITCQASQDISNNLHWYQQKPGKAPKLLIYDASNLETGVPSRFSGSGSGTEFTLTISSLQPEDFATYYCLQHNSYPYTFGQGTKLEIK

^^^^^^ ^^^ ^^^^^^^^^

Best Alignment of Olokizumab heavy chain CDRs to a sequence from OAS

EVQLVESGGGLVQPGGSLRLSCAASGFNFNDYFMNWVRQAPGKGLEWVAQMRNKNYQYGTYYAESLEGRFTISRDDSKNSLYLQMNSLKTEDTAVYYCARESYYGFTSYWGQGTLVTVSS

||.|.||||||||||||||||||.|||.|.||.|.||||.|||.|||....|||...|.|.|..|..||||||||.|...||||||.|..||.|.||||||||||..||||||||||||.

EVKLMESGGGLVQPGGSLRLSCATSGFTFTDYYMSWVRQPPGKALEWLGFIRNKANGYTTEYSASVKGRFTISRDNSQSILYLQMNTLRAEDSATYYCARESYYGSSSYWGQGTLVTVSA

^^^^^^^^ ^^^^^^^^^^ ^^^^^^^^^^^

Best Alignment of Olokizumab light chain CDRs to a sequence from OAS

DIQMTQSPSSLSASVGDRVTITCQASQDIGISLSWYQQKPGKAPKLLIYNANNLADGVPSRFSGSGSGTDFTLTISSLQPEDFATYYCLQHNSAPYTFGQGTKLEIK

|||||||||||||||||.|||||.|||||.|||.|.|||||||||||||.|.....||||||||.||||||||.||||||||||.||||||||.||.||||||.|||

DIQMTQSPSSLSASVGDTVTITCRASQDISISLNWFQQKPGKAPKLLIYAASSVESGVPSRFSGGGSGTDFTLSISSLQPEDFAVYYCLQHNSYPYSFGQGTKVEIK

^^^^^^ ^^^ ^^^^^^^^^

Best Alignment of Olokizumab CDR-H3 to a sequence from OAS

EVQLVESGGGLVQPGGSLRLSCAASGFNFNDYFMNWVRQAPGKGLEWVAQMRNKNYQYGTYYAESLEGRFTISRDDSKNSLYLQMNSLKTEDTAVYYCARESYYGFTSYWGQGTLVTVSS

.............||||||||||||||.|..|.|.||||||||||||||..|........|||.|..||||||||..|||||||||||..|||||||||||||||..|||||||||||||

-------------PGGSLRLSCAASGFTFSSYGMHWVRQAPGKGLEWVAFIRYD--GSNKYYADSVKGRFTISRDNAKNSLYLQMNSLRAEDTAVYYCARESYYGSGSYWGQGTLVTVSS

^^^^^^^^ ^^^^^^^^^^ ^^^^^^^^^^^

Therapeutic : Omalizumab

Best Alignment of Omalizumab heavy chain to a sequence from OAS

EVQLVESGGGLVQPGGSLRLSCAVSGYSITSGYSWNWIRQAPGKGLEWVASITYD-GSTNYNPSLKGRITISRDDSKNTFYLQMNSLRAEDTAVYYCARGSHYF-GHWHFAVWGQGTLVTVSS

|||||||||||||||||||||||.||....|.|..||.|||||||||||..|....|||.|..|.|||.|||||.||||.||||||||||||||||||.||....|.|.|..|||||||||||

EVQLVESGGGLVQPGGSLRLSCAASGFTF-SSYAMNWVRQAPGKGLEWVSAISGSGGSTYYADSVKGRFTISRDNSKNTLYLQMNSLRAEDTAVYYCAKGSDFWSGYWWFDPWGQGTLVTVSS

^^^^^^^^^ ^^^^^^^^ ^^^^^^^^^^^^^^^

Best Alignment of Omalizumab light chain to a sequence from OAS

DIQLTQSPSSLSASVGDRVTITCRASQSVDYDGDSYMNWYQQKPGKAPKLLIYAASYLESGVPSRFSGSGSGTDFTLTISSLQPEDFATYYCQQSHEDPYTFGQGTKVEIK

||||||||||||||||||||||||||||......||.|||||||||||||||||||.|.|||||||||||||||||||||||||||||||||||||..|.|||||||||||

DIQLTQSPSSLSASVGDRVTITCRASQSI----SSYLNWYQQKPGKAPKLLIYAASSLQSGVPSRFSGSGSGTDFTLTISSLQPEDFATYYCQQSHSTPRTFGQGTKVEIK

^^^^^^^^^^ ^^^ ^^^^^^^^^

Best Alignment of Omalizumab heavy chain CDRs to a sequence from OAS

EVQLVESGGGLVQPGGSLRLSCAVSGYSITSGYSWNWIRQAPGKGLEWVASITYDGSTNYNPSLKGRITISRDDSKNTFYLQMNSLRAEDTAVYYCARGSHYFGHWHFAVWGQGTLVTVSS

.|||.|||.|||.|..||.|.|.|.||||||||.||||||.||..|||...|.||||.|||||||.||.|.||.|||.|.|..||...||||.|||||||.|.|||.|.|||.||.|||||

-VQLKESGPGLVKPSQSLSLTCSVTGYSITSGYYWNWIRQFPGNKLEWMGYISYDGSNNYNPSLKNRISITRDTSKNQFFLKLNSVTTEDTATYYCARGSDYYGHWYFDVWGAGTTVTVSS

^^^^^^^^^ ^^^^^^^ ^^^^^^^^^^^^^^

Best Alignment of Omalizumab light chain CDRs to a sequence from OAS

DIQLTQSPSSLSASVGDRVTITCRASQSVDYDGDSYMNWYQQKPGKAPKLLIYAASYLESGVPSRFSGSGSGTDFTLTISSLQPEDFATYYCQQSHEDPYTFGQGTKVEIK

||.|||||.||..|.|.|.||.|.|||||||||||||||||||||.||||||||||.|.||.|.|||||||||||||.|.....||.||||||||||||||||.|||.|||

DIVLTQSPASLAVSLGQRATISCKASQSVDYDGDSYMNWYQQKPGQAPKLLIYAASNLDSGIPARFSGSGSGTDFTLNIHPVEEEDAATYYCQQSHEDPYTFGGGTKLEIK

^^^^^^^^^^ ^^^ ^^^^^^^^^

Best Alignment of Omalizumab CDR-H3 to a sequence from OAS

EVQLVESGGGLVQPGGSLRLSCAVSGYSITSGYSWNWIRQAPGKGLEWVASITYD-GSTNYNPSLKGRITISRDDSKNTFYLQMNSLRAEDTAVYYCARGSHYFGHWHFAVWGQGTLVTVSS

................|||||||.||......|...|.|||||||||||..|.....|..|..|..||..||||.||||.|||||.||.||||.||.||||.|..||.||||..|.||||||

----------------SLRLSCAASGFTF-NNYAMAWVRQAPGKGLEWVSVISGSATSMYYAESVRGRFIISRDKSKNTLYLQMNRLRVEDTAEYYWARGSGYYFHWYFAVWASGALVTVSS

^^^^^^^^^ ^^^^^^^^ ^^^^^^^^^^^^^^

Therapeutic : Onartuzumab

Best Alignment of Onartuzumab heavy chain to a sequence from OAS

EVQLVESGGGLVQPGGSLRLSCAASGYTFTSYWLHWVRQAPGKGLEWVGMIDPSNSDTRFNPNFKDRFTISADTSKNTAYLQMNSLRAEDTAVYYCATYRSYVTPLDYWGQGTLVTVSS

||||||||||||||||||||||||||.||.|||.||||||||||||||..|..|...|......|.|||||.|.||||.||||||||||||||||||..|..|.|||||||||||||||

EVQLVESGGGLVQPGGSLRLSCAASGFTFSSYWMHWVRQAPGKGLEWVSAISGSGGSTYYADSVKGRFTISRDNSKNTLYLQMNSLRAEDTAVYYCARGRIAVAPLDYWGQGTLVTVSS

^^^^^^^^ ^^^^^^^^ ^^^^^^^^^^^^

Best Alignment of Onartuzumab light chain to a sequence from OAS

DIQMTQSPSSLSASVGDRVTITCKSSQSLLYTSSQKNYLAWYQQKPGKAPKLLIYWASTRESGVPSRFSGSGSGTDFTLTISSLQPEDFATYYCQQYYAYPWTFGQGTKVEIK

||.|||||.||..|.|.||||.|||||||||||..||||||||||||.|||||||||||||||||.||||||||||||||||.|||||||||||||...||.|||||||||||

DIVMTQSPDSLAVSLGERVTINCKSSQSLLYTSNNKNYLAWYQQKPGQAPKLLIYWASTRESGVPNRFSGSGSGTDFTLTISGLQPEDFATYYCQQHNSYPRTFGQGTKVEIK

^^^^^^^^^^^^ ^^^ ^^^^^^^^^

Best Alignment of Onartuzumab heavy chain CDRs to a sequence from OAS

EVQLVESGGGLVQPGGSLRLSCAASGYTFTSYWLHWVRQAPGKGLEWVGMIDPSNSDTRFNPNFKDRFTISADTSKNTAYLQMNSLRAEDTAVYYCATYRSYVTPLDYWGQGTLVTVSS

.|||..||..||.||.|...||.||||||||||.|||.|.||.||||.||||||||.||.|..|||..|...|.|.||||.|..||..||.|||||||||..|...|||||||.|||||

QVQLKQSGPELVRPGASVKMSCKASGYTFTSYWMHWVKQRPGQGLEWIGMIDPSNSETRLNQKFKDKATLNVDKSSNTAYMQLSSLTSEDSAVYYCATYRYDVYAMDYWGQGTSVTVSS

^^^^^^^^ ^^^^^^^^ ^^^^^^^^^^^^

Best Alignment of Onartuzumab light chain CDRs to a sequence from OAS

DIQMTQSPSSLSASVGDRVTITCKSSQSLLYTSSQKNYLAWYQQKPGKAPKLLIYWASTRESGVPSRFSGSGSGTDFTLTISSLQPEDFATYYCQQYYAYPWTFGQGTKVEIK

.|.|.||||||..|||..||..|||||||||||.|||||.|||||||..||.||||||.||||||.||.|||.||||||||||...||.|.|||||||.||.|||.|||.|||

-IVMSQSPSSLAVSVGEKVTMSCKSSQSLLYTSNQKNYLPWYQQKPGHSPKPLIYWASPRESGVPGRFTGSGTGTDFTLTISSVKAEDLAVYYCQQYYSYPPTFGGGTKLEIK

^^^^^^^^^^^^ ^^^ ^^^^^^^^^

Best Alignment of Onartuzumab CDR-H3 to a sequence from OAS

EVQLVESGGGLVQPGGSLRLSCAASGYTFTSYWLHWVRQAPGKGLEWVGMIDPSNSDTRFNPNFKDRFTISADTSKNTAYLQMNSLRAEDTAVYYCATYRSYVTPLDYWGQGTLVTVSS

..............||||||||||||.||.|....|||||||||||||..|..|...|......|.|||||.|.|.||.|||||||||||||||||||||||..|.|||||||||||||

--------------GGSLRLSCAASGFTFSSNAMSWVRQAPGKGLEWVSAISGSADSTYYADSVKGRFTISRDNSENTVYLQMNSLRAEDTAVYYCATYRSYYHPFDYWGQGTLVTVSS

^^^^^^^^ ^^^^^^^^ ^^^^^^^^^^^^

Therapeutic : Ontuxizumab

Best Alignment of Ontuxizumab heavy chain to a sequence from OAS

QVQLQESGPGLVRPSQTLSLTCTASGYTFTDYVIHWVKQPPGRGLEWIGYINPYDDDTTYNQKFKGRVTMLVDTSSNTAYLRLSSVTAEDTAVYYCARRGNSYDGYFDYSMDYWGSGTPVTVSS

.||||.|||.||.|.......|.|||||||.||.|||||.||.|||||||||||.|.|.||.||||..|...|.||.|||..|||.|.||.|||||||.|..|||||.|||||||.||.|||||

EVQLQQSGPELVKPGASGKMSCKASGYTFTSYVMHWVKQKPGQGLEWIGYINPYNDGTKYNEKFKGKATLTSDKSSSTAYMELSSLTSEDSAVYYCARGGTPYDGYFYYSMDYWGQGTSVTVSS

^^^^^^^^ ^^^^^^^^ ^^^^^^^^^^^^^^^^^

Best Alignment of Ontuxizumab light chain to a sequence from OAS

DIQMTQSPSSLSASVGDRVTITCRASQNVGTAVAWLQQTPGKAPKLLIYSASNRYTGVPSRFSGSGSGTDYTFTISSLQPEDIATYYCQQYTNYPMYTFGQGTKVQIK

|||||||||||||||||||||||||||......||.||.||||||||||.|||..|||||||||||||||.||||||||||||||||||||.|.||||||||||..||

DIQMTQSPSSLSASVGDRVTITCRASQGISNYLAWYQQKPGKAPKLLIYDASNLETGVPSRFSGSGSGTDFTFTISSLQPEDIATYYCQQYDNLPMYTFGQGTKLEIK

^^^^^^ ^^^ ^^^^^^^^^^

Best Alignment of Ontuxizumab heavy chain CDRs to a sequence from OAS

QVQLQESGPGLVRPSQTLSLTCTASGYTFTDYVIHWVKQPPGRGLEWIGYINPYDDDTTYNQKFKGRVTMLVDTSSNTAYLRLSSVTAEDTAVYYCARRGNSYDGYFDYSMDYWGSGTPVTVSS

.||||.|||.||.|.......|.|||||||.||.|||||.||.|||||||||||.|.|.||.||||..|...|.||.|||..|||.|.||.|||||||.|..|||||.|||||||.||.|||||

EVQLQQSGPELVKPGASGKMSCKASGYTFTSYVMHWVKQKPGQGLEWIGYINPYNDGTKYNEKFKGKATLTSDKSSSTAYMELSSLTSEDSAVYYCARGGTPYDGYFYYSMDYWGQGTSVTVSS

^^^^^^^^ ^^^^^^^^ ^^^^^^^^^^^^^^^^^

Best Alignment of Ontuxizumab light chain CDRs to a sequence from OAS

DIQMTQSPSSLSASVGDRVTITCRASQNVGTAVAWLQQTPGKAPKLLIYSASNRYTGVPSRFSGSGSGTDYTFTISSLQPEDIATYYCQQYTNYPMYTFGQGTKVQIK

.|..|||....|.|||||...||.|||||||.|||.||.||..||.||||||.||.|||.||.|||||||.|.|.|..|.||.|.|.||||.|||.||||.|||..||

-IVLTQSQKFVSPSVGDRDSVTCKASQNVGTNVAWYQQKPGQSPKPLIYSASYRYSGVPDRFTGSGSGTDFTLTVSNVQSEDLAEYFCQQYNNYPPYTFGGGTKLEIK

^^^^^^ ^^^ ^^^^^^^^^^

Best Alignment of Ontuxizumab CDR-H3 to a sequence from OAS

QVQLQESGPGLVRPSQTLSLTCTASGYTFTDYVIHWVKQPPGRGLEWIGYINPYDDDTTYNQKFKGRVTMLVDTSSNTAYLRLSSVTAEDTAVYYCARRGNSYDGYFDYSMDYWGSGTPVTVSS

.||||.||..||||.....|.|.|||||||||.|.||||.||.|||||..|.|....|.||.||||..|.....||.|||..|||.|.||.|||.|||||.|||||.||.|||||.||.|||||

-VQLQQSGAELVRPGASVKLSCKASGYTFTDYYINWVKQRPGQGLEWIARIYPGSGNTYYNEKFKGKATLTAEKSSSTAYMQLSSLTSEDSAVYVCARRGVSYDGYYDYAMDYWGQGTSVTVSS

^^^^^^^^ ^^^^^^^^ ^^^^^^^^^^^^^^^^^

Therapeutic : Opicinumab

Best Alignment of Opicinumab heavy chain to a sequence from OAS

EVQLLESGGGLVQPGGSLRLSCAASGFTFSAYEMKWVRQAPGKGLEWVSVIGPSGGFTFYADSVKGRFTISRDNSKNTLYLQMNSLRAEDTAVYYCATEGDN---DAFDIWGQGTTVTVSS

||||||||||||||||||||||||||||||.|.|.||||||||||||||.|..|||.|.||||||||||||||||||||||||||||||||||||||.||||...||||||||||.|||||

EVQLLESGGGLVQPGGSLRLSCAASGFTFSSYAMSWVRQAPGKGLEWVSTISGSGGSTYYADSVKGRFTISRDNSKNTLYLQMNSLRAEDTAVYYCAREGDNWNEDAFDIWGQGTMVTVSS

^^^^^^^^ ^^^^^^^^ ^^^^^^^^^^^^^^

Best Alignment of Opicinumab light chain to a sequence from OAS

DIQMTQSPATLSLSPGERATLSCRASQSVSSYLAWYQQKPGQAPRLLIYDASNRATGIPARFSGSGSGTDFTLTISSLEPEDFAVYYCQQRSNWPMYTFGQGTKLEIK

.|.|||||||||||||||||||||||||||||||||||||||||||||||||||||||||||||||||||||||||||||||||||||||||||||||||||||||||

EIVMTQSPATLSLSPGERATLSCRASQSVSSYLAWYQQKPGQAPRLLIYDASNRATGIPARFSGSGSGTDFTLTISSLEPEDFAVYYCQQRSNWPMYTFGQGTKLEIK

^^^^^^ ^^^ ^^^^^^^^^^

Best Alignment of Opicinumab heavy chain CDRs to a sequence from OAS

EVQLLESGGGLVQPGGSLRLSCAASGFTFSAYEMKWVRQAPGKGLEWVSVIGPSGGFTFYADSVKGRFTISRDNSKNTLYLQMNSLRAEDTAVYYCATEGDNDAFDIWGQGTTVTVSS

..............|.||..||||||||||.|||.||||||||||||||.|..||..|..||||||||||||||.||.|||||||||||||||||||.||||||||||||||.|||||

--------------GESLKISCAASGFTFSNYEMIWVRQAPGKGLEWVSYISSSGSTTYSADSVKGRFTISRDNAKNSLYLQMNSLRAEDTAVYYCAREGDNDAFDIWGQGTMVTVSS

^^^^^^^^ ^^^^^^^^ ^^^^^^^^^^^

Best Alignment of Opicinumab light chain CDRs to a sequence from OAS

DIQMTQSPATLSLSPGERATLSCRASQSVSSYLAWYQQKPGQAPRLLIYDASNRATGIPARFSGSGSGTDFTLTISSLEPEDFAVYYCQQRSNWPMYTFGQGTKLEIK

.|..||||.|||||||||||||||||||||||||||||||||||||||||||||||||||||||||||||||||||||||||||||||||||||||||||||||||||

EIVLTQSPVTLSLSPGERATLSCRASQSVSSYLAWYQQKPGQAPRLLIYDASNRATGIPARFSGSGSGTDFTLTISSLEPEDFAVYYCQQRSNWPMYTFGQGTKLEIK

^^^^^^ ^^^ ^^^^^^^^^^

Best Alignment of Opicinumab CDR-H3 to a sequence from OAS

EVQLLESGGGLVQPGGSLRLSCAASGFTFSAYEMKWVRQAPGKGLEWVSVIGPSGGFTFYADSVKGRFTISRDNSKNTLYLQMNSLRAEDTAVYYCATEGDNDAFDIWGQGTTVTVSS

.................|.|.|..||...|.|...|.||.|||||||...|..|.|.|.|..|.|.|.|||.|.|||...|...|..|.||||||||||||.||||||||||.|||||

----------------TLSLTCTVSGGSISSYYWSWIRQPPGKGLEWIGYIYYS-GSTNYNPSLKSRVTISVDTSKNQFSLKLSSVTAADTAVYYCATEGDRDAFDIWGQGTMVTVSS

^^^^^^^^ ^^^^^^^^ ^^^^^^^^^^^

Therapeutic : Orticumab

Best Alignment of Orticumab heavy chain to a sequence from OAS

EVQLLESGGGLVQPGGSLRLSCAASGFTFSNAWMSWVRQAPGKGLEWVSSISVGGHRTYYADSVKGRSTISRDNSKNTLYLQMNSLRAEDTAVYYCARIRVGP-SGGAFDYWGQGTLVTVSS

||||||||||||||||||||||||||||||...||||||||||||||||.||.||..||||||||||.||||||||||||||||||||||||||||||..||..|.|.||||||||||||||

EVQLLESGGGLVQPGGSLRLSCAASGFTFSSYAMSWVRQAPGKGLEWVSAISGGGGSTYYADSVKGRFTISRDNSKNTLYLQMNSLRAEDTAVYYCARGLVGATSRGYFDYWGQGTLVTVSS

^^^^^^^^ ^^^^^^^^ ^^^^^^^^^^^^^^^

Best Alignment of Orticumab light chain to a sequence from OAS

QSVLTQPPSASGTPGQRVTISCSGSNTNIGKNYVSWYQQLPGTAPKLLIYANSNRPSGVPDRFSGSKSGTSASLAISGLRSEDEADYYCASWDASLNGWVFGGGTKLTVL

|||||||||||||||||||||||||..|||||||.|||||||||||||||.||.||||||||||||||||||||||||||||||||||||.||.||.|||||||||||||

QSVLTQPPSASGTPGQRVTISCSGSSSNIGKNYVYWYQQLPGTAPKLLIYRNSQRPSGVPDRFSGSKSGTSASLAISGLRSEDEADYYCAVWDDSLGGWVFGGGTKLTVL

^^^^^^^^ ^^^ ^^^^^^^^^^^

Best Alignment of Orticumab heavy chain CDRs to a sequence from OAS

EVQLLESGGGLVQPGGSLRLSCAASGFTFSNAWMSWVRQAPGKGLEWVSSISVGGHRTYYADSVKGRSTISRDNSKNTLYLQMNSLRAEDTAVYYCARIRVGPSGGAFDYWGQGTLVTVSS

||||.||||||||||.||.||||||||||||..|.||||.|.||||||.|||.||..|||.||||||.||||||.|||.||||.|||.||||.|||||..||||||.|||||||..|||||

EVQLVESGGGLVQPGRSLKLSCAASGFTFSNYGMAWVRQTPTKGLEWVASISTGGGNTYYRDSVKGRFTISRDNAKNTQYLQMDSLRSEDTATYYCARHEVGPSGGYFDYWGQGVMVTVSS

^^^^^^^^ ^^^^^^^^ ^^^^^^^^^^^^^^

Best Alignment of Orticumab light chain CDRs to a sequence from OAS

QSVLTQPPSASGTPGQRVTISCSGSNTNIGKNYVSWYQQLPGTAPKLLIYANSNRPSGVPDRFSGSKSGTSASLAISGLRSEDEADYYCASWDASLNGWVFGGGTKLTVL

|||||||||.|..|||.|||||||||.|||.|||.||||.||||||.|||.||.||||.|.||||||||||||||||||..|||||||||.||.||||||||||||||||

QSVLTQPPSVSAAPGQKVTISCSGSNSNIGNNYVTWYQQFPGTAPKVLIYDNSRRPSGIPARFSGSKSGTSASLAISGLQAEDEADYYCAAWDDSLNGWVFGGGTKLTVL

^^^^^^^^ ^^^ ^^^^^^^^^^^

Best Alignment of Orticumab CDR-H3 to a sequence from OAS

EVQLLESGGGLVQPGGSLRLSCAASGFTFSNAWMSWVRQAPGKGLEWVSSISVGGHRTYYADSVKGRSTISRDNSKNTLYLQMNSLRAEDTAVYYCARIRVGPSGGAFDYWGQGTLVTVSS

.|||.|||.|||.....|.|.|..||...|....||.||..|||||....|......|.|..|.|.|.|.|.|.|||...|...|..|.|||||||||.||..||||||||||||||||||

QVQLQESGPGLVETSETLSLTCNVSGGSISSYYWSWIRQPAGKGLEYIGRIHTS-GSTNYNPSLKSRVTMSVDMSKNQFSLMLTSVTAADTAVYYCARLRVPASGGAFDYWGQGTLVTVSS

^^^^^^^^ ^^^^^^^^ ^^^^^^^^^^^^^^

Therapeutic : Otelixizumab

Best Alignment of Otelixizumab heavy chain to a sequence from OAS

EVQLLESGGGLVQPGGSLRLSCAASGFTFSSFPMAWVRQAPGKGLEWVSTISTSGGRTYYRDSVKGRFTISRDNSKNTLYLQMNSLRAEDTAVYYCAKFRQY---SGGFDYWGQGTLVTVSS

|||||||||||||||||||||||||||||||..|.||||||||||||||.||.|||.|||.|||||||||||||||||||||||||||||||||||||.|||...|||.|||||||||||||

EVQLLESGGGLVQPGGSLRLSCAASGFTFSSYAMSWVRQAPGKGLEWVSAISGSGGSTYYADSVKGRFTISRDNSKNTLYLQMNSLRAEDTAVYYCAKDRQYYDSSGGVDYWGQGTLVTVSS

^^^^^^^^ ^^^^^^^^ ^^^^^^^^^^^^^^^

Best Alignment of Otelixizumab light chain to a sequence from OAS

DIQLTQPNSVSTSLGSTVKLSCTLSSGNIENNYVHWYQLYEGRSPTTMIYDDDKRPDGVPDRFSGSIDRSSNSAFLTIHNVAIEDEAIYFCHSYVS-SFNVFGGGTKLTVL

...||||.|||.|.|.||..|||.|||||.||||.|||...|..|||.||||||||.|||||||||||||||||.|||.....||||.|.|.||.|.|..|||||||||||

NFMLTQPHSVSESPGKTVIISCTRSSGNIGNNYVQWYQQRPGSAPTTVIYDDDKRPSGVPDRFSGSIDRSSNSASLTISGLKTEDEADYYCQSYDSSSDVVFGGGTKLTVL

^^^^^^^^ ^^^ ^^^^^^^^^^

Best Alignment of Otelixizumab heavy chain CDRs to a sequence from OAS

EVQLLESGGGLVQPGGSLRLSCAASGFTFSSFPMAWVRQAPGKGLEWVSTISTSGGRTYYRDSVKGRFTISRDNSKNTLYLQMNSLRAEDTAVYYCAKFRQYSGGFDYWGQGTLVTVSS

||||.||||||||||.|..||||||||||||||||||||||.||||||.|||||||.|||||||||||||||||.|.||||||||||.||||.|||...|.|||||||||||..|||||

EVQLVESGGGLVQPGRSMKLSCAASGFTFSSFPMAWVRQAPTKGLEWVATISTSGGSTYYRDSVKGRFTISRDNAKSTLYLQMNSLRSEDTATYYCTRDRYYSGGFDYWGQGVMVTVSS

^^^^^^^^ ^^^^^^^^ ^^^^^^^^^^^^

Best Alignment of Otelixizumab light chain CDRs to a sequence from OAS

DIQLTQPNSVSTSLGSTVKLSCTLSSGNIENNYVHWYQLYEGRSPTTMIYDDDKRPDGVPDRFSGSIDRSSNSAFLTIHNVAIEDEAIYFCHSYVSSFNVFGGGTKLTVL

...||||.|||.|.|.||..|||.|||||.||||.|||...|..|||.||||||||.|||||||||||.|||||.|||.....||||.|.|.||.||..|||||||||||

NFMLTQPHSVSESPGKTVIISCTRSSGNIGNNYVQWYQQRPGSAPTTVIYDDDKRPSGVPDRFSGSIDSSSNSASLTISGLKTEDEADYYCQSYDSSNQVFGGGTKLTVL

^^^^^^^^ ^^^ ^^^^^^^^^

Best Alignment of Otelixizumab CDR-H3 to a sequence from OAS

EVQLLESGGGLVQPGGSLRLSCAASGFTFSSFPMAWVRQAPGKGLEWVSTISTSGGRTYYRDSVKGRFTISRDNSKNTLYLQMNSLRAEDTAVYYCAKFRQYSGGFDYWGQGTLVTVSS

.|||...|..||.||.|..|||.|||.||.|..|.||.|.||.||||...|....|.|.|....|...|...|....|.|....||..||.|||||||||.|.||||||||||..||||

QVQLQQPGAELVKPGASVKLSCKASGYTFTSYWMHWVKQRPGRGLEWIGRIDPNSGGTKYNEKFKSKATLTVDKPTSTAYMKLSSLTSEDSAVYYCAKFRDYDGGFDYWGQGTTLTVSS

^^^^^^^^ ^^^^^^^^ ^^^^^^^^^^^^

Therapeutic : Otlertuzumab

Best Alignment of Otlertuzumab heavy chain to a sequence from OAS

EVQLVQSGAEVKKPGESLKISCKGSGYSFTGYNMNWVRQMPGKGLEWMGNIDPYYGGTTYNRKFKGQVTISADKSISTAYLQWSSLKASDTAMYYCARSVG--PFDSWGQGTLVTVSS

||||||||||||||||||.|||||||||||.|..|||||||||||||||||||....|.|...|.|||||||||||||||||||||||||||||||||.||..|||.|||||||||||

EVQLVQSGAEVKKPGESLRISCKGSGYSFTSYWINWVRQMPGKGLEWMGNIDPGDSDTRYSPSFQGQVTISADKSISTAYLQWSSLKASDTAMYYCARHVGATPFDYWGQGTLVTVSS

^^^^^^^^ ^^^^^^^^ ^^^^^^^^^^^

Best Alignment of Otlertuzumab light chain to a sequence from OAS

EIVLTQSPATLSLSPGERATLSCRASENVYSYLAWYQQKPGQAPRLLIYFAKTLAEGIPARFSGSGSGTDFTLTISSLEPEDFAVYYCQHHSDNPWTFGQGTKVEIK

||||||||||||||||||||||||||.||.|||||||||||||||||||.|.|.|.|||||||||||||||||||||||||||||||||..|..|||||||||||||

EIVLTQSPATLSLSPGERATLSCRASQNVSSYLAWYQQKPGQAPRLLIYDASTRATGIPARFSGSGSGTDFTLTISSLEPEDFAVYYCQQRSIWPWTFGQGTKVEIK

^^^^^^ ^^^ ^^^^^^^^^

Best Alignment of Otlertuzumab heavy chain CDRs to a sequence from OAS

EVQLVQSGAEVKKPGESLKISCKGSGYSFTGYNMNWVRQMPGKGLEWMGNIDPYYGGTTYNRKFKGQVTISADKSISTAYLQWSSLKASDTAMYYCARSVGPFDSWGQGTLVTVSS

||||.|||.|..|||.|.|||||.|||||||||||||.|..||.|||.||||||||||.||.||||..|...|||.||||.|..||...|.|.||||||.||||.|||||..||||

EVQLQQSGPELEKPGASVKISCKASGYSFTGYNMNWVKQSNGKSLEWIGNIDPYYGGTSYNQKFKGKATLTVDKSSSTAYMQLKSLTSEDSAVYYCARSSGPFDYWGQGTTLTVSS

^^^^^^^^ ^^^^^^^^ ^^^^^^^^^

Best Alignment of Otlertuzumab light chain CDRs to a sequence from OAS

EIVLTQSPATLSLSPGERATLSCRASENVYSYLAWYQQKPGQAPRLLIYFAKTLAEGIPARFSGSGSGTDFTLTISSLEPEDFAVYYCQHHSDNPWTFGQGTKVEIK

.|..|||||.||.|.||..|...|||||.||||||||||.|..|..|.|.|||||||.|.|||||||||.|.|.|.||.||||..||||||.|.|||||.|||.|||

DIQITQSPASLSASVGEAVTITYRASENIYSYLAWYQQKQGKSPQVLVYNAKTLAEGVPSRFSGSGSGTQFSLKINSLQPEDFGNYYCQHHYDTPWTFGGGTKLEIK

^^^^^^ ^^^ ^^^^^^^^^

Best Alignment of Otlertuzumab CDR-H3 to a sequence from OAS

EVQLVQSGAEVKKPGESLKISCKGSGYSF--TGYNMNWVRQMPGKGLEWMGNIDPY-YGGTTYNRKFKGQVTISADKSISTAYLQWSSLKASDTAMYYCARSVGPFDSWGQGTLVTVSS

.................|...|..||.|...|....||.||.|..||||.|...........|.....|..||....|.....||..|....|||.|||||||||.|||||||||||||

--------------SQTLSLTCAISGDSVSSTNAAWNWIRQSPSRGLEWLGRTYYRSRWYNEYAVSLRGRITINPVTSKNQFSLQLNSVSPEDTAVYYCARSVGPIDSWGQGTLVTVSS

^^^^^^^^^^ ^^^^^^^^^ ^^^^^^^^^

Therapeutic : Ozanezumab

Best Alignment of Ozanezumab heavy chain to a sequence from OAS

QVQLVQSGAEVKKPGASVKVSCKASGYTFTSYWMHWVRQAPGQGLEWIGNINPSNGGTNYNEKFKSKATMTRDTSTSTAYMELSSLRSEDTAVYYCELM--------QGYWGQGTLVTVSS

||||||||||||||||||||||||||||||||.||||||||||||||.|.||||.|||.|..|||.|.||||||||||.|||||||||||||||||.............||||||||||||

QVQLVQSGAEVKKPGASVKVSCKASGYTFTSYYMHWVRQAPGQGLEWMGIINPSGGGTSYAQKFKGKVTMTRDTSTSTVYMELSSLRSEDTAVYYCARGYGSGSYYLFDYWGQGTLVTVSS

^^^^^^^^ ^^^^^^^^ ^^^^^^^^^^^^^^

Best Alignment of Ozanezumab light chain to a sequence from OAS

DIVMTQSPLSNPVTLGQPVSISCRSSKSLLYKDGKTYLNWFLQRPGQSPQLLIYLMSTRASGVPDRFSGGGSGTDFTLKISRVEAEDVGVYYCQQLVEYPLTFGQGTKLEIK

|||.||..||||||.|..|||||||||||||||||||||||||||||||||||||||||||||.|||||.||||||||.||||.||||||||||||||||||||.|||||||

DIVITQDELSNPVTSGESVSISCRSSKSLLYKDGKTYLNWFLQRPGQSPQLLIYLMSTRASGVSDRFSGSGSGTDFTLEISRVKAEDVGVYYCQQLVEYPLTFGSGTKLEIK

^^^^^^^^^^^ ^^^ ^^^^^^^^^

Best Alignment of Ozanezumab heavy chain CDRs to a sequence from OAS

QVQLVQSGAEVKKPGASVKVSCKASGYTFTSYWMHWVRQAPGQGLEWIGNINPSNGGTNYNEKFKSKATMTRDTSTSTAYMELSSLRSEDTAVYYCELMQGYWGQGTLVTVSS

.|||.|.|.|..|||||||.|||||||||||||||||.|.|||||||||||||||||||||||||||||.|.|.|.|||||.||||.|||.|||||.|.||||||||..||||

-VQLQQPGTELVKPGASVKLSCKASGYTFTSYWMHWVKQRPGQGLEWIGNINPSNGGTNYNEKFKSKATLTVDKSSSTAYMQLSSLTSEDSAVYYCGLGQGYWGQGTTLTVSS

^^^^^^^^ ^^^^^^^^ ^^^^^^

Best Alignment of Ozanezumab light chain CDRs to a sequence from OAS

DIVMTQSPLSNPVTLGQPVSISCRSSKSLLYKDGKTYLNWFLQRPGQSPQLLIYLMSTRASGVPDRFSGGGSGTDFTLKISRVEAEDVGVYYCQQLVEYPLTFGQGTKLEIK

|||.||..||||||.|..|||||||||||||||||||||||||||||||||||||||||||||.|||||.||||||||.||||.||||||||||||||||||||.|||||||

DIVITQDELSNPVTSGESVSISCRSSKSLLYKDGKTYLNWFLQRPGQSPQLLIYLMSTRASGVSDRFSGSGSGTDFTLEISRVKAEDVGVYYCQQLVEYPLTFGSGTKLEIK

^^^^^^^^^^^ ^^^ ^^^^^^^^^

Best Alignment of Ozanezumab CDR-H3 to a sequence from OAS

QVQLVQSGAEVKKPGASVKVSCKASGYTFTSYWMHWVRQAPGQGLEWIGNINPSNGGTNYNEKFKSKATMTRDTSTSTAYMELSSLRSEDTAVYYCELMQGYWGQGTLVTVSS

||||...|||..|||||||.||||||||||||||.||.|.|||||||||.|.||...||||.|||..||.|.|||.|||||.||||..|.|.||||||.|||||.||..||||

QVQLQPPGAELVKPGASVKLSCKASGYTFTSYWMQWVKQRPGQGLEWIGVIDPSDSYTNYNQKFKGQATLTVDTSSSTAYMQLSSLTYEETGVYYCELGQGYWGKGTTRTVSS

^^^^^^^^ ^^^^^^^^ ^^^^^^

Therapeutic : Palivizumab

Best Alignment of Palivizumab heavy chain to a sequence from OAS

QVTLRESGPALVKPTQTLTLTCTFSGFSLSTSGMSVGWIRQPPGKALEWLADIWWDDKKDYNPSLKSRLTISKDTSKNQVVLKVTNMDPADTATYYCARSMIT--------NWYFDVWGAGTTVTVSS

||||||||||||||||||||||||||||||||||.|.||||||||||||||.|.|||.|.|..|||.|||||||||||||||..|||||.||||||||||..|........|...||||.||||||||

QVTLRESGPALVKPTQTLTLTCTFSGFSLSTSGMCVSWIRQPPGKALEWLALIDWDDDKYYSTSLKTRLTISKDTSKNQVVLTMTNMDPVDTATYYCARSTVTAPIRRRSKNYGMDVWGQGTTVTVSS

^^^^^^^^^^ ^^^^^^^ ^^^^^^^^^^^^^^^^^^^^

Best Alignment of Palivizumab light chain to a sequence from OAS

DIQMTQSPSTLSASVGDRVTITCKCQLSV-GYMHWYQQKPGKAPKLLIYDTSKLASGVPSRFSGSGSGTEFTLTISSLQPDDFATYYCFQGSGYPFTFGGGTKLEIK

|||||||||||||||||||||||....|...|..|||||||||||||||..|.|||||||||||||||||||||||||||||||||||.|...||||||.|||||||

DIQMTQSPSTLSASVGDRVTITCRASQSISSYLAWYQQKPGKAPKLLIYKASSLASGVPSRFSGSGSGTEFTLTISSLQPDDFATYYCQQYNSYPFTFGQGTKLEIK

^^^^^^ ^^^ ^^^^^^^^^

Best Alignment of Palivizumab heavy chain CDRs to a sequence from OAS

QVTLRESGPALVKPTQTLTLTCTFSGFSLSTSGMSVGWIRQPPGKALEWLADIWWDDKKDYNPSLKSRLTISKDTSKNQVVLKVTNMDPADTATYYCARSMITNWYFDVWGAGTTVTVSS

||||..|||......|||.|||.|.|||||||||.|.|||||.||.|||||.|.|||.|.||||||||||||||||.|||.||.|..|.||||||||||||||.|||||||.||||||||

QVTLKVSGPGILQSSQTLSLTCSFPGFSLSTSGMGVSWIRQPSGKGLEWLAHIYWDDDKRYNPSLKSRLTISKDTSRNQVFLKITSVDTADTATYYCARSMITTWYFDVWGTGTTVTVSS

^^^^^^^^^^ ^^^^^^^ ^^^^^^^^^^^^

Best Alignment of Palivizumab light chain CDRs to a sequence from OAS

DIQMTQSPSTLSASVGDRVTITCKCQLSVGYMHWYQQKPGKAPKLLIYDTSKLASGVPSRFSGSGSGTEFTLTISSLQPDDFATYYCFQGSGYPFTFGGGTKLEIK

||..||.|...|||.|..||.||...|||.||||||||....|||.||||||||||||.||||||||....|||||....|.||||||||||||||||.|||||||

DIVLTQTPAIMSASPGEKVTMTCSASLSVSYMHWYQQKSSTSPKLWIYDTSKLASGVPGRFSGSGSGNSYSLTISSMEAEDVATYYCFQGSGYPFTFGSGTKLEIK

^^^^^ ^^^ ^^^^^^^^^

Best Alignment of Palivizumab CDR-H3 to a sequence from OAS

QVTLRESGPALVKPTQTLTLTCTFSGFSLSTSGMSVGWIRQPPGKALEWLADIWWD-DKKDYNPSLKSRLTISKDTSKNQVVLKVTNMDPADTATYYCARSMITNWYFDVWGAGTTVTVSS

||.|......||.|.....|.|..||...........|..|.||..|||...|........||...|...|...|.|..............|.|.|||||||||||||||||.||||||||

QVQLQQPVAELVMPGASVKLSCKASGYTF--TSYWMHWVKQRPGQGLEWIGEIDPSDSYTNYNQKFKGKSTLTVDKSSSTAYMQLSSLTSEDSAVYYCARSMITNWYFDVWGTGTTVTVSS

^^^^^^^^^^ ^^^^^^^^ ^^^^^^^^^^^^

Therapeutic : Pamrevlumab

Best Alignment of Pamrevlumab heavy chain to a sequence from OAS

EGQLVQSGGGLVHPGGSLRLSCAGSGFTFSSYGMHWVRQAPGKGLEWVSGIGTGGGTYSTDSVKGRFTISRDNAKNSLYLQMNSLRAEDMAVYYCARGDYYG--SGSFFDCWGQGTLVTVSS

|.||||||||||||||||||||.|||||||||.||||||||||||||||.||||||||..|||||||||||||||||||||||||||||.|||||||..||...||...|.||.||||||||

EVQLVQSGGGLVHPGGSLRLSCSGSGFTFSSYAMHWVRQAPGKGLEWVSAIGTGGGTYYADSVKGRFTISRDNAKNSLYLQMNSLRAEDTAVYYCARDGYYYESSGYTLDYWGRGTLVTVSS

^^^^^^^^ ^^^^^^^ ^^^^^^^^^^^^^^^^

Best Alignment of Pamrevlumab light chain to a sequence from OAS

DIQMTQSPSSLSASVGDRVTITCRASQGISSWLAWYQQKPEKAPKSLIYAASSLQSGVPSRFSGSGSGTDFTLTISSLQPEDFATYYCQQYNSYPPTFGQGTKLEIK

|||||||||||||||||||||||||||||||||||||||||||||||||||||||||||||||||||||||||||||||||||||||||||||||||||||||||||

DIQMTQSPSSLSASVGDRVTITCRASQGISSWLAWYQQKPEKAPKSLIYAASSLQSGVPSRFSGSGSGTDFTLTISSLQPEDFATYYCQQYNSYPPTFGQGTKLEIK

^^^^^^ ^^^ ^^^^^^^^^

Best Alignment of Pamrevlumab heavy chain CDRs to a sequence from OAS

EGQLVQSGGGLVHPGGSLRLSCAGSGFTFSSYGMHWVRQAPGKGLEWVSGIGTGGGTYSTDSVKGRFTISRDNAKNSLYLQMNSLRAEDMAVYYCARGDYYGSGSFFDCWGQGTLVTVSS

................|||||||.||||||||.|||||||.||||||||.|||.|.||...||||||||||.|||||||||||||||.|.|||||||||||||||.||.||||.||||||

----------------SLRLSCAASGFTFSSYDMHWVRQATGKGLEWVSAIGTAGDTYYPGSVKGRFTISRENAKNSLYLQMNSLRAGDTAVYYCARGDYYGSGSYFDYWGQGALVTVSS

^^^^^^^^ ^^^^^^^ ^^^^^^^^^^^^^^

Best Alignment of Pamrevlumab light chain CDRs to a sequence from OAS

DIQMTQSPSSLSASVGDRVTITCRASQGISSWLAWYQQKPEKAPKSLIYAASSLQSGVPSRFSGSGSGTDFTLTISSLQPEDFATYYCQQYNSYPPTFGQGTKLEIK

|.||..|..|...|||||||||||||||||||||||||||.||||.|||||||||||||||||||||||||||||||||||||||||||||||||||||.|||.|||

DSQMRKSTASVPPSVGDRVTITCRASQGISSWLAWYQQKPGKAPKLLIYAASSLQSGVPSRFSGSGSGTDFTLTISSLQPEDFATYYCQQYNSYPPTFGEGTKVEIK

^^^^^^ ^^^ ^^^^^^^^^

Best Alignment of Pamrevlumab CDR-H3 to a sequence from OAS

EGQLVQSGGGLVHPGGSLRLSCAGSGFTFSSYGMHWVRQAPGKGLEWVSGIGTGGGTYSTDSVKGRFTISRDNAKNSLYLQMNSLRAEDMAVYYCARGDYYGSGSFFDCWGQGTLVTVSS

.................|.|.|..|....|.|...|.||.|||||||...|...|.|....|.|.|.|||.|...|...|...|..|.|.||||||||||||||||||.|||||||||||

--------------SETLSLTCTVSAGSISGYFWSWIRQPPGKGLEWIGYIYYSGSTNYNPSLKSRVTISVDTSNNQFSLKLSSVTAADKAVYYCARGDYYGSGSFFDYWGQGTLVTVSS

^^^^^^^^ ^^^^^^^ ^^^^^^^^^^^^^^

Therapeutic : Panitumumab

Best Alignment of Panitumumab heavy chain to a sequence from OAS

QVQLQESGPGLVKPSETLSLTCTVSGGSVSSGDYYWTWIRQSPGKGLEWIGHIYYSGNTNYNPSLKSRLTISIDTSKTQFSLKLSSVTAADTAIYYCVRDRVT-GAFDIWGQGTMVTVSS

||||||||||||||||||||||||||||||||.|||.||||.|||||||||.|||||.||||||||||.|||.||||.|||||||||||||||.|||.||.||.||||||||||||||||

QVQLQESGPGLVKPSETLSLTCTVSGGSVSSGSYYWSWIRQPPGKGLEWIGYIYYSGSTNYNPSLKSRVTISVDTSKNQFSLKLSSVTAADTAVYYCARDLVTQGAFDIWGQGTMVTVSS

^^^^^^^^^^ ^^^^^^^ ^^^^^^^^^^^^

Best Alignment of Panitumumab light chain to a sequence from OAS

DIQMTQSPSSLSASVGDRVTITCQASQDISNYLNWYQQKPGKAPKLLIYDASNLETGVPSRFSGSGSGTDFTFTISSLQPEDIATYFCQHFDHLPLAFGGGTKVEIK

||||||||||||||||||||||||||||||||||||||||||||||||||||||||||||||||||||||||||||||||||||||||||.|.|||.||||||||||

DIQMTQSPSSLSASVGDRVTITCQASQDISNYLNWYQQKPGKAPKLLIYDASNLETGVPSRFSGSGSGTDFTFTISSLQPEDIATYFCQHYDNLPLTFGGGTKVEIK

^^^^^^ ^^^ ^^^^^^^^^

Best Alignment of Panitumumab heavy chain CDRs to a sequence from OAS

QVQLQESGPGLVKPSETLSLTCTVSGGSVSSGDYYWTWIRQSPGKGLEWIGHIYYSGNTNYNPSLKSRLTISIDTSKTQFSLKLSSVTAADTAIYYCVRDRVTGAFDIWGQGTMVTVSS

|||||||||||||||.||||||||||||||||||||.||||.||||||.||.|||||||.||||||||.|||.||||.||||||.||||||||.|||.||..|||||||||||||||||

QVQLQESGPGLVKPSQTLSLTCTVSGGSVSSGDYYWSWIRQPPGKGLECIGYIYYSGNTYYNPSLKSRVTISVDTSKNQFSLKLNSVTAADTAVYYCARDGETGAFDIWGQGTMVTVSS

^^^^^^^^^^ ^^^^^^^ ^^^^^^^^^^^

Best Alignment of Panitumumab light chain CDRs to a sequence from OAS

DIQMTQSPSSLSASVGDRVTITCQASQDISNYLNWYQQKPGKAPKLLIYDASNLETGVPSRFSGSGSGTDFTFTISSLQPEDIATYFCQHFDHLPLAFGGGTKVEIK

||||||||||||||||||||||||||||||||||||||.||||||||||||||||.||||||||.||||.|.||||.|||||||||.|||||||||.|||||..|||

DIQMTQSPSSLSASVGDRVTITCQASQDISNYLNWYQQRPGKAPKLLIYDASNLESGVPSRFSGRGSGTHFSFTISGLQPEDIATYYCQHFDHLPLTFGGGTRLEIK

^^^^^^ ^^^ ^^^^^^^^^

Best Alignment of Panitumumab CDR-H3 to a sequence from OAS

QVQLQESGPGLVKPSETLSLTCTVSGGSVSSGDYYWTWIRQSPGKGLEWIGHIYYS-GNTNYNPSLKSRLTISIDTSKTQFSLKLSSVTAADTAIYYCVRDRVTGAFDIWGQGTMVTVSS

.................|.|.|.||........|...|.||.||||||||..|....|...|..|...|.|||.|.......|...|..|.|||.|.||||||.||||||||||||||||

--------------GGSLRLSCAVSEFTF--SSYDMNWVRQAPGKGLEWISFITTRSGTIYYADSVRGRFTISRDNAQNSLYLQMNSLRAEDTAVYHCVRDRVGGAFDIWGQGTMVTVSS

^^^^^^^^^^ ^^^^^^^^ ^^^^^^^^^^^

Therapeutic : Panobacumab

Best Alignment of Panobacumab heavy chain to a sequence from OAS

EEQVVESGGGFVQPGGSLRLSCAASGFTFSPYWMHWVRQAPGKGLVWVSRINSD-GSTYYADSVKGRFTISRDNARNTLYLQMNSLRAEDTAVYYCARDRY--------------YGPEMWGQGTMVTVSS

|.|.||||||||||||||||||||||||||.|||||||||||||||||||||||..||.||||||||||||||||.|||||||||||||||||||||||||..............||...|||||.|||||

EVQLVESGGGFVQPGGSLRLSCAASGFTFSSYWMHWVRQAPGKGLVWVSRINSDGSSTSYADSVKGRFTISRDNAKNTLYLQMNSLRAEDTAVYYCARDRYYDFWSGYYPFDYYYYGMDVWGQGTTVTVSS

^^^^^^^^ ^^^^^^^^ ^^^^^^^^^^^^^^^^^^^^^^^^

Best Alignment of Panobacumab light chain to a sequence from OAS

DVVMTQSPLSLPVTLGQPASISCRSSQSLVYSDGNTYLNWFQQRPGQSPRRLIYKVSNRDSGVPDRFSGSGSGTDFTLKISRVEAEDVGVYYCMQGTHWPLTFGGGTKVEIK

||||||||||||||||||||||||||||||||||||||||||||||||||||||||||||||||||||||||||||||||||||||||||||||||||||||||||||||||

DVVMTQSPLSLPVTLGQPASISCRSSQSLVYSDGNTYLNWFQQRPGQSPRRLIYKVSNRDSGVPDRFSGSGSGTDFTLKISRVEAEDVGVYYCMQGTHWPLTFGGGTKVEIK

^^^^^^^^^^^ ^^^ ^^^^^^^^^

Best Alignment of Panobacumab heavy chain CDRs to a sequence from OAS

EEQVVESGGGFVQPGGSLRLSCAASGFTFSPYWMHWVRQAPGKGLVWVSRINSDGSTYYADSVKGRFTISRDNARNTLYLQMNSLRAEDTAVYYCARDRYYGPEMWGQGTMVTVSS

..............||||||||||||||||.|||||||.||||||||||||||||||.||||||||||||||||.|||||||||||||||||||||||||||...|||||.||||.

--------------GGSLRLSCAASGFTFSTYWMHWVRHAPGKGLVWVSRINSDGSTTYADSVKGRFTISRDNAKNTLYLQMNSLRAEDTAVYYCARDRYYGMDVWGQGTTVTVS-

^^^^^^^^ ^^^^^^^ ^^^^^^^^^^

Best Alignment of Panobacumab light chain CDRs to a sequence from OAS

DVVMTQSPLSLPVTLGQPASISCRSSQSLVYSDGNTYLNWFQQRPGQSPRRLIYKVSNRDSGVPDRFSGSGSGTDFTLKISRVEAEDVGVYYCMQGTHWPLTFGGGTKVEIK

|||||||||||||||||||||||||||||||||||||||||||||||||||||||||||||||||||||||||||||||||||||||||.||||||||||||||.|||.|||

DVVMTQSPLSLPVTLGQPASISCRSSQSLVYSDGNTYLNWFQQRPGQSPRRLIYKVSNRDSGVPDRFSGSGSGTDFTLKISRVEAEDVGIYYCMQGTHWPLTFGQGTKPEIK

^^^^^^^^^^^ ^^^ ^^^^^^^^^

Best Alignment of Panobacumab CDR-H3 to a sequence from OAS

EEQVVESGGGFVQPGGSLRLSCAASGFTF--SPYWMHWVRQAPGKGLVWVSRINSDGSTYYADSVKGRFTISRDNARNTLYLQMNSLRAEDTAVYYCARDRYYGPEMWGQGTMVTVSS

..|..|||.|.|.|...|.|.|..||.......|...|.||..||||.|..||...|||.|..|.|.|.|||.|...|...|...|..|.|||||||||||||||..|||||.|||||

QVQLQESGPGLVKPSQTLSLTCTVSGGSISSGSYYWSWIRQPAGKGLEWIGRIYTSGSTNYNPSLKSRVTISVDTSKNQFSLKLSSVTAADTAVYYCARDRYYGPLDWGQGTLVTVSS

^^^^^^^^^^ ^^^^^^^ ^^^^^^^^^^

Therapeutic : Parsatuzumab

Best Alignment of Parsatuzumab heavy chain to a sequence from OAS

EVQLVESGGGLVQPGGSLRLSCAASGYTFIDYYMNWVRQAPGKGLEWVGDINLDNSGTHYNQKFKGRFTISRDKSKNTAYLQMNSLRAEDTAVYYCAREGVYHD-----YDDYAMDYWGQGTLVTVSS

||||||||||||||||||||||||||.||..|.|.||||||||||.||..||.|.|.|.|....|||||||||..|||.|||||||||||||||||||||.|.|.....||....|||||||||||||

EVQLVESGGGLVQPGGSLRLSCAASGFTFSSYWMHWVRQAPGKGLVWVSRINSDGSSTSYADSVKGRFTISRDNAKNTLYLQMNSLRAEDTAVYYCAREGTYYDFWSGYYDRGCSDYWGQGTLVTVSS

^^^^^^^^ ^^^^^^^^ ^^^^^^^^^^^^^^^^^^^^^

Best Alignment of Parsatuzumab light chain to a sequence from OAS

DIQMTQSPSSLSASVGDRVTITCRTSQSLVHINAITYLHWYQQKPGKAPKLLIYRVSNRFSGVPSRFSGSGSGTDFTLTISSLQPEDFATYYCGQSTHVPLTFGQGTKVEIK

||||||||||||||||||||||||.||||......|||.|||||||||||||||..|...|||||||||||||||||||||||||||||||||.||...|||||||||||||

DIQMTQSPSSLSASVGDRVTITCRASQSL-----KTYLNWYQQKPGKAPKLLIYAASTLQSGVPSRFSGSGSGTDFTLTISSLQPEDFATYYCQQSYSTPLTFGQGTKVEIK

^^^^^^^^^^^ ^^^ ^^^^^^^^^

Best Alignment of Parsatuzumab heavy chain CDRs to a sequence from OAS

EVQLVESGGGLVQPGGSLRLSCAASGYTFIDYYMNWVRQAPGKGLEWVGDINLDNSGTHYNQKFKGRFTISRDKSKNTAYLQMNSLRAEDTAVYYCAREGVYHDYDDYAMDYWGQGTLVTVSS

||||..||..||.||.|...||.||||||.|||||||.|..||.|||.||||..|.||.|||||||..|...|||..|||....||..||.|||||||||.|.||||||||||||||.|||||

EVQLQQSGPELVKPGASVKISCKASGYTFTDYYMNWVKQSHGKSLEWIGDINPNNGGTSYNQKFKGKATLTVDKSSSTAYMELRSLTSEDSAVYYCAREGGYYDYDDYAMDYWGQGTSVTVSS

^^^^^^^^ ^^^^^^^^ ^^^^^^^^^^^^^^^^

Best Alignment of Parsatuzumab light chain CDRs to a sequence from OAS

DIQMTQSPSSLSASVGDRVTITCRTSQSLVHINAITYLHWYQQKPGKAPKLLIYRVSNRFSGVPSRFSGSGSGTDFTLTISSLQPEDFATYYCGQSTHVPLTFGQGTKVEIK

|..|||||.||....|....|.||.||||||.|..|||.||||||...|..|||.||||.||.|.||||||.||||||.||....||...||||||||||||||.|||||||

DVVMTQSPLSLPITPGQPASISCRSSQSLVHSNGNTYLNWYQQKPCQPPRRLIYQVSNRDSGGPDRFSGSGAGTDFTLNISRVESEDVGVYYCGQSTHVPLTFGGGTKVEIK

^^^^^^^^^^^ ^^^ ^^^^^^^^^

Best Alignment of Parsatuzumab CDR-H3 to a sequence from OAS

EVQLVESGGGLVQPGGSLRLSCAASGYTFIDYYMNWVRQAPGKGLEWVGDINLDNSGTHYNQKFKGRFTISRDKSKNTAYLQMNSLRAEDTAVYYCAREGVYHDYDDYAMDYWGQGTLVTVSS

||.||||||||||||||..|||||||.||....|.||||||.||||||..|....|...|....|||||||||..|||..|||.|||.||||.|||||||||.||||||||||||||.|||||

EVMLVESGGGLVQPGGSRKLSCAASGFTFSSFGMHWVRQAPEKGLEWVAYISSGSSTIYYADTVKGRFTISRDNPKNTLFLQMTSLRSEDTAMYYCAREGVYYDYDDYAMDYWGQGTSVTVSS

^^^^^^^^ ^^^^^^^^ ^^^^^^^^^^^^^^^^

Therapeutic : Pateclizumab

Best Alignment of Pateclizumab heavy chain to a sequence from OAS

EVQLVESGGGLVQPGGSLRLSCAASGYTFTSYVIHWVRQAPGKGLEWVGYNNPYNAGTNYNEKFKGRFTISSDKSKNTAYLQMNSLRAEDTAVYYCSRPTML---------PWFAYWGQGTLVTVSS

||||||||||||||||||||||||||.||.||..|||||||||||.||...|.....|.|....|||||||.|.||||.|||||||||||||||||.|...|.........|.|.||||||||||||

EVQLVESGGGLVQPGGSLRLSCAASGFTFSSYWMHWVRQAPGKGLVWVSRINSDGSSTSYADSVKGRFTISRDNSKNTLYLQMNSLRAEDTAVYYCARYGPLEGVAAATQVPYFDYWGQGTLVTVSS

^^^^^^^^ ^^^^^^^^ ^^^^^^^^^^^^^^^^^^^^

Best Alignment of Pateclizumab light chain to a sequence from OAS

DIQMTQSPSSLSASVGDRVTITCRASQAVSSAVAWYQQKPGKAPKLLIYSASHRYTGVPSRFSGSGSGTDFTLTISSLQPEDFATYYCQESYSTPWTFGQGTKVEIK

|||||||||||||||||||||||||||..|||.||||||||||||||||.||....|||||||||||||||||||||||||||||||||.|||||||||||||||||

DIQMTQSPSSLSASVGDRVTITCRASQNISSALAWYQQKPGKAPKLLIYDASSLESGVPSRFSGSGSGTDFTLTISSLQPEDFATYYCQQSYSTPWTFGQGTKVEIK

^^^^^^ ^^^ ^^^^^^^^^

Best Alignment of Pateclizumab heavy chain CDRs to a sequence from OAS

EVQLVESGGGLVQPGGSLRLSCAASGYTFTSYVIHWVRQAPGKGLEWVGYNNPYNAGTNYNEKFKGRFTISSDKSKNTAYLQMNSLRAEDTAVYYCSRPTMLPWFAYWGQGTLVTVSS

||||..||..||.||.|...||.||||||||||.|||.|.||.||||.||.||||.||.|||||||..|..||||..|||....||..||.|||||.||||..||||||||||||||.

EVQLQQSGPELVKPGASVKMSCKASGYTFTSYVMHWVKQKPGQGLEWIGYINPYNDGTKYNEKFKGKATLTSDKSSSTAYMELSSLTSEDSAVYYCARPTMTTWFAYWGQGTLVTVSA

^^^^^^^^ ^^^^^^^^ ^^^^^^^^^^^

Best Alignment of Pateclizumab light chain CDRs to a sequence from OAS

DIQMTQSPSSLSASVGDRVTITCRASQAVSSAVAWYQQKPGKAPKLLIYSASHRYTGVPSRFSGSGSGTDFTLTISSLQPEDFATYYCQESYSTPWTFGQGTKVEIK

||||||||||||||||||||.||||||.||||.|||.|||||||||||||||....||.||||||||||||||||||||||||||||||||||||.|||.|||||||

DIQMTQSPSSLSASVGDRVTVTCRASQGVSSALAWYHQKPGKAPKLLIYSASTLESGVASRFSGSGSGTDFTLTISSLQPEDFATYYCQESYSTPHTFGGGTKVEIK

^^^^^^ ^^^ ^^^^^^^^^

Best Alignment of Pateclizumab CDR-H3 to a sequence from OAS

EVQLVESGGGLVQPGGSLRLSCAASGYTFTSYVIHWVRQAPGKGLEWVGYNNPYNAGTNYNEKFKGRFTISSDKSKNTAYLQMNSLRAEDTAVYYCSRPTMLPWFAYWGQGTLVTVSS

.|||..||..|..||.|...||.|.||||.||.|.||.|.||.||||.|...|....|||||||||..|...|.|.||||.|..||..||.|||||||.||.|||||||||||||||.

-VQLQQSGAELMKPGASVKISCKATGYTFSSYWIEWVKQRPGHGLEWIGEILPGSGSTNYNEKFKGKATFTADTSSNTAYMQLSSLTSEDSAVYYCSRSTMIPWFAYWGQGTLVTVSA

^^^^^^^^ ^^^^^^^^ ^^^^^^^^^^^

Therapeutic : Patritumab

Best Alignment of Patritumab heavy chain to a sequence from OAS

QVQLQQWGAGLLKPSETLSLTCAVYGGSFSGYYWSWIRQPPGKGLEWIGEINHSGSTNYNPSLKSRVTISVETSKNQFSLKLSSVTAADTAVYYCARDKWT-------WYFDLWGRGTLVTVSS

|||||||||||||||||||||||||||||||||||||||||||||||||||||||||||||||||||||||.|||||||||||||||||||||||||||||.......||||||||||||||||

QVQLQQWGAGLLKPSETLSLTCAVYGGSFSGYYWSWIRQPPGKGLEWIGEINHSGSTNYNPSLKSRVTISVDTSKNQFSLKLSSVTAADTAVYYCARDKWTSNWGPRYWYFDLWGRGTLVTVSS

^^^^^^^^ ^^^^^^^ ^^^^^^^^^^^^^^^^^^

Best Alignment of Patritumab light chain to a sequence from OAS

DIEMTQSPDSLAVSLGERATINCRSSQSVLYSSSNRNYLAWYQQNPGQPPKLLIYWASTRESGVPDRFSGSGSGTDFTLTISSLQAEDVAVYYCQQYYSTPRTFGQGTKVEIK

||.||||||||||||||||||||||||||||||.||||||||||.||||||||||||||||||||||||||||||||||||||||||||||||||||||||||||||||||||

DIVMTQSPDSLAVSLGERATINCRSSQSVLYSSDNRNYLAWYQQKPGQPPKLLIYWASTRESGVPDRFSGSGSGTDFTLTISSLQAEDVAVYYCQQYYSTPRTFGQGTKVEIK

^^^^^^^^^^^^ ^^^ ^^^^^^^^^

Best Alignment of Patritumab heavy chain CDRs to a sequence from OAS

QVQLQQWGAGLLKPSETLSLTCAVYGGSFSGYYWSWIRQPPGKGLEWIGEINHSGSTNYNPSLKSRVTISVETSKNQFSLKLSSVTAADTAVYYCARDKWTWYFDLWGRGTLVTVSS

...............|||||||||||||||||||.||||||||||||||||||||||||||||||||||||.||||||||||||||||||||||||||.|.||||||||||||||||

---------------ETLSLTCAVYGGSFSGYYWRWIRQPPGKGLEWIGEINHSGSTNYNPSLKSRVTISVDTSKNQFSLKLSSVTAADTAVYYCARDSWYWYFDLWGRGTLVTVSS

^^^^^^^^ ^^^^^^^ ^^^^^^^^^^^

Best Alignment of Patritumab light chain CDRs to a sequence from OAS

DIEMTQSPDSLAVSLGERATINCRSSQSVLYSSSNRNYLAWYQQNPGQPPKLLIYWASTRESGVPDRFSGSGSGTDFTLTISSLQAEDVAVYYCQQYYSTPRTFGQGTKVEIK

||.|||||||||||||||||.||.|||||||||.||||||||||.|||.|||||.||||||||||||||||||||||||||||||||||||||||||||||||||||||.|||

DIVMTQSPDSLAVSLGERATVNCKSSQSVLYSSNNRNYLAWYQQRPGQSPKLLISWASTRESGVPDRFSGSGSGTDFTLTISSLQAEDVAVYYCQQYYSTPRTFGQGTKLEIK

^^^^^^^^^^^^ ^^^ ^^^^^^^^^

Best Alignment of Patritumab CDR-H3 to a sequence from OAS

QVQLQQWGAGLLKPSETLSLTCAVYGGSFSGYYWSWIRQPPGKGLEWIGEINHS-GSTNYNPSLKSRVTISVETSKNQFSLKLSSVTAADTAVYYCARDKWTWYFDLWGRGTLVTVSS

||||.|.||...||.......|...|..|..||..|.||..|.||||.|.||...|.|.|......|||....||.......||.....||||||||||||.||||||||||||||||

QVQLVQSGAEVKKPGASVKVSCKASGYTFTAYYLHWVRQASGQGLEWMGWINPNNGDTIYAQNFQGRVTMTRDTSISTAYMELSNLRSDDTAVYYCARDKWFWYFDLWGRGTLVTVSS

^^^^^^^^ ^^^^^^^^ ^^^^^^^^^^^

Therapeutic : Pembrolizumab

Best Alignment of Pembrolizumab heavy chain to a sequence from OAS

QVQLVQSGVEVKKPGASVKVSCKASGYTFTNYYMYWVRQAPGQGLEWMGGINPSNGGTNFNEKFKNRVTLTTDSSTTTAYMELKSLQFDDTAVYYCARRDYRFDMGFDYWGQGTTVTVSS

||||||||.|||||||||||||||||||||.|||.||||||||||||||.|||..||||...|...|||.|||.||.||||||.||..||||||||||.||..|.|.|||||||.|||||

QVQLVQSGAEVKKPGASVKVSCKASGYTFTGYYMHWVRQAPGQGLEWMGRINPNSGGTNYAQKLQGRVTMTTDTSTSTAYMELRSLRSDDTAVYYCARDDYG-DYGGDYWGQGTLVTVSS

^^^^^^^^ ^^^^^^^^ ^^^^^^^^^^^^^

Best Alignment of Pembrolizumab light chain to a sequence from OAS

EIVLTQSPATLSLSPGERATLSCRASKGVSTSGYSYLHWYQQKPGQAPRLLIYLASYLESGVPARFSGSGSGTDFTLTISSLEPEDFAVYYCQHSRDLPLTFGGGTKVEIK

||||||||||||||||||||||||||.||.....|||.|||||||||||||||.||....|.||||||||||||||||||||||||||||||||..|.|||||||||||||

EIVLTQSPATLSLSPGERATLSCRASQGV----RSYLAWYQQKPGQAPRLLIYDASNRATGIPARFSGSGSGTDFTLTISSLEPEDFAVYYCQHRDDWPLTFGGGTKVEIK

^^^^^^^^^^ ^^^ ^^^^^^^^^

Best Alignment of Pembrolizumab heavy chain CDRs to a sequence from OAS

QVQLVQSGVEVKKPGASVKVSCKASGYTFTNYYMYWVRQAPGQGLEWMGGINPSNGGTNFNEKFKNRVTLTTDSSTTTAYMELKSLQFDDTAVYYCARRDYRFDMGFDYWGQGTTVTVSS

.|||.|||....|||||||.||||||||||.|||.||.|..|.||||.|.|||.||||..|.|||...|||.|.|..||||||.||...|.|||.||||||.||.|||||||||.||||.

EVQLQQSGPVLVKPGASVKMSCKASGYTFTDYYMNWVKQSHGKGLEWIGVINPYNGGTSSNQKFKGKATLTVDKSSSTAYMELNSLTSEDAAVYSCARRDYGFDGGFDYWGQGTLVTVSA

^^^^^^^^ ^^^^^^^^ ^^^^^^^^^^^^^

Best Alignment of Pembrolizumab light chain CDRs to a sequence from OAS

EIVLTQSPATLSLSPGERATLSCRASKGVSTSGYSYLHWYQQKPGQAPRLLIYLASYLESGVPARFSGSGSGTDFTLTISSLEPEDFAVYYCQHSRDLPLTFGGGTKVEIK

.||||||||.|..|.|.|||.||||||.||||||||.|||||||||.|.|||||||.||||||||||||||||||||.|...|.||.|.|||||||.||||||.|||.|||

DIVLTQSPASLAVSLGQRATISCRASKSVSTSGYSYMHWYQQKPGQPPKLLIYLASNLESGVPARFSGSGSGTDFTLNIHPVEEEDAATYYCQHSRELPLTFGSGTKLEIK

^^^^^^^^^^ ^^^ ^^^^^^^^^

Best Alignment of Pembrolizumab CDR-H3 to a sequence from OAS

QVQLVQSGVEVKKPGASVKVSCKASGYTFTNYYMYWVRQAPGQGLEWMGGINPSNGGTNFNEKFKNRVTLTTDSSTTTAYMELKSLQFDDTAVYYCARRDYRFDMGFDYWGQGTTVTVSS

.|.||.||.....||.|.|.||.|||.||....|.||||||..||||...|.............|.|.|...|....|......||...|||.|||||||||.|.||||||||||.||||

EVKLVESGGGLVQPGGSRKLSCAASGFTFSSFGMHWVRQAPEKGLEWVAYISSGSSTIYYADTVKGRFTISRDNPKNTLFLQMTSLRSEDTAMYYCARRDYRYDVGFDYWGQGTTLTVSS

^^^^^^^^ ^^^^^^^^ ^^^^^^^^^^^^^

Therapeutic : Pertuzumab

Best Alignment of Pertuzumab heavy chain to a sequence from OAS

EVQLVESGGGLVQPGGSLRLSCAASGFTFTDYTMDWVRQAPGKGLEWVADVNPNSGGSIYNQRFKGRFTLSVDRSKNTLYLQMNSLRAEDTAVYYCARNLGP---SFYFDYWGQGTLVTVSS

|||||||||||||||.|||||||||||||.||.|.|||||||||||||.....|||...|....|||||.|.|.||||||||||||||||||||||||.||.....||||||||||||||||

EVQLVESGGGLVQPGRSLRLSCAASGFTFDDYAMHWVRQAPGKGLEWVSGISWNSGSIGYADSVKGRFTISRDNSKNTLYLQMNSLRAEDTAVYYCARDLGELELLFYFDYWGQGTLVTVSS

^^^^^^^^ ^^^^^^^^ ^^^^^^^^^^^^^^^

Best Alignment of Pertuzumab light chain to a sequence from OAS

DIQMTQSPSSLSASVGDRVTITCKASQDVSIGVAWYQQKPGKAPKLLIYSASYRYTGVPSRFSGSGSGTDFTLTISSLQPEDFATYYCQQYYIYPYTFGQGTKVEIK

|||||||||||||||||||||||.|||...|...|||||||||||.||||||...||||||||||||||||||||||||||||||||||||||||||||||||.|||

DIQMTQSPSSLSASVGDRVTITCRASQVIRIDLGWYQQKPGKAPKRLIYSASSLQTGVPSRFSGSGSGTDFTLTISSLQPEDFATYYCQQYYIYPYTFGQGTKLEIK

^^^^^^ ^^^ ^^^^^^^^^

Best Alignment of Pertuzumab heavy chain CDRs to a sequence from OAS

EVQLVESGGGLVQPGGSLRLSCAASGFTFTDYTMDWVRQAPGKGLEWVADVNPNSGGSIYNQRFKGRFTLSVDRSKNTLYLQMNSLRAEDTAVYYCARNLGPSFYFDYWGQGTLVTVSS

||||..||..||.||.|...||.|||.|||||.|.||.|..||.|||....|||.||..|||.|||..||.|..|..|.|....||..||.||||||||.|.|||||||||||..||||

EVQLQQSGPELVKPGASVKMSCKASGYTFTDYNMHWVKQSHGKSLEWIGYINPNNGGTSYNQKFKGKATLTVNKSSSTAYMELRSLTSEDSAVYYCARNYGSSFYFDYWGQGTTLTVSS

^^^^^^^^ ^^^^^^^^ ^^^^^^^^^^^^

Best Alignment of Pertuzumab light chain CDRs to a sequence from OAS

DIQMTQSPSSLSASVGDRVTITCKASQDVSIGVAWYQQKPGKAPKLLIYSASYRYTGVPSRFSGSGSGTDFTLTISSLQPEDFATYYCQQYYIYPYTFGQGTKVEIK

.........|||||||||||.|..|.||||...|..|||||||||....|||....||||.|||.||||||.|||..|||||||||||||||.||||||||||.|||

---------SLSASVGDRVTLTWRAGQDVSNYLALFQQKPGKAPKSVSSSASTLRSGVPSKFSGGGSGTDFALTITGLQPEDFATYYCQQYYTYPYTFGQGTKLEIK

^^^^^^ ^^^ ^^^^^^^^^

Best Alignment of Pertuzumab CDR-H3 to a sequence from OAS

EVQLVESGGGLVQPGGSLRLSCAASGFTFTDYTMDWVRQAPGKGLEWVADVNPNSGGSIYNQRFKGRFTLSVDRSKNTLYLQMNSLRAEDTAVYYCARNLGPSFYFDYWGQGTLVTVSS

.............|||||||||.||||||....|.||||||||||||||..........|....|||||.|.|..||.|||||||||||||||||||||||||.|||||||||||||||

-------------PGGSLRLSCEASGFTFSNDFMTWVRQAPGKGLEWVANMRVDGSDIHYVDSVKGRFTISRDNAKNSLYLQMNSLRAEDTAVYYCARNLGPSYYFDYWGQGTLVTVSS

^^^^^^^^ ^^^^^^^^ ^^^^^^^^^^^^

Therapeutic : Pidilizumab

Best Alignment of Pidilizumab heavy chain to a sequence from OAS

QVQLVQSGSELKKPGASVKISCKASGYTFTNYGMNWVRQAPGQGLQWMGWINTDSGESTYAEEFKGRFVFSLDTSVNTAYLQITSLTAEDTGMYFCVRVGY---------DALDYWGQGTLVTVSS

||||||||||||||||||||||||||||||||.||||||||||||.|||||||..|..|||..|.||||||||||||||||||.||.||||..|.|.|||..........||.|.|||||.|||||

QVQLVQSGSELKKPGASVKISCKASGYTFTNYAMNWVRQAPGQGLEWMGWINTNTGNPTYAQGFTGRFVFSLDTSVNTAYLQISSLKAEDTAFYYCARVGRFLEWFQTKDDAFDIWGQGTMVTVSS

^^^^^^^^ ^^^^^^^^ ^^^^^^^^^^^^^^^^^^^

Best Alignment of Pidilizumab light chain to a sequence from OAS

EIVLTQSPSSLSASVGDRVTITCSARSSVSYMHWFQQKPGKAPKLWIYRTSNLASGVPSRFSGSGSGTSYCLTINSLQPEDFATYYCQQRSSFPLTFGGGTKLEIK

.|||||||...|||.|..|||||||.||||||||||||||..||||||.|||||||||||||||||||||.|||||...||.||||||||||.|||||.|||||.|

-IVLTQSPAIMSASPGEKVTITCSASSSVSYMHWFQQKPGTSPKLWIYSTSNLASGVPSRFSGSGSGTSYSLTINSMEAEDAATYYCQQRSSYPLTFGAGTKLELK

^^^^^ ^^^ ^^^^^^^^^

Best Alignment of Pidilizumab heavy chain CDRs to a sequence from OAS

QVQLVQSGSELKKPGASVKISCKASGYTFTNYGMNWVRQAPGQGLQWMGWINTDSGESTYAEEFKGRFVFSLDTSVNTAYLQITSLTAEDTGMYFCVRVGYDALDYWGQGTLVTVSS

|.||||||.||||||..||||||||||||||||||||.||||.||.|||||||..||.|||..|||||.|||.||..||||||..|..||...|||.|.||||.|||||||.|||||

QIQLVQSGPELKKPGETVKISCKASGYTFTNYGMNWVKQAPGKGLKWMGWINTYTGEPTYADDFKGRFAFSLETSASTAYLQINNLKNEDMATYFCARRGYDAMDYWGQGTSVTVSS

^^^^^^^^ ^^^^^^^^ ^^^^^^^^^^

Best Alignment of Pidilizumab light chain CDRs to a sequence from OAS

EIVLTQSPSSLSASVGDRVTITCSARSSVSYMHWFQQKPGKAPKLWIYRTSNLASGVPSRFSGSGSGTSYCLTINSLQPEDFATYYCQQRSSFPLTFGGGTKLEIK

..||||.|...|||.|..|||||||.||||||||.|||||..||||||||||||||||.|||||||||||.|||.....||.||||||||||.|||||.|||||||

QMVLTQPPQIMSASPGEKVTITCSASSSVSYMHWYQQKPGTSPKLWIYRTSNLASGVPARFSGSGSGTSYSLTISRMEAEDAATYYCQQRSSYPLTFGAGTKLEIK

^^^^^ ^^^ ^^^^^^^^^

Best Alignment of Pidilizumab CDR-H3 to a sequence from OAS

QVQLVQSGSELKKPGASVKISCKASGYTF-TNYGMNWVRQAPGQGLQWMGWINTDSGESTYAEEFKGRFVFSLDTSVNTAYLQITSLTAEDTGMYFCVRVGYDALDYWGQGTLVTVSS

.....................|..||..........||||.||.||.|.|.|....|...|....|.|...|.|.|.|...|...|.||.||..|.|||.||||||||||||||||||

--------------SETLSLTCAVSGGFMSSGDWWSWVRQPPGKGLEWFGEIHHR-GSTNYHPSPKRRVTISVDQSKNQFPLTVNSVTAADTASYDCVRNGYDALDYWGQGTLVTVSS

^^^^^^^^^ ^^^^^^^^ ^^^^^^^^^^

Therapeutic : Pinatuzumab

Best Alignment of Pinatuzumab heavy chain to a sequence from OAS

EVQLVESGGGLVQPGGSLRLSCAASGYEFSRSWMNWVRQAPGKGLEWVGRIYPGDGDTNYSGKFKGRFTISADTSKNTAYLQMNSLRAEDTAVYYCARDGSSW--DWYFDVWGQGTLVTVSS

||||||||||||||||||||||||||..|||..|||||||||||||||..||.|.|||||.|..|||||||.|.||||.|||||||||||||||.|||||........||||||||||||||

EVQLVESGGGLVQPGGSLRLSCAASGFTFSRNAMNWVRQAPGKGLEWVSLIYSG-GDTNYAGSVKGRFTISRDGSKNTLYLQMNSLRAEDTAVYFCARDGGRDGYNLAFDVWGQGTLVTVSS

^^^^^^^^ ^^^^^^^^ ^^^^^^^^^^^^^^^

Best Alignment of Pinatuzumab light chain to a sequence from OAS

DIQMTQSPSSLSASVGDRVTITCRSSQSIVHSVGNTFLEWYQQKPGKAPKLLIYKVSNRFSGVPSRFSGSGSGTDFTLTISSLQPEDFATYYCFQGSQFPYTFGQGTKVEIK

||||||||||||||||||||||||.||||.....|..|.|||||||||||||||||||...||||||||||||||||||||||||||||||||.|...||.|||||||||||

DIQMTQSPSSLSASVGDRVTITCRASQSI-----NNYLNWYQQKPGKAPKLLIYKVSNLQNGVPSRFSGSGSGTDFTLTISSLQPEDFATYYCQQCYNFPWTFGQGTKVEIK

^^^^^^^^^^^ ^^^ ^^^^^^^^^

Best Alignment of Pinatuzumab heavy chain CDRs to a sequence from OAS

EVQLVESGGGLVQPGGSLRLSCAASGYEFSRSWMNWVRQAPGKGLEWVGRIYPGDGDTNYSGKFKGRFTISADTSKNTAYLQMNSLRAEDTAVYYCARDGSSWDWYFDVWGQGTLVTVSS

.|||..||..||.||.|...||.||||.||.||||||.|.|||||||.||||||||||||.|||||..|..||.|..|||.|..||..||.|||.|||||||.||||||||.||.|||||

QVQLQQSGPELVKPGASVKISCKASGYAFSSSWMNWVKQRPGKGLEWIGRIYPGDGDTNYNGKFKGKATLTADKSSSTAYMQLSSLTSEDSAVYFCARDGSSYDWYFDVWGTGTTVTVSS

^^^^^^^^ ^^^^^^^^ ^^^^^^^^^^^^^

Best Alignment of Pinatuzumab light chain CDRs to a sequence from OAS

DIQMTQSPSSLSASVGDRVTITCRSSQSIVHSVGNTFLEWYQQKPGKAPKLLIYKVSNRFSGVPSRFSGSGSGTDFTLTISSLQPEDFATYYCFQGSQFPYTFGQGTKVEIK

|..|||.|.||..|.||...|.||||||||||.|||.||||.||||..||||||||||||||||.|||||||||||||.||....||...||||||||.|||||.|||.|||

DVLMTQTPLSLPVSLGDQASISCRSSQSIVHSNGNTYLEWYLQKPGQSPKLLIYKVSNRFSGVPDRFSGSGSGTDFTLKISRVEAEDLGVYYCFQGSQVPYTFGGGTKLEIK

^^^^^^^^^^^ ^^^ ^^^^^^^^^

Best Alignment of Pinatuzumab CDR-H3 to a sequence from OAS

EVQLVESGGGLVQPGGSLRLSCAASGYEFSRSWMNWVRQAPGKGLEWVGRIYPGDGDTNYSGKFKGRFTISADTSKNTAYLQMNSLRAEDTAVYYCARDGSSWDWYFDVWGQGTLVTVSS

||.|||||||||.|||||.|||||||..||...|.||||||.||||||..|..|.....|....|||||||.|..|||..|||.|||.||||.||||||||||||||||||.||.|||||

EVMLVESGGGLVKPGGSLKLSCAASGFTFSDYGMHWVRQAPEKGLEWVAYISSGSSTIYYADTVKGRFTISRDNAKNTLFLQMTSLRSEDTAMYYCARDGSSWDWYFDVWGTGTTVTVSS

^^^^^^^^ ^^^^^^^^ ^^^^^^^^^^^^^

Therapeutic : Plozalizumab

Best Alignment of Plozalizumab heavy chain to a sequence from OAS

EVQLVESGGGLVKPGGSLRLSCAASGFTFSAYAMNWVRQAPGKGLEWVGRIRTKNNNYATYYADSVKDRFTISRDDSKNTLYLQMNSLKTEDTAVYYCTTFY----------GNGVWGQGTLVTVSS

||||||||||||||||||||||||||||||.|.|||||||.|||||||||||.|.|.|||.||.|||.||||||||||||||||||||||||||||||||............|..||||||.|||||

EVQLVESGGGLVKPGGSLRLSCAASGFTFSSYSMNWVRQASGKGLEWVGRIRSKANSYATAYAASVKGRFTISRDDSKNTLYLQMNSLKTEDTAVYYCTTDPPYSSDYYYYYGMDVWGQGTTVTVSS

^^^^^^^^ ^^^^^^^^^^ ^^^^^^^^^^^^^^^^^^

Best Alignment of Plozalizumab light chain to a sequence from OAS

DVVMTQSPLSLPVTLGQPASISCKSSQSLLDSDGKTFLNWFQQRPGQSPRRLIYLVSKLDSGVPDRFSGSGSGTDFTLKISRVEAEDVGVYYCWQGTHFPYTFGQGTRLEIK

|||||||||||||||||||||||.|||||..|||.|.|||||||||||||||||.|||.||||||||||||||||||||||||||||||||||.||||.|.|||||||||||

DVVMTQSPLSLPVTLGQPASISCRSSQSLVYSDGNTYLNWFQQRPGQSPRRLIYKVSKWDSGVPDRFSGSGSGTDFTLKISRVEAEDVGVYYCMQGTHWPPTFGQGTRLEIK

^^^^^^^^^^^ ^^^ ^^^^^^^^^

Best Alignment of Plozalizumab heavy chain CDRs to a sequence from OAS

EVQLVESGGGLVKPGGSLRLSCAASGFTFSAYAMNWVRQAPGKGLEWVGRIRTKNNNYATYYADSVKDRFTISRDDSKNTLYLQMNSLKTEDTAVYYCTTFYGNGVWGQGTLVTVSS

.|.|||||||||.||||..|||.|||||||.|.||||||.|.||||||..||.|.|||||.||.|||.||||||||||...|||||.|..|||..||||..|||.|||||||||||.

DVKLVESGGGLVQPGGSMKLSCVASGFTFSNYWMNWVRQSPEKGLEWVAEIRLKSNNYATHYAESVKGRFTISRDDSKSSVYLQMNNLRAEDTGIYYCTRTYGNYVWGQGTLVTVSA

^^^^^^^^ ^^^^^^^^^^ ^^^^^^^^

Best Alignment of Plozalizumab light chain CDRs to a sequence from OAS

DVVMTQSPLSLPVTLGQPASISCKSSQSLLDSDGKTFLNWFQQRPGQSPRRLIYLVSKLDSGVPDRFSGSGSGTDFTLKISRVEAEDVGVYYCWQGTHFPYTFGQGTRLEIK

||||.|.||.|.||.|||||||||||||||||||||||||..|||||||.|||||||||||||||||.|||||||||||||||||||.||||||||||||||||.||.||||
[truncated: 157,664 more chars]
